# Supplementary figures and images for: Cephalopod species identification using integrated analysis of machine learning and deep learning approaches (part 2 of 4)
Source: PeerJ. 2021 Aug 9;9:e11825. doi: 10.7717/peerj.11825 (PMC8359798; doi:10.7717/peerj.11825)

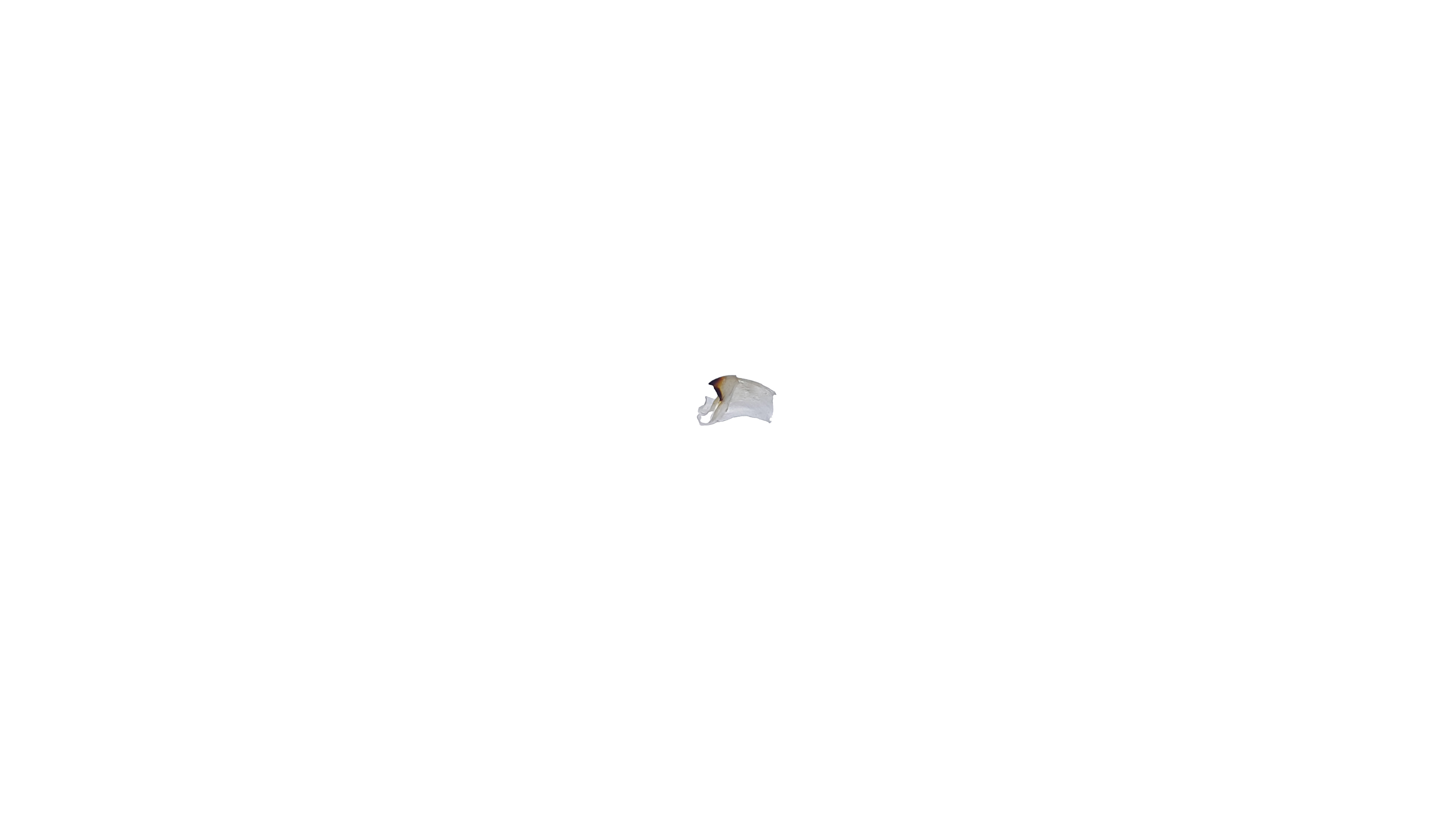

Supplement: Supplemental Information 1 — C2-Sepia aculeata, C3-Sepioteuthis lessoniana, C6-Sepia esculenta, O2-Amphioctopus aegina, S1-Loliolus uyii, S3-Uroteuthis chinensis, S4-Uroteuthis edulis [file peerj-09-11825-s001.zip › _Preprocessing_Lower_Beak/S1/L-l-S1-14.jpg]

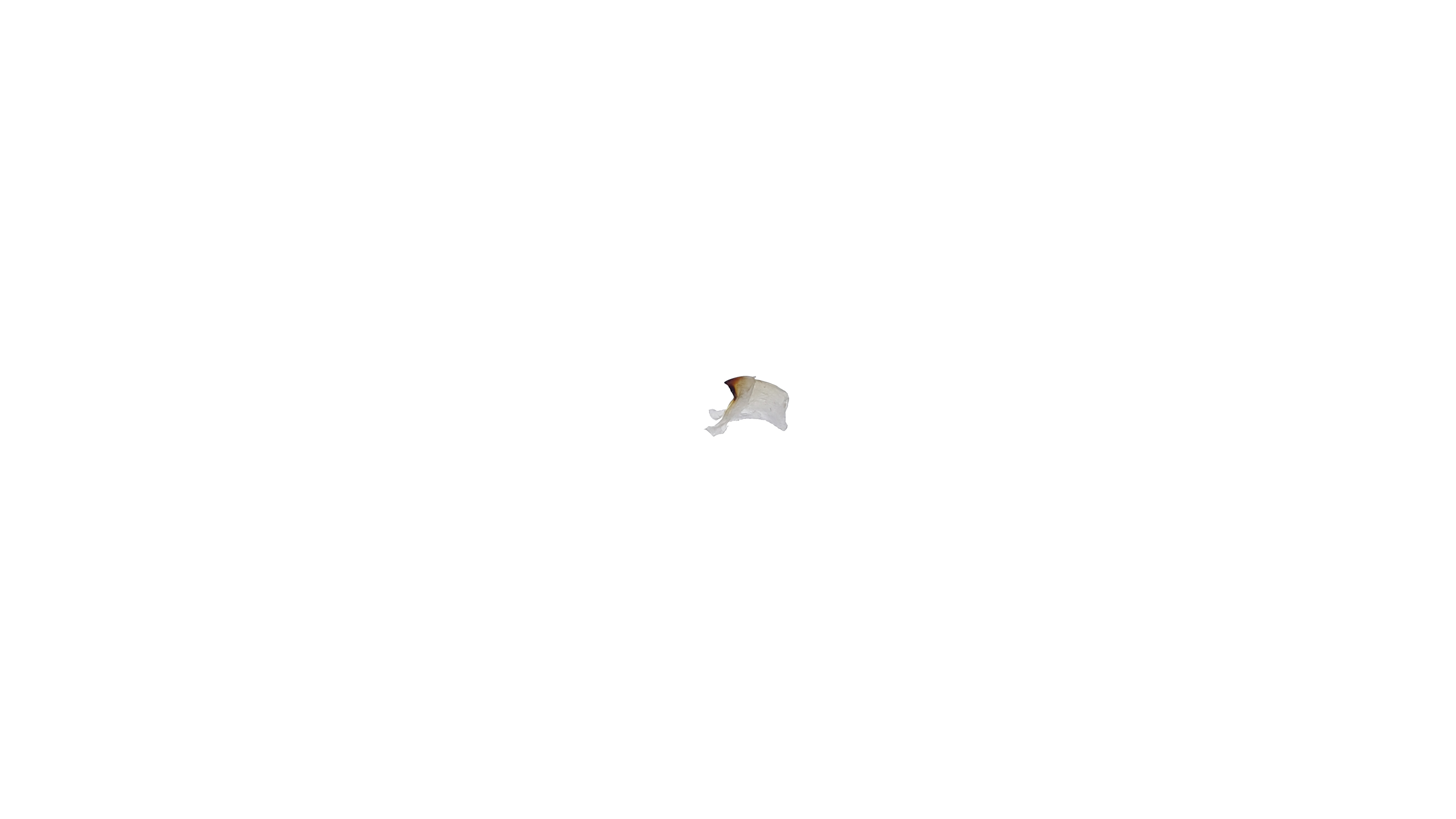

Supplement: Supplemental Information 1 — C2-Sepia aculeata, C3-Sepioteuthis lessoniana, C6-Sepia esculenta, O2-Amphioctopus aegina, S1-Loliolus uyii, S3-Uroteuthis chinensis, S4-Uroteuthis edulis [file peerj-09-11825-s001.zip › _Preprocessing_Lower_Beak/S1/L-l-S1-15.jpg]

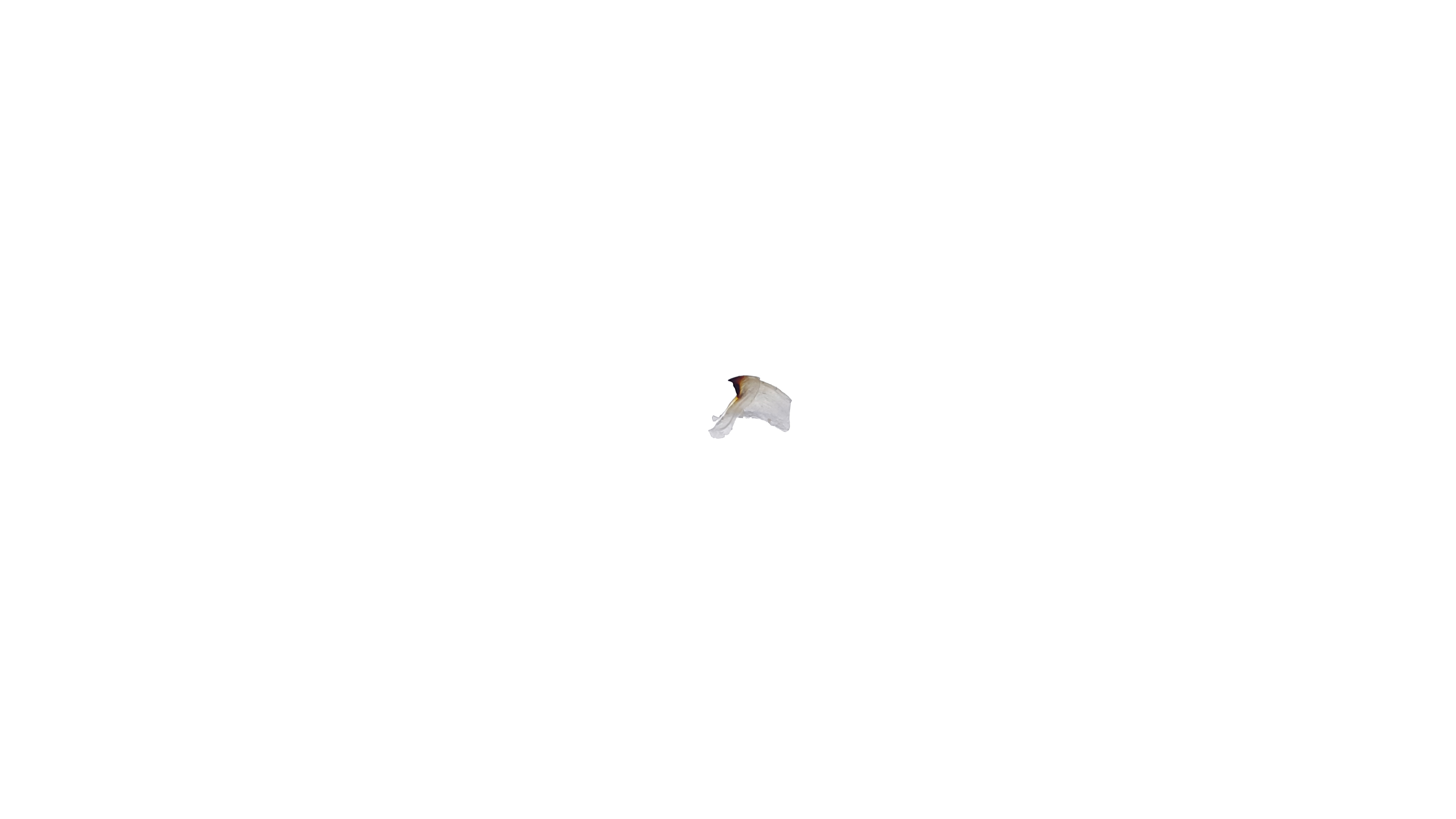

Supplement: Supplemental Information 1 — C2-Sepia aculeata, C3-Sepioteuthis lessoniana, C6-Sepia esculenta, O2-Amphioctopus aegina, S1-Loliolus uyii, S3-Uroteuthis chinensis, S4-Uroteuthis edulis [file peerj-09-11825-s001.zip › _Preprocessing_Lower_Beak/S1/L-l-S1-16.jpg]

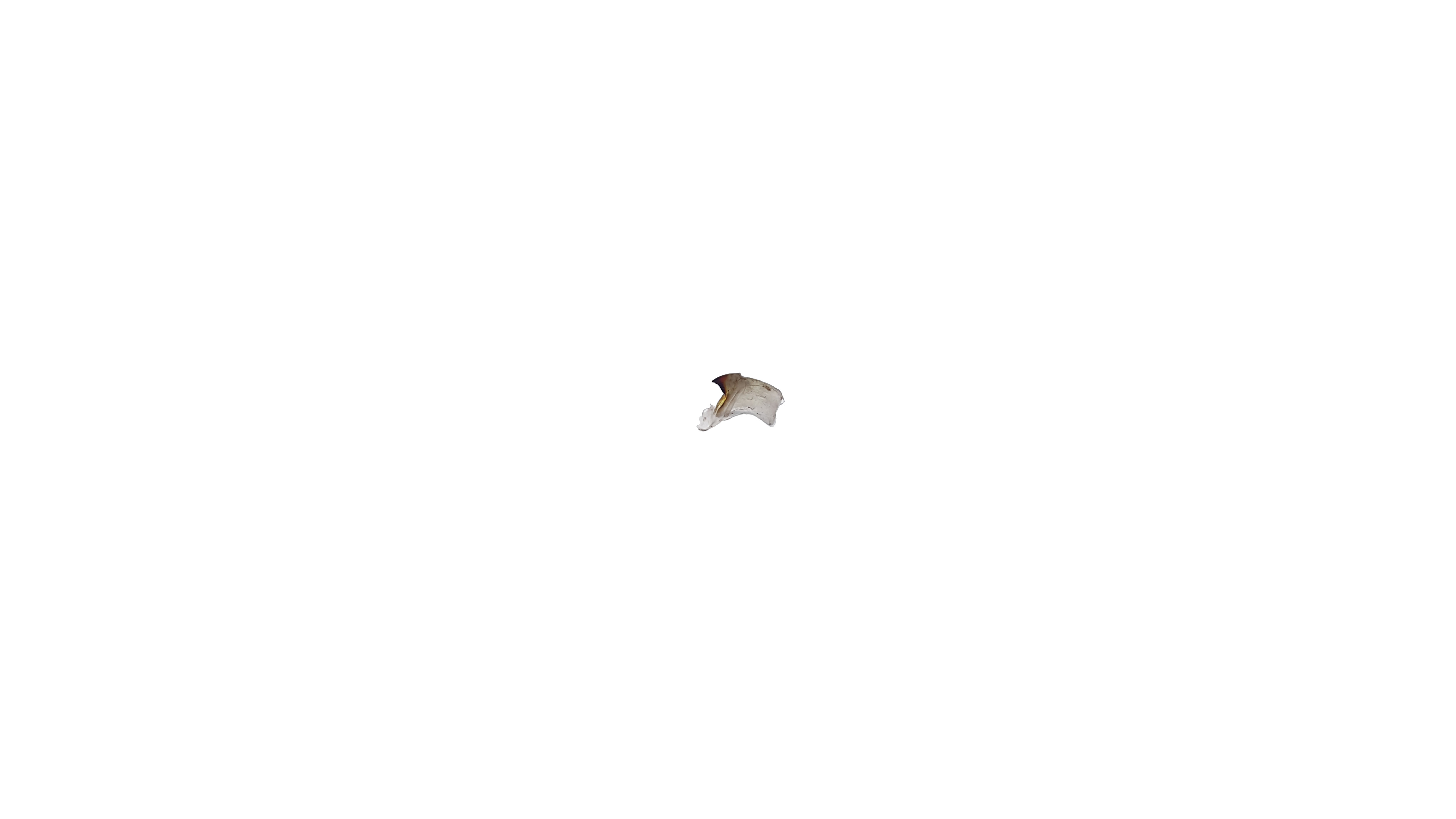

Supplement: Supplemental Information 1 — C2-Sepia aculeata, C3-Sepioteuthis lessoniana, C6-Sepia esculenta, O2-Amphioctopus aegina, S1-Loliolus uyii, S3-Uroteuthis chinensis, S4-Uroteuthis edulis [file peerj-09-11825-s001.zip › _Preprocessing_Lower_Beak/S1/L-l-S1-17.jpg]

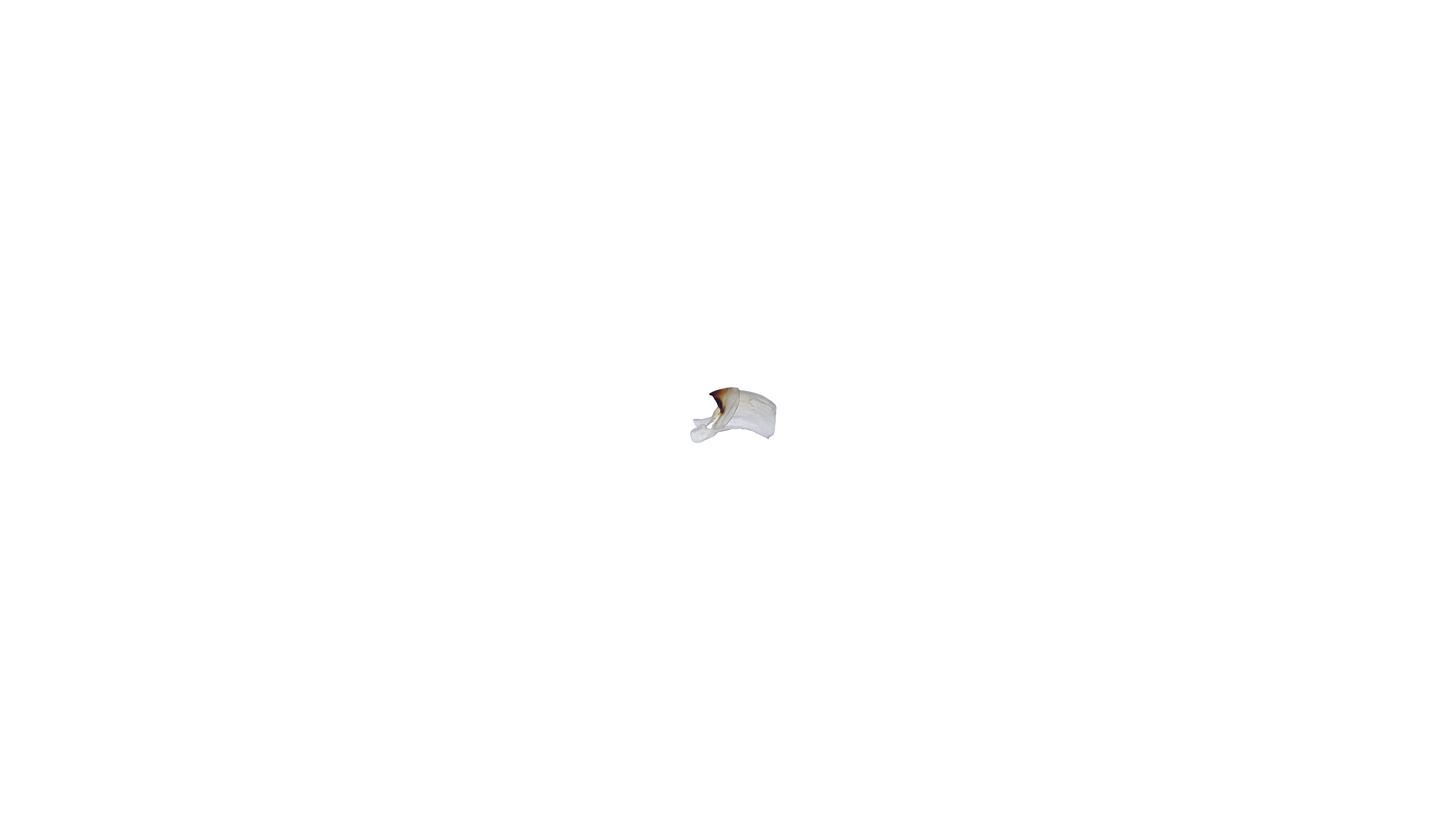

Supplement: Supplemental Information 1 — C2-Sepia aculeata, C3-Sepioteuthis lessoniana, C6-Sepia esculenta, O2-Amphioctopus aegina, S1-Loliolus uyii, S3-Uroteuthis chinensis, S4-Uroteuthis edulis [file peerj-09-11825-s001.zip › _Preprocessing_Lower_Beak/S1/L-l-S1-18.jpg]

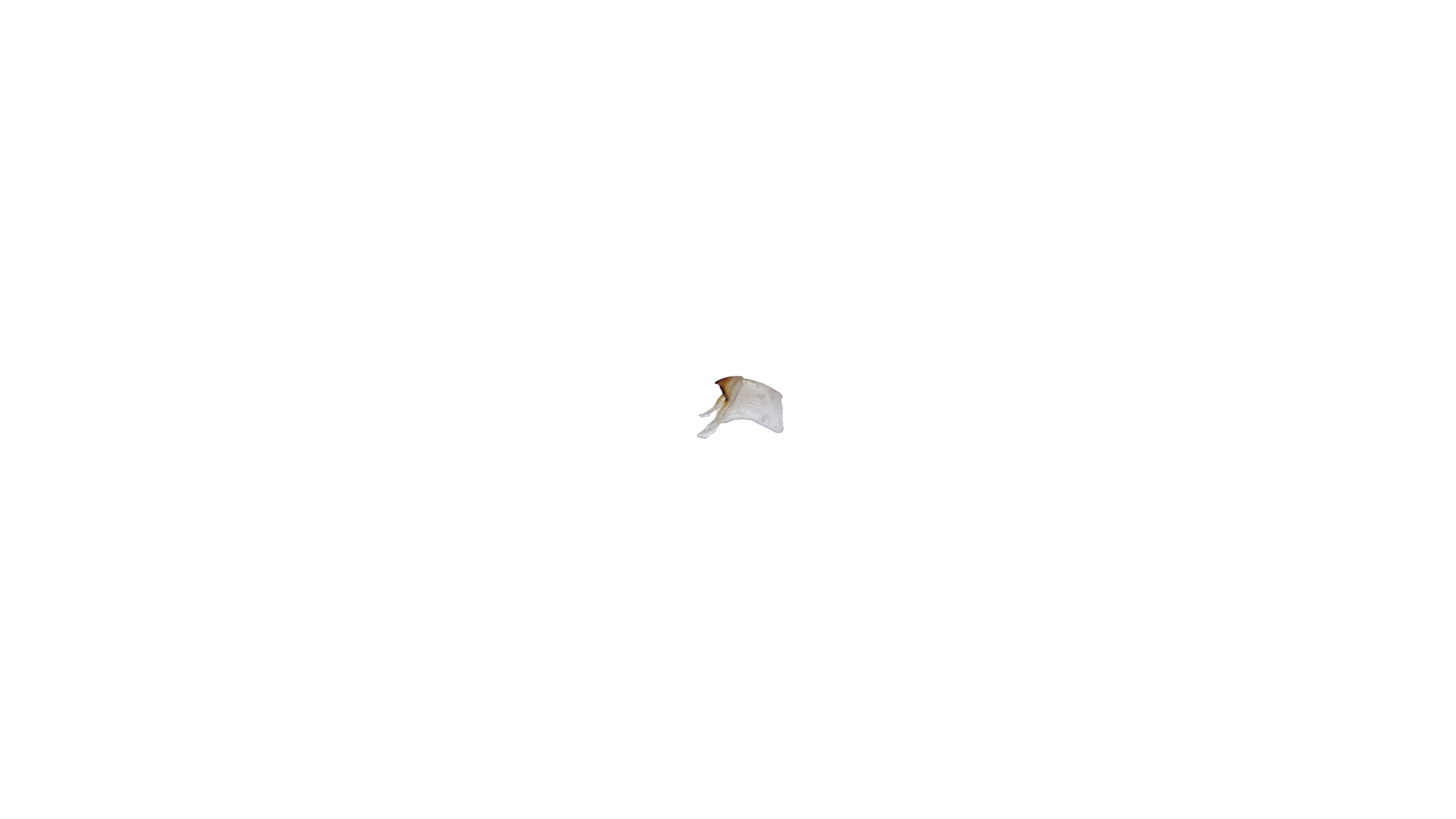

Supplement: Supplemental Information 1 — C2-Sepia aculeata, C3-Sepioteuthis lessoniana, C6-Sepia esculenta, O2-Amphioctopus aegina, S1-Loliolus uyii, S3-Uroteuthis chinensis, S4-Uroteuthis edulis [file peerj-09-11825-s001.zip › _Preprocessing_Lower_Beak/S1/L-l-S1-19.jpg]

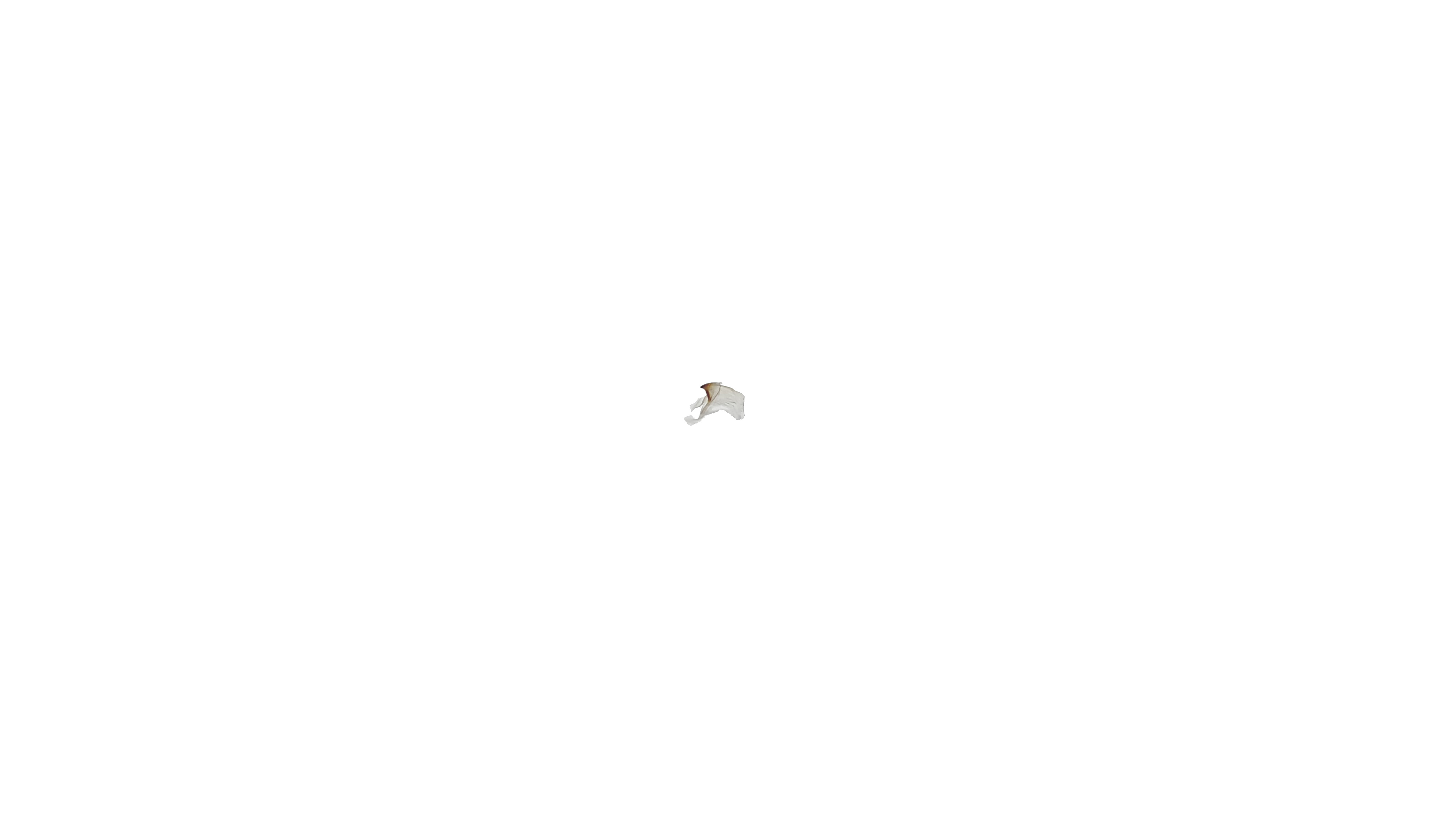

Supplement: Supplemental Information 1 — C2-Sepia aculeata, C3-Sepioteuthis lessoniana, C6-Sepia esculenta, O2-Amphioctopus aegina, S1-Loliolus uyii, S3-Uroteuthis chinensis, S4-Uroteuthis edulis [file peerj-09-11825-s001.zip › _Preprocessing_Lower_Beak/S1/L-l-S1-2.jpg]

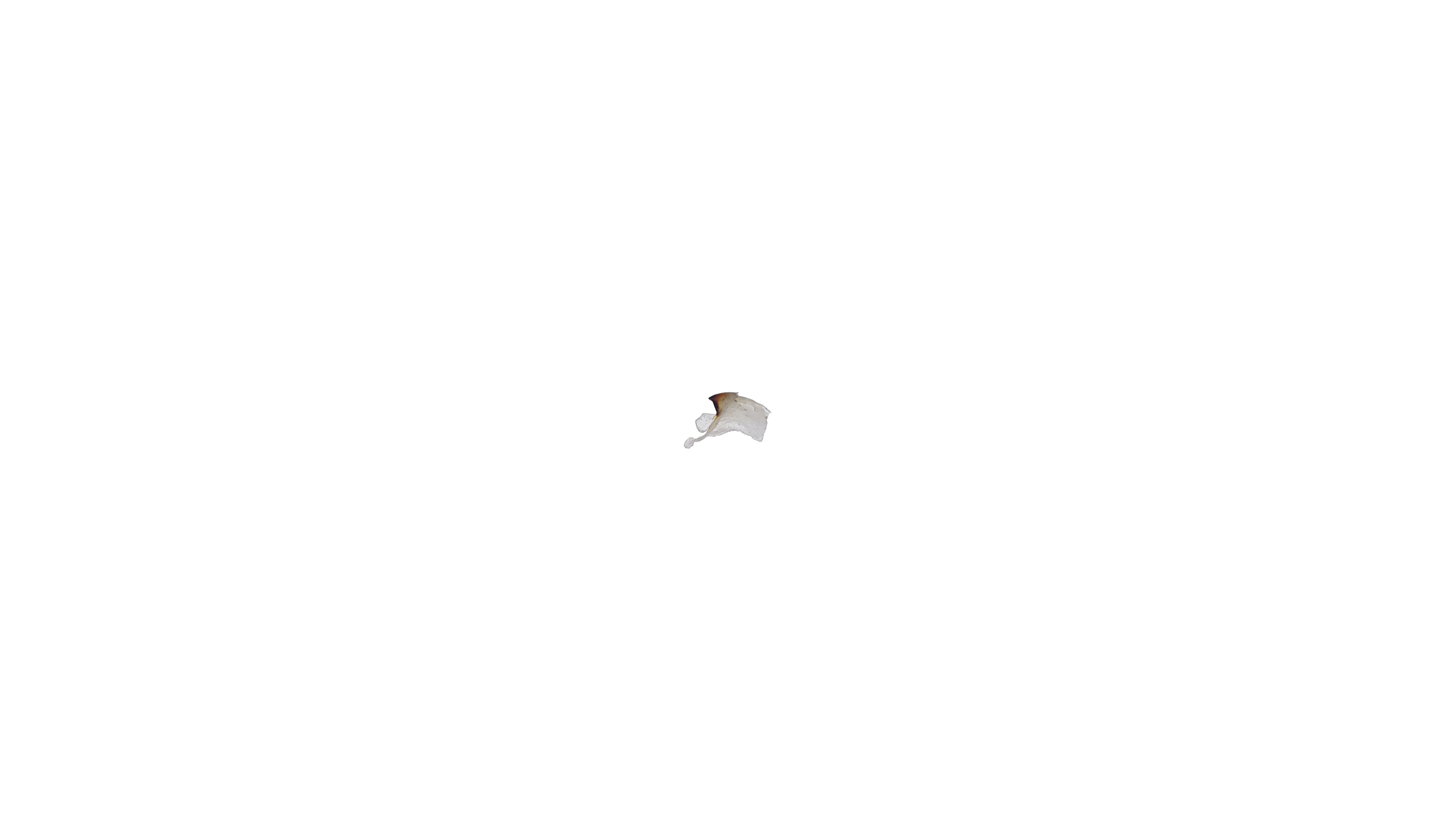

Supplement: Supplemental Information 1 — C2-Sepia aculeata, C3-Sepioteuthis lessoniana, C6-Sepia esculenta, O2-Amphioctopus aegina, S1-Loliolus uyii, S3-Uroteuthis chinensis, S4-Uroteuthis edulis [file peerj-09-11825-s001.zip › _Preprocessing_Lower_Beak/S1/L-l-S1-20.jpg]

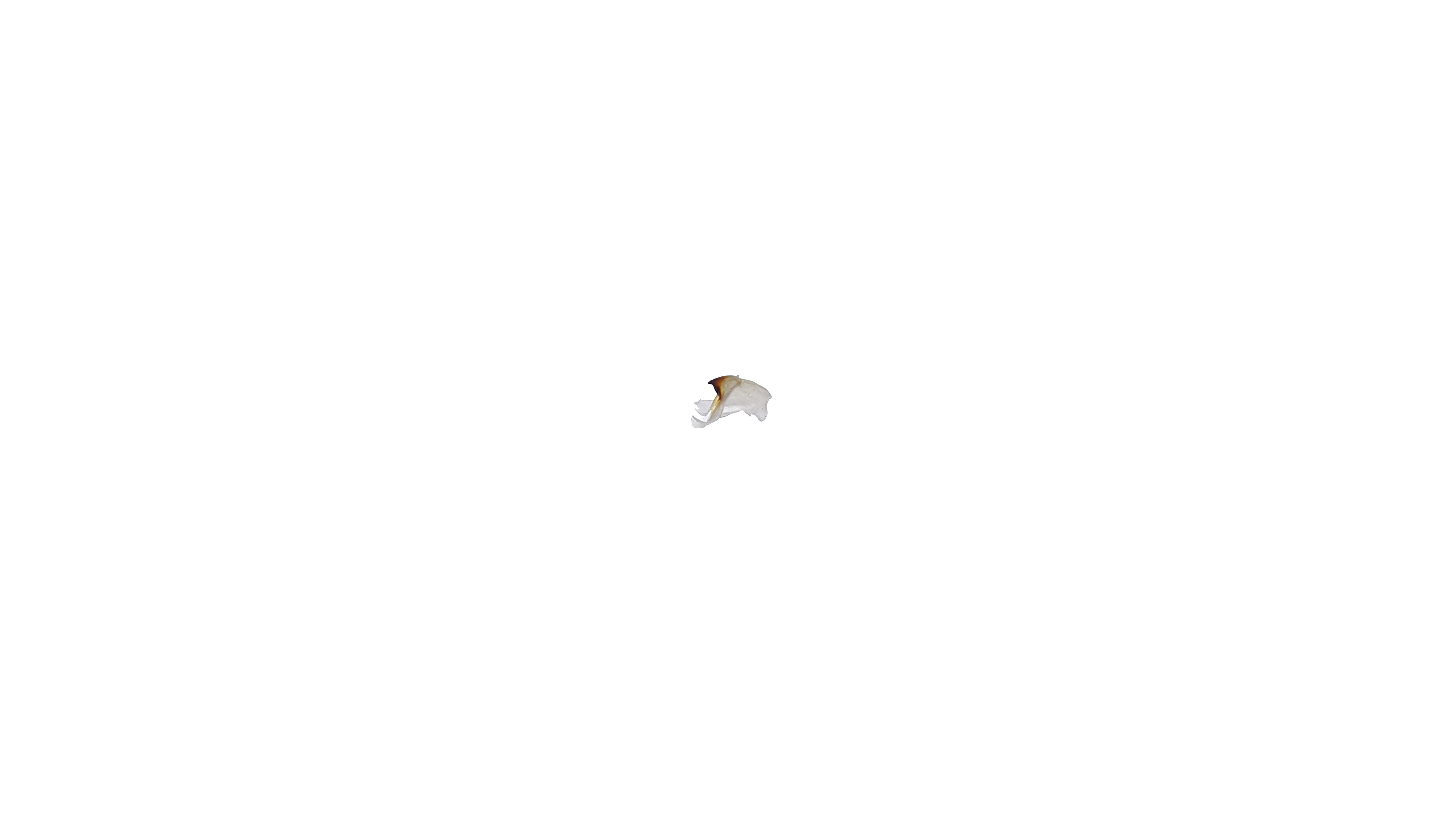

Supplement: Supplemental Information 1 — C2-Sepia aculeata, C3-Sepioteuthis lessoniana, C6-Sepia esculenta, O2-Amphioctopus aegina, S1-Loliolus uyii, S3-Uroteuthis chinensis, S4-Uroteuthis edulis [file peerj-09-11825-s001.zip › _Preprocessing_Lower_Beak/S1/L-l-S1-21.jpg]

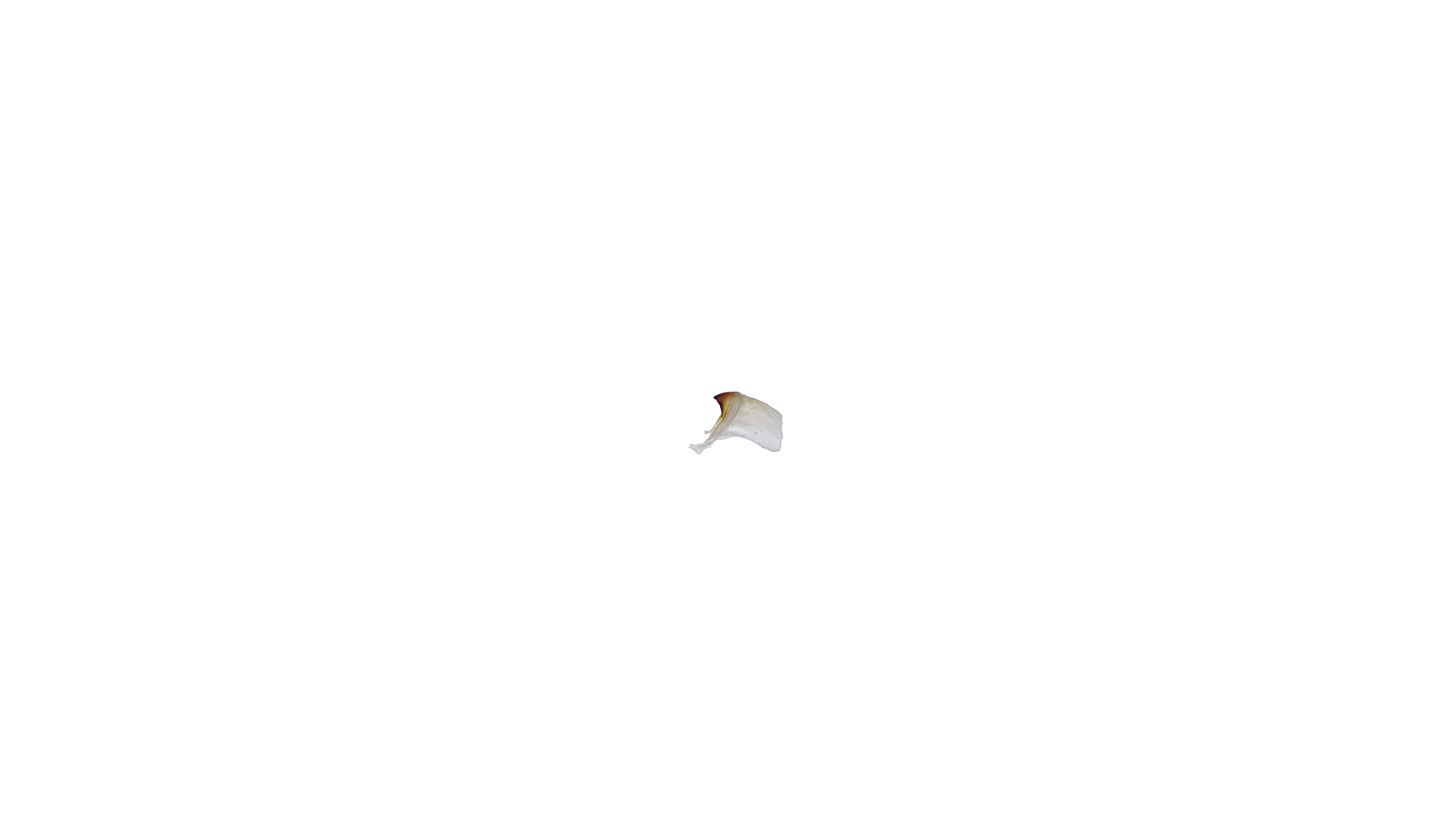

Supplement: Supplemental Information 1 — C2-Sepia aculeata, C3-Sepioteuthis lessoniana, C6-Sepia esculenta, O2-Amphioctopus aegina, S1-Loliolus uyii, S3-Uroteuthis chinensis, S4-Uroteuthis edulis [file peerj-09-11825-s001.zip › _Preprocessing_Lower_Beak/S1/L-l-S1-22.jpg]

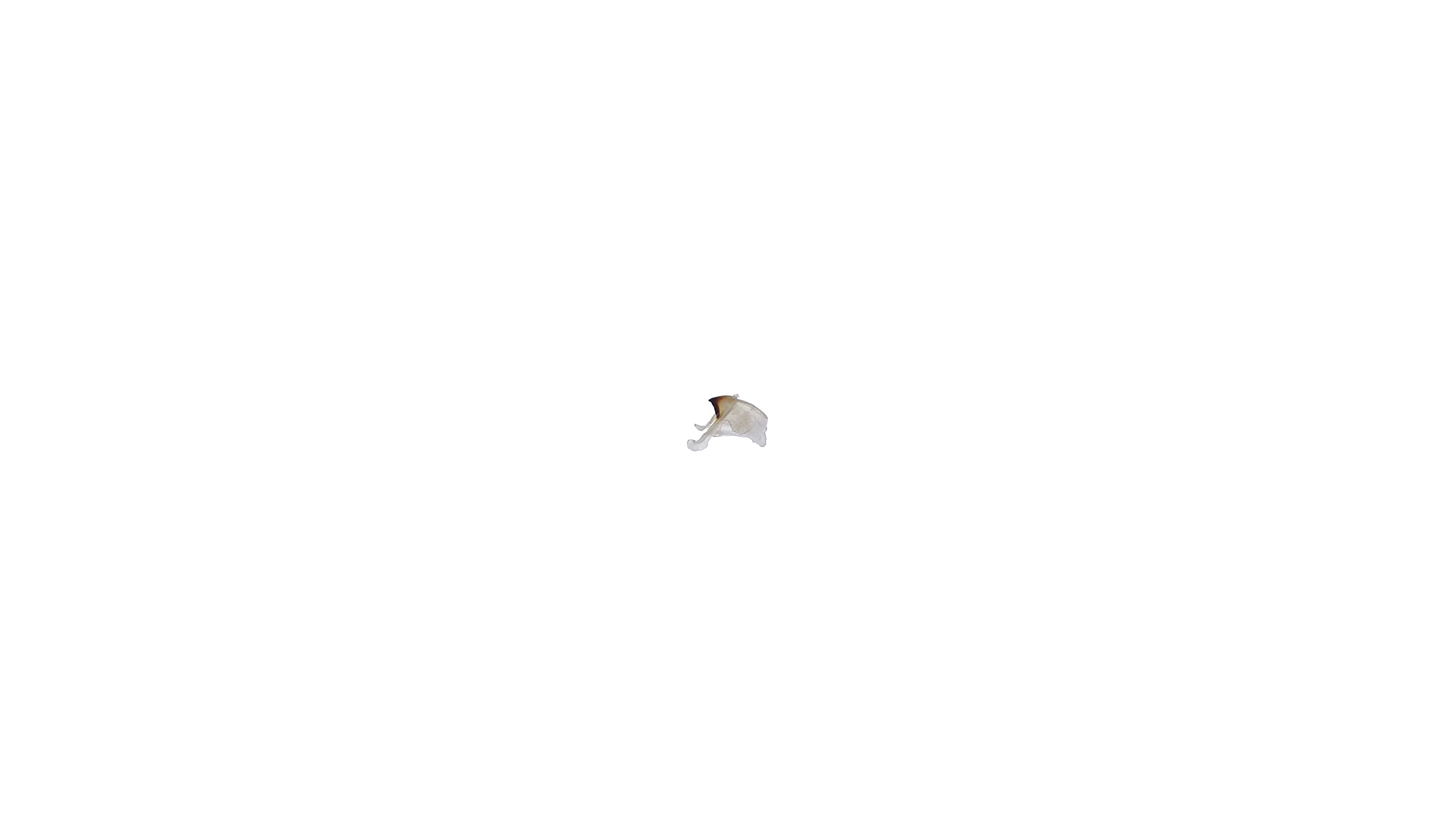

Supplement: Supplemental Information 1 — C2-Sepia aculeata, C3-Sepioteuthis lessoniana, C6-Sepia esculenta, O2-Amphioctopus aegina, S1-Loliolus uyii, S3-Uroteuthis chinensis, S4-Uroteuthis edulis [file peerj-09-11825-s001.zip › _Preprocessing_Lower_Beak/S1/L-l-S1-23.jpg]

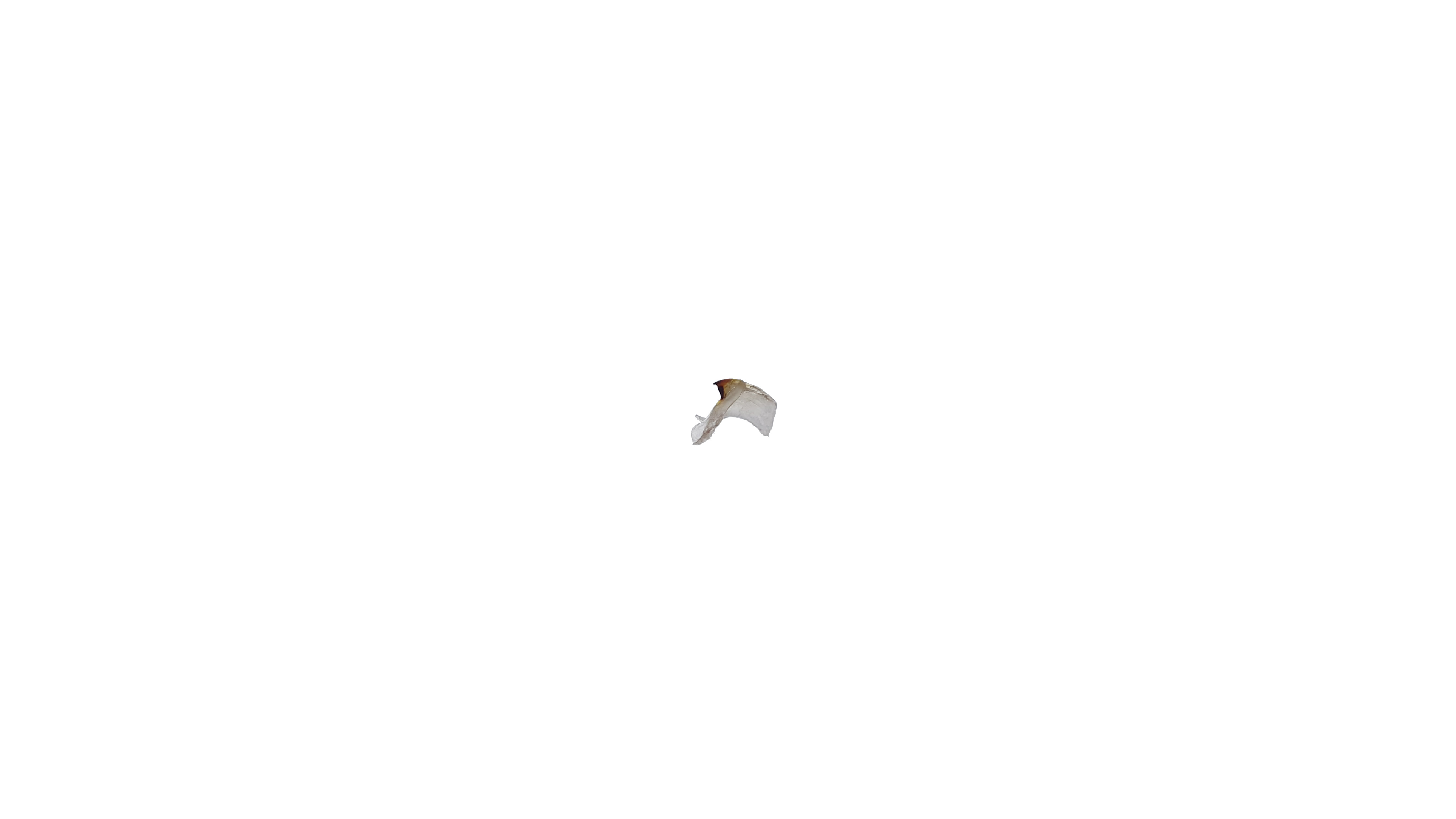

Supplement: Supplemental Information 1 — C2-Sepia aculeata, C3-Sepioteuthis lessoniana, C6-Sepia esculenta, O2-Amphioctopus aegina, S1-Loliolus uyii, S3-Uroteuthis chinensis, S4-Uroteuthis edulis [file peerj-09-11825-s001.zip › _Preprocessing_Lower_Beak/S1/L-l-S1-24.jpg]

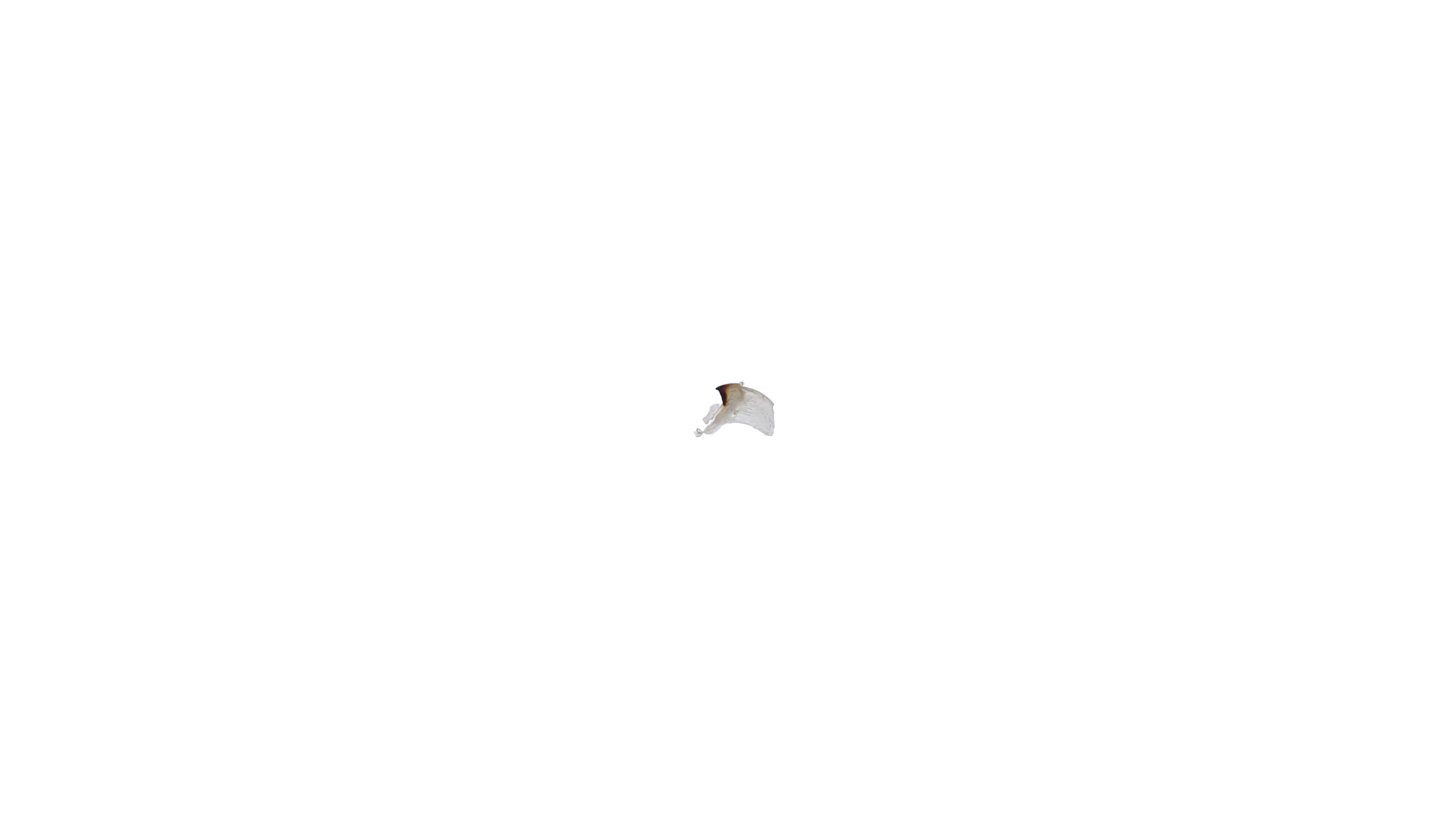

Supplement: Supplemental Information 1 — C2-Sepia aculeata, C3-Sepioteuthis lessoniana, C6-Sepia esculenta, O2-Amphioctopus aegina, S1-Loliolus uyii, S3-Uroteuthis chinensis, S4-Uroteuthis edulis [file peerj-09-11825-s001.zip › _Preprocessing_Lower_Beak/S1/L-l-S1-25.jpg]

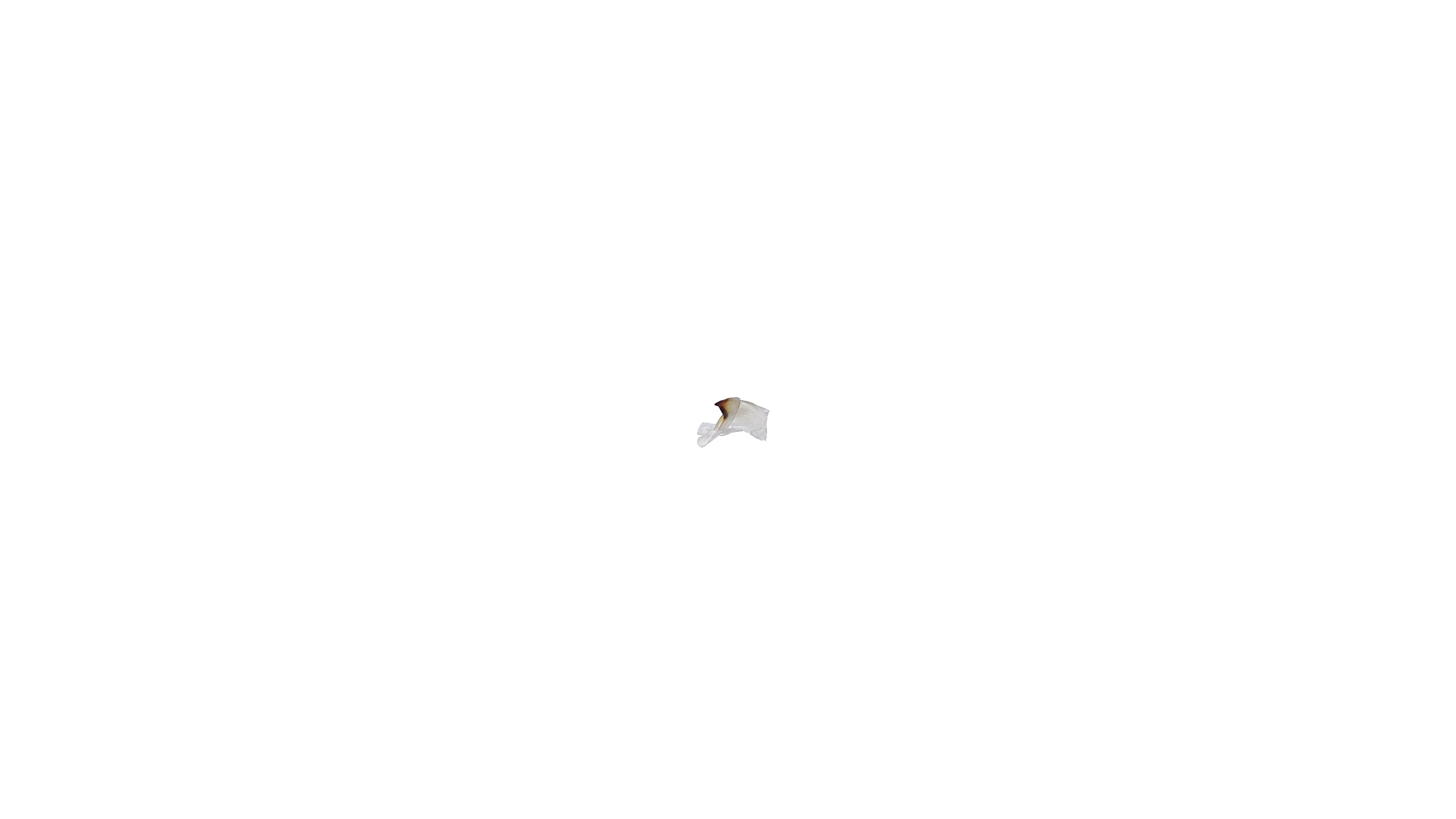

Supplement: Supplemental Information 1 — C2-Sepia aculeata, C3-Sepioteuthis lessoniana, C6-Sepia esculenta, O2-Amphioctopus aegina, S1-Loliolus uyii, S3-Uroteuthis chinensis, S4-Uroteuthis edulis [file peerj-09-11825-s001.zip › _Preprocessing_Lower_Beak/S1/L-l-S1-26.jpg]

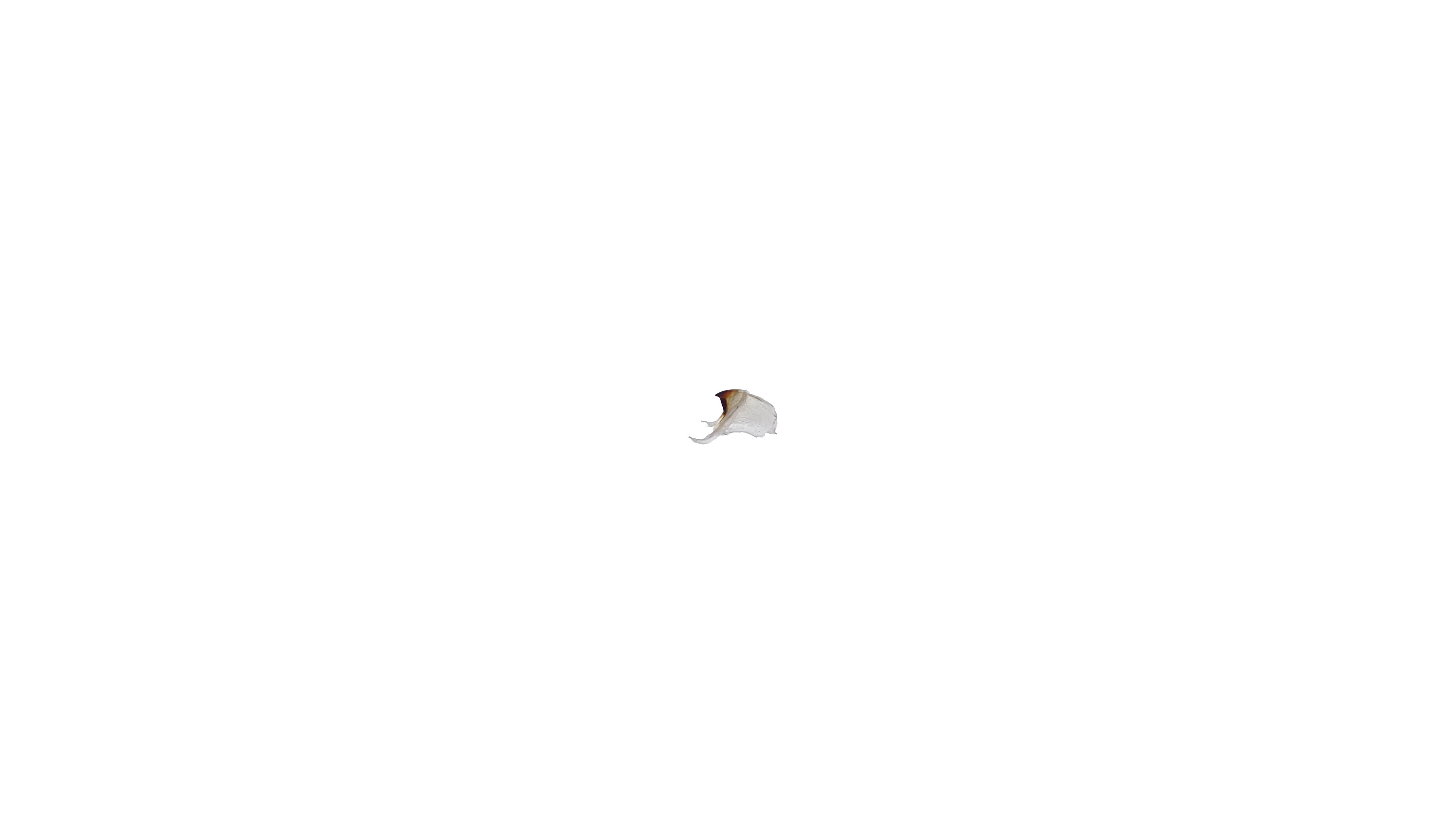

Supplement: Supplemental Information 1 — C2-Sepia aculeata, C3-Sepioteuthis lessoniana, C6-Sepia esculenta, O2-Amphioctopus aegina, S1-Loliolus uyii, S3-Uroteuthis chinensis, S4-Uroteuthis edulis [file peerj-09-11825-s001.zip › _Preprocessing_Lower_Beak/S1/L-l-S1-27.jpg]

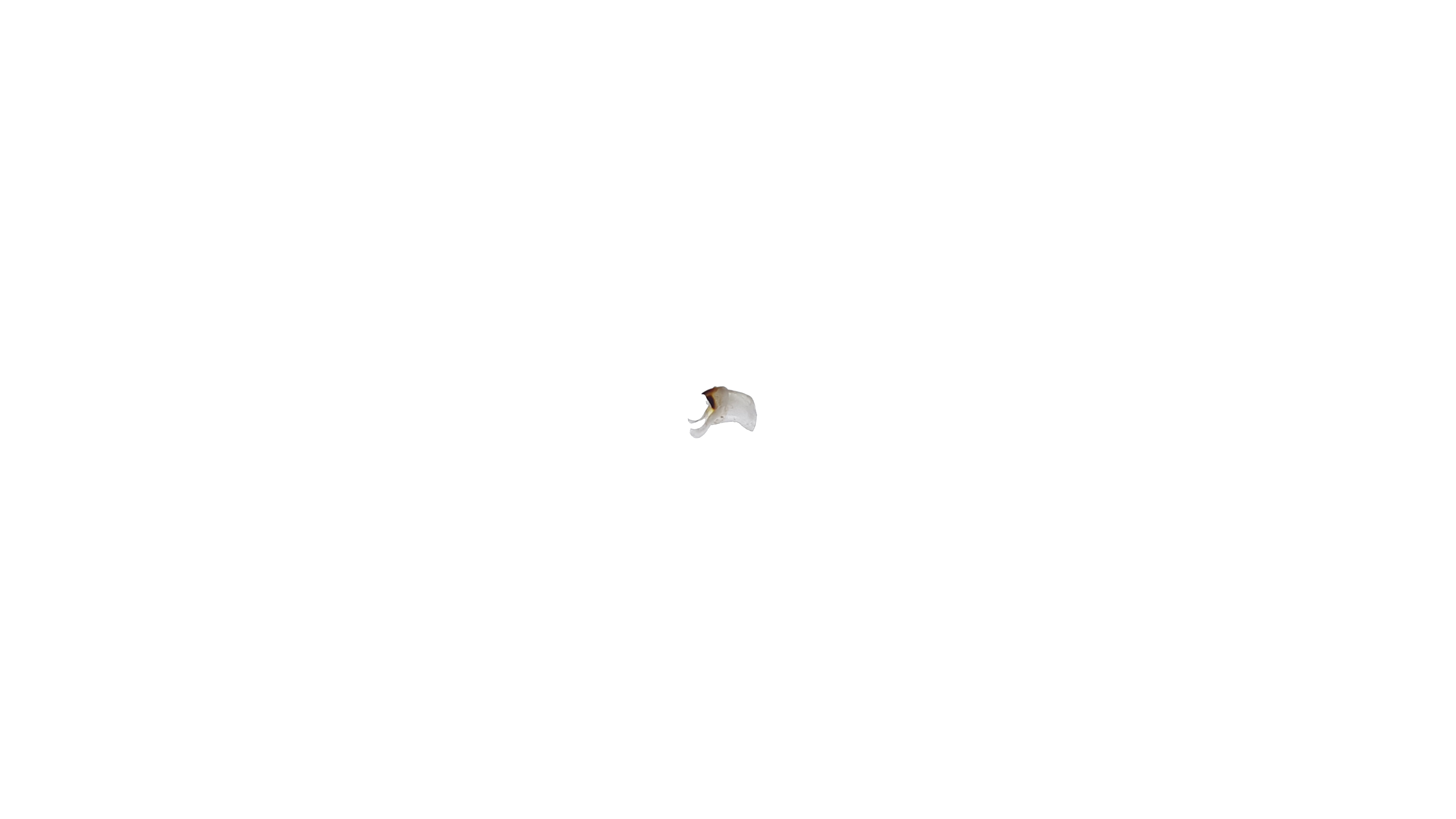

Supplement: Supplemental Information 1 — C2-Sepia aculeata, C3-Sepioteuthis lessoniana, C6-Sepia esculenta, O2-Amphioctopus aegina, S1-Loliolus uyii, S3-Uroteuthis chinensis, S4-Uroteuthis edulis [file peerj-09-11825-s001.zip › _Preprocessing_Lower_Beak/S1/L-l-S1-28.jpg]

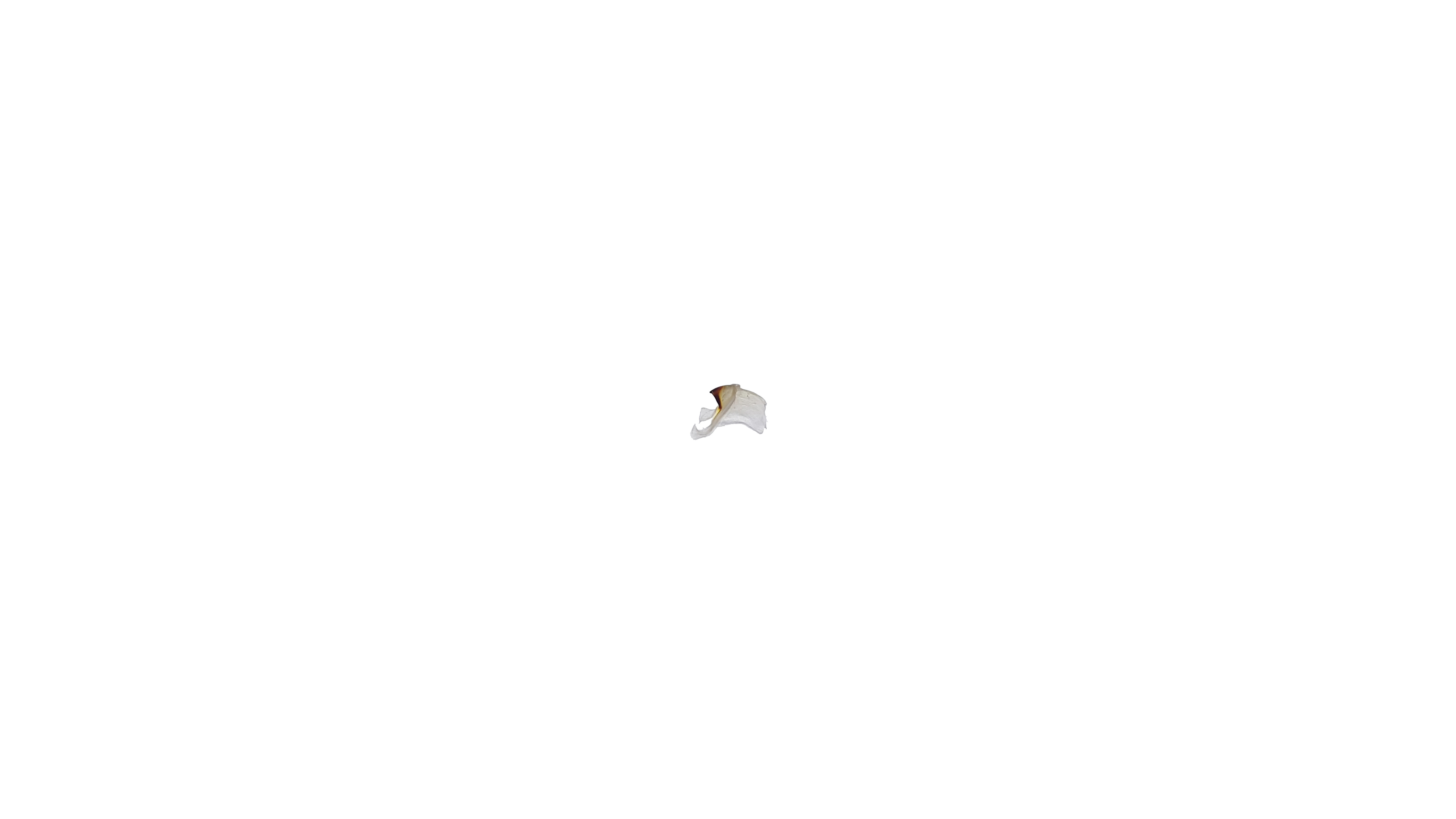

Supplement: Supplemental Information 1 — C2-Sepia aculeata, C3-Sepioteuthis lessoniana, C6-Sepia esculenta, O2-Amphioctopus aegina, S1-Loliolus uyii, S3-Uroteuthis chinensis, S4-Uroteuthis edulis [file peerj-09-11825-s001.zip › _Preprocessing_Lower_Beak/S1/L-l-S1-29.jpg]

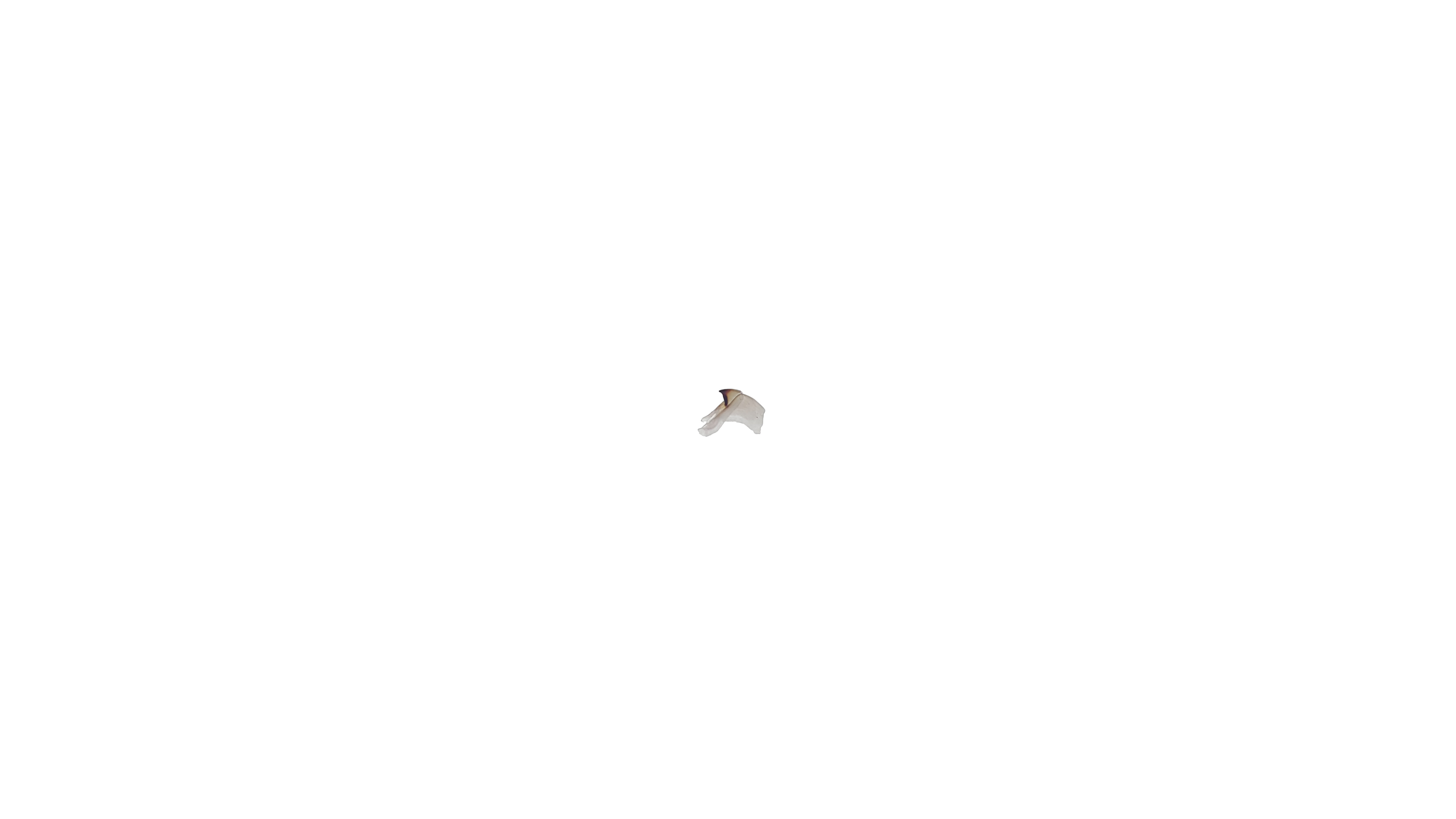

Supplement: Supplemental Information 1 — C2-Sepia aculeata, C3-Sepioteuthis lessoniana, C6-Sepia esculenta, O2-Amphioctopus aegina, S1-Loliolus uyii, S3-Uroteuthis chinensis, S4-Uroteuthis edulis [file peerj-09-11825-s001.zip › _Preprocessing_Lower_Beak/S1/L-l-S1-3.jpg]

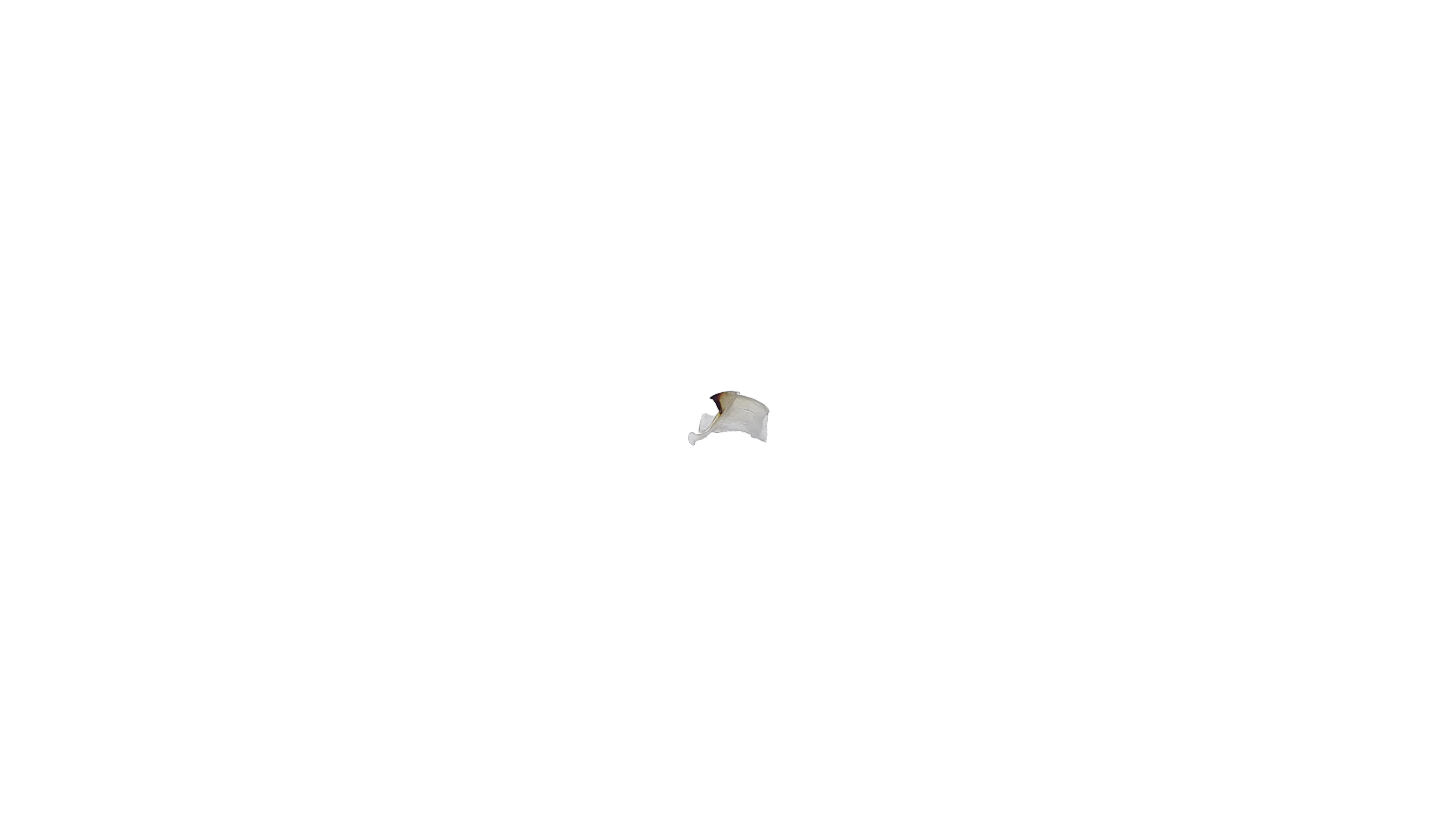

Supplement: Supplemental Information 1 — C2-Sepia aculeata, C3-Sepioteuthis lessoniana, C6-Sepia esculenta, O2-Amphioctopus aegina, S1-Loliolus uyii, S3-Uroteuthis chinensis, S4-Uroteuthis edulis [file peerj-09-11825-s001.zip › _Preprocessing_Lower_Beak/S1/L-l-S1-30.jpg]

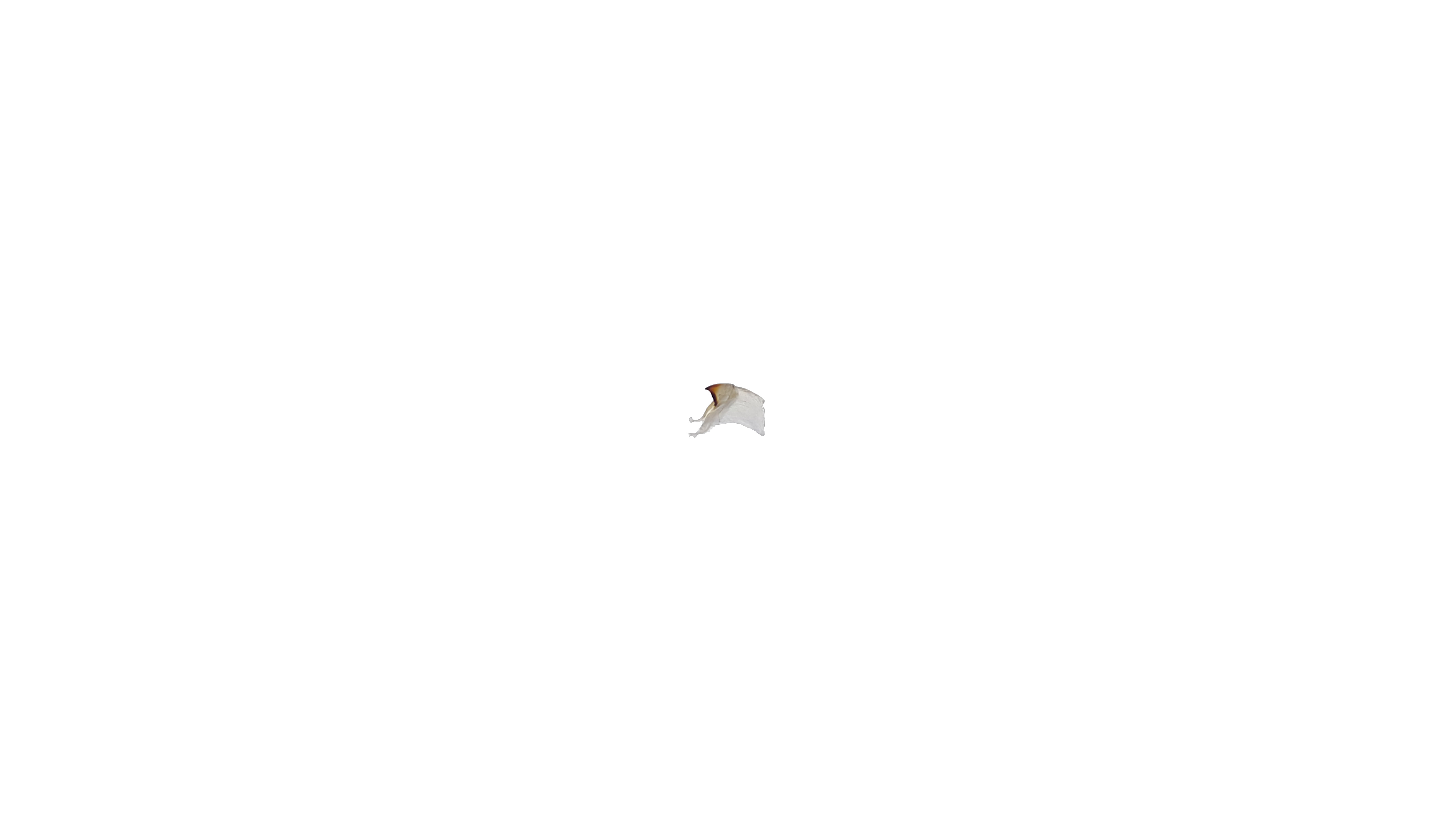

Supplement: Supplemental Information 1 — C2-Sepia aculeata, C3-Sepioteuthis lessoniana, C6-Sepia esculenta, O2-Amphioctopus aegina, S1-Loliolus uyii, S3-Uroteuthis chinensis, S4-Uroteuthis edulis [file peerj-09-11825-s001.zip › _Preprocessing_Lower_Beak/S1/L-l-S1-31.jpg]

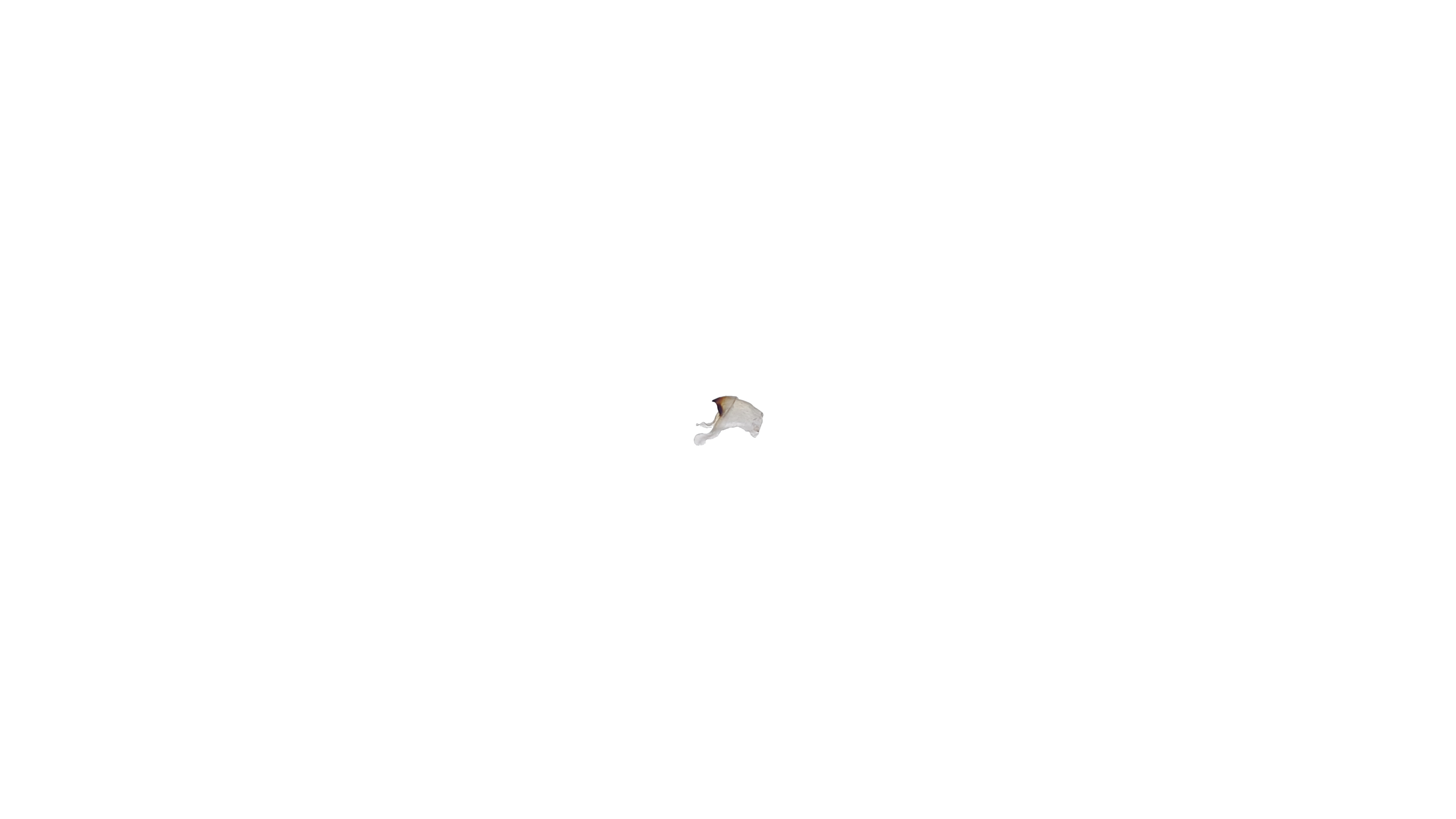

Supplement: Supplemental Information 1 — C2-Sepia aculeata, C3-Sepioteuthis lessoniana, C6-Sepia esculenta, O2-Amphioctopus aegina, S1-Loliolus uyii, S3-Uroteuthis chinensis, S4-Uroteuthis edulis [file peerj-09-11825-s001.zip › _Preprocessing_Lower_Beak/S1/L-l-S1-32.jpg]

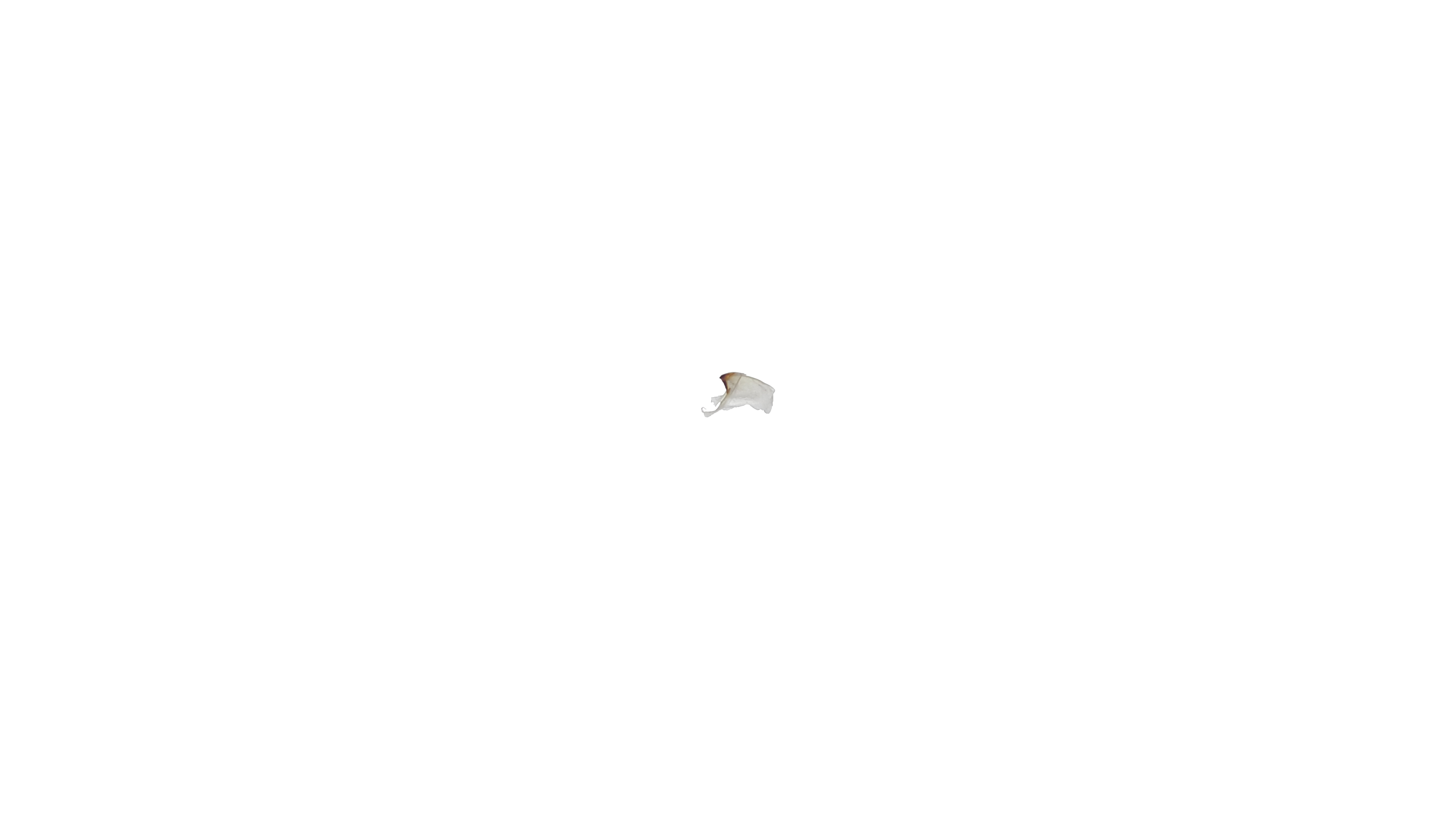

Supplement: Supplemental Information 1 — C2-Sepia aculeata, C3-Sepioteuthis lessoniana, C6-Sepia esculenta, O2-Amphioctopus aegina, S1-Loliolus uyii, S3-Uroteuthis chinensis, S4-Uroteuthis edulis [file peerj-09-11825-s001.zip › _Preprocessing_Lower_Beak/S1/L-l-S1-4.jpg]

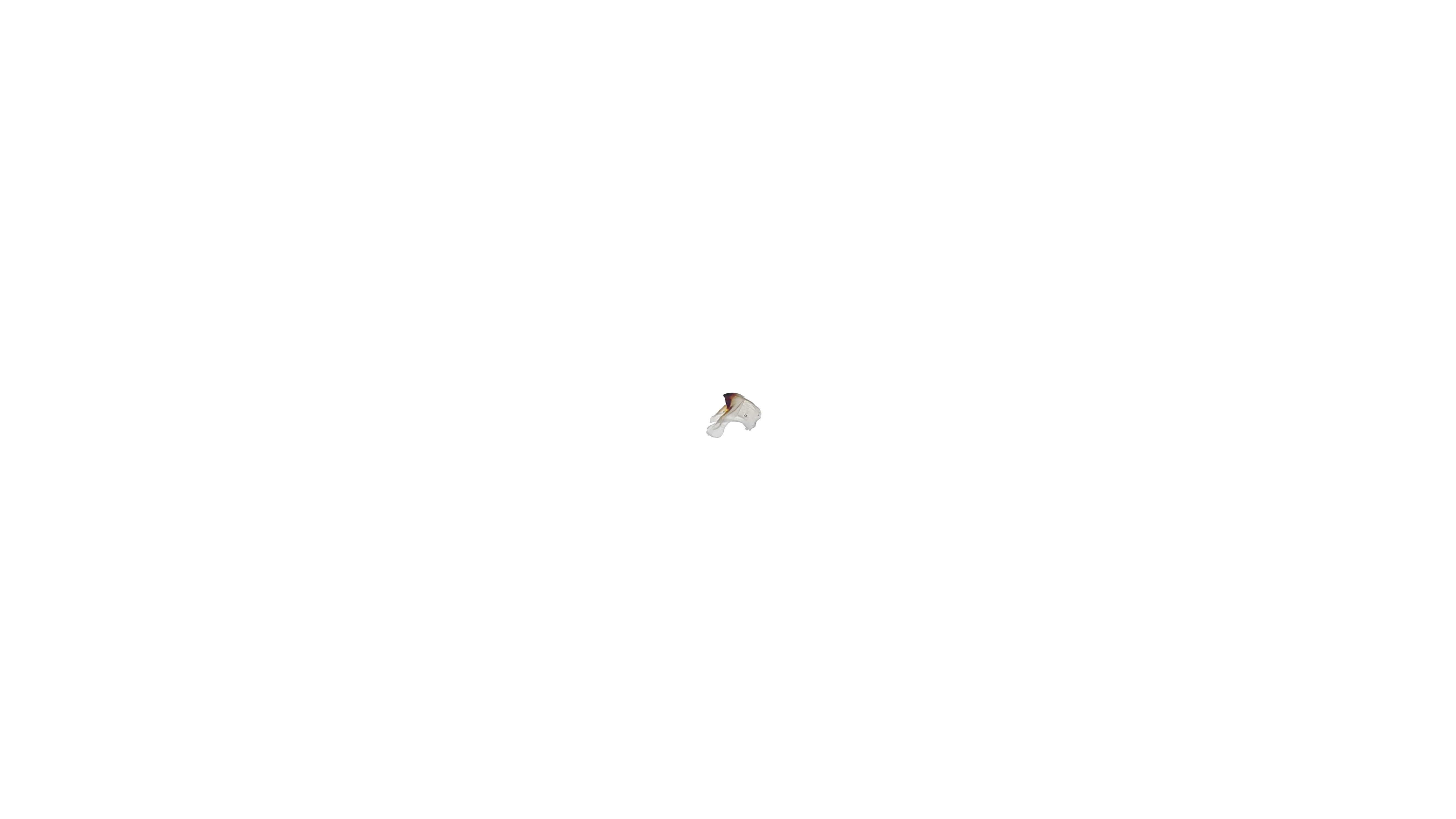

Supplement: Supplemental Information 1 — C2-Sepia aculeata, C3-Sepioteuthis lessoniana, C6-Sepia esculenta, O2-Amphioctopus aegina, S1-Loliolus uyii, S3-Uroteuthis chinensis, S4-Uroteuthis edulis [file peerj-09-11825-s001.zip › _Preprocessing_Lower_Beak/S1/L-l-S1-5.jpg]

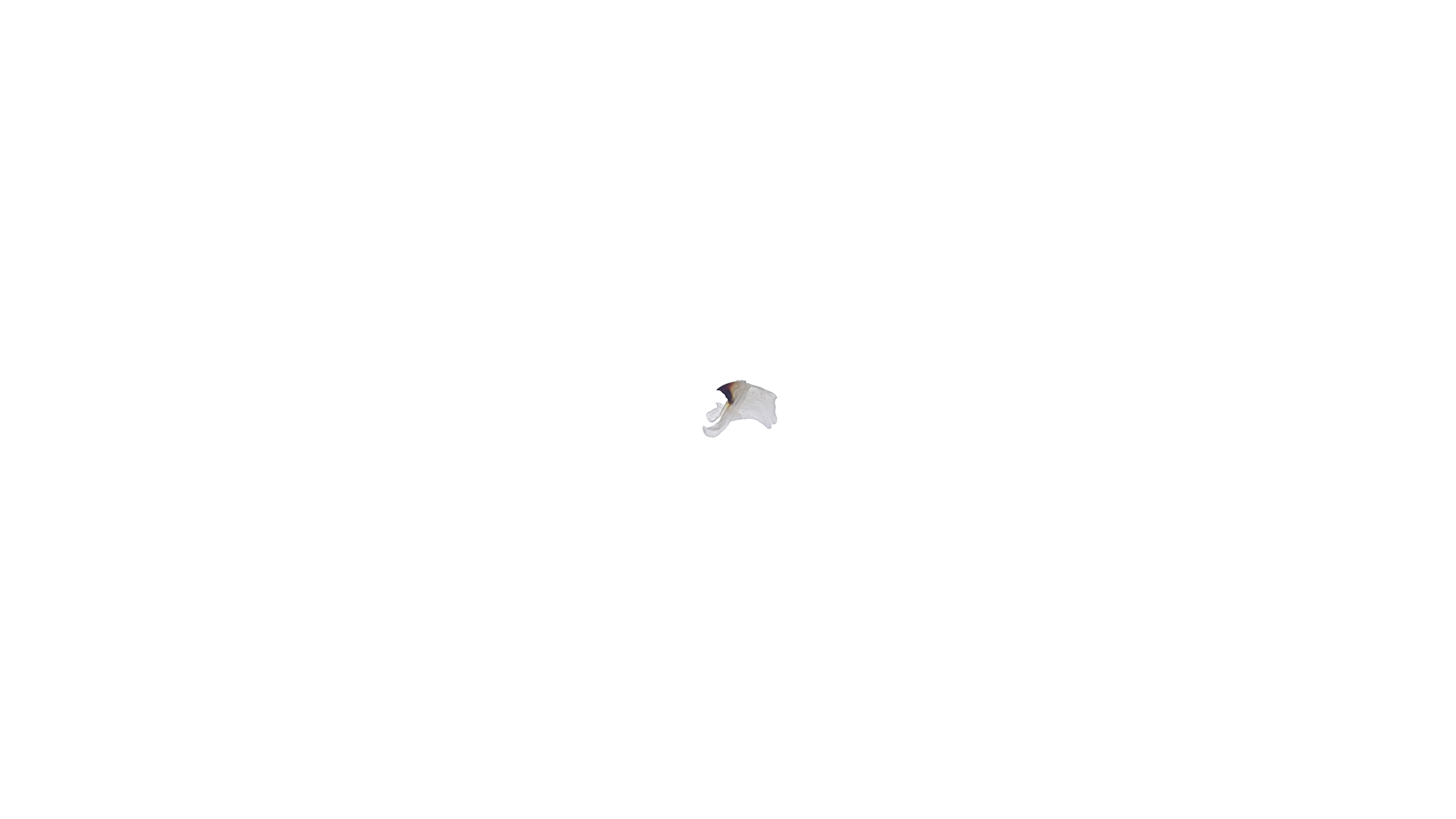

Supplement: Supplemental Information 1 — C2-Sepia aculeata, C3-Sepioteuthis lessoniana, C6-Sepia esculenta, O2-Amphioctopus aegina, S1-Loliolus uyii, S3-Uroteuthis chinensis, S4-Uroteuthis edulis [file peerj-09-11825-s001.zip › _Preprocessing_Lower_Beak/S1/L-l-S1-6.jpg]

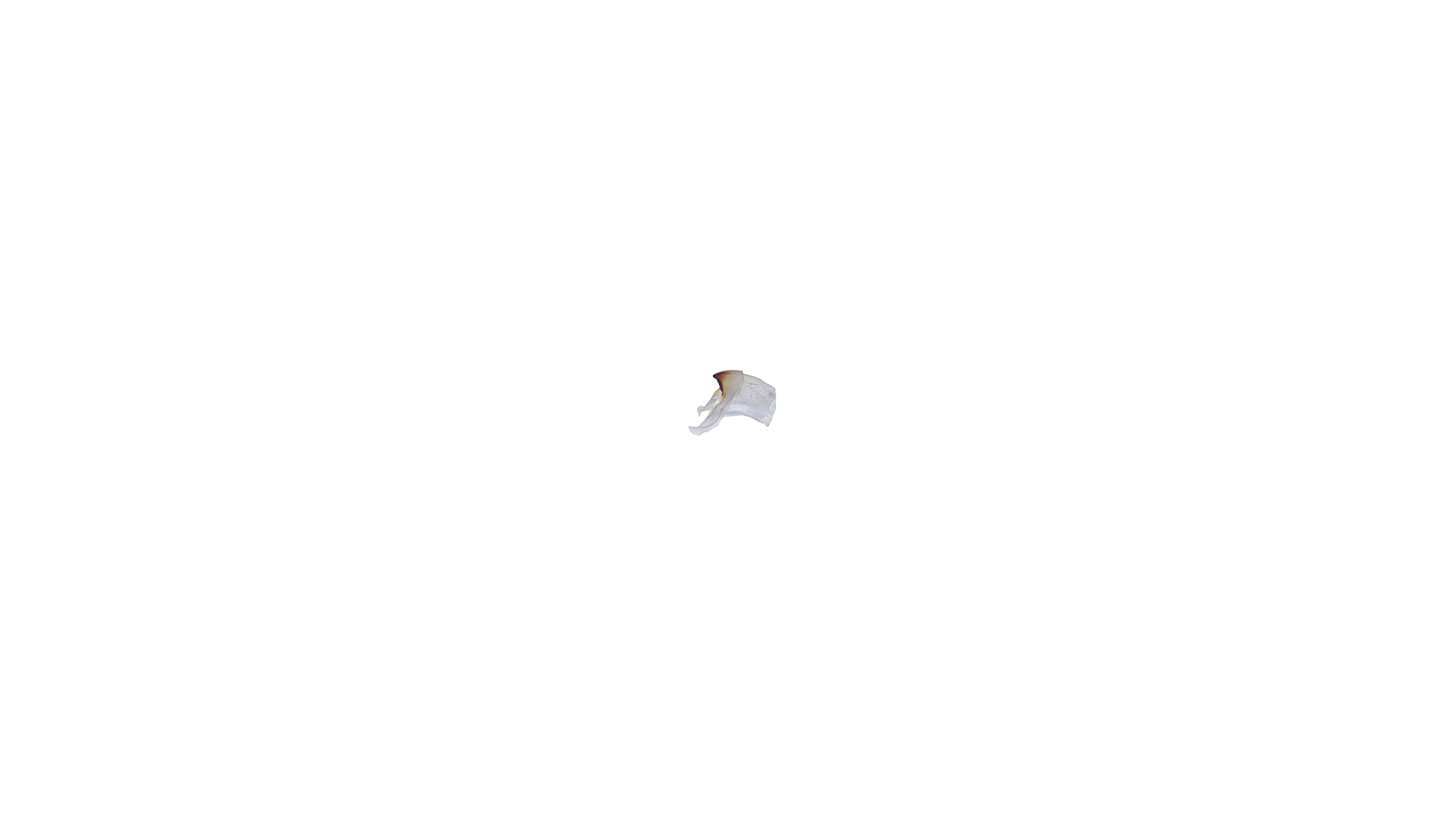

Supplement: Supplemental Information 1 — C2-Sepia aculeata, C3-Sepioteuthis lessoniana, C6-Sepia esculenta, O2-Amphioctopus aegina, S1-Loliolus uyii, S3-Uroteuthis chinensis, S4-Uroteuthis edulis [file peerj-09-11825-s001.zip › _Preprocessing_Lower_Beak/S1/L-l-S1-7.jpg]

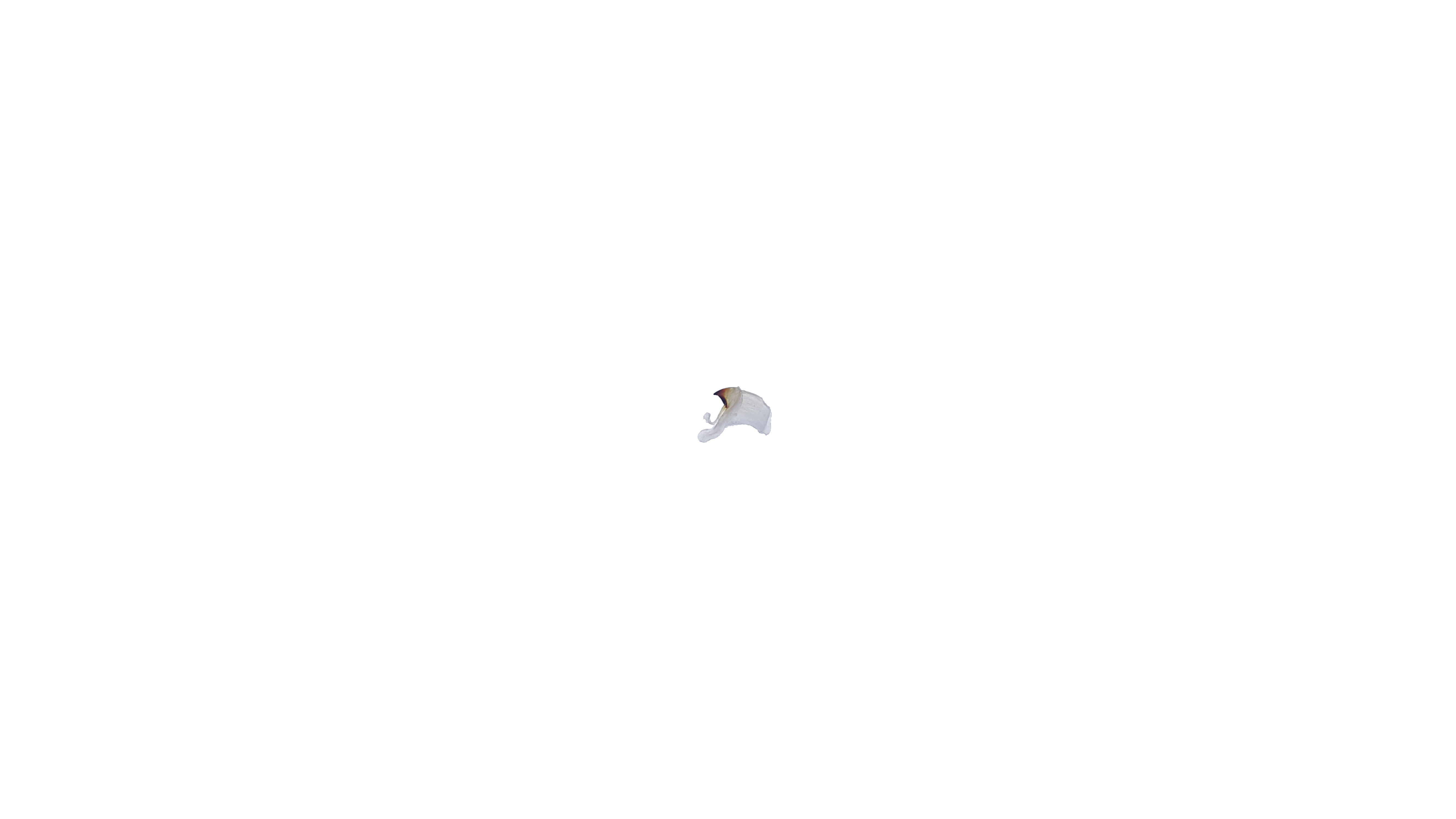

Supplement: Supplemental Information 1 — C2-Sepia aculeata, C3-Sepioteuthis lessoniana, C6-Sepia esculenta, O2-Amphioctopus aegina, S1-Loliolus uyii, S3-Uroteuthis chinensis, S4-Uroteuthis edulis [file peerj-09-11825-s001.zip › _Preprocessing_Lower_Beak/S1/L-l-S1-8.jpg]

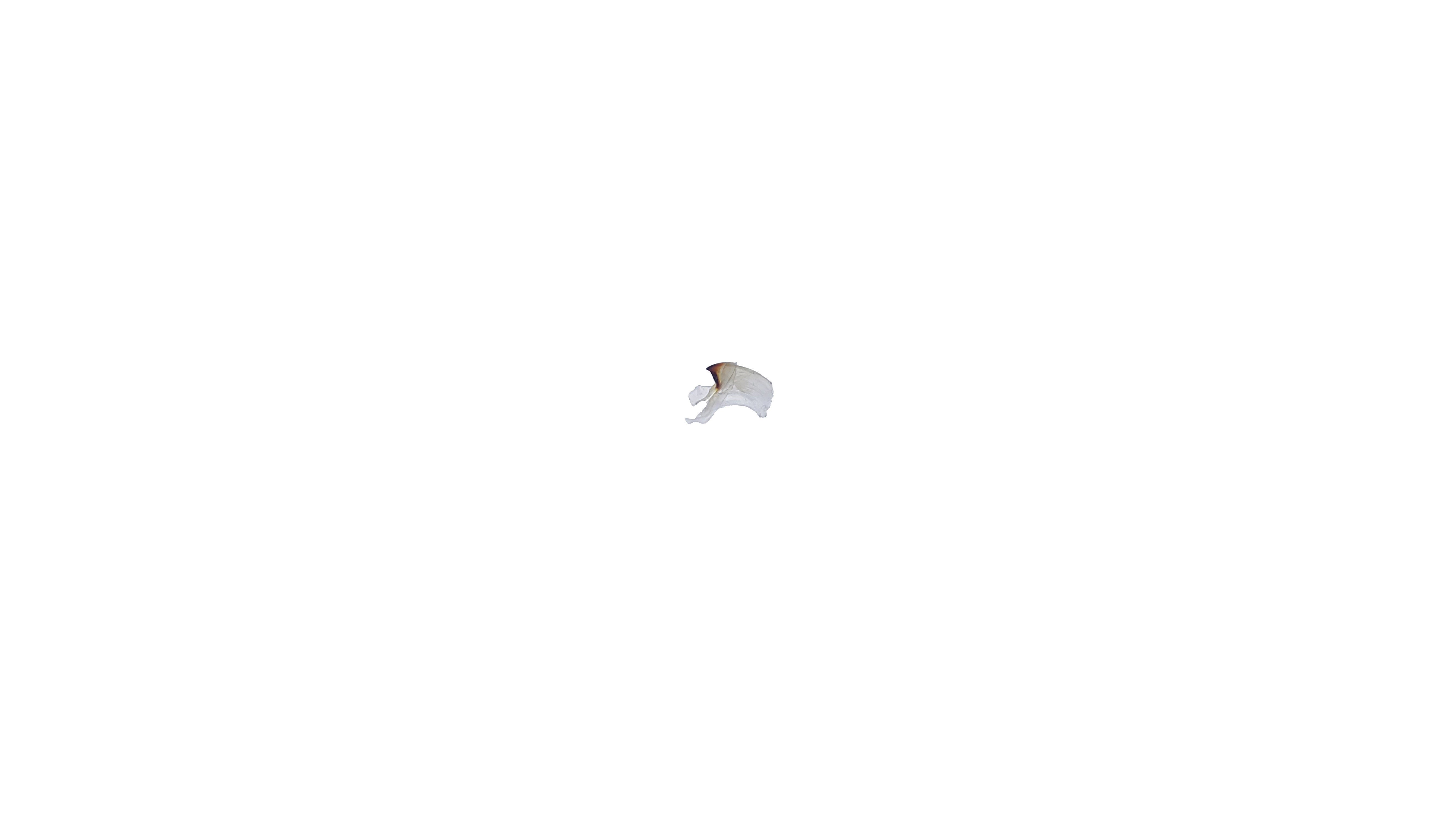

Supplement: Supplemental Information 1 — C2-Sepia aculeata, C3-Sepioteuthis lessoniana, C6-Sepia esculenta, O2-Amphioctopus aegina, S1-Loliolus uyii, S3-Uroteuthis chinensis, S4-Uroteuthis edulis [file peerj-09-11825-s001.zip › _Preprocessing_Lower_Beak/S1/L-l-S1-9.jpg]

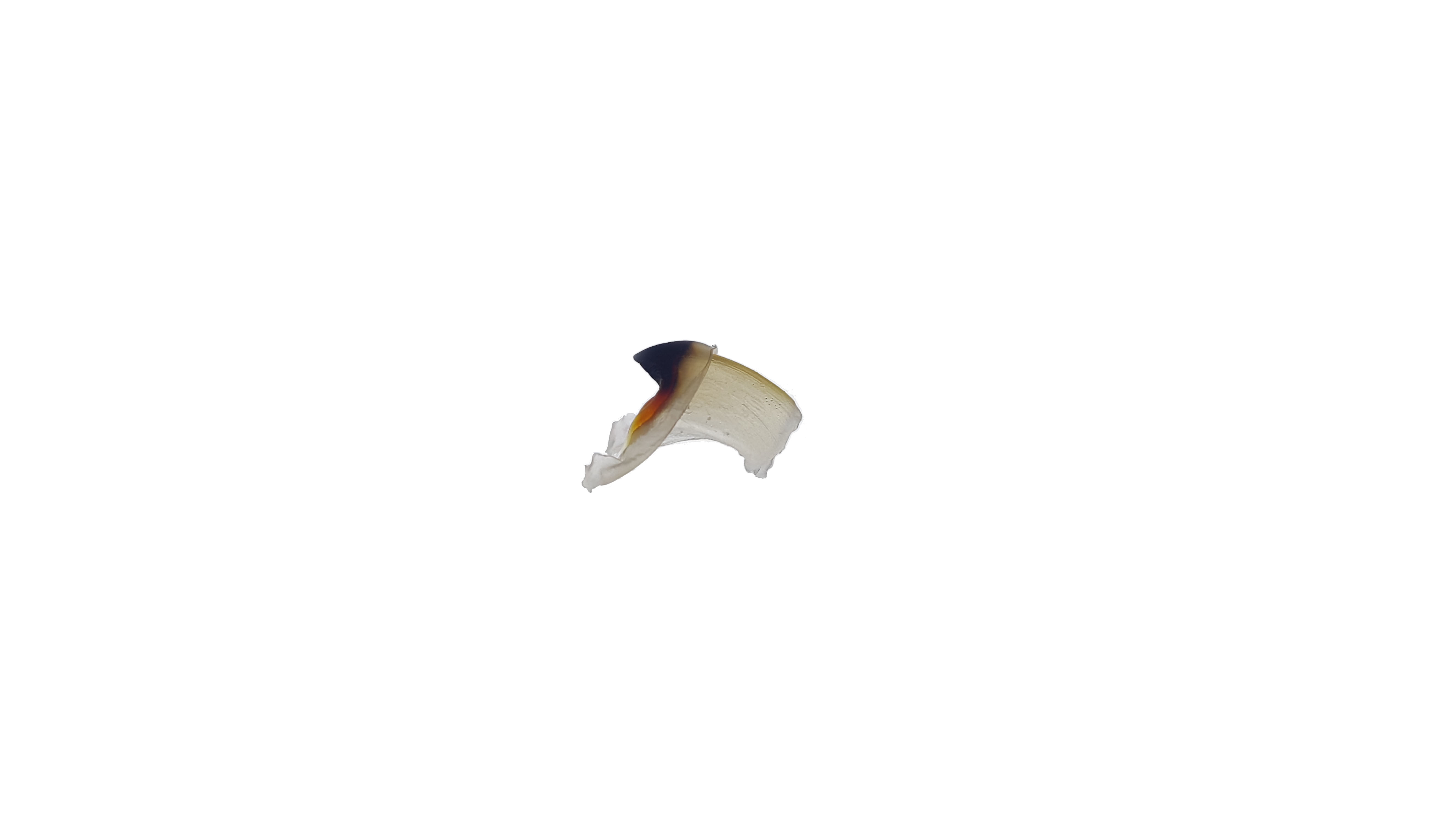

Supplement: Supplemental Information 1 — C2-Sepia aculeata, C3-Sepioteuthis lessoniana, C6-Sepia esculenta, O2-Amphioctopus aegina, S1-Loliolus uyii, S3-Uroteuthis chinensis, S4-Uroteuthis edulis [file peerj-09-11825-s001.zip › _Preprocessing_Lower_Beak/S3/L-l-S3-1.jpg]

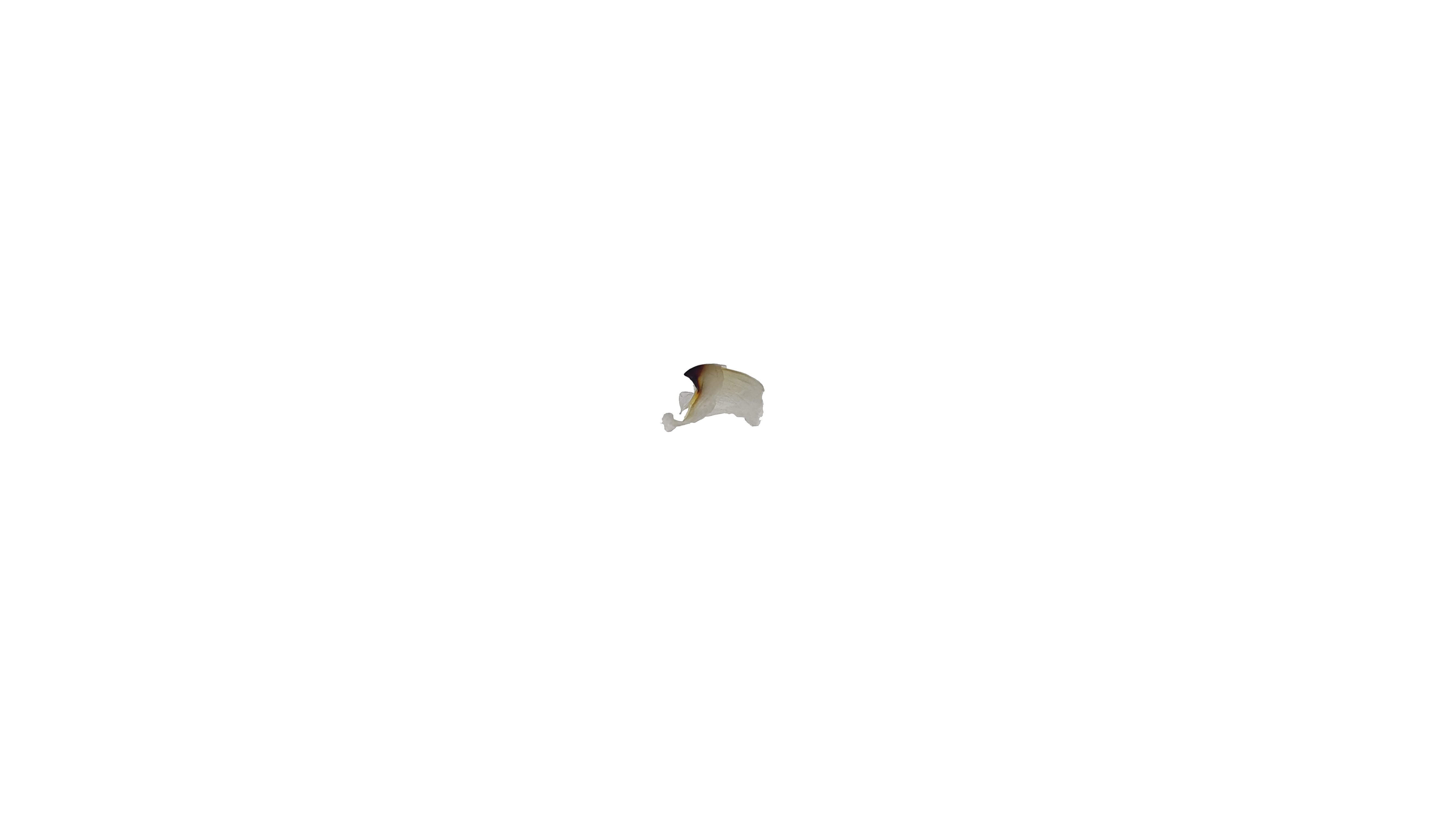

Supplement: Supplemental Information 1 — C2-Sepia aculeata, C3-Sepioteuthis lessoniana, C6-Sepia esculenta, O2-Amphioctopus aegina, S1-Loliolus uyii, S3-Uroteuthis chinensis, S4-Uroteuthis edulis [file peerj-09-11825-s001.zip › _Preprocessing_Lower_Beak/S3/L-l-S3-11.jpg]

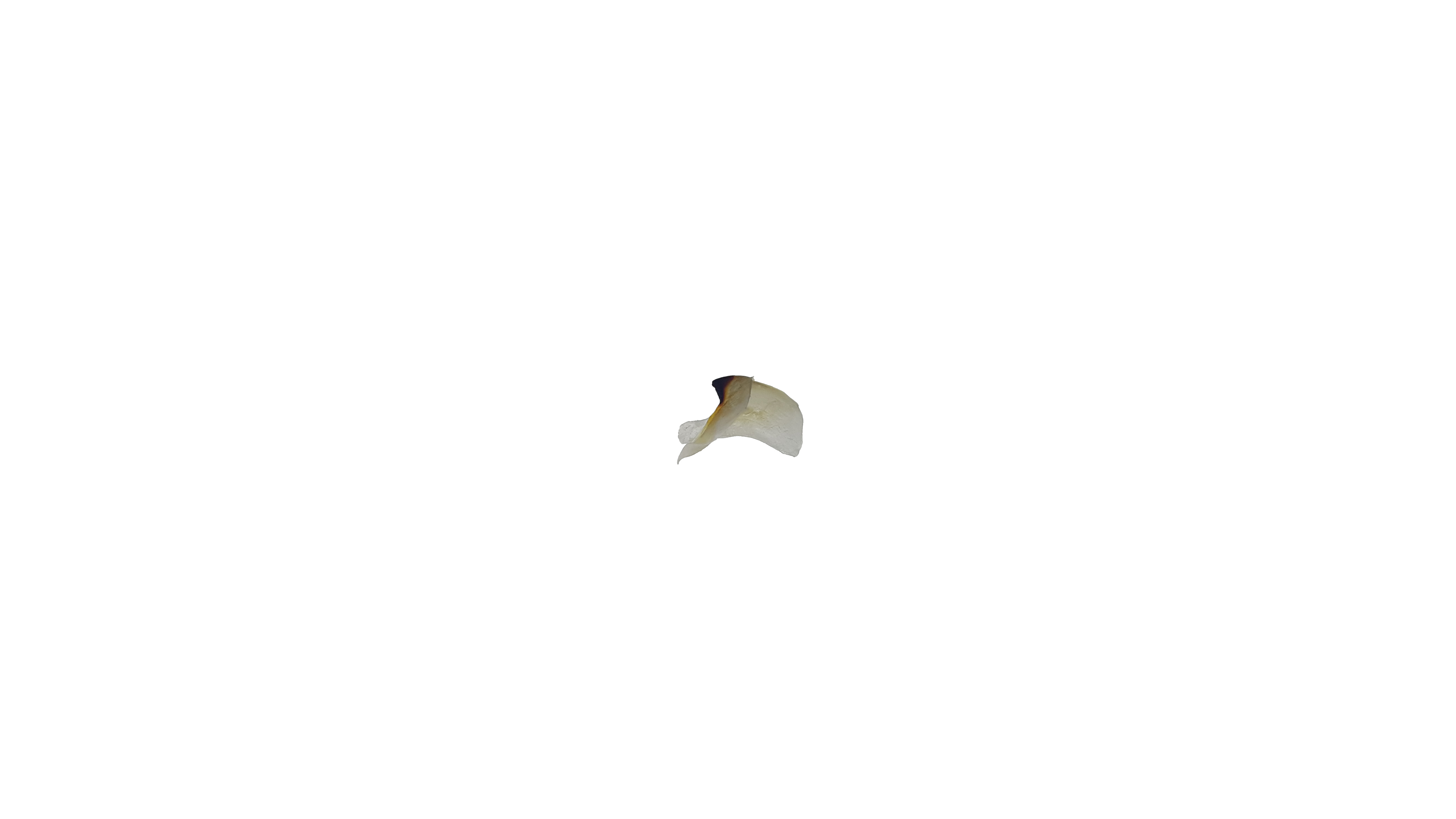

Supplement: Supplemental Information 1 — C2-Sepia aculeata, C3-Sepioteuthis lessoniana, C6-Sepia esculenta, O2-Amphioctopus aegina, S1-Loliolus uyii, S3-Uroteuthis chinensis, S4-Uroteuthis edulis [file peerj-09-11825-s001.zip › _Preprocessing_Lower_Beak/S3/L-l-S3-12.jpg]

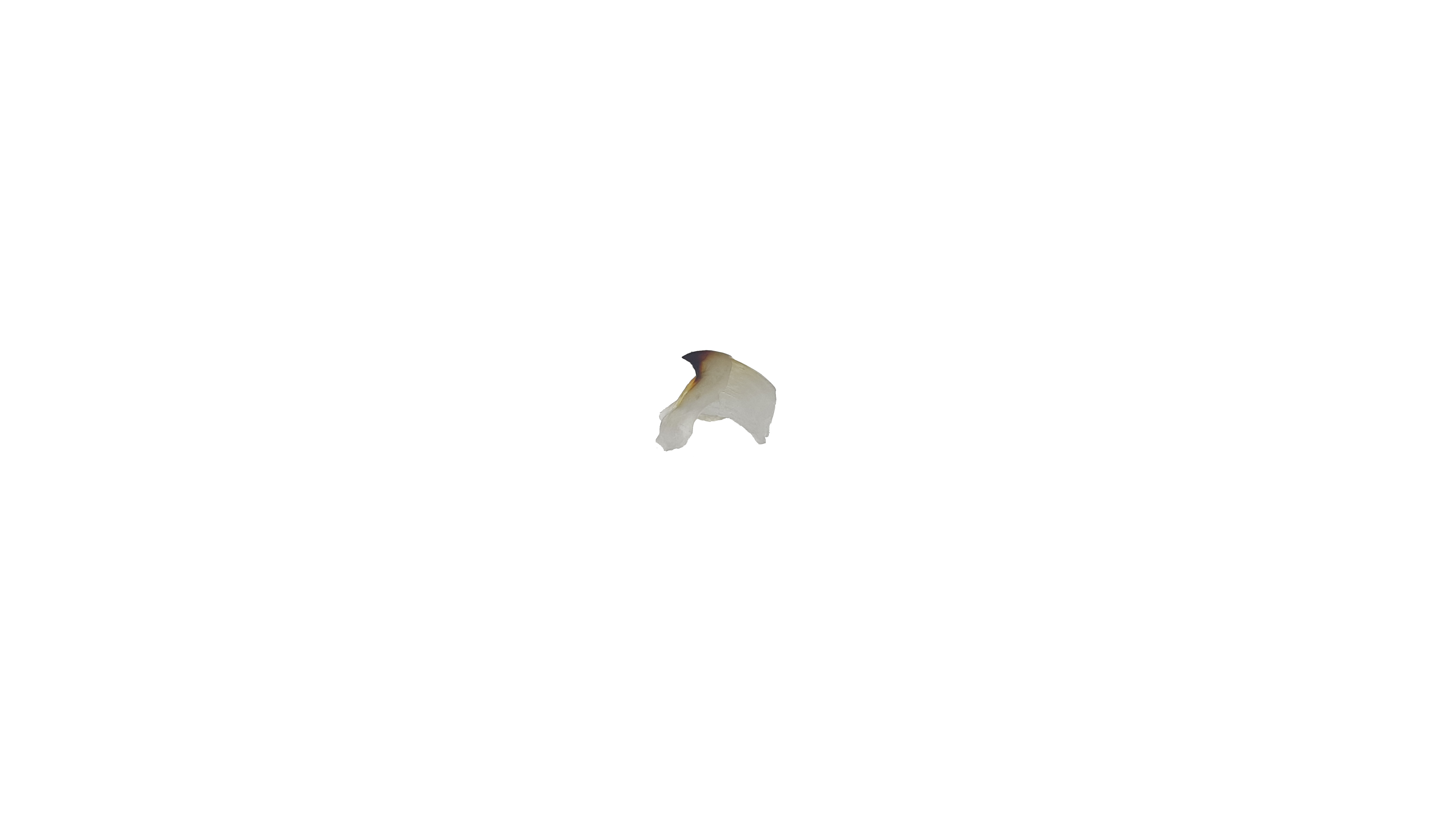

Supplement: Supplemental Information 1 — C2-Sepia aculeata, C3-Sepioteuthis lessoniana, C6-Sepia esculenta, O2-Amphioctopus aegina, S1-Loliolus uyii, S3-Uroteuthis chinensis, S4-Uroteuthis edulis [file peerj-09-11825-s001.zip › _Preprocessing_Lower_Beak/S3/L-l-S3-13.jpg]

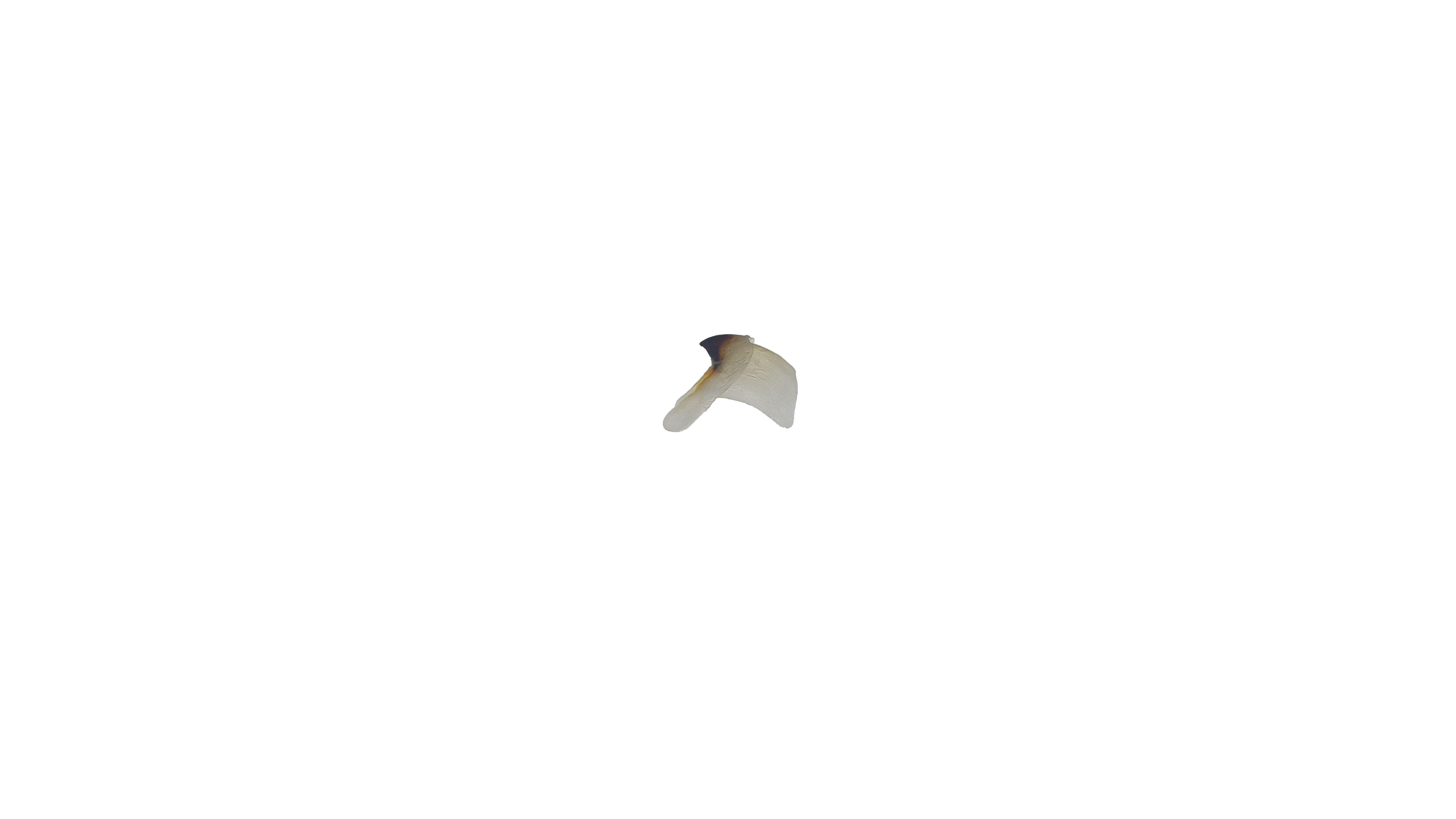

Supplement: Supplemental Information 1 — C2-Sepia aculeata, C3-Sepioteuthis lessoniana, C6-Sepia esculenta, O2-Amphioctopus aegina, S1-Loliolus uyii, S3-Uroteuthis chinensis, S4-Uroteuthis edulis [file peerj-09-11825-s001.zip › _Preprocessing_Lower_Beak/S3/L-l-S3-14.jpg]

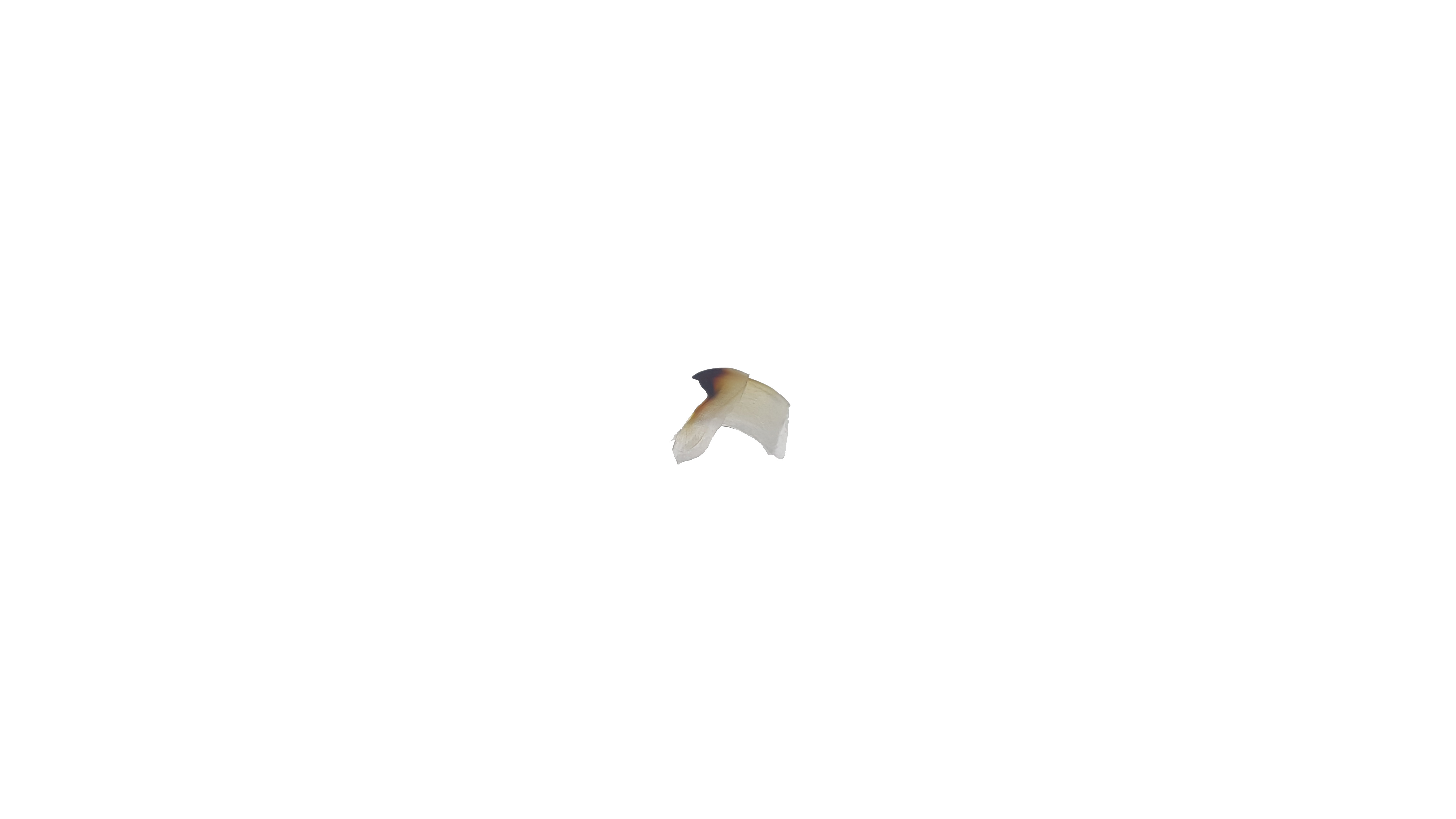

Supplement: Supplemental Information 1 — C2-Sepia aculeata, C3-Sepioteuthis lessoniana, C6-Sepia esculenta, O2-Amphioctopus aegina, S1-Loliolus uyii, S3-Uroteuthis chinensis, S4-Uroteuthis edulis [file peerj-09-11825-s001.zip › _Preprocessing_Lower_Beak/S3/L-l-S3-15.jpg]

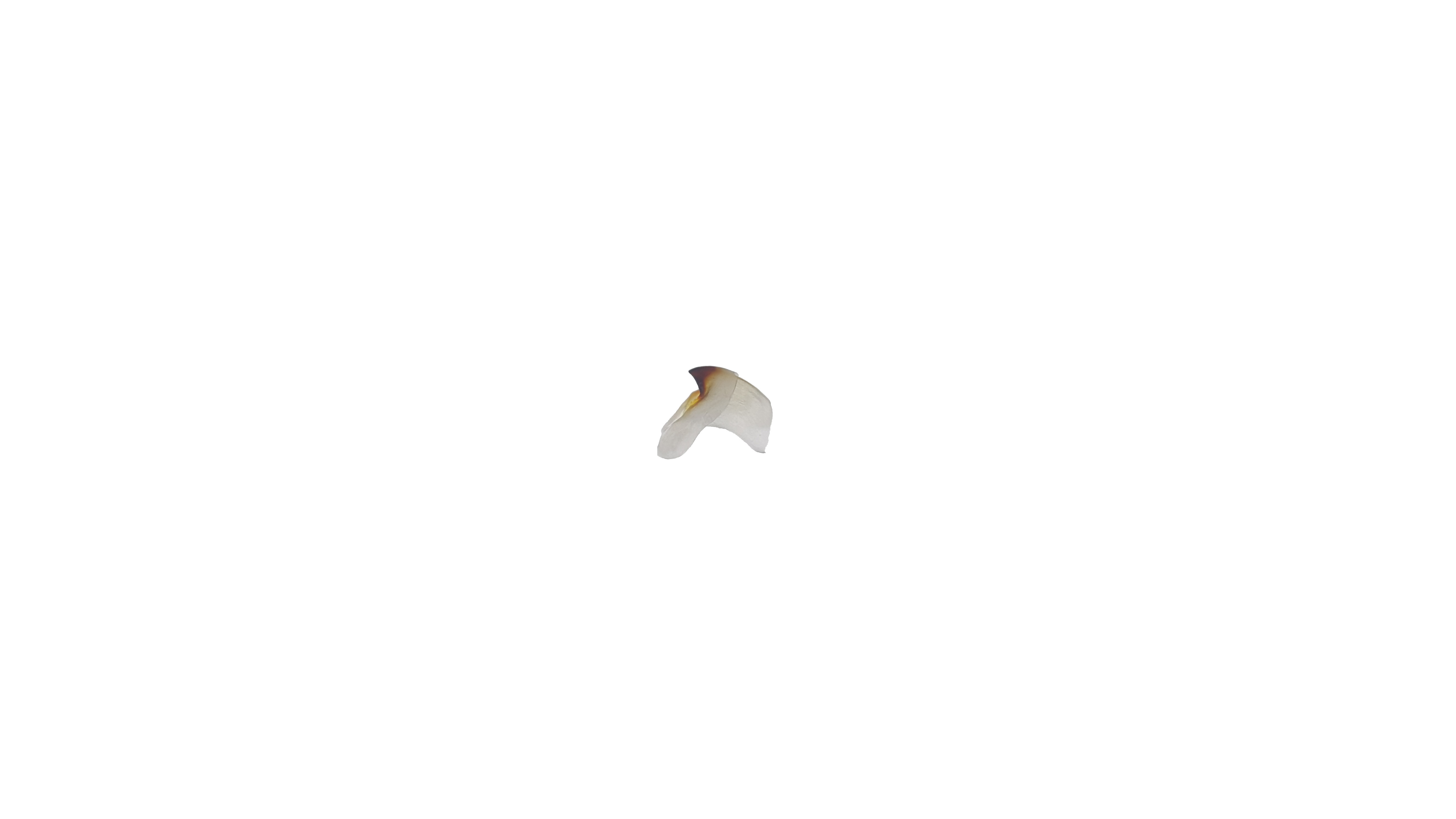

Supplement: Supplemental Information 1 — C2-Sepia aculeata, C3-Sepioteuthis lessoniana, C6-Sepia esculenta, O2-Amphioctopus aegina, S1-Loliolus uyii, S3-Uroteuthis chinensis, S4-Uroteuthis edulis [file peerj-09-11825-s001.zip › _Preprocessing_Lower_Beak/S3/L-l-S3-16.jpg]

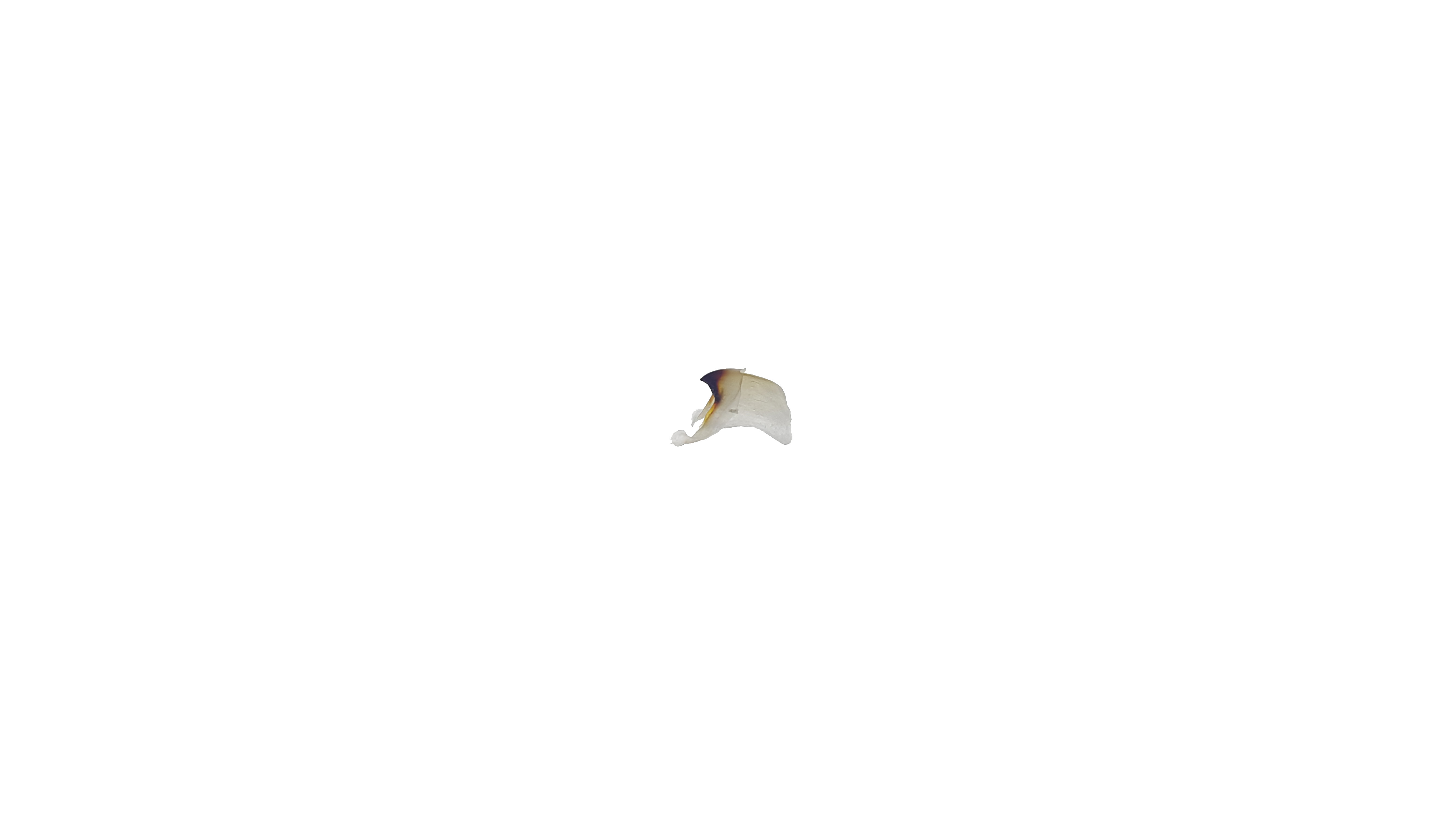

Supplement: Supplemental Information 1 — C2-Sepia aculeata, C3-Sepioteuthis lessoniana, C6-Sepia esculenta, O2-Amphioctopus aegina, S1-Loliolus uyii, S3-Uroteuthis chinensis, S4-Uroteuthis edulis [file peerj-09-11825-s001.zip › _Preprocessing_Lower_Beak/S3/L-l-S3-17.jpg]

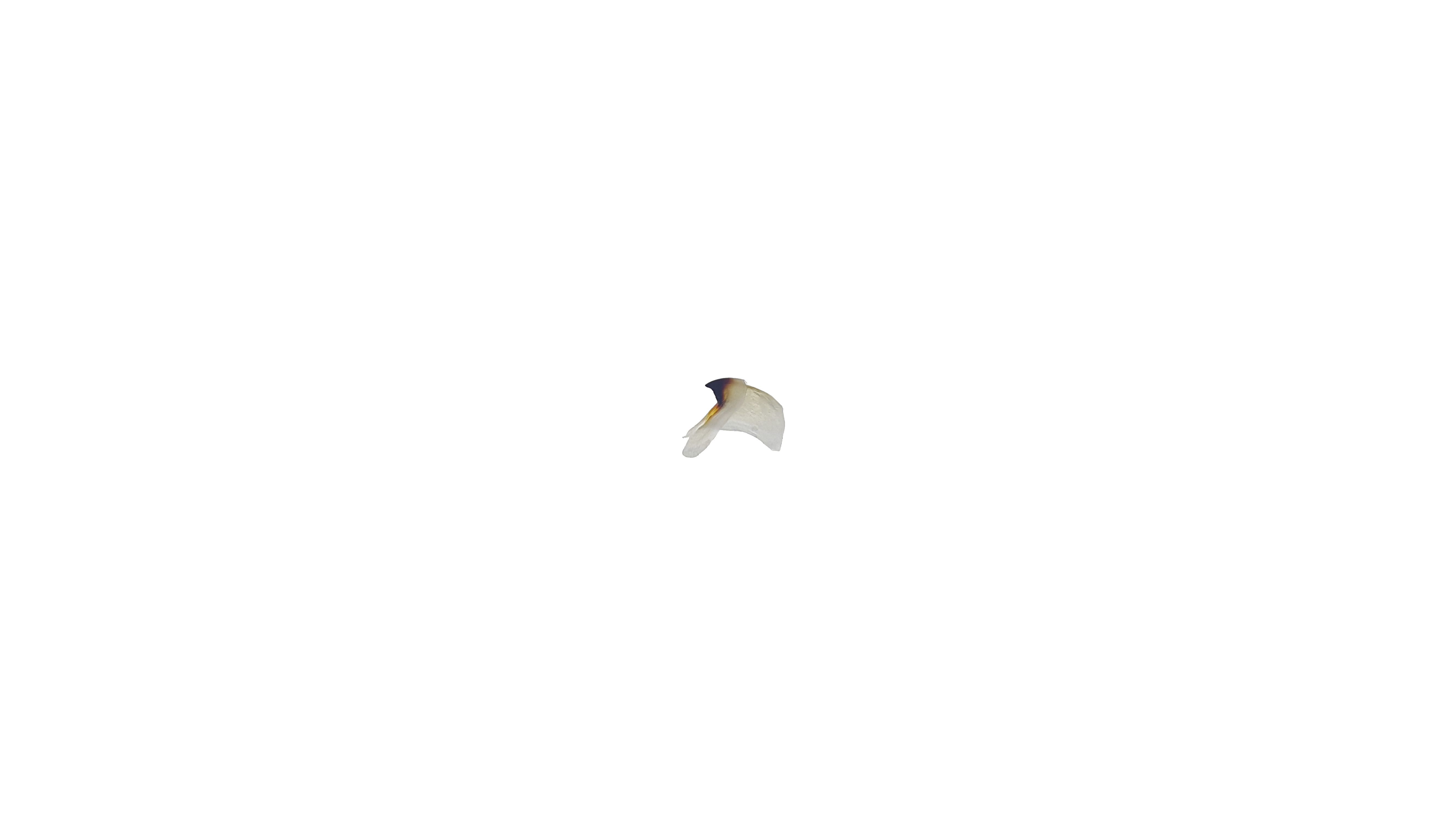

Supplement: Supplemental Information 1 — C2-Sepia aculeata, C3-Sepioteuthis lessoniana, C6-Sepia esculenta, O2-Amphioctopus aegina, S1-Loliolus uyii, S3-Uroteuthis chinensis, S4-Uroteuthis edulis [file peerj-09-11825-s001.zip › _Preprocessing_Lower_Beak/S3/L-l-S3-18.jpg]

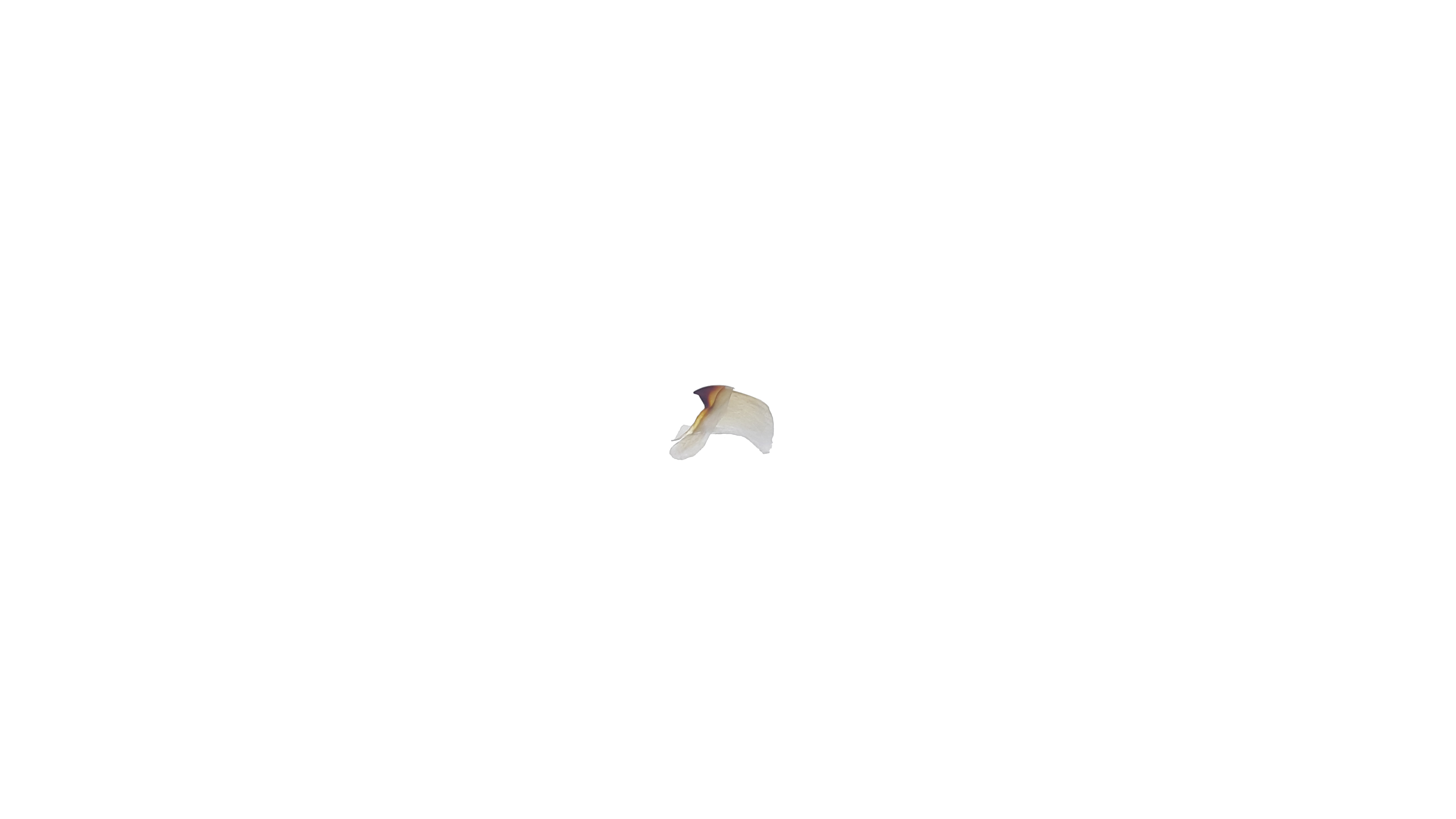

Supplement: Supplemental Information 1 — C2-Sepia aculeata, C3-Sepioteuthis lessoniana, C6-Sepia esculenta, O2-Amphioctopus aegina, S1-Loliolus uyii, S3-Uroteuthis chinensis, S4-Uroteuthis edulis [file peerj-09-11825-s001.zip › _Preprocessing_Lower_Beak/S3/L-l-S3-19.jpg]

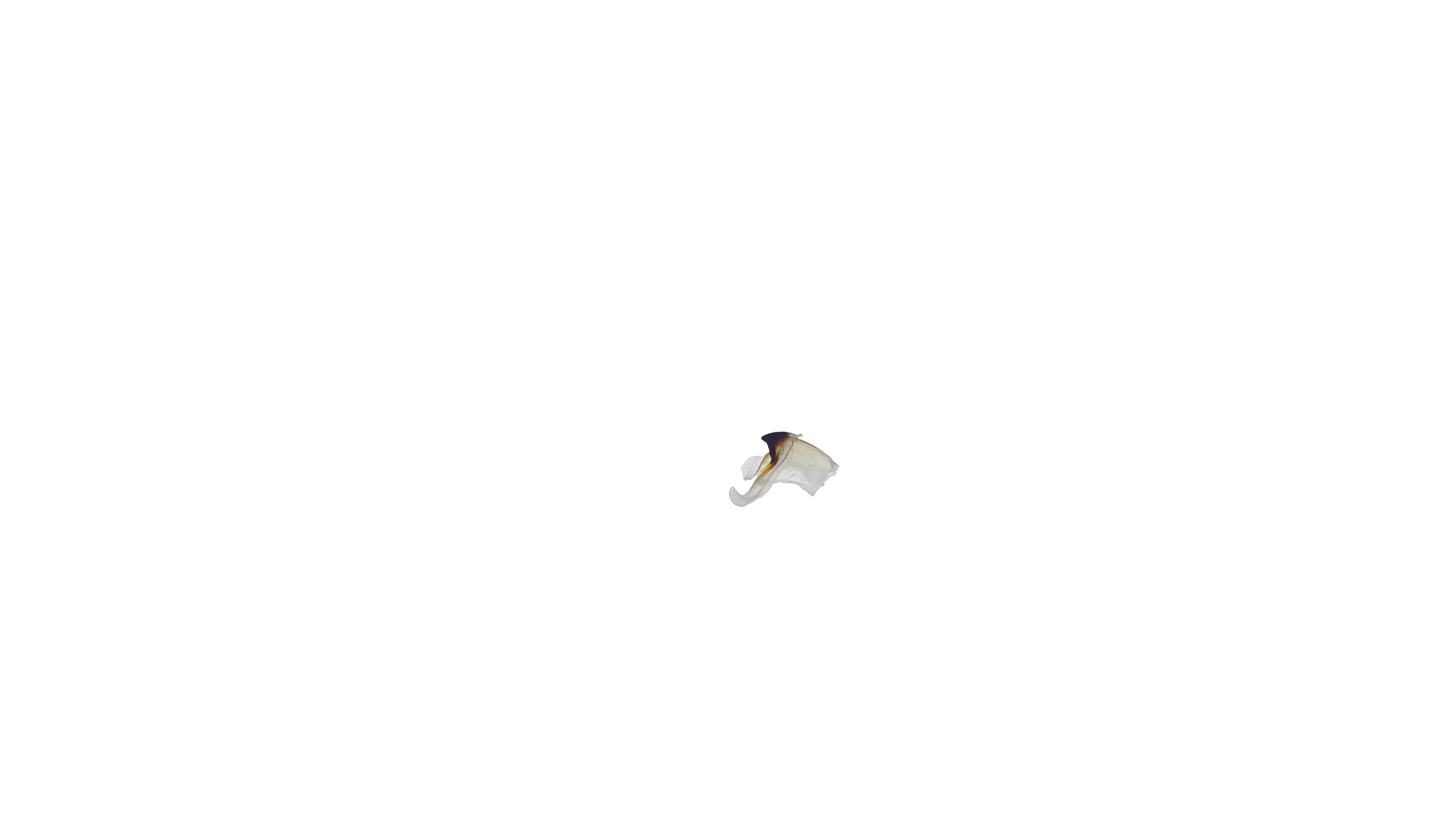

Supplement: Supplemental Information 1 — C2-Sepia aculeata, C3-Sepioteuthis lessoniana, C6-Sepia esculenta, O2-Amphioctopus aegina, S1-Loliolus uyii, S3-Uroteuthis chinensis, S4-Uroteuthis edulis [file peerj-09-11825-s001.zip › _Preprocessing_Lower_Beak/S3/L-l-S3-2.jpg]

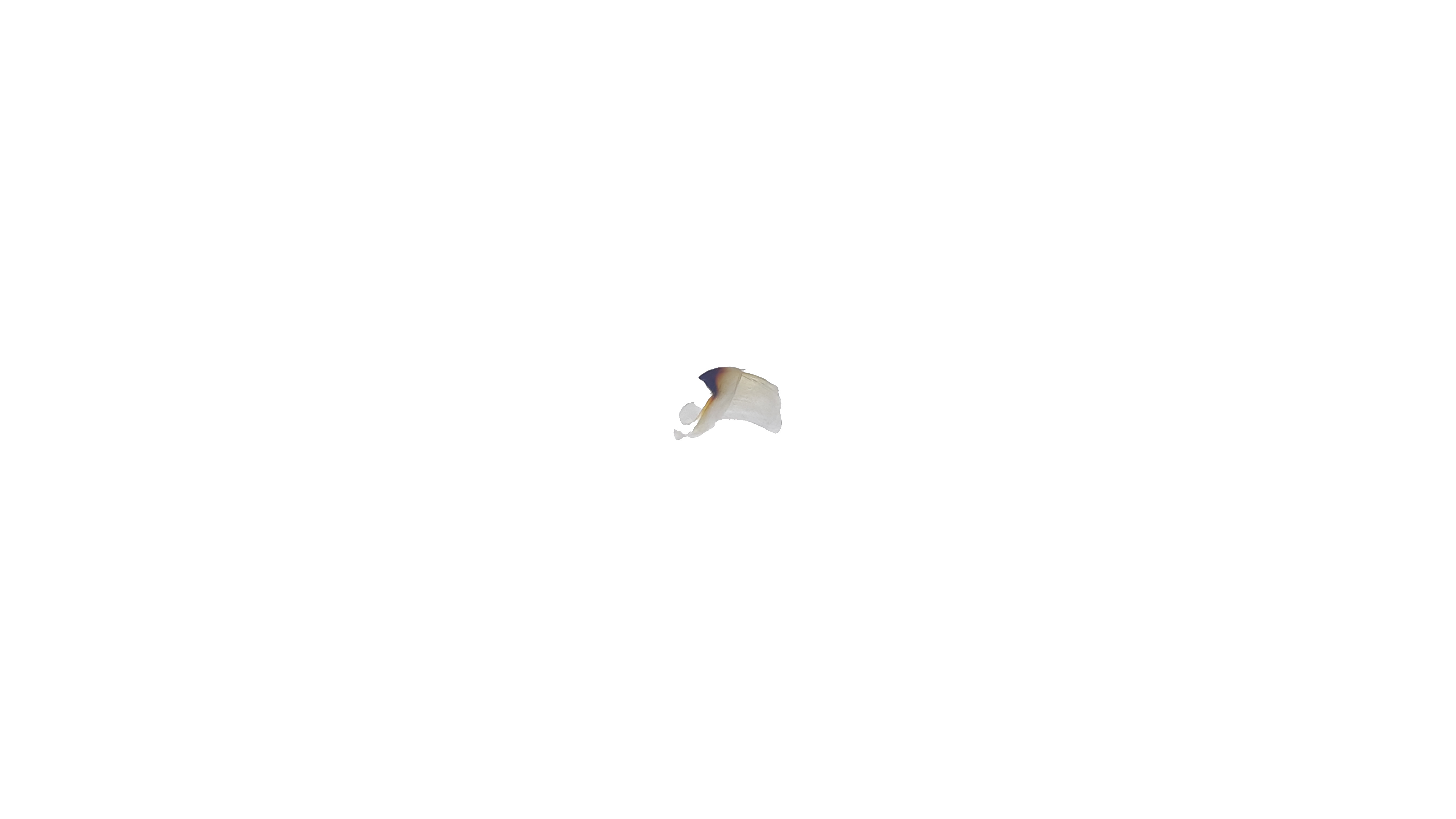

Supplement: Supplemental Information 1 — C2-Sepia aculeata, C3-Sepioteuthis lessoniana, C6-Sepia esculenta, O2-Amphioctopus aegina, S1-Loliolus uyii, S3-Uroteuthis chinensis, S4-Uroteuthis edulis [file peerj-09-11825-s001.zip › _Preprocessing_Lower_Beak/S3/L-l-S3-20.jpg]

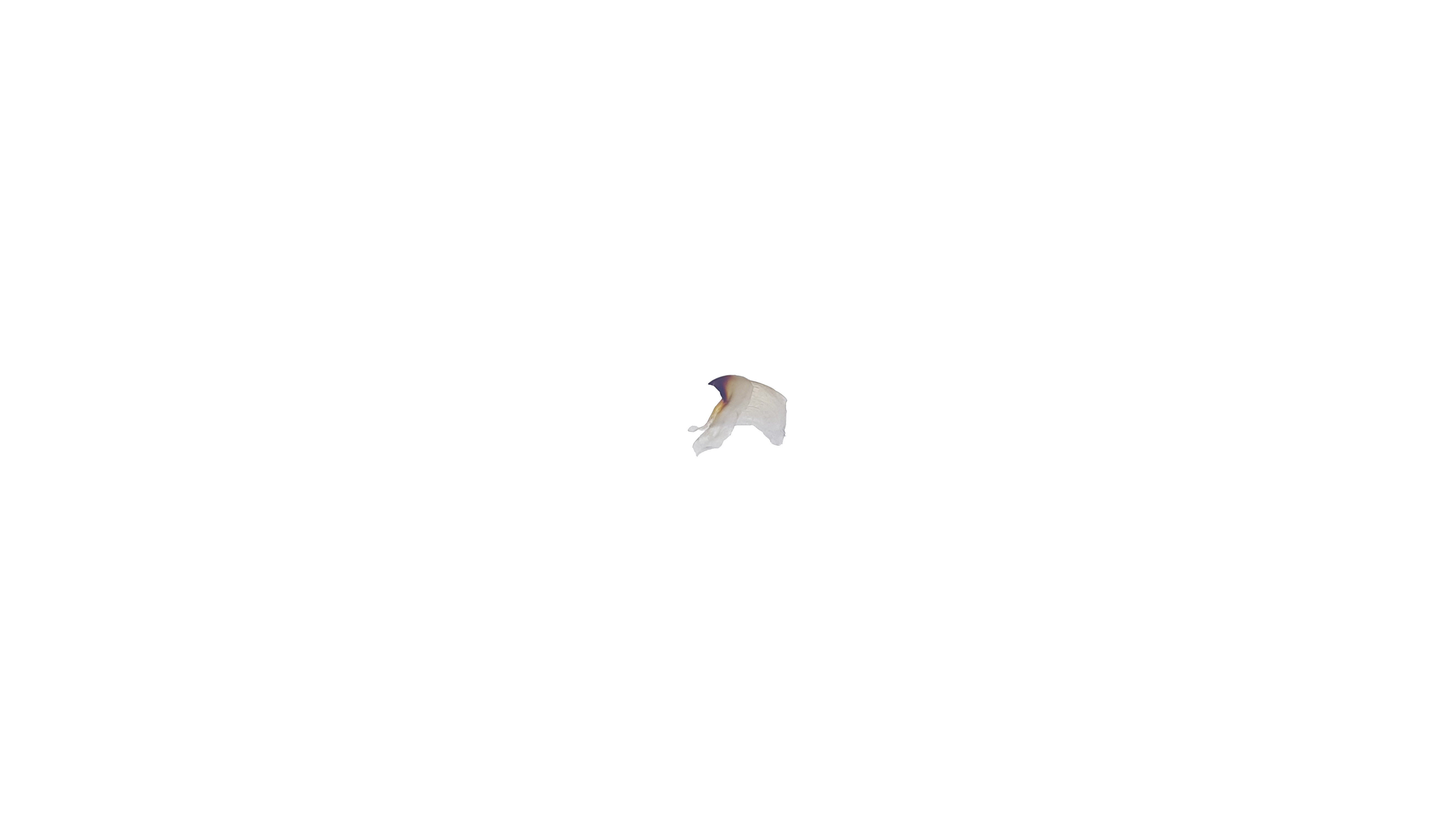

Supplement: Supplemental Information 1 — C2-Sepia aculeata, C3-Sepioteuthis lessoniana, C6-Sepia esculenta, O2-Amphioctopus aegina, S1-Loliolus uyii, S3-Uroteuthis chinensis, S4-Uroteuthis edulis [file peerj-09-11825-s001.zip › _Preprocessing_Lower_Beak/S3/L-l-S3-21.jpg]

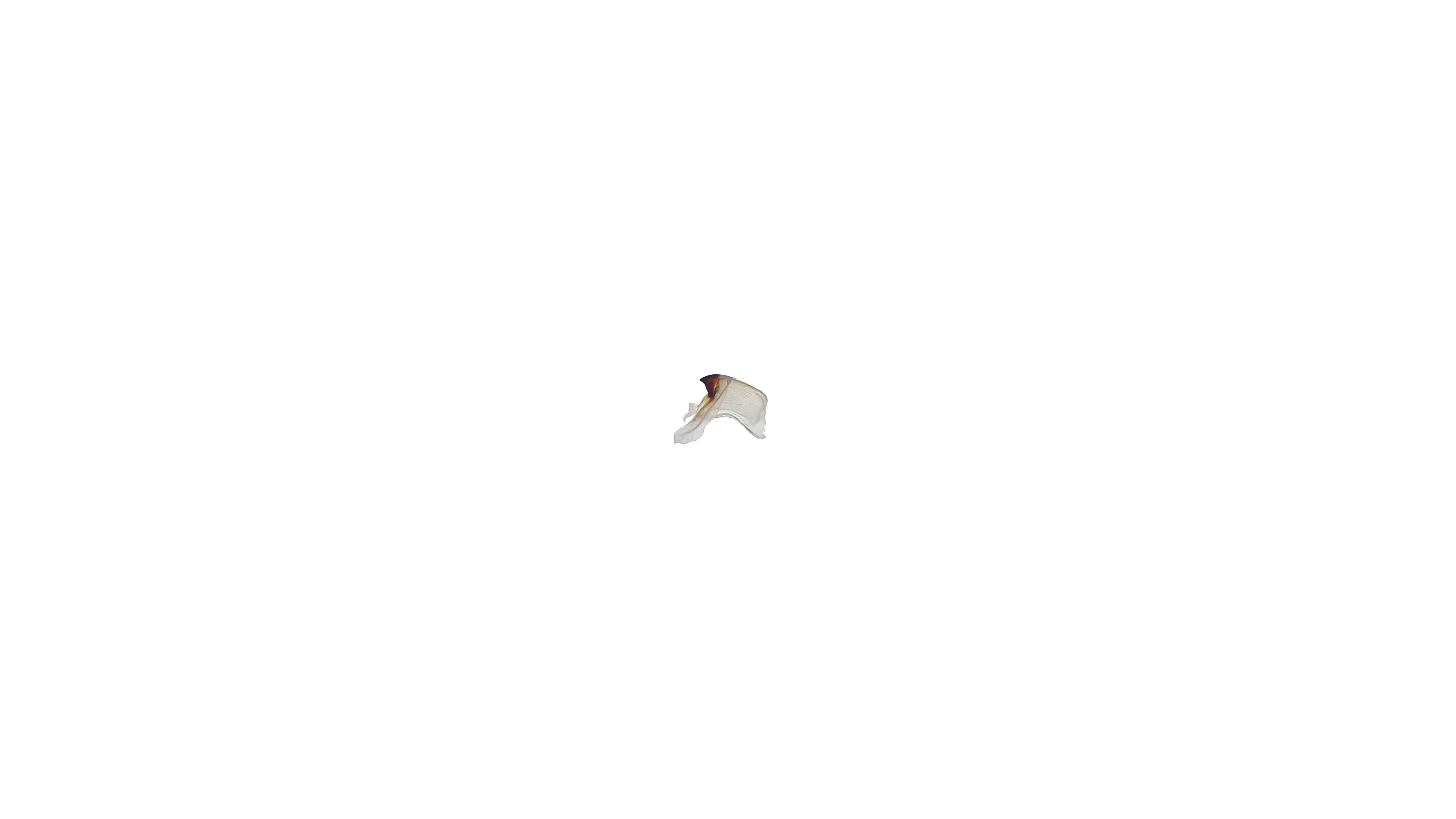

Supplement: Supplemental Information 1 — C2-Sepia aculeata, C3-Sepioteuthis lessoniana, C6-Sepia esculenta, O2-Amphioctopus aegina, S1-Loliolus uyii, S3-Uroteuthis chinensis, S4-Uroteuthis edulis [file peerj-09-11825-s001.zip › _Preprocessing_Lower_Beak/S3/L-l-S3-3.jpg]

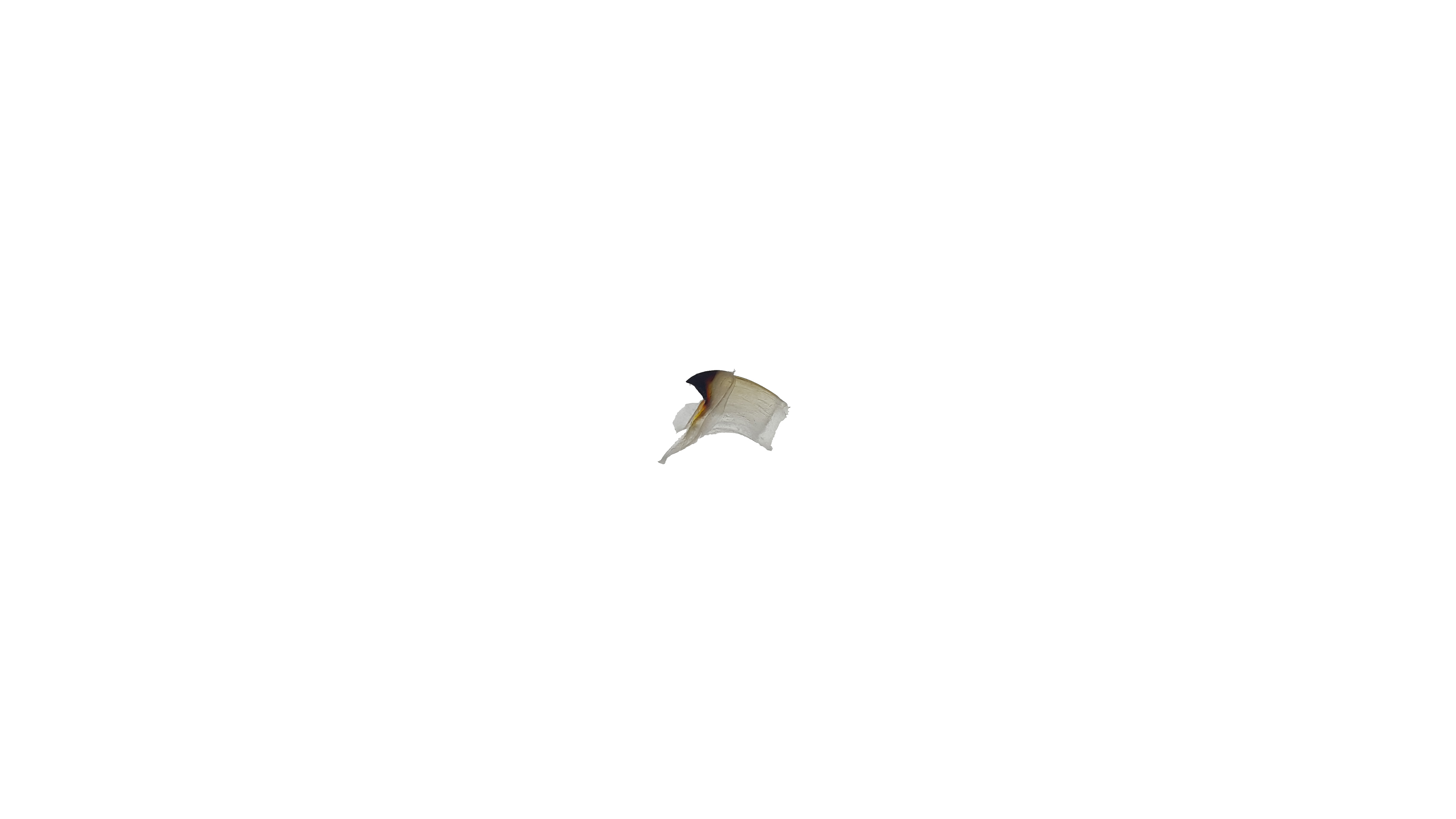

Supplement: Supplemental Information 1 — C2-Sepia aculeata, C3-Sepioteuthis lessoniana, C6-Sepia esculenta, O2-Amphioctopus aegina, S1-Loliolus uyii, S3-Uroteuthis chinensis, S4-Uroteuthis edulis [file peerj-09-11825-s001.zip › _Preprocessing_Lower_Beak/S3/L-l-S3-4.jpg]

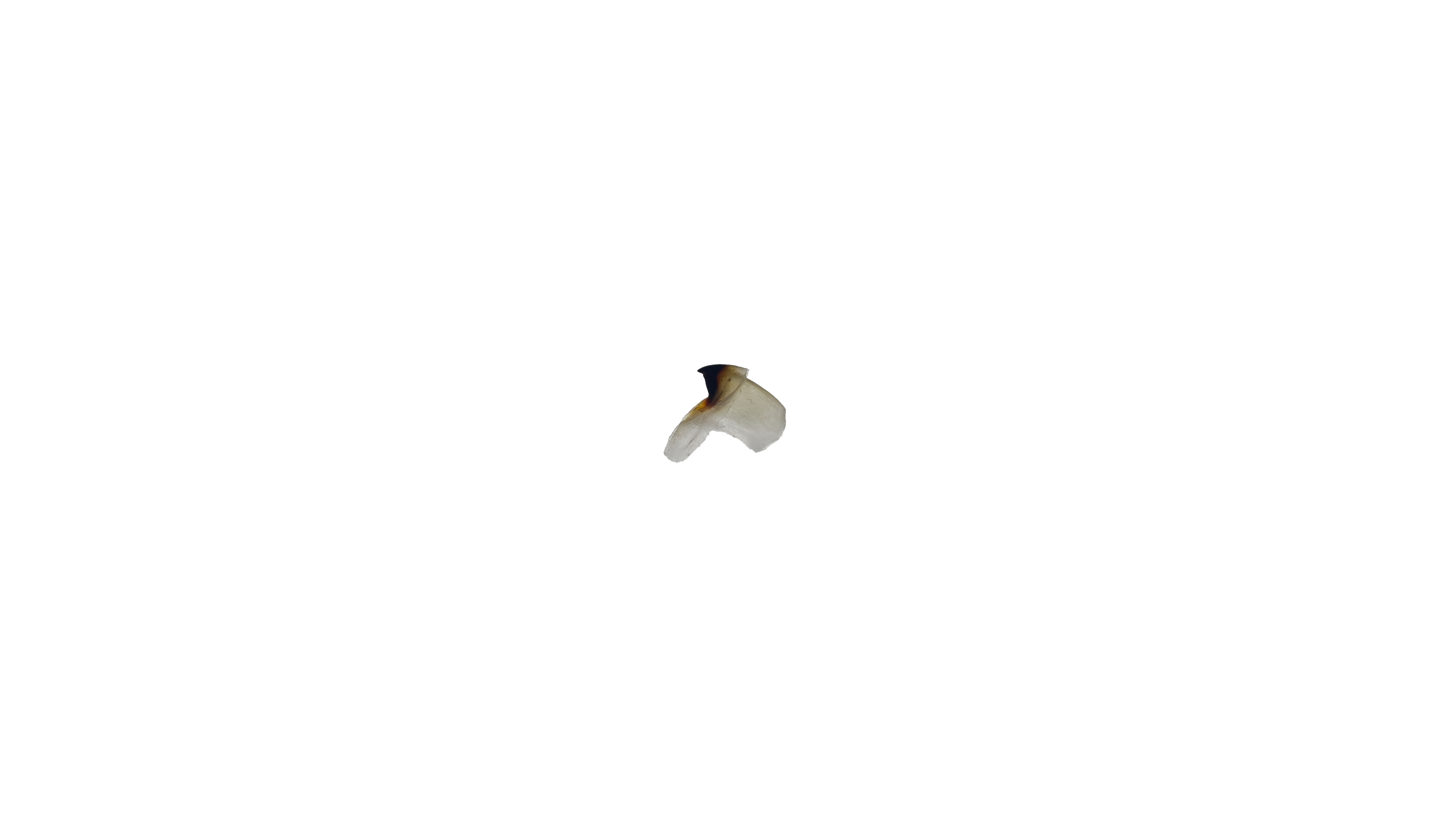

Supplement: Supplemental Information 1 — C2-Sepia aculeata, C3-Sepioteuthis lessoniana, C6-Sepia esculenta, O2-Amphioctopus aegina, S1-Loliolus uyii, S3-Uroteuthis chinensis, S4-Uroteuthis edulis [file peerj-09-11825-s001.zip › _Preprocessing_Lower_Beak/S3/L-l-S3-5.jpg]

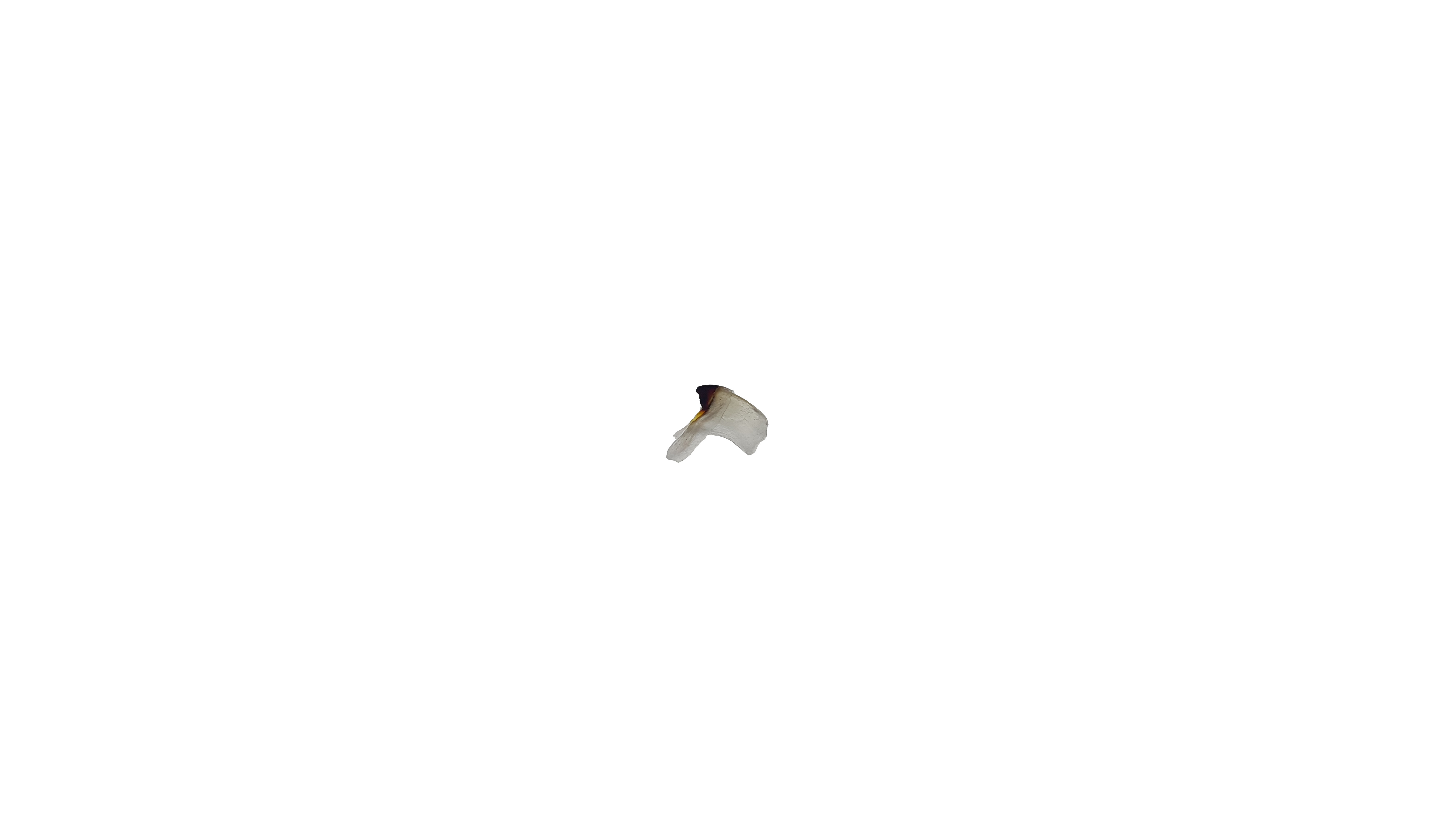

Supplement: Supplemental Information 1 — C2-Sepia aculeata, C3-Sepioteuthis lessoniana, C6-Sepia esculenta, O2-Amphioctopus aegina, S1-Loliolus uyii, S3-Uroteuthis chinensis, S4-Uroteuthis edulis [file peerj-09-11825-s001.zip › _Preprocessing_Lower_Beak/S3/L-l-S3-6.jpg]

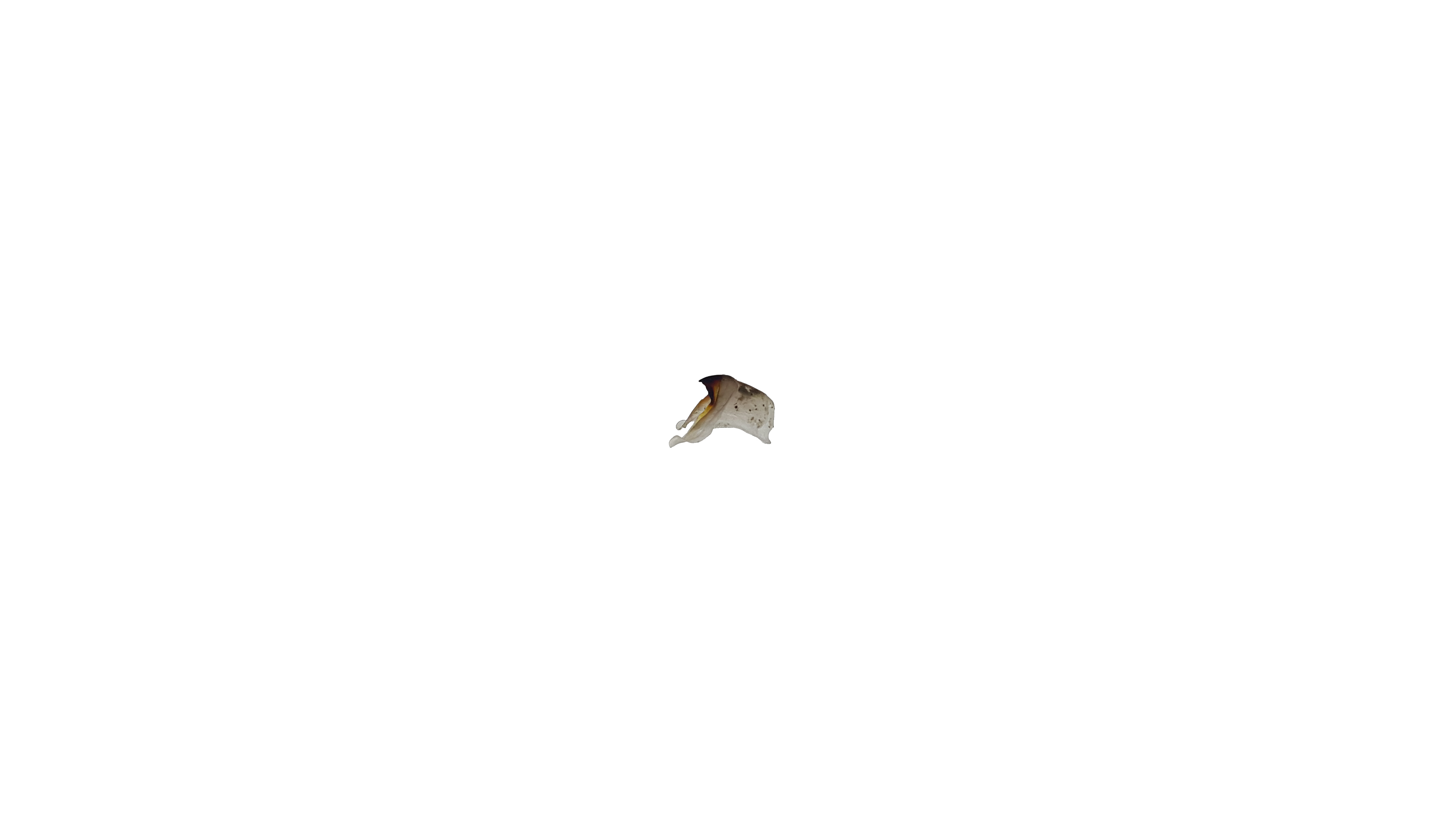

Supplement: Supplemental Information 1 — C2-Sepia aculeata, C3-Sepioteuthis lessoniana, C6-Sepia esculenta, O2-Amphioctopus aegina, S1-Loliolus uyii, S3-Uroteuthis chinensis, S4-Uroteuthis edulis [file peerj-09-11825-s001.zip › _Preprocessing_Lower_Beak/S3/L-l-S3-7.jpg]

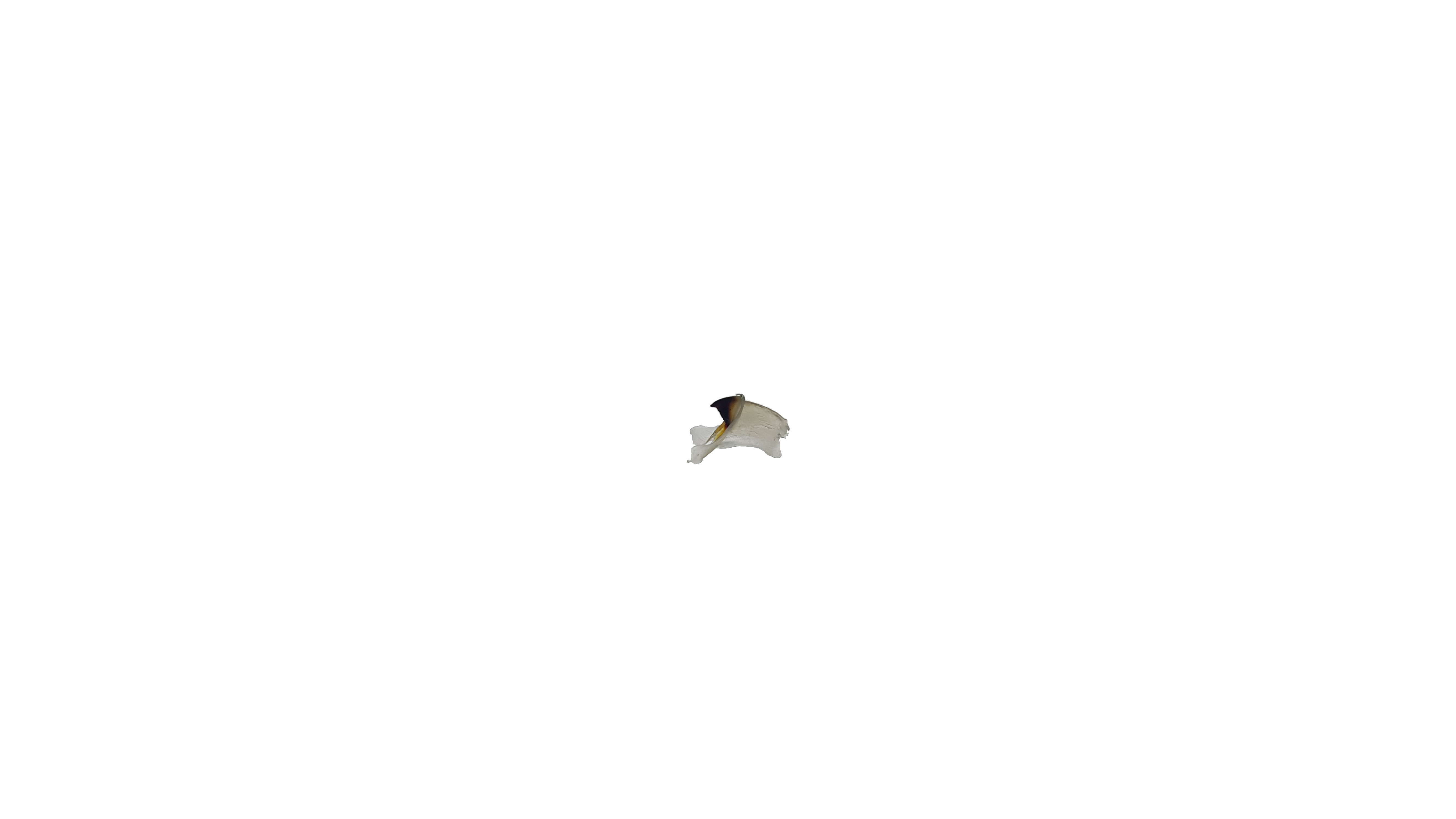

Supplement: Supplemental Information 1 — C2-Sepia aculeata, C3-Sepioteuthis lessoniana, C6-Sepia esculenta, O2-Amphioctopus aegina, S1-Loliolus uyii, S3-Uroteuthis chinensis, S4-Uroteuthis edulis [file peerj-09-11825-s001.zip › _Preprocessing_Lower_Beak/S3/L-l-S3-8.jpg]

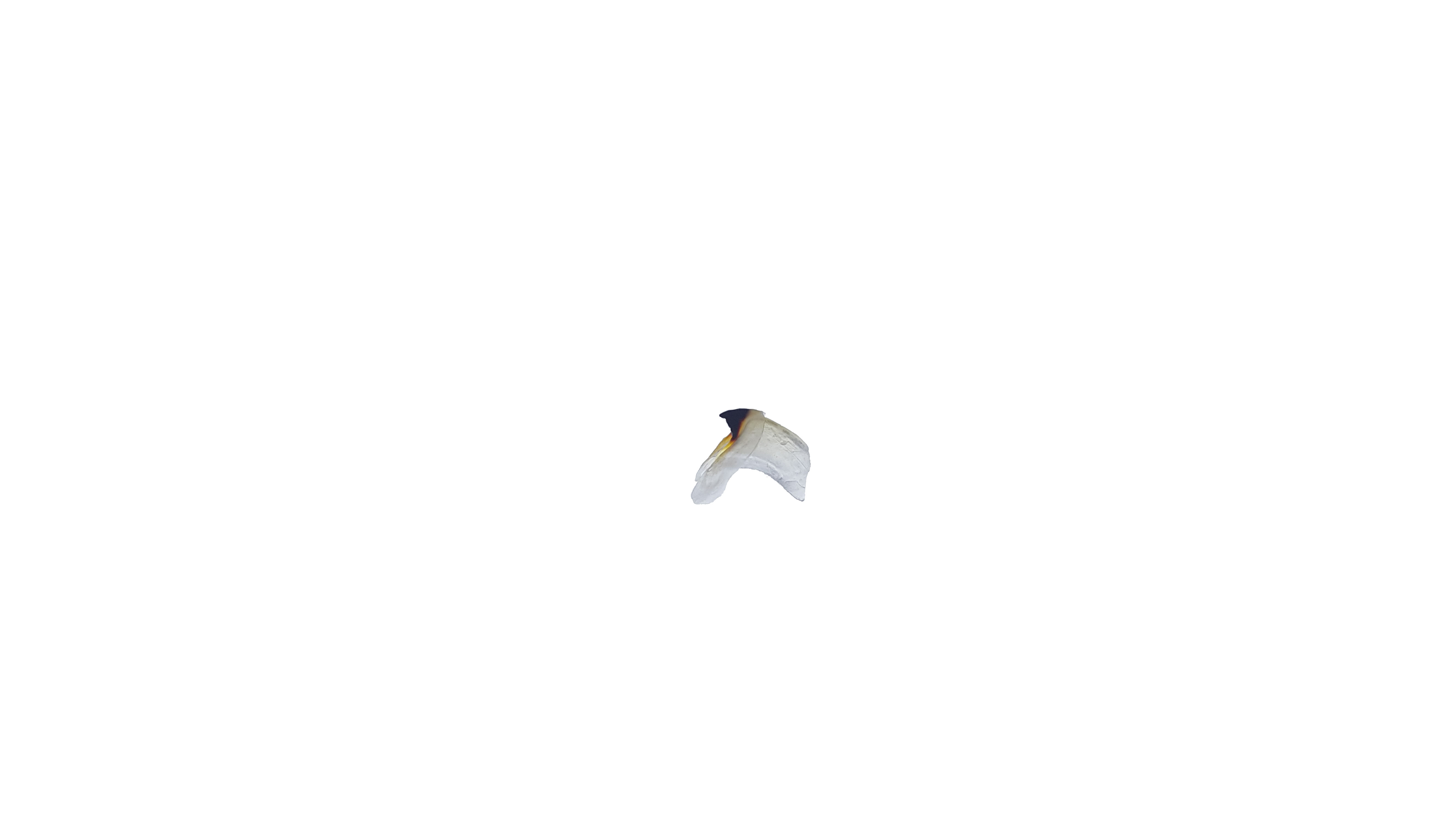

Supplement: Supplemental Information 1 — C2-Sepia aculeata, C3-Sepioteuthis lessoniana, C6-Sepia esculenta, O2-Amphioctopus aegina, S1-Loliolus uyii, S3-Uroteuthis chinensis, S4-Uroteuthis edulis [file peerj-09-11825-s001.zip › _Preprocessing_Lower_Beak/S4/L-l-S4-1.jpg]

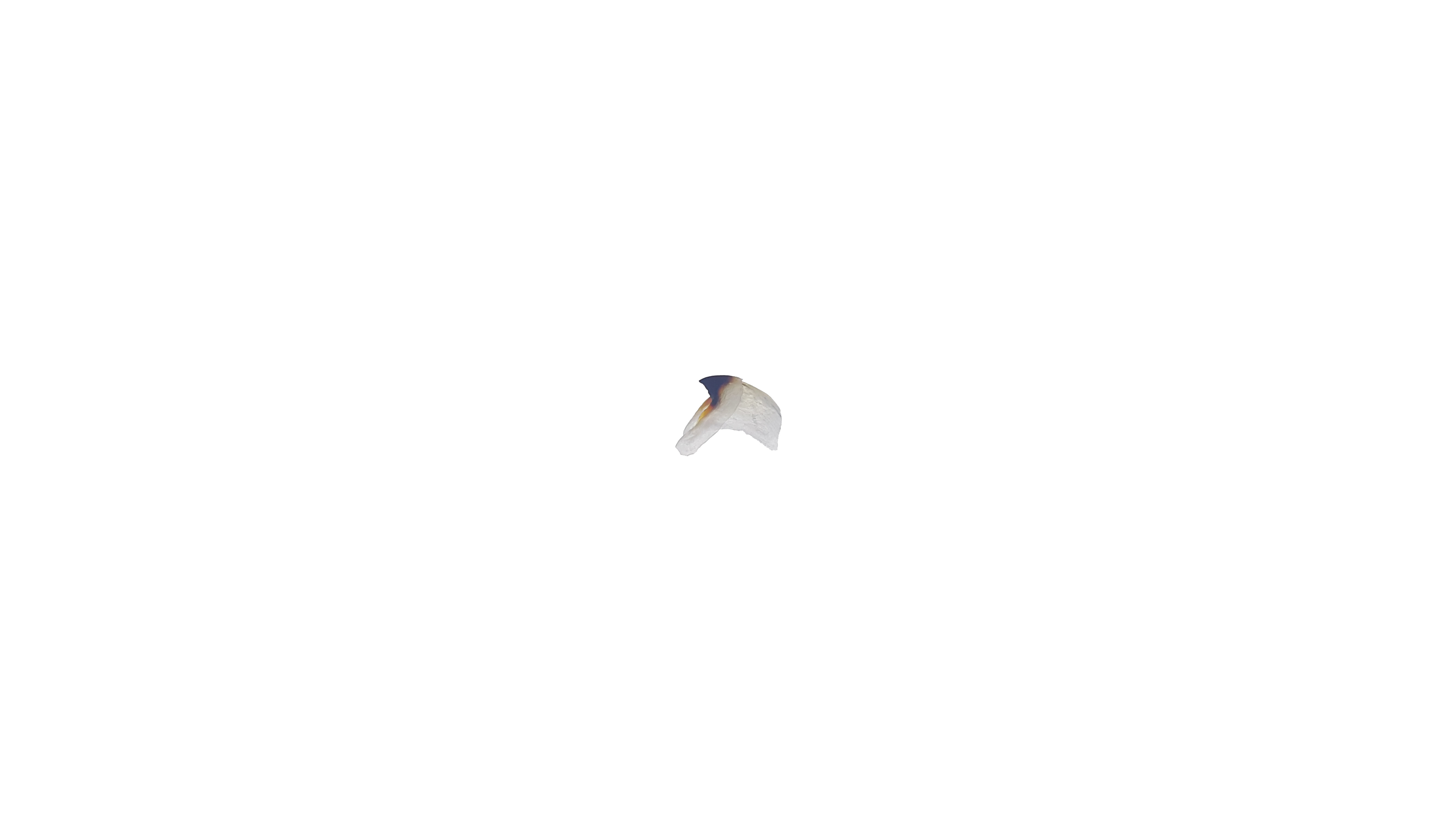

Supplement: Supplemental Information 1 — C2-Sepia aculeata, C3-Sepioteuthis lessoniana, C6-Sepia esculenta, O2-Amphioctopus aegina, S1-Loliolus uyii, S3-Uroteuthis chinensis, S4-Uroteuthis edulis [file peerj-09-11825-s001.zip › _Preprocessing_Lower_Beak/S4/L-l-S4-10.jpg]

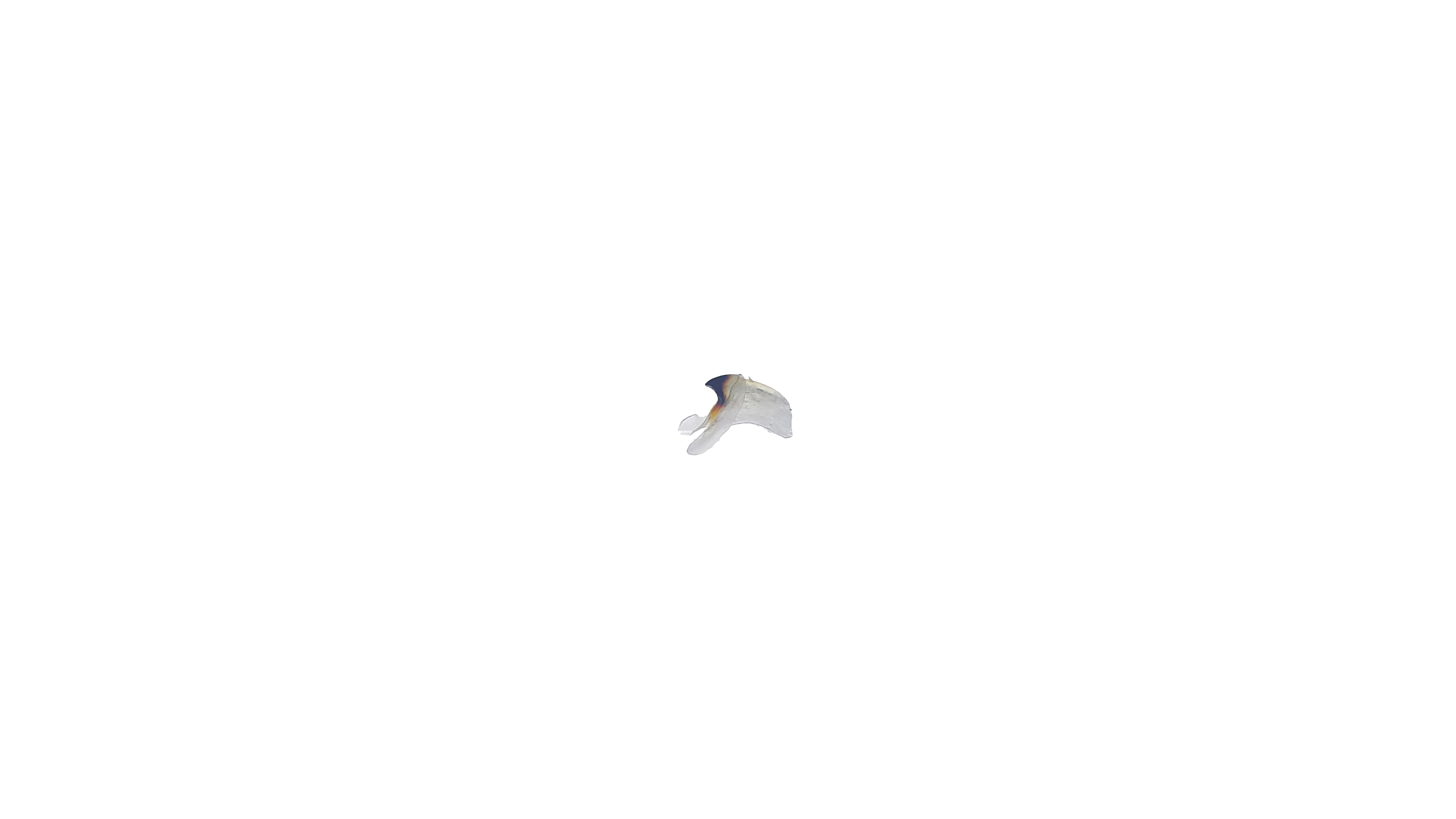

Supplement: Supplemental Information 1 — C2-Sepia aculeata, C3-Sepioteuthis lessoniana, C6-Sepia esculenta, O2-Amphioctopus aegina, S1-Loliolus uyii, S3-Uroteuthis chinensis, S4-Uroteuthis edulis [file peerj-09-11825-s001.zip › _Preprocessing_Lower_Beak/S4/L-l-S4-11.jpg]

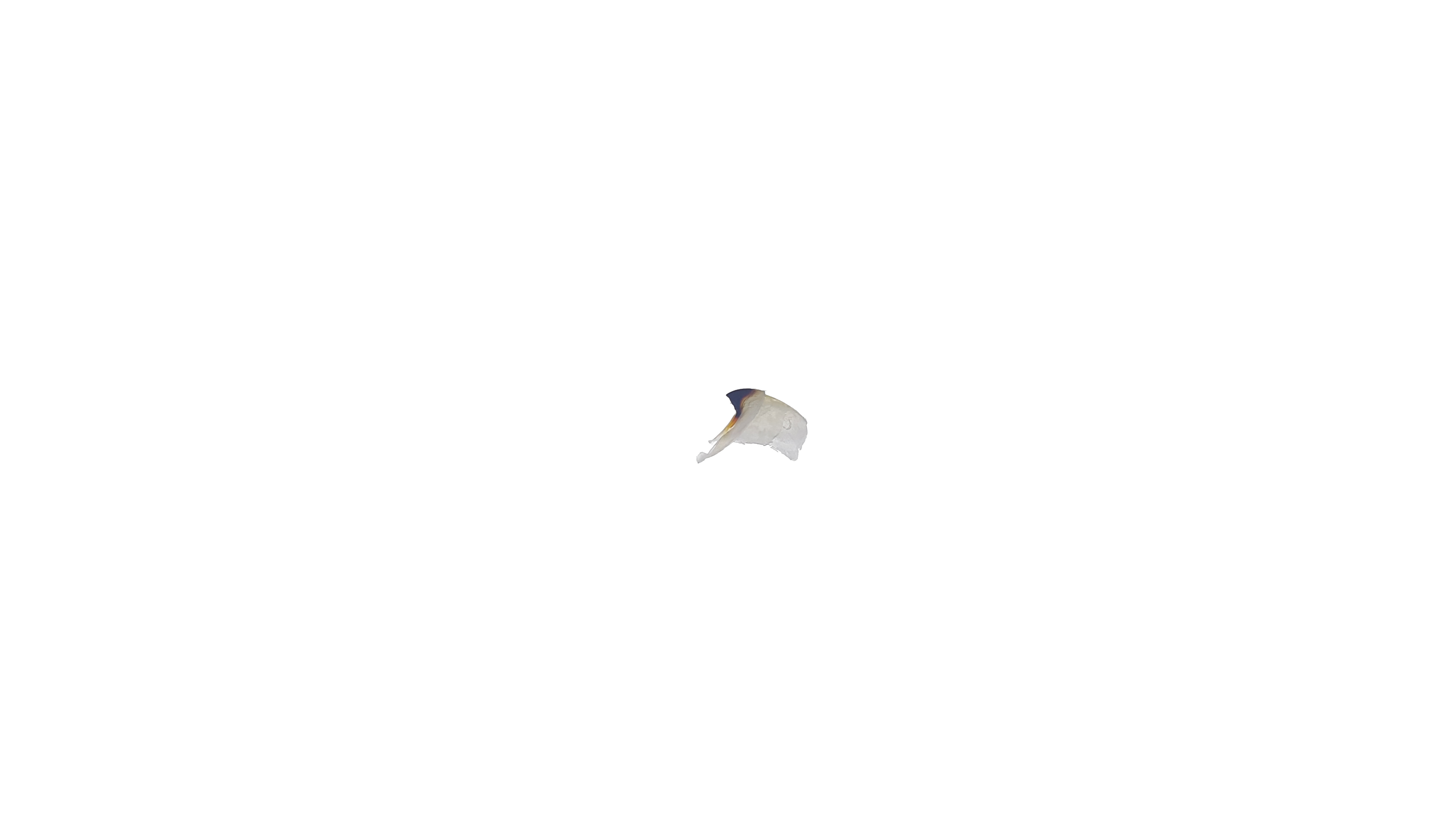

Supplement: Supplemental Information 1 — C2-Sepia aculeata, C3-Sepioteuthis lessoniana, C6-Sepia esculenta, O2-Amphioctopus aegina, S1-Loliolus uyii, S3-Uroteuthis chinensis, S4-Uroteuthis edulis [file peerj-09-11825-s001.zip › _Preprocessing_Lower_Beak/S4/L-l-S4-12.jpg]

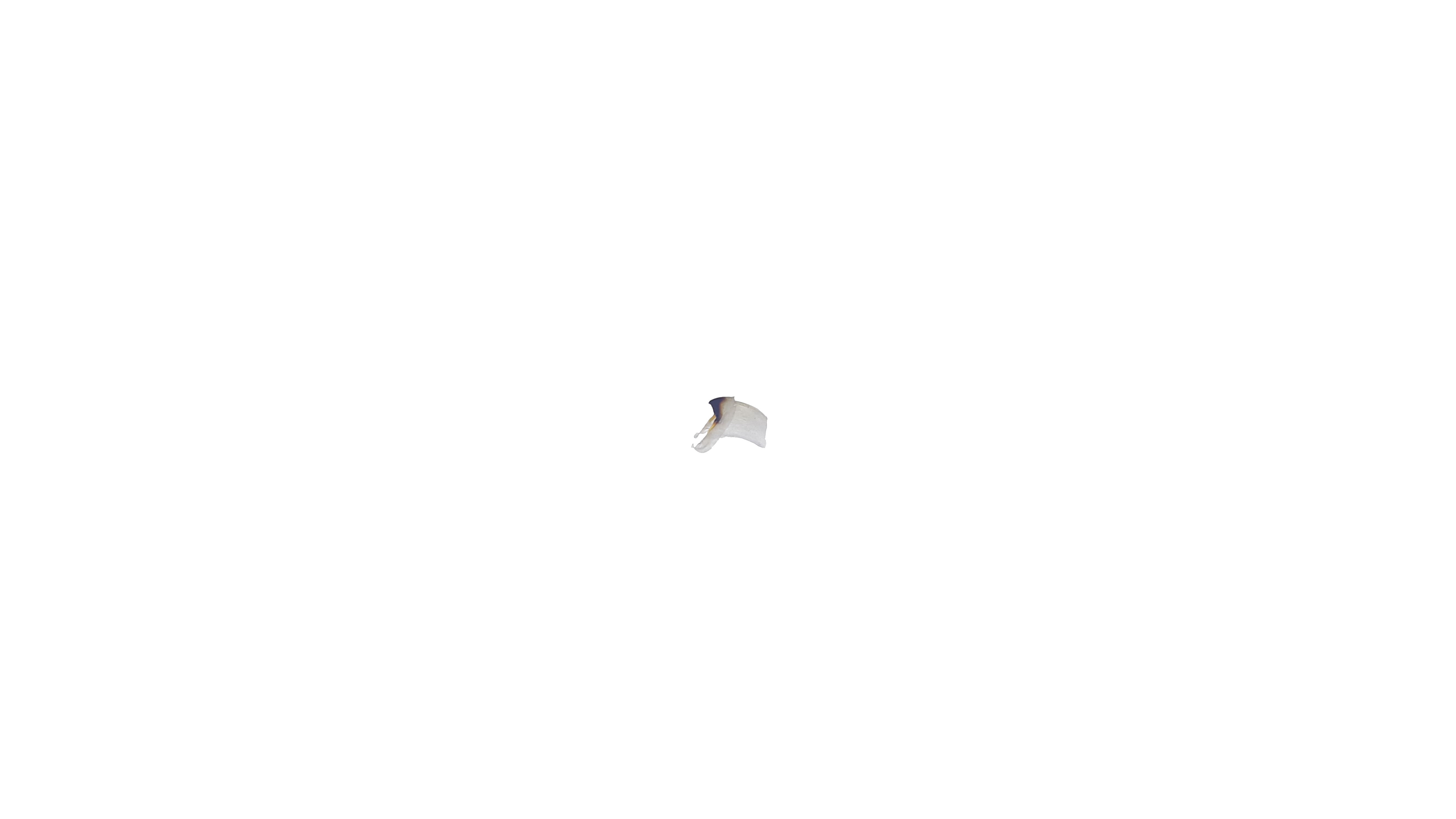

Supplement: Supplemental Information 1 — C2-Sepia aculeata, C3-Sepioteuthis lessoniana, C6-Sepia esculenta, O2-Amphioctopus aegina, S1-Loliolus uyii, S3-Uroteuthis chinensis, S4-Uroteuthis edulis [file peerj-09-11825-s001.zip › _Preprocessing_Lower_Beak/S4/L-l-S4-13.jpg]

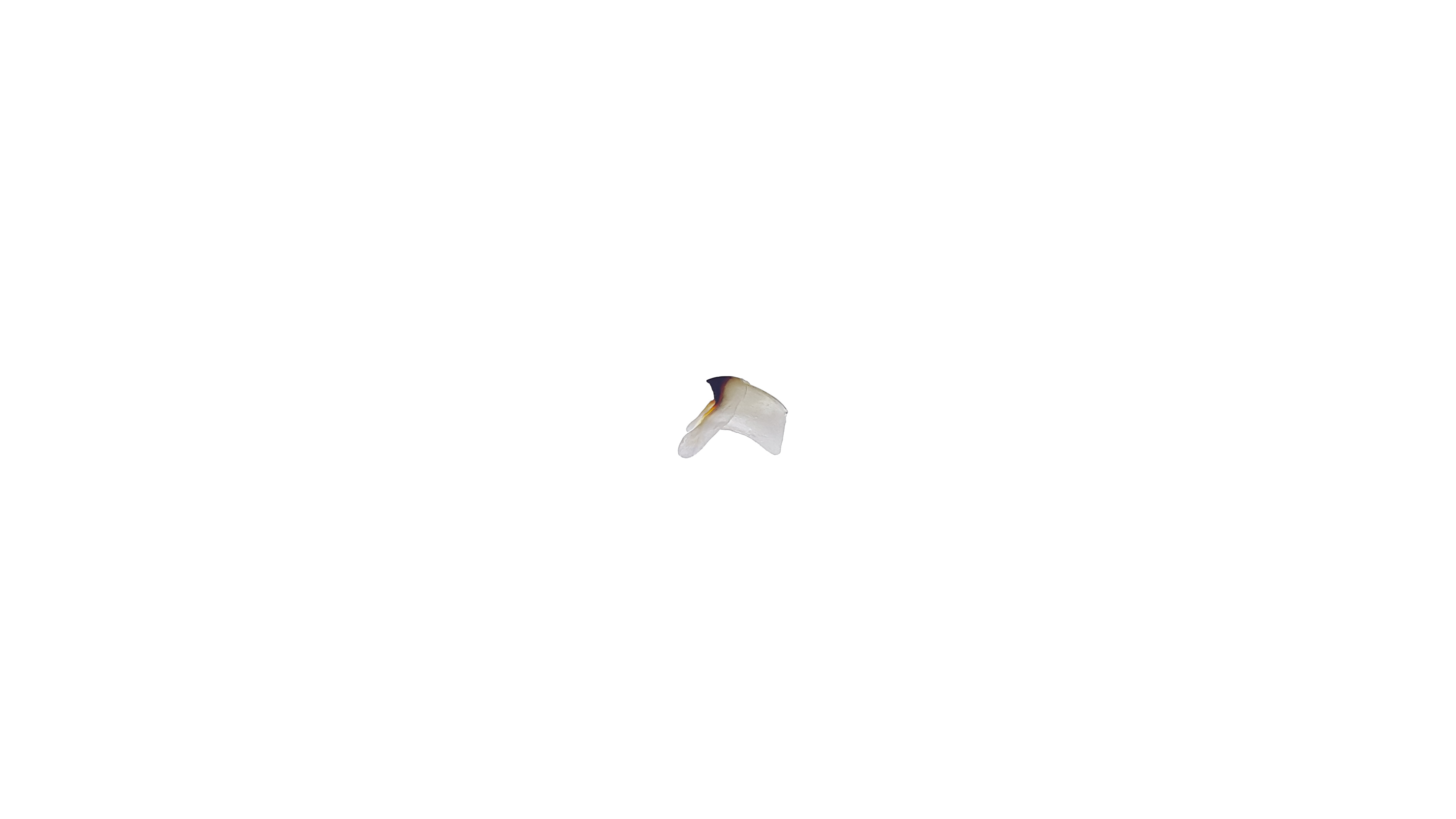

Supplement: Supplemental Information 1 — C2-Sepia aculeata, C3-Sepioteuthis lessoniana, C6-Sepia esculenta, O2-Amphioctopus aegina, S1-Loliolus uyii, S3-Uroteuthis chinensis, S4-Uroteuthis edulis [file peerj-09-11825-s001.zip › _Preprocessing_Lower_Beak/S4/L-l-S4-14.jpg]

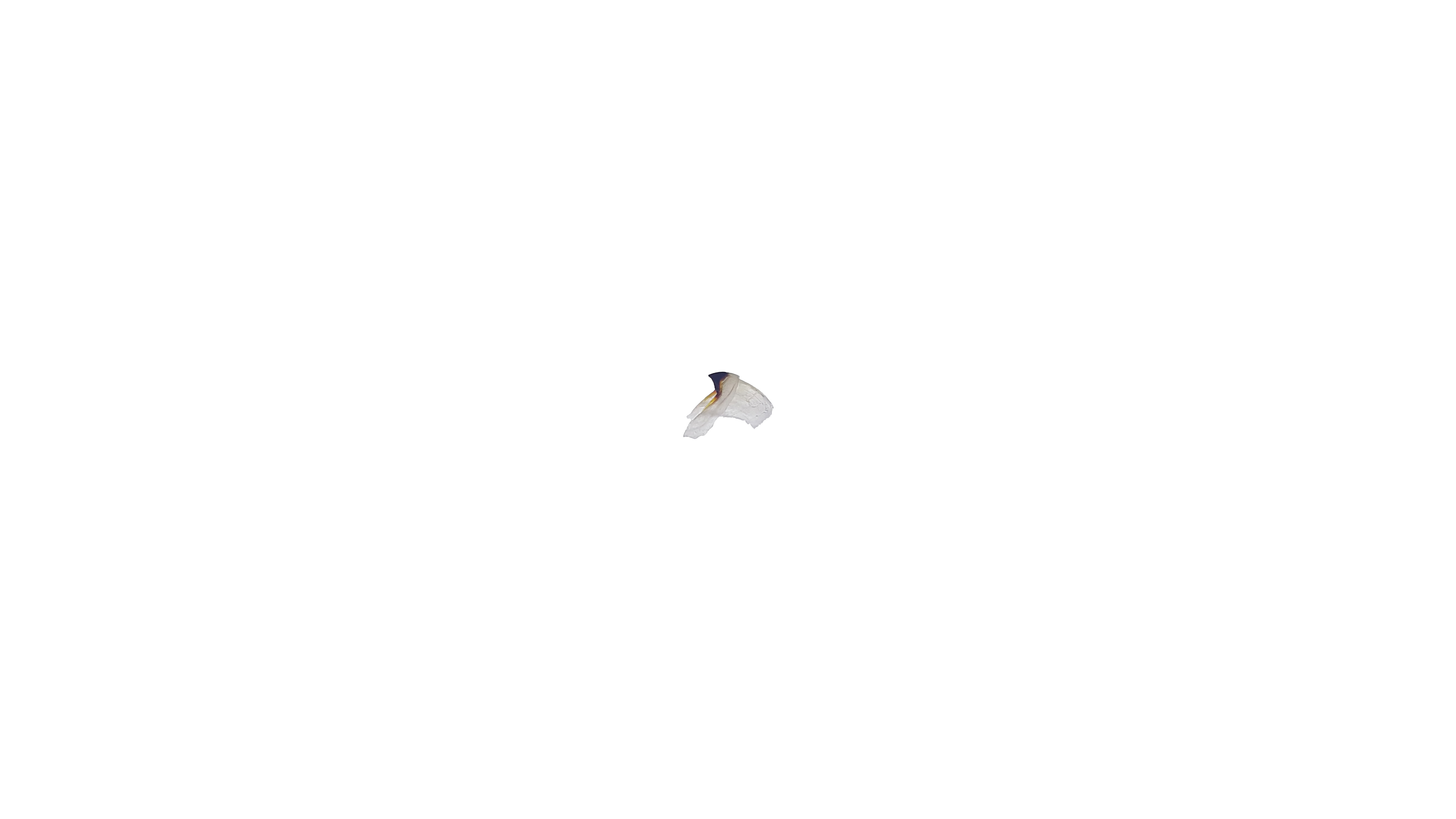

Supplement: Supplemental Information 1 — C2-Sepia aculeata, C3-Sepioteuthis lessoniana, C6-Sepia esculenta, O2-Amphioctopus aegina, S1-Loliolus uyii, S3-Uroteuthis chinensis, S4-Uroteuthis edulis [file peerj-09-11825-s001.zip › _Preprocessing_Lower_Beak/S4/L-l-S4-15.jpg]

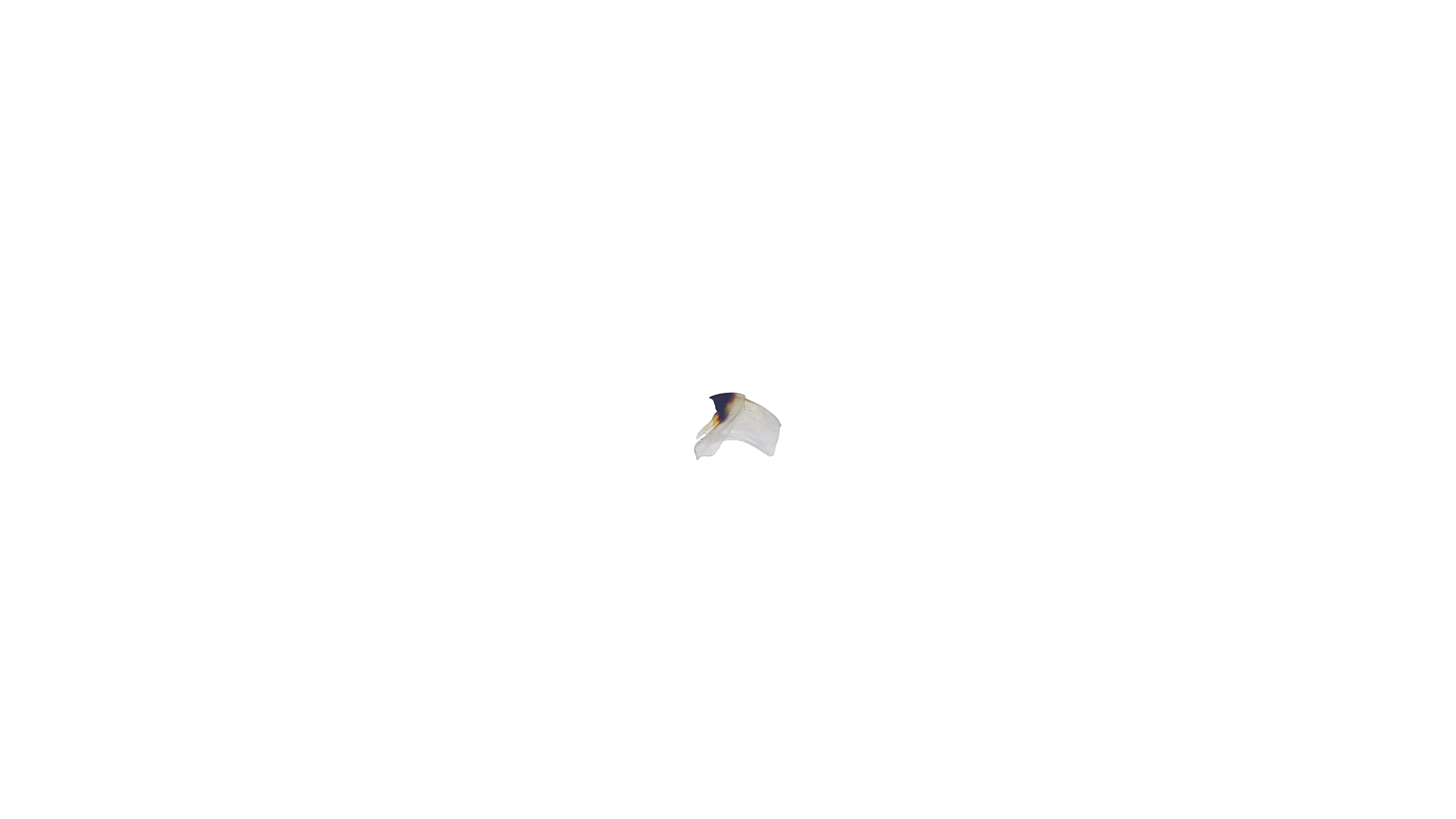

Supplement: Supplemental Information 1 — C2-Sepia aculeata, C3-Sepioteuthis lessoniana, C6-Sepia esculenta, O2-Amphioctopus aegina, S1-Loliolus uyii, S3-Uroteuthis chinensis, S4-Uroteuthis edulis [file peerj-09-11825-s001.zip › _Preprocessing_Lower_Beak/S4/L-l-S4-16.jpg]

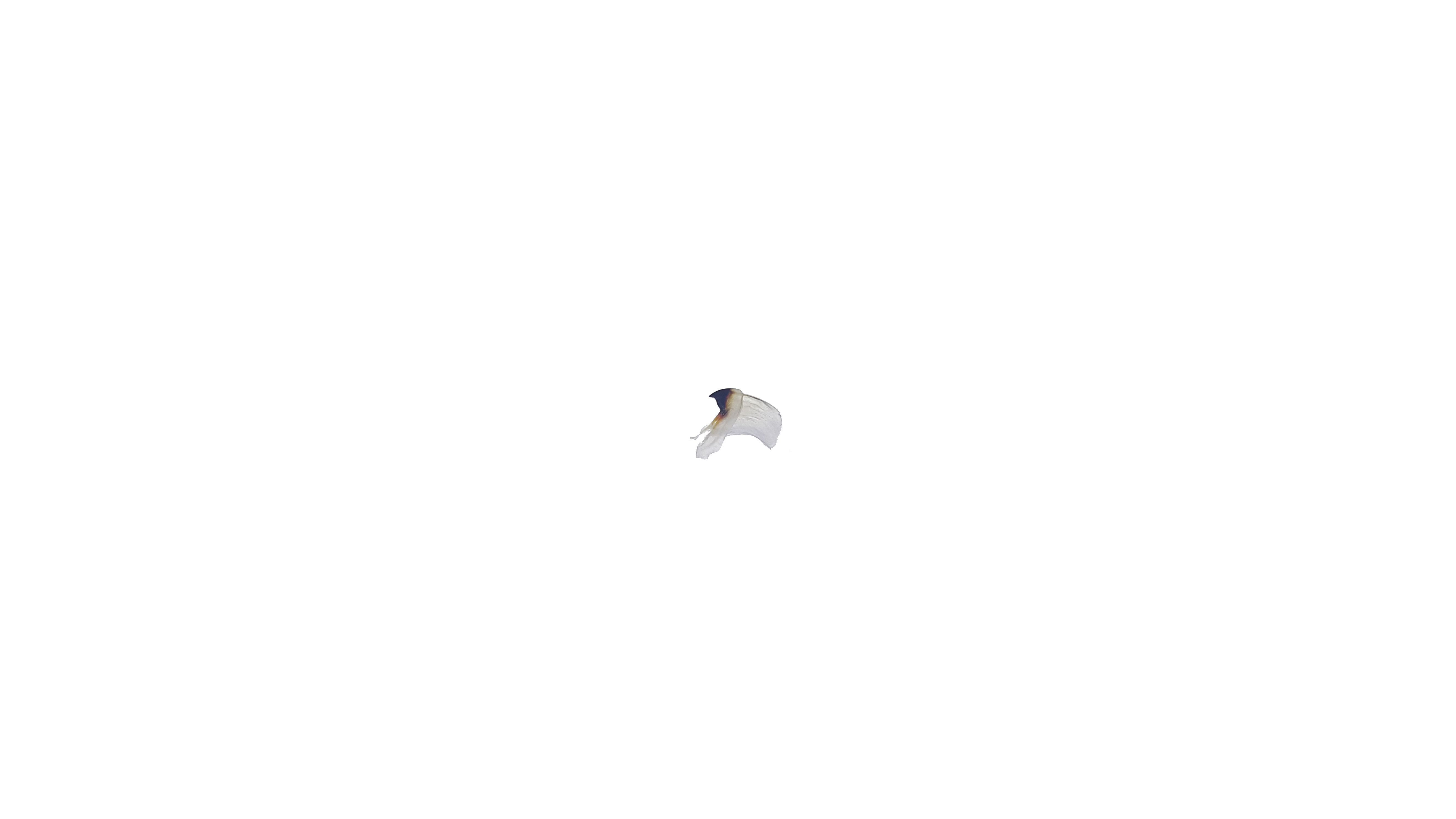

Supplement: Supplemental Information 1 — C2-Sepia aculeata, C3-Sepioteuthis lessoniana, C6-Sepia esculenta, O2-Amphioctopus aegina, S1-Loliolus uyii, S3-Uroteuthis chinensis, S4-Uroteuthis edulis [file peerj-09-11825-s001.zip › _Preprocessing_Lower_Beak/S4/L-l-S4-17.jpg]

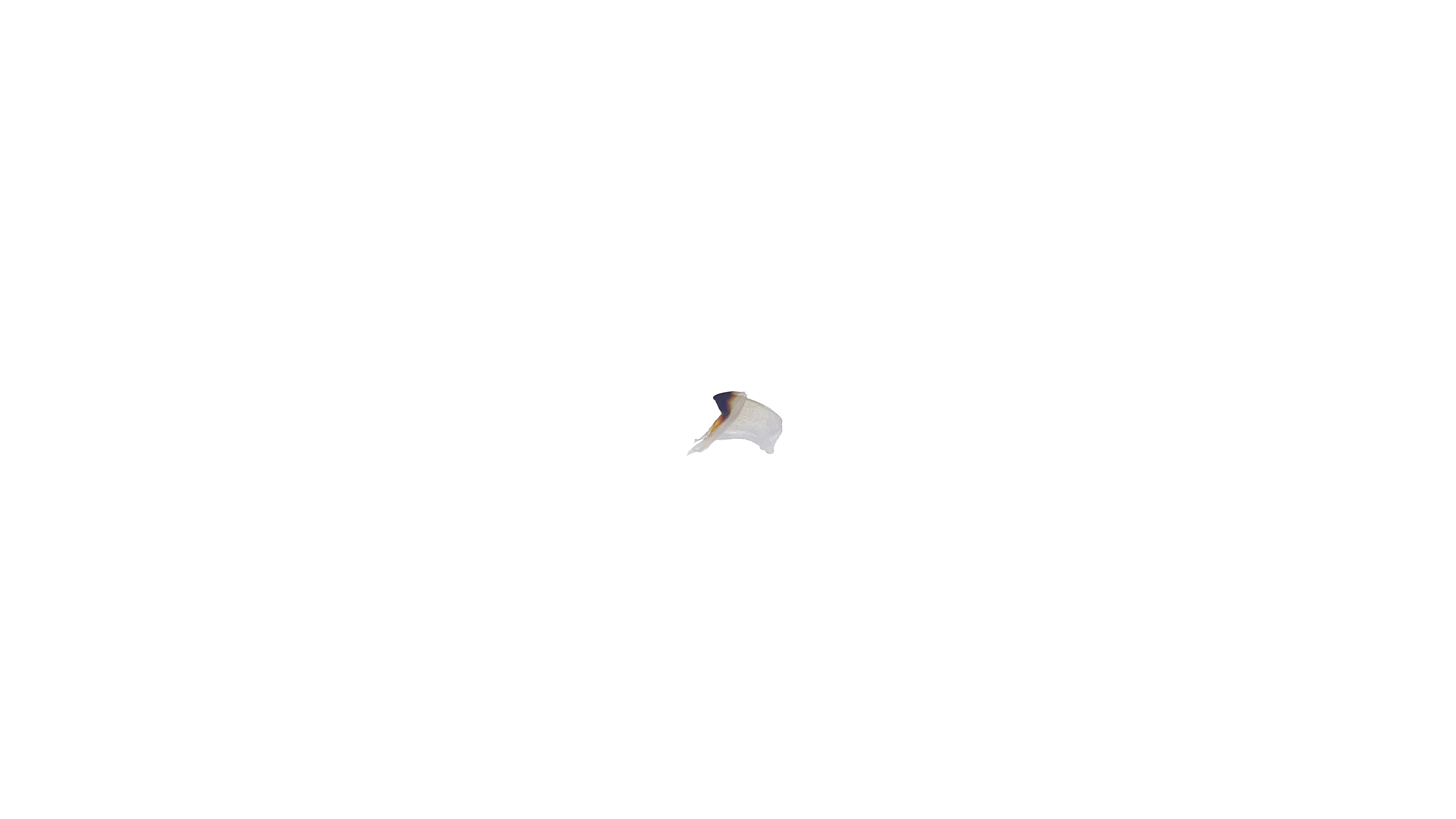

Supplement: Supplemental Information 1 — C2-Sepia aculeata, C3-Sepioteuthis lessoniana, C6-Sepia esculenta, O2-Amphioctopus aegina, S1-Loliolus uyii, S3-Uroteuthis chinensis, S4-Uroteuthis edulis [file peerj-09-11825-s001.zip › _Preprocessing_Lower_Beak/S4/L-l-S4-18.jpg]

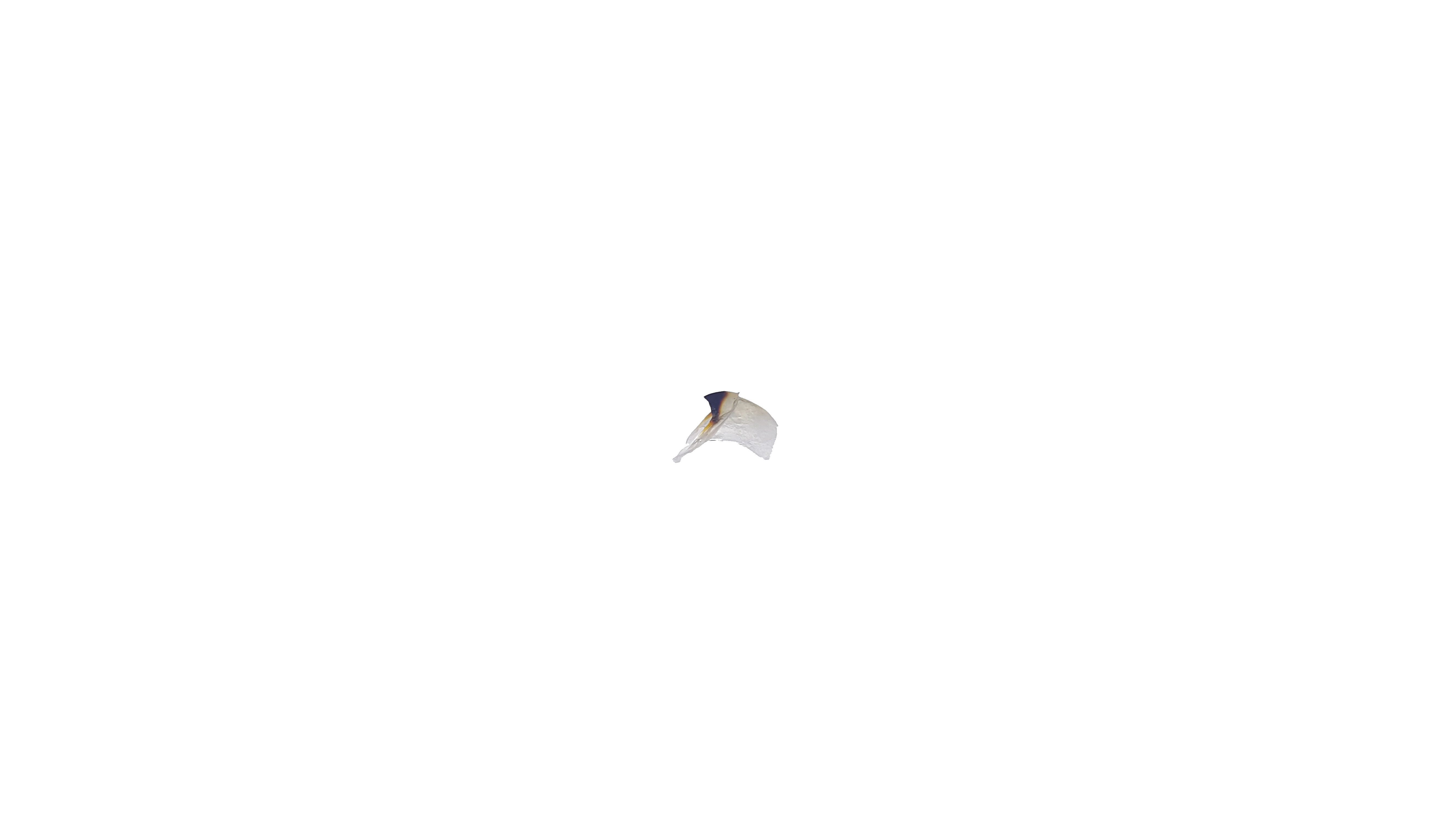

Supplement: Supplemental Information 1 — C2-Sepia aculeata, C3-Sepioteuthis lessoniana, C6-Sepia esculenta, O2-Amphioctopus aegina, S1-Loliolus uyii, S3-Uroteuthis chinensis, S4-Uroteuthis edulis [file peerj-09-11825-s001.zip › _Preprocessing_Lower_Beak/S4/L-l-S4-19.jpg]

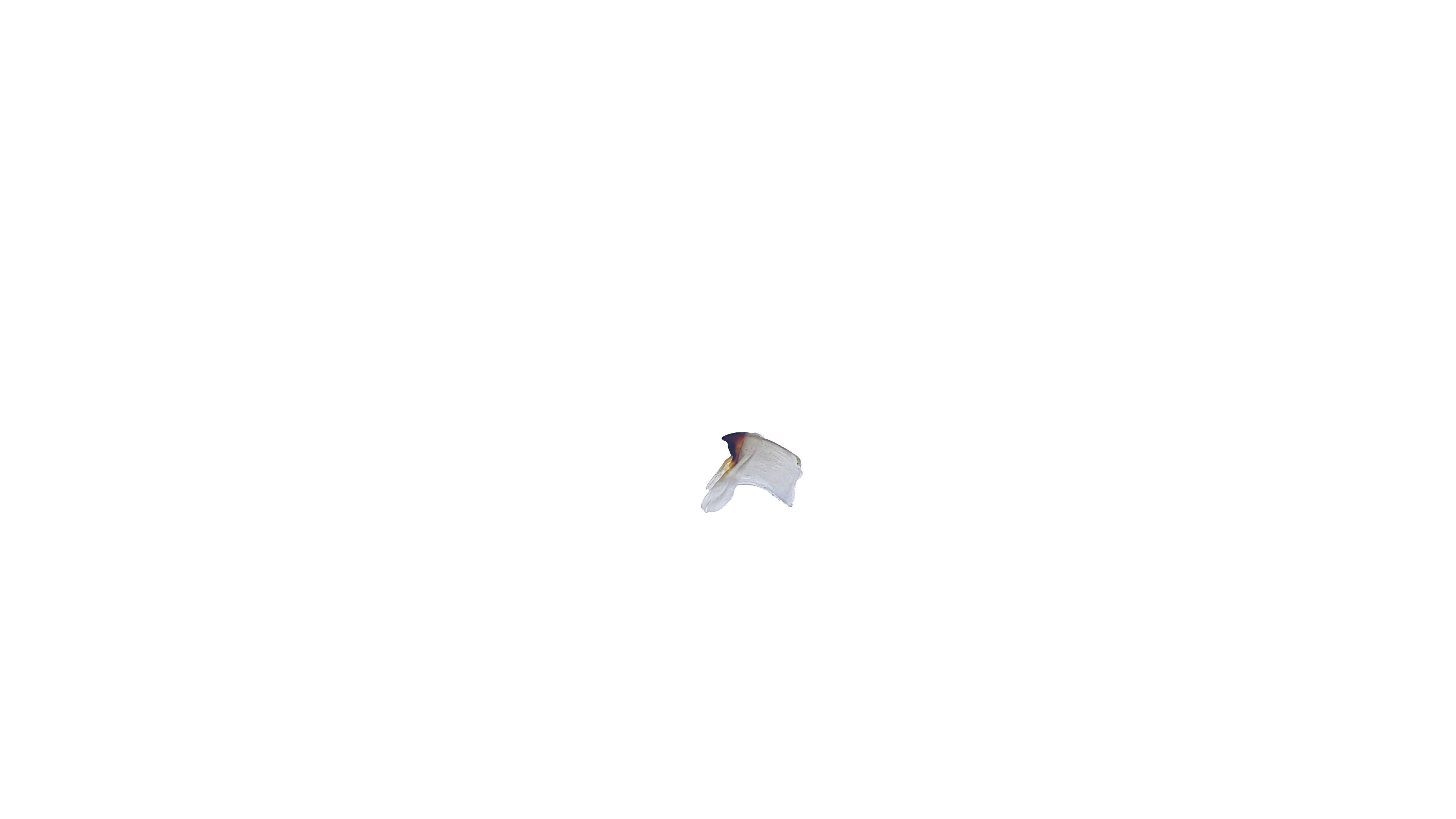

Supplement: Supplemental Information 1 — C2-Sepia aculeata, C3-Sepioteuthis lessoniana, C6-Sepia esculenta, O2-Amphioctopus aegina, S1-Loliolus uyii, S3-Uroteuthis chinensis, S4-Uroteuthis edulis [file peerj-09-11825-s001.zip › _Preprocessing_Lower_Beak/S4/L-l-S4-2.jpg]

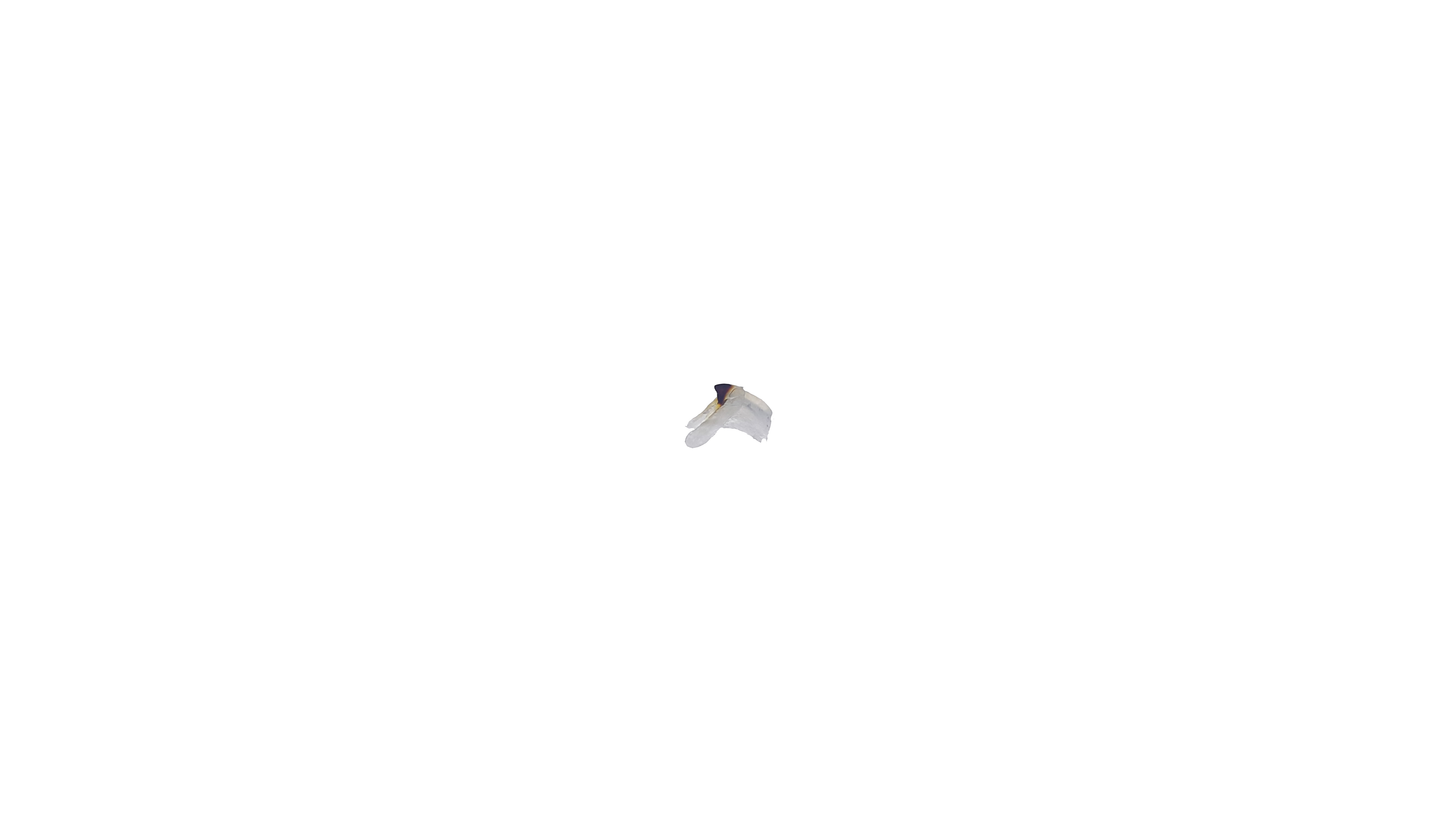

Supplement: Supplemental Information 1 — C2-Sepia aculeata, C3-Sepioteuthis lessoniana, C6-Sepia esculenta, O2-Amphioctopus aegina, S1-Loliolus uyii, S3-Uroteuthis chinensis, S4-Uroteuthis edulis [file peerj-09-11825-s001.zip › _Preprocessing_Lower_Beak/S4/L-l-S4-20.jpg]

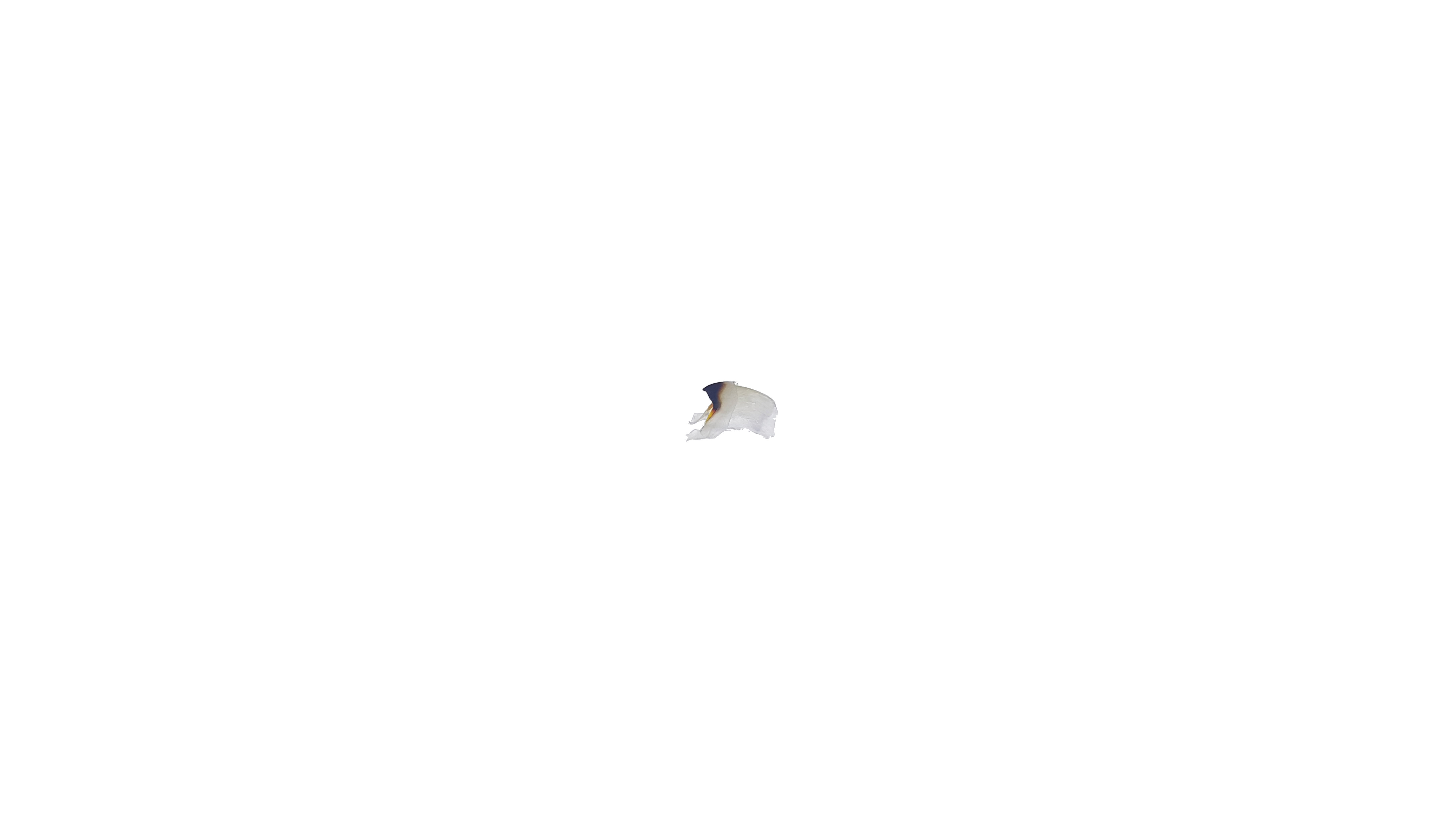

Supplement: Supplemental Information 1 — C2-Sepia aculeata, C3-Sepioteuthis lessoniana, C6-Sepia esculenta, O2-Amphioctopus aegina, S1-Loliolus uyii, S3-Uroteuthis chinensis, S4-Uroteuthis edulis [file peerj-09-11825-s001.zip › _Preprocessing_Lower_Beak/S4/L-l-S4-21.jpg]

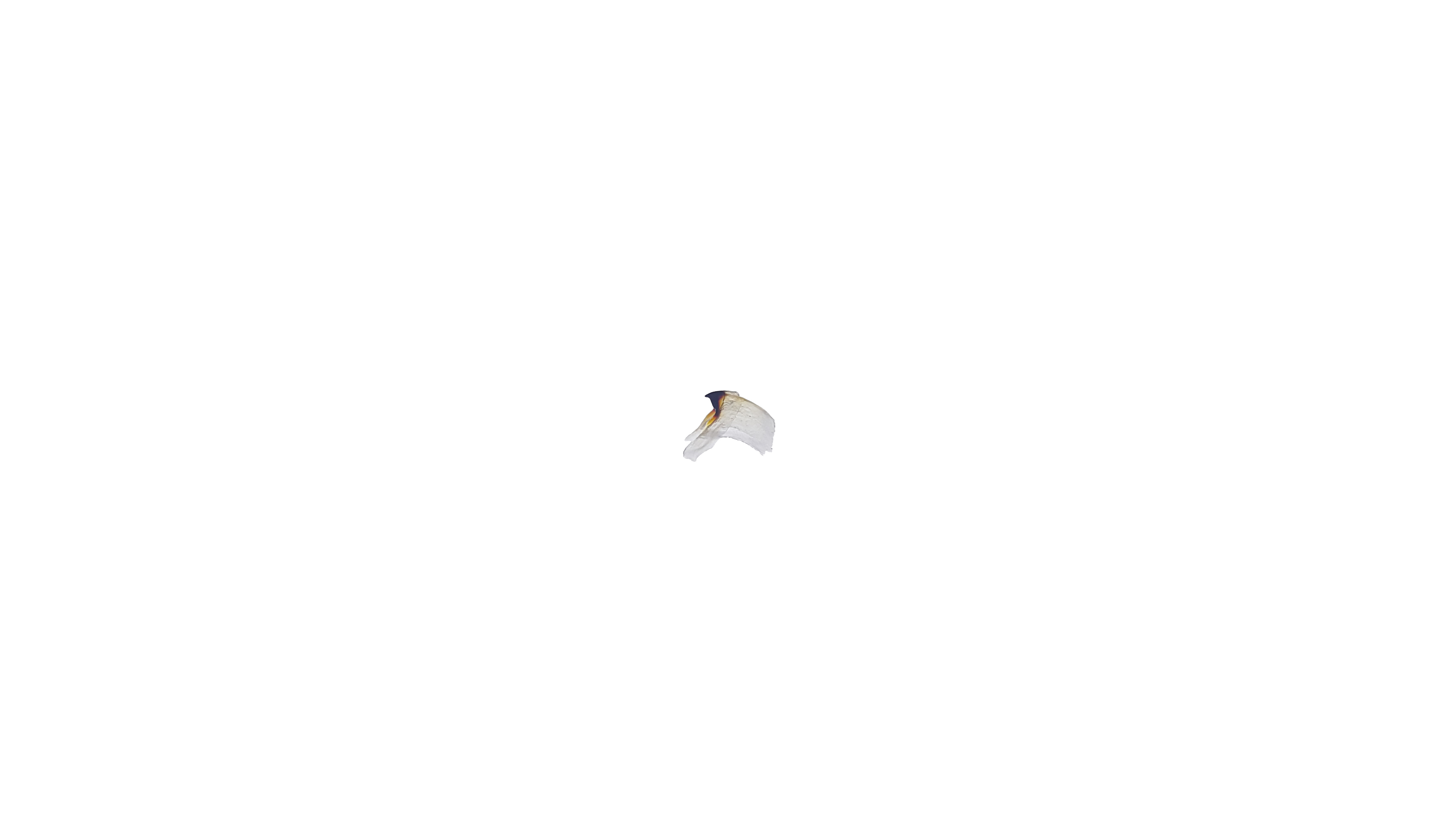

Supplement: Supplemental Information 1 — C2-Sepia aculeata, C3-Sepioteuthis lessoniana, C6-Sepia esculenta, O2-Amphioctopus aegina, S1-Loliolus uyii, S3-Uroteuthis chinensis, S4-Uroteuthis edulis [file peerj-09-11825-s001.zip › _Preprocessing_Lower_Beak/S4/L-l-S4-22.jpg]

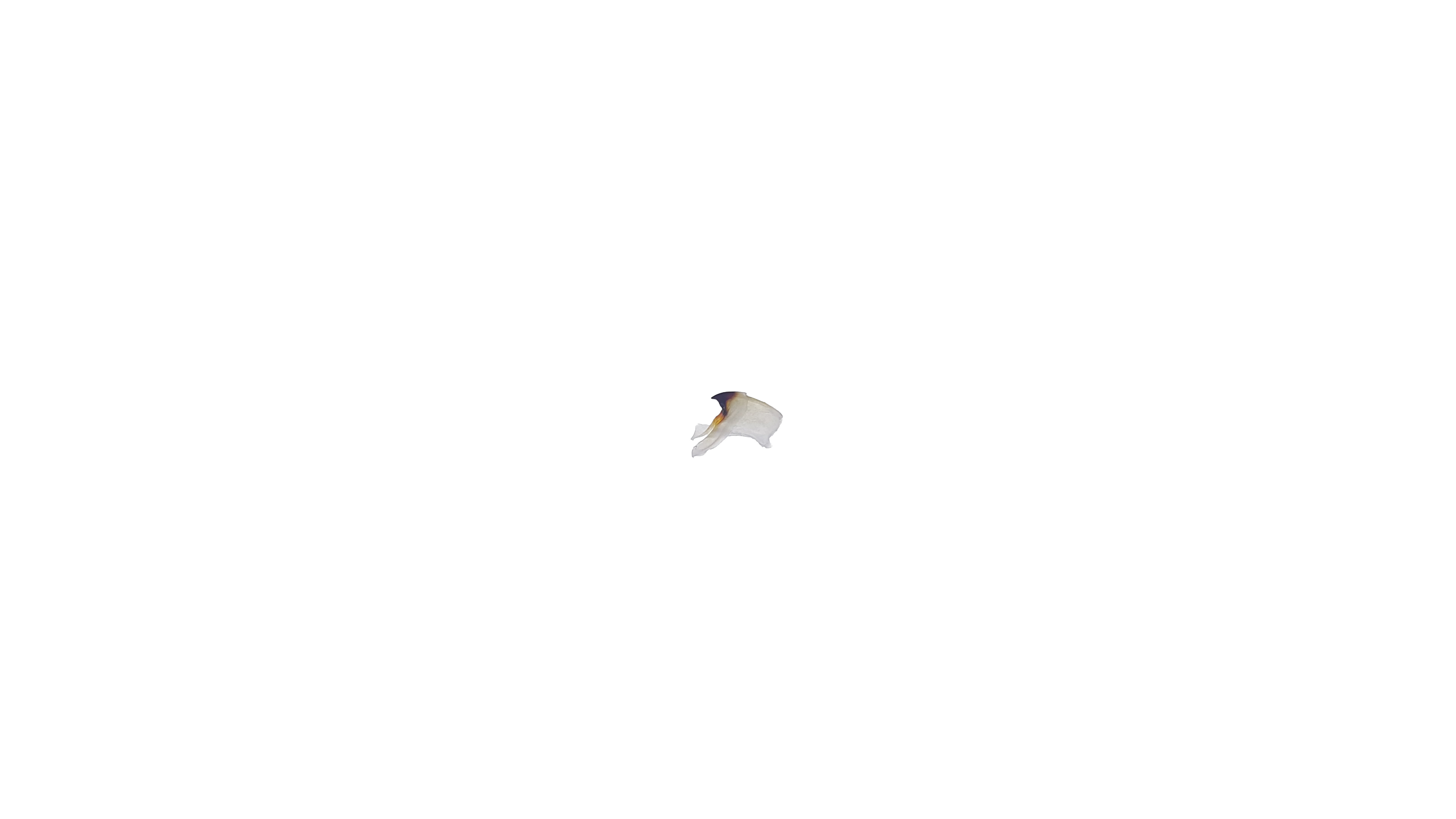

Supplement: Supplemental Information 1 — C2-Sepia aculeata, C3-Sepioteuthis lessoniana, C6-Sepia esculenta, O2-Amphioctopus aegina, S1-Loliolus uyii, S3-Uroteuthis chinensis, S4-Uroteuthis edulis [file peerj-09-11825-s001.zip › _Preprocessing_Lower_Beak/S4/L-l-S4-23.jpg]

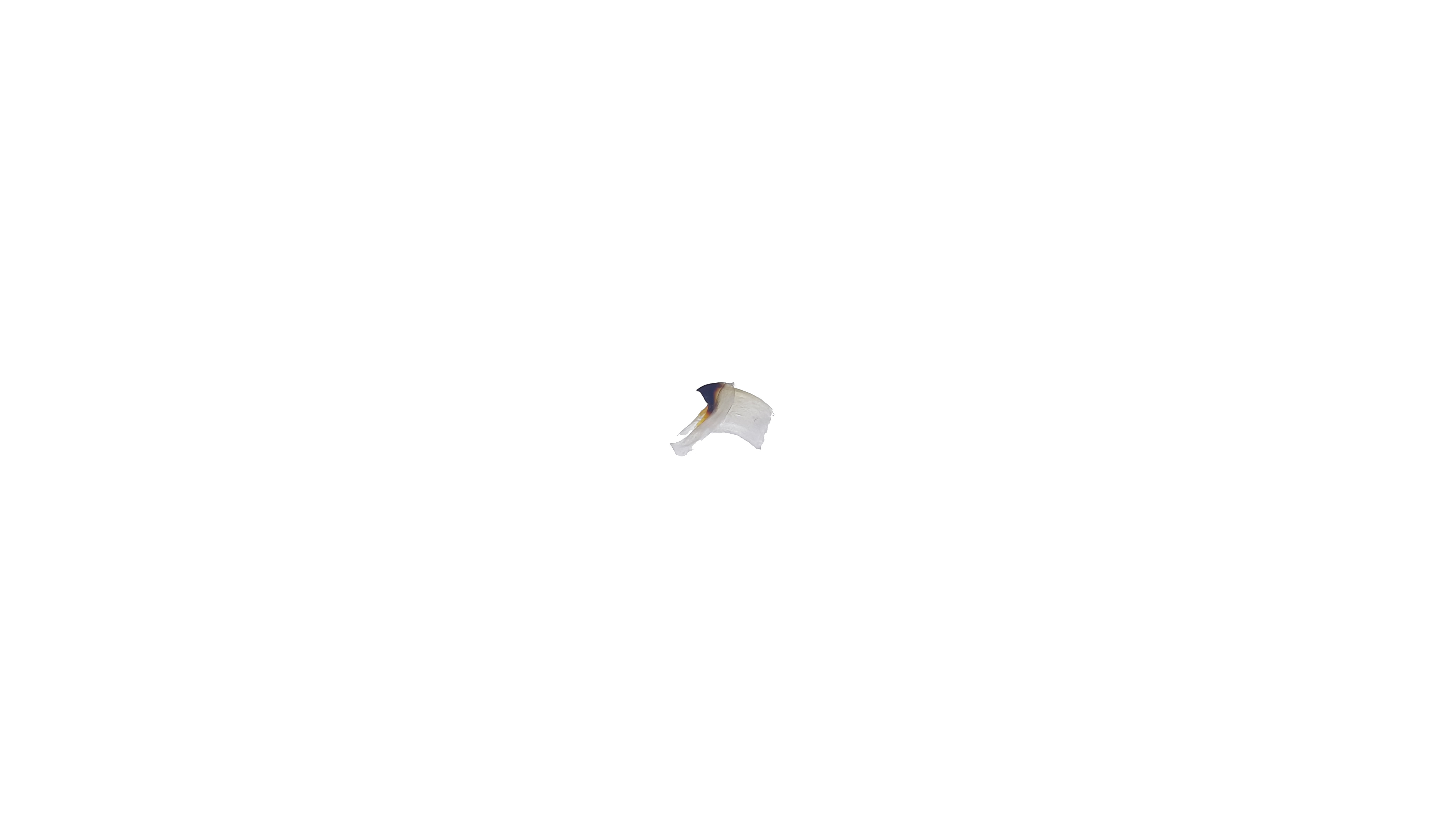

Supplement: Supplemental Information 1 — C2-Sepia aculeata, C3-Sepioteuthis lessoniana, C6-Sepia esculenta, O2-Amphioctopus aegina, S1-Loliolus uyii, S3-Uroteuthis chinensis, S4-Uroteuthis edulis [file peerj-09-11825-s001.zip › _Preprocessing_Lower_Beak/S4/L-l-S4-24.jpg]

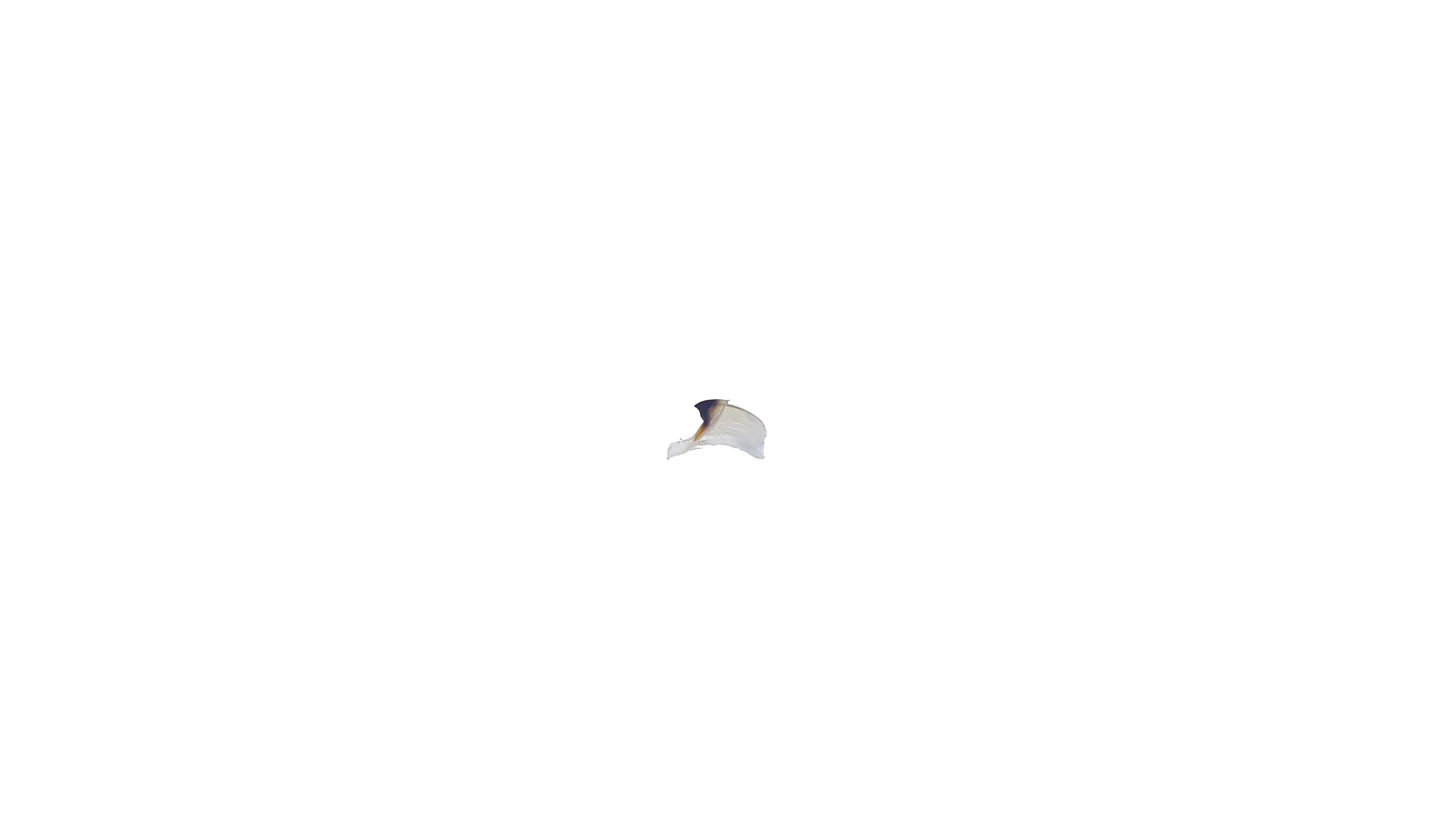

Supplement: Supplemental Information 1 — C2-Sepia aculeata, C3-Sepioteuthis lessoniana, C6-Sepia esculenta, O2-Amphioctopus aegina, S1-Loliolus uyii, S3-Uroteuthis chinensis, S4-Uroteuthis edulis [file peerj-09-11825-s001.zip › _Preprocessing_Lower_Beak/S4/L-l-S4-25.jpg]

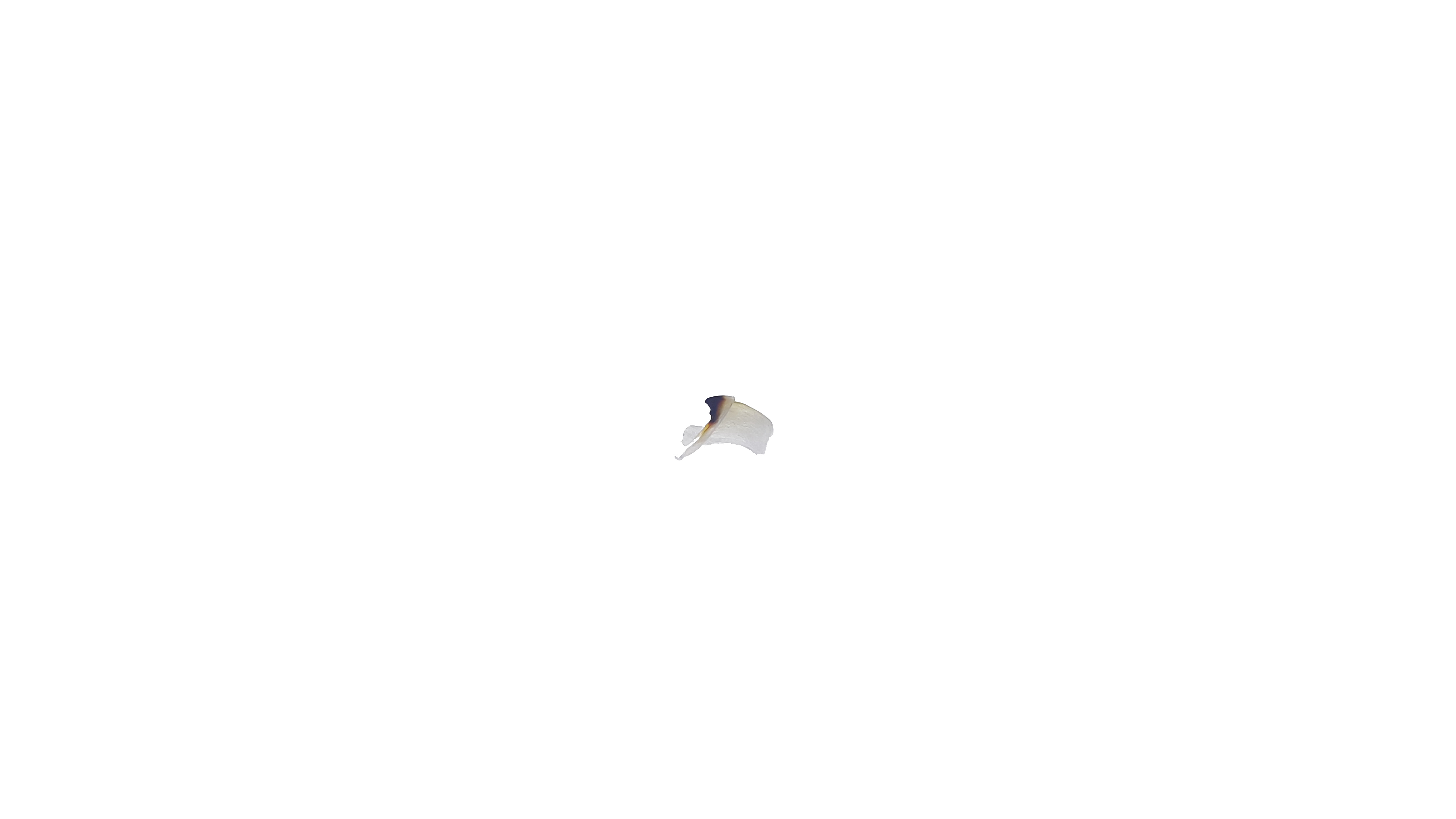

Supplement: Supplemental Information 1 — C2-Sepia aculeata, C3-Sepioteuthis lessoniana, C6-Sepia esculenta, O2-Amphioctopus aegina, S1-Loliolus uyii, S3-Uroteuthis chinensis, S4-Uroteuthis edulis [file peerj-09-11825-s001.zip › _Preprocessing_Lower_Beak/S4/L-l-S4-26.jpg]

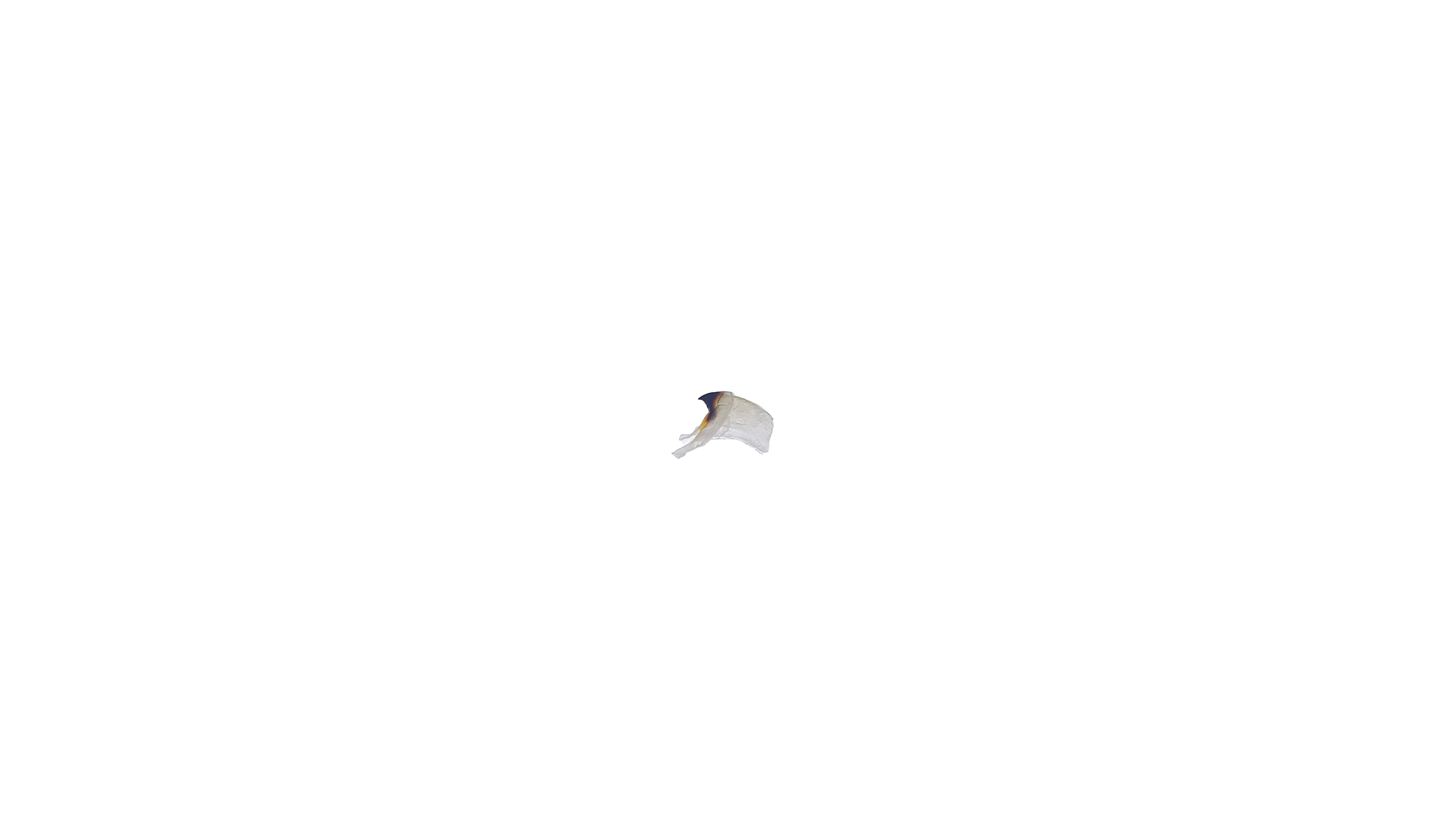

Supplement: Supplemental Information 1 — C2-Sepia aculeata, C3-Sepioteuthis lessoniana, C6-Sepia esculenta, O2-Amphioctopus aegina, S1-Loliolus uyii, S3-Uroteuthis chinensis, S4-Uroteuthis edulis [file peerj-09-11825-s001.zip › _Preprocessing_Lower_Beak/S4/L-l-S4-27.jpg]

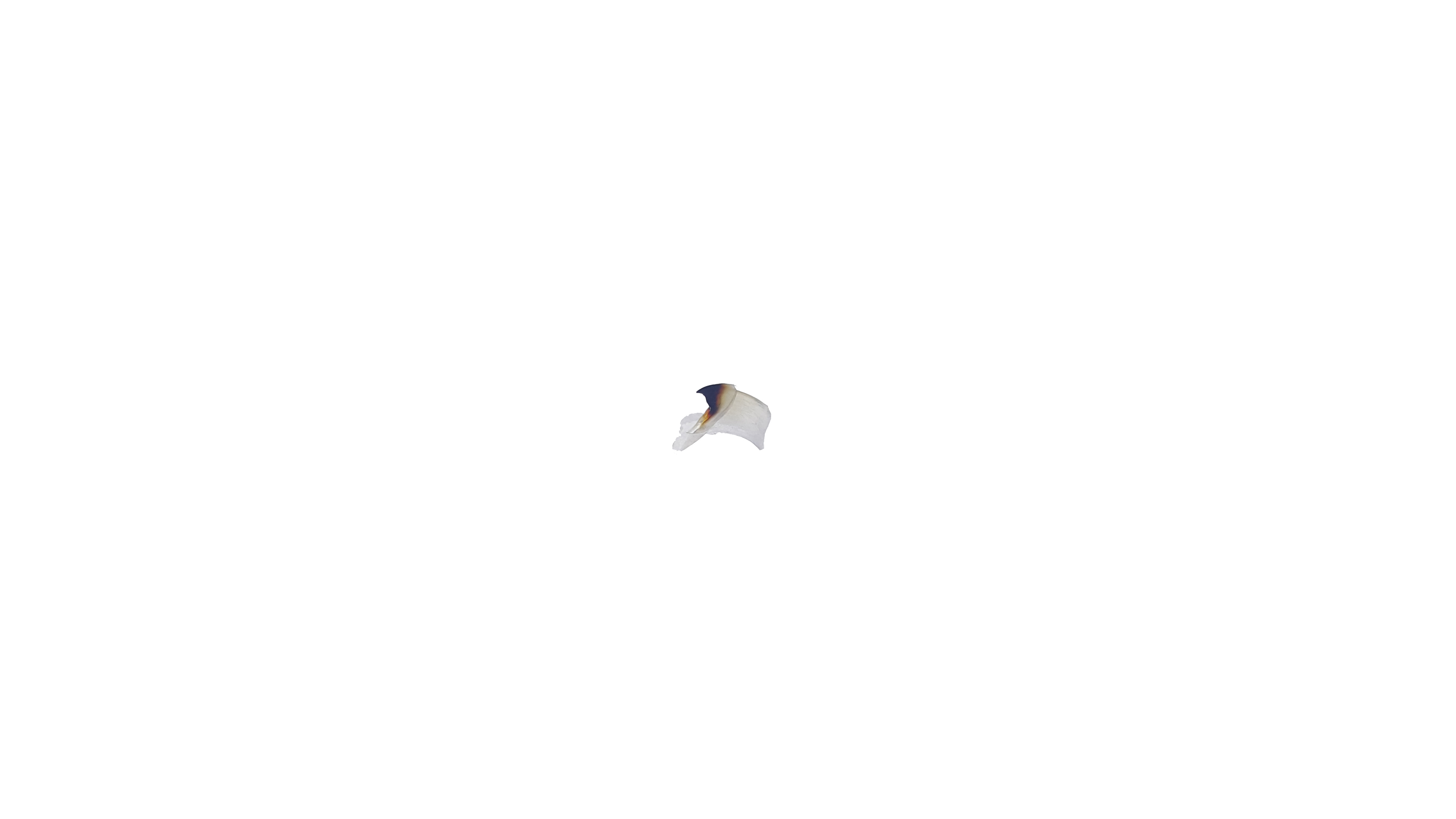

Supplement: Supplemental Information 1 — C2-Sepia aculeata, C3-Sepioteuthis lessoniana, C6-Sepia esculenta, O2-Amphioctopus aegina, S1-Loliolus uyii, S3-Uroteuthis chinensis, S4-Uroteuthis edulis [file peerj-09-11825-s001.zip › _Preprocessing_Lower_Beak/S4/L-l-S4-28.jpg]

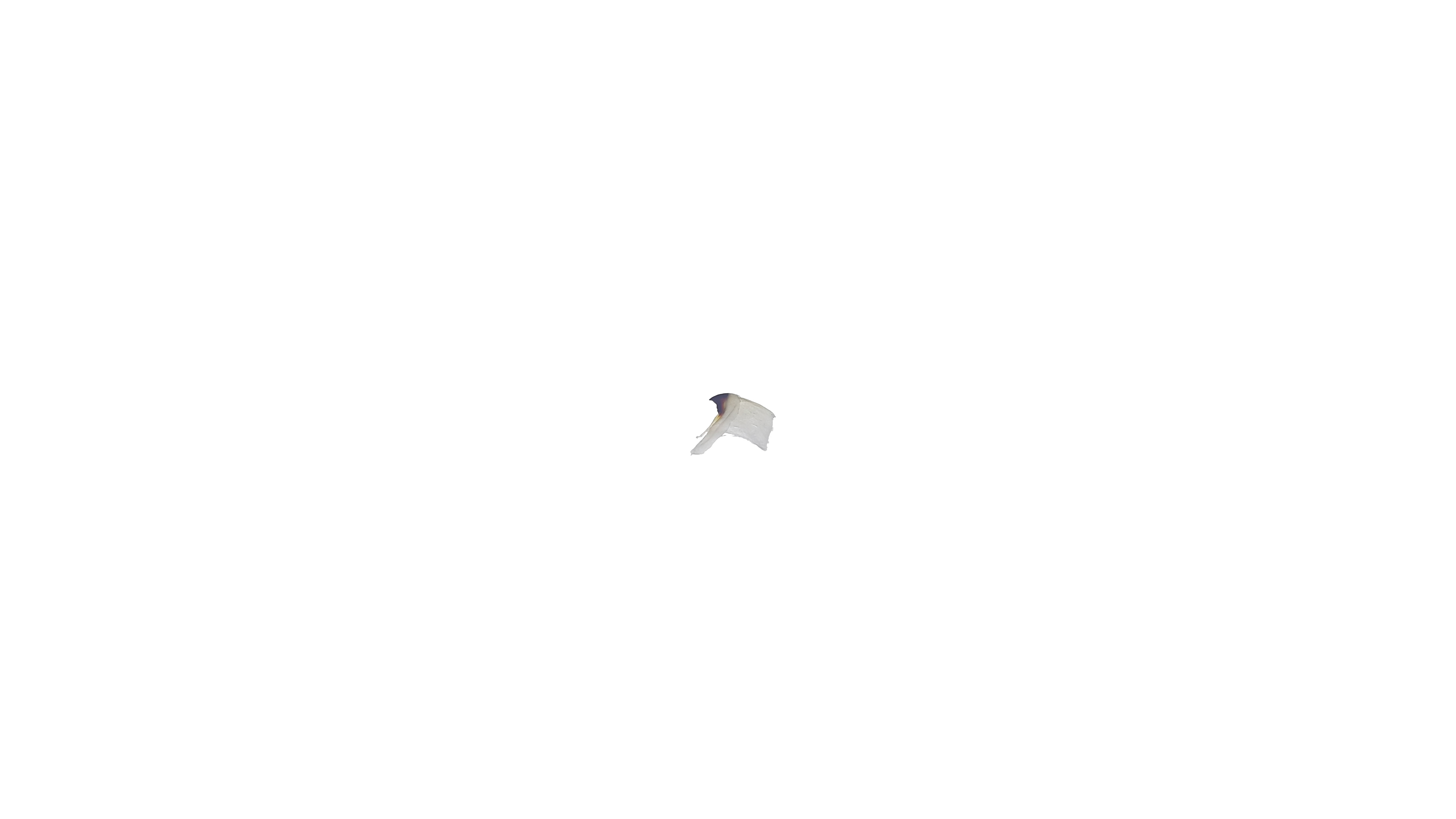

Supplement: Supplemental Information 1 — C2-Sepia aculeata, C3-Sepioteuthis lessoniana, C6-Sepia esculenta, O2-Amphioctopus aegina, S1-Loliolus uyii, S3-Uroteuthis chinensis, S4-Uroteuthis edulis [file peerj-09-11825-s001.zip › _Preprocessing_Lower_Beak/S4/L-l-S4-3.jpg]

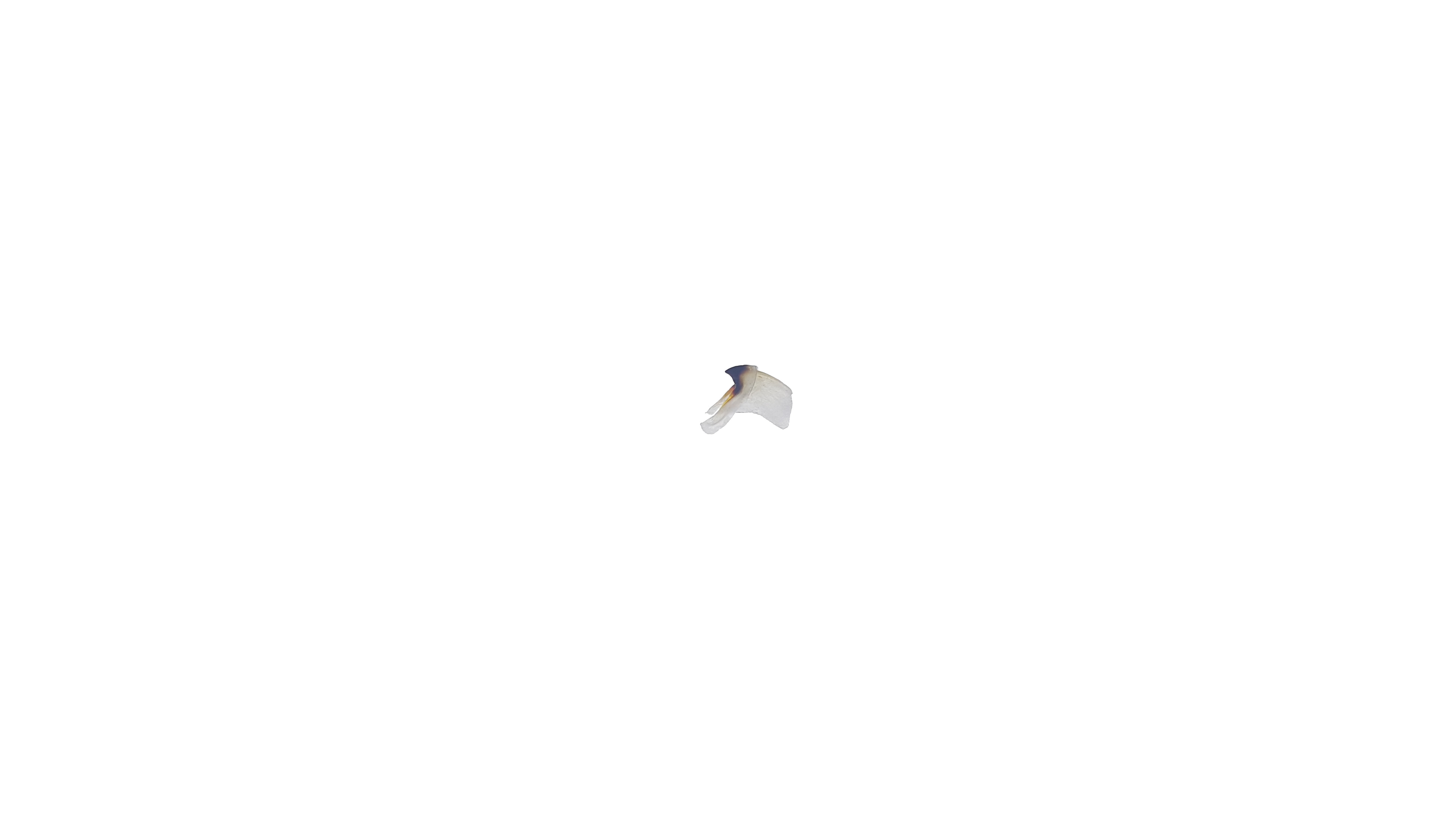

Supplement: Supplemental Information 1 — C2-Sepia aculeata, C3-Sepioteuthis lessoniana, C6-Sepia esculenta, O2-Amphioctopus aegina, S1-Loliolus uyii, S3-Uroteuthis chinensis, S4-Uroteuthis edulis [file peerj-09-11825-s001.zip › _Preprocessing_Lower_Beak/S4/L-l-S4-4.jpg]

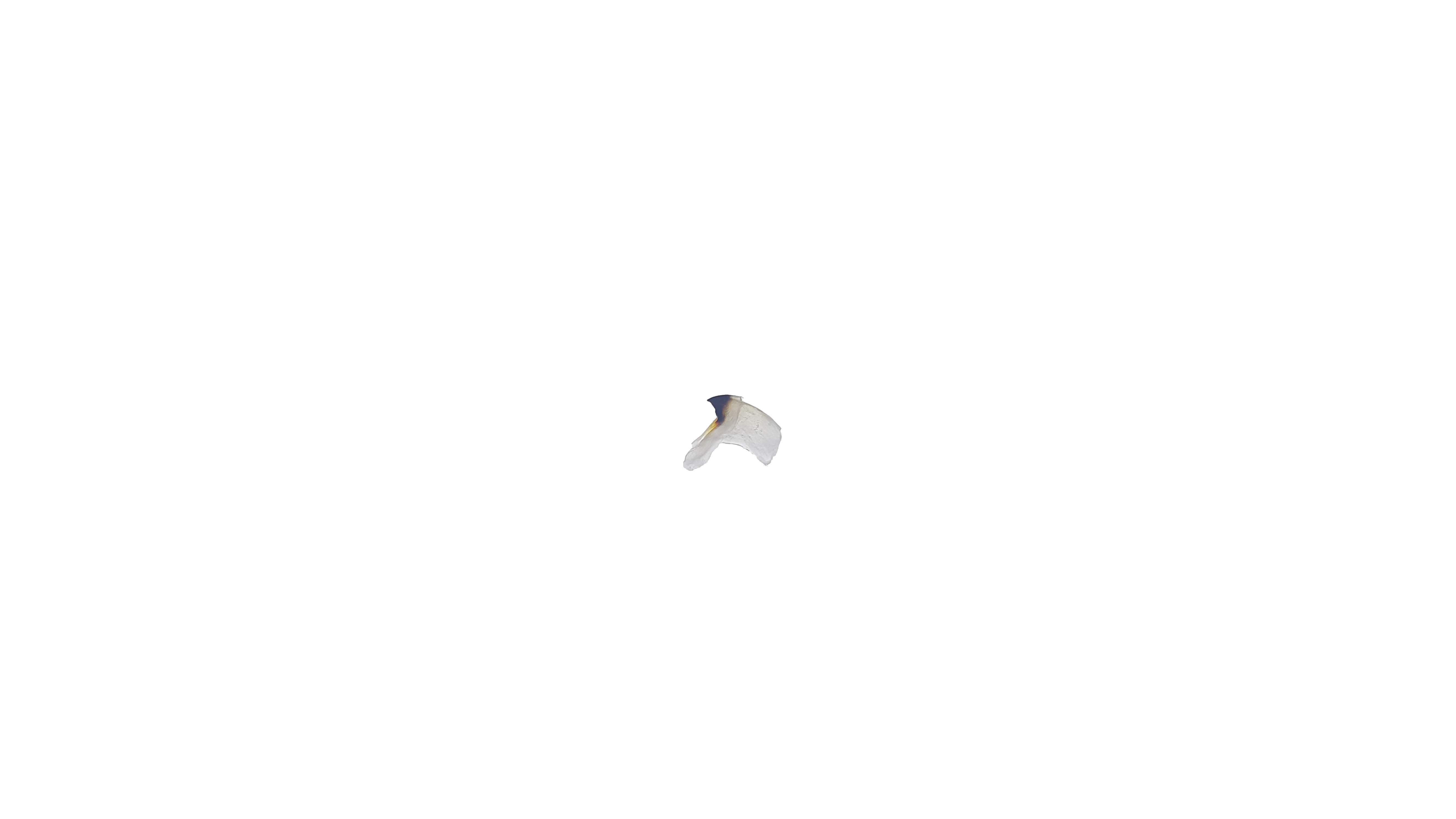

Supplement: Supplemental Information 1 — C2-Sepia aculeata, C3-Sepioteuthis lessoniana, C6-Sepia esculenta, O2-Amphioctopus aegina, S1-Loliolus uyii, S3-Uroteuthis chinensis, S4-Uroteuthis edulis [file peerj-09-11825-s001.zip › _Preprocessing_Lower_Beak/S4/L-l-S4-5.jpg]

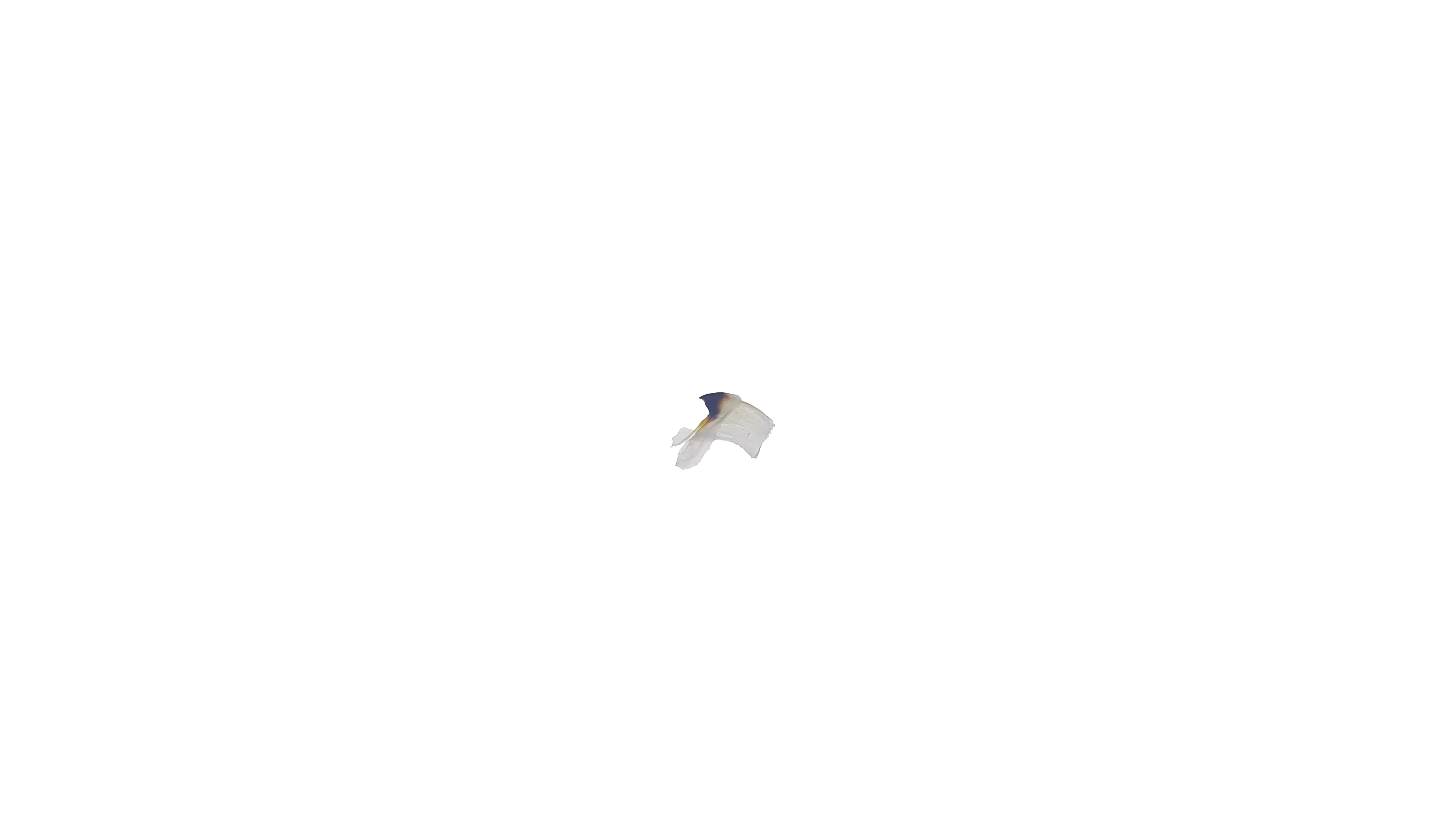

Supplement: Supplemental Information 1 — C2-Sepia aculeata, C3-Sepioteuthis lessoniana, C6-Sepia esculenta, O2-Amphioctopus aegina, S1-Loliolus uyii, S3-Uroteuthis chinensis, S4-Uroteuthis edulis [file peerj-09-11825-s001.zip › _Preprocessing_Lower_Beak/S4/L-l-S4-6.jpg]

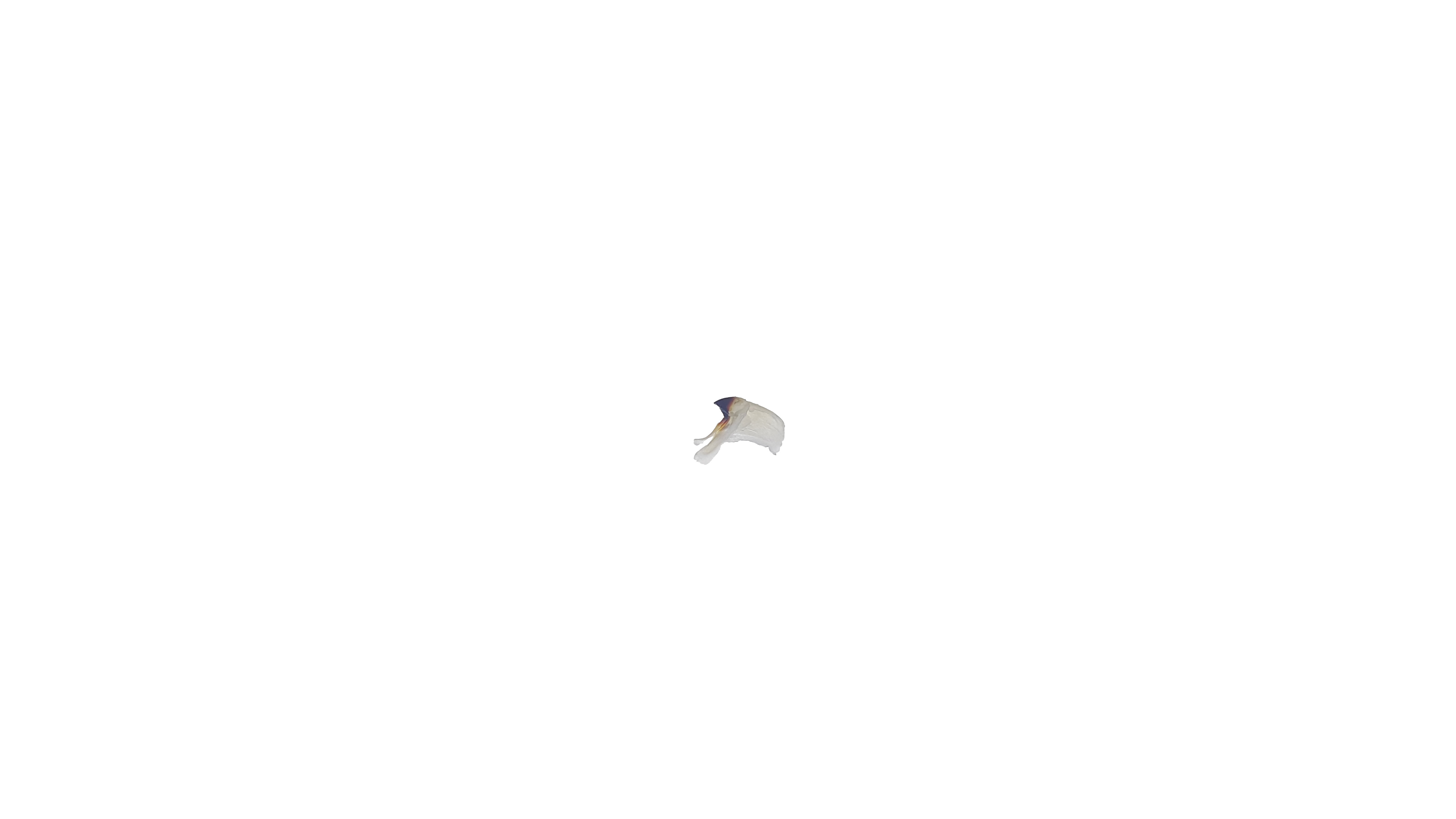

Supplement: Supplemental Information 1 — C2-Sepia aculeata, C3-Sepioteuthis lessoniana, C6-Sepia esculenta, O2-Amphioctopus aegina, S1-Loliolus uyii, S3-Uroteuthis chinensis, S4-Uroteuthis edulis [file peerj-09-11825-s001.zip › _Preprocessing_Lower_Beak/S4/L-l-S4-7.jpg]

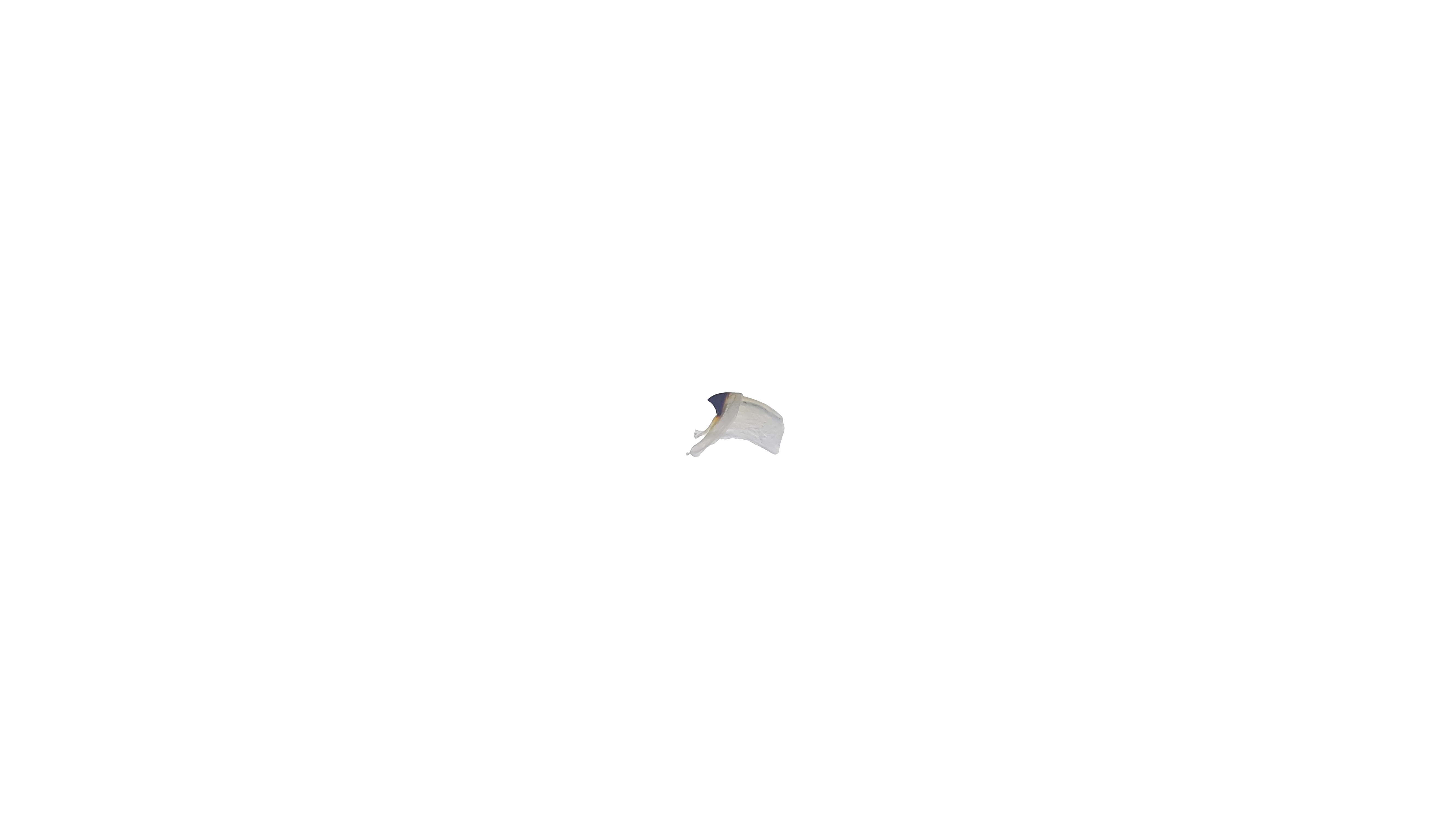

Supplement: Supplemental Information 1 — C2-Sepia aculeata, C3-Sepioteuthis lessoniana, C6-Sepia esculenta, O2-Amphioctopus aegina, S1-Loliolus uyii, S3-Uroteuthis chinensis, S4-Uroteuthis edulis [file peerj-09-11825-s001.zip › _Preprocessing_Lower_Beak/S4/L-l-S4-8.jpg]

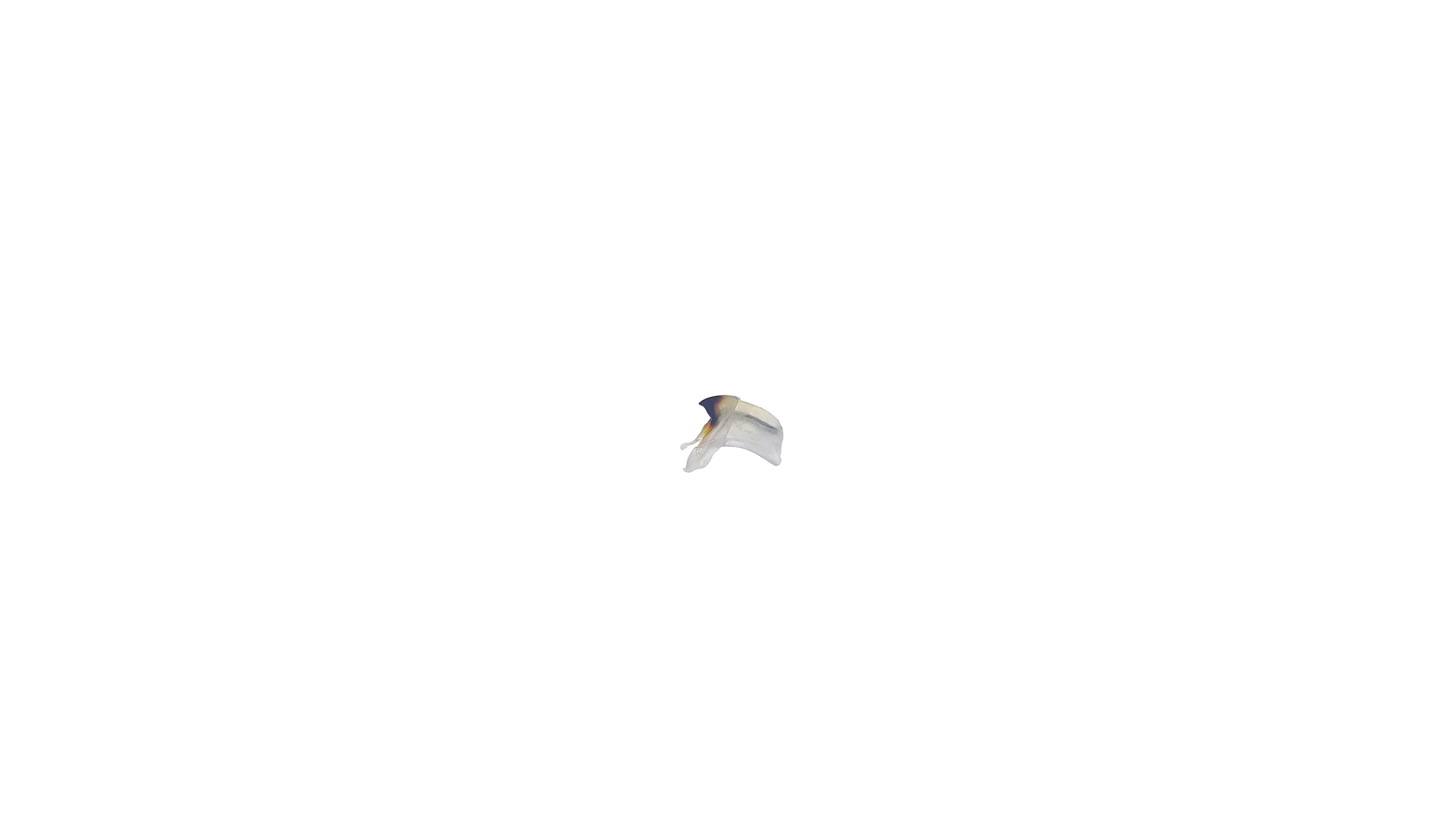

Supplement: Supplemental Information 1 — C2-Sepia aculeata, C3-Sepioteuthis lessoniana, C6-Sepia esculenta, O2-Amphioctopus aegina, S1-Loliolus uyii, S3-Uroteuthis chinensis, S4-Uroteuthis edulis [file peerj-09-11825-s001.zip › _Preprocessing_Lower_Beak/S4/L-l-S4-9.jpg]

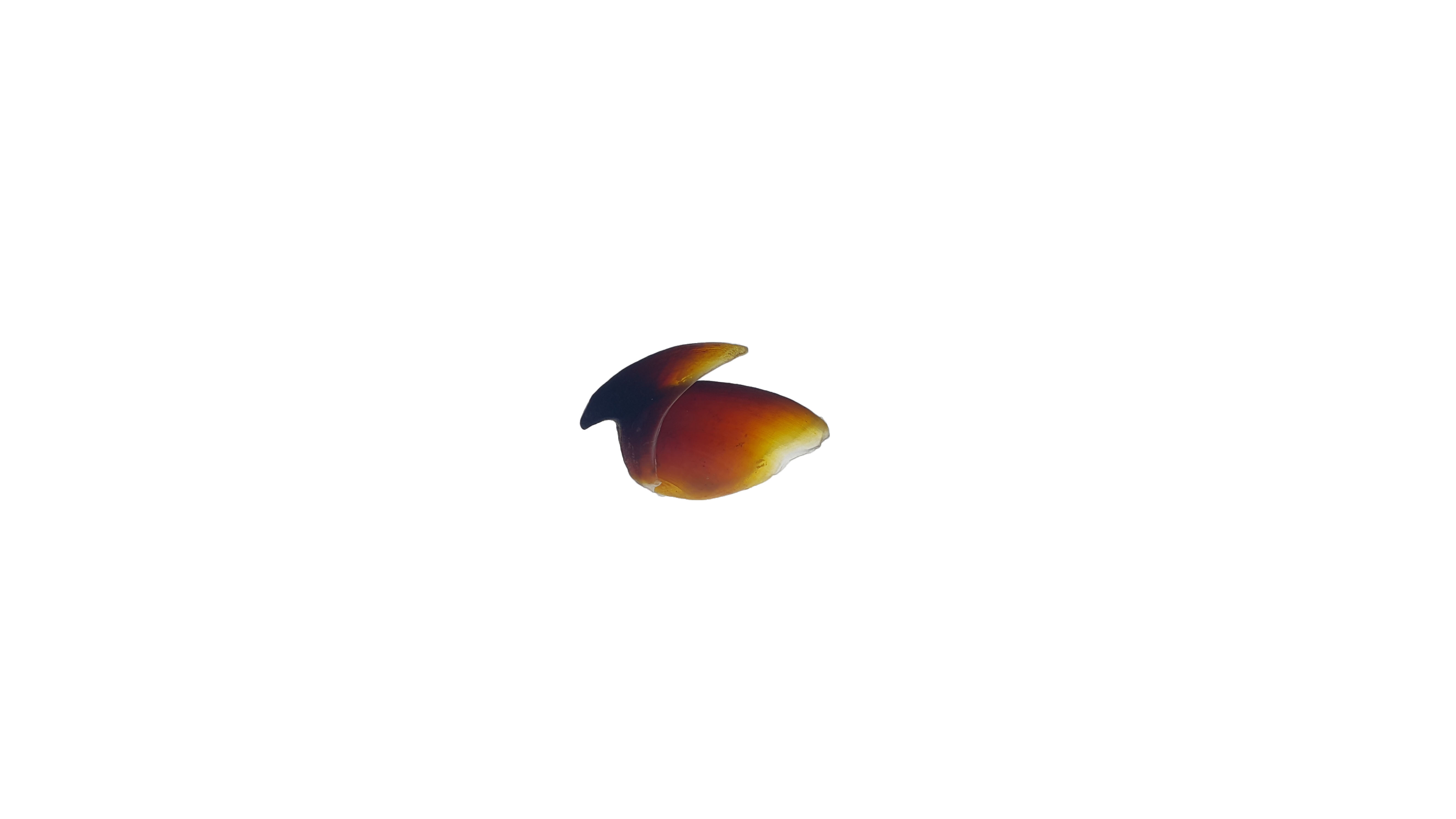

Supplement: Supplemental Information 2 — C2-Sepia aculeata, C3-Sepioteuthis lessoniana, C6-Sepia esculenta, O2-Amphioctopus aegina, S1-Loliolus uyii, S3-Uroteuthis chinensis, S4-Uroteuthis edulis [file peerj-09-11825-s002.zip › _Preprocessing_Upper_Beak/C2/U-l-C2-1.jpg]

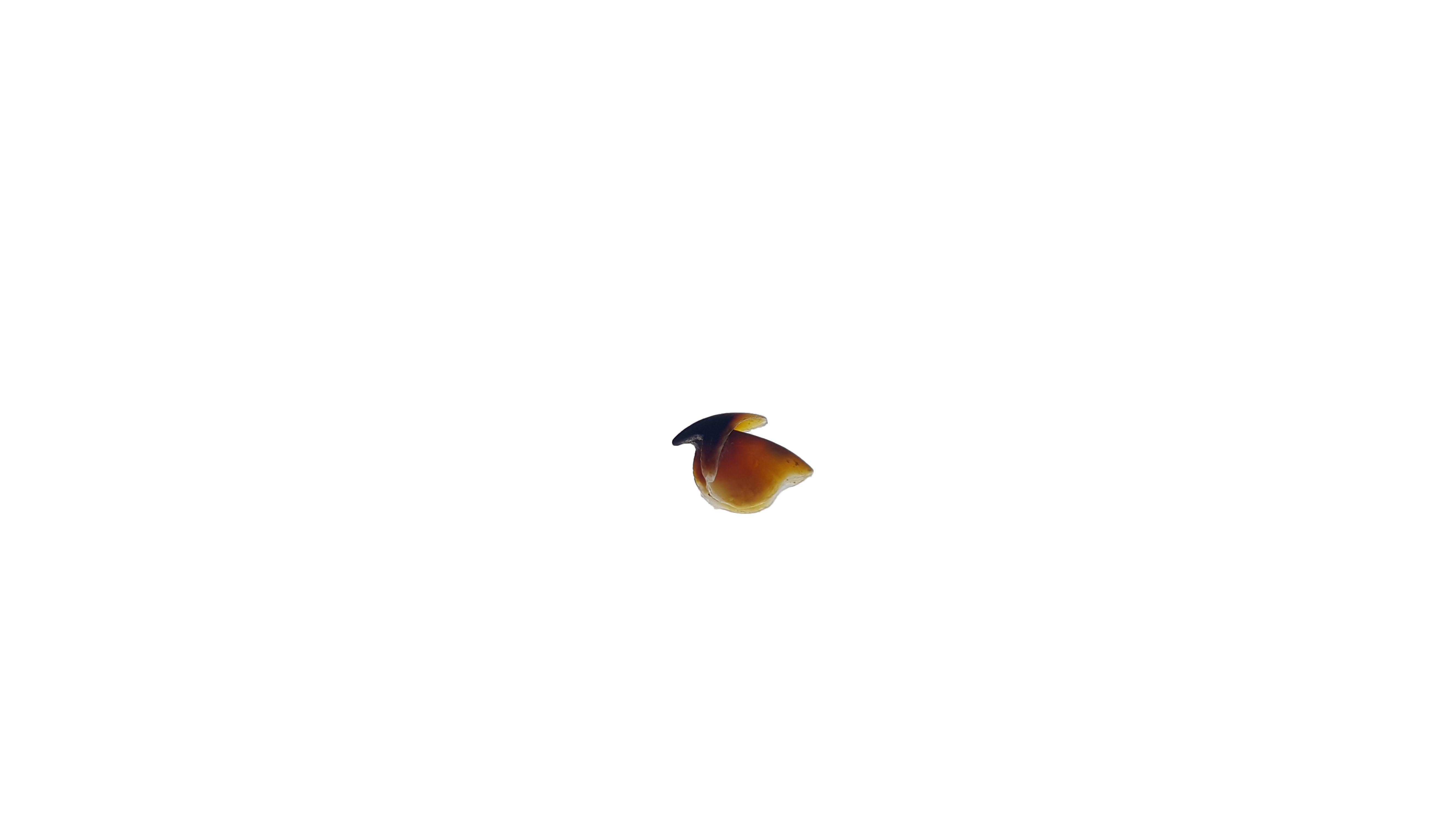

Supplement: Supplemental Information 2 — C2-Sepia aculeata, C3-Sepioteuthis lessoniana, C6-Sepia esculenta, O2-Amphioctopus aegina, S1-Loliolus uyii, S3-Uroteuthis chinensis, S4-Uroteuthis edulis [file peerj-09-11825-s002.zip › _Preprocessing_Upper_Beak/C2/U-l-C2-10.jpg]

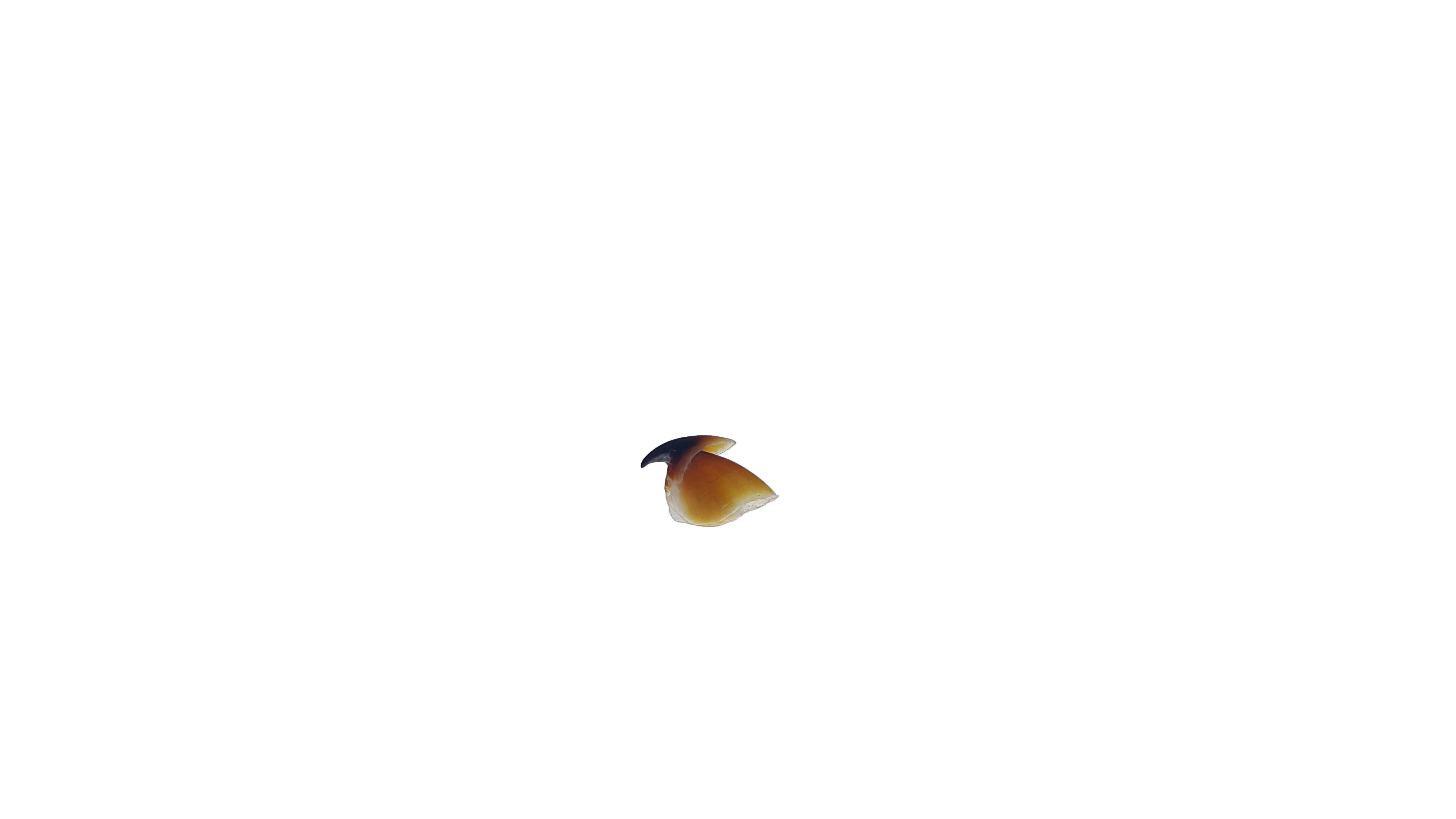

Supplement: Supplemental Information 2 — C2-Sepia aculeata, C3-Sepioteuthis lessoniana, C6-Sepia esculenta, O2-Amphioctopus aegina, S1-Loliolus uyii, S3-Uroteuthis chinensis, S4-Uroteuthis edulis [file peerj-09-11825-s002.zip › _Preprocessing_Upper_Beak/C2/U-l-C2-11.jpg]

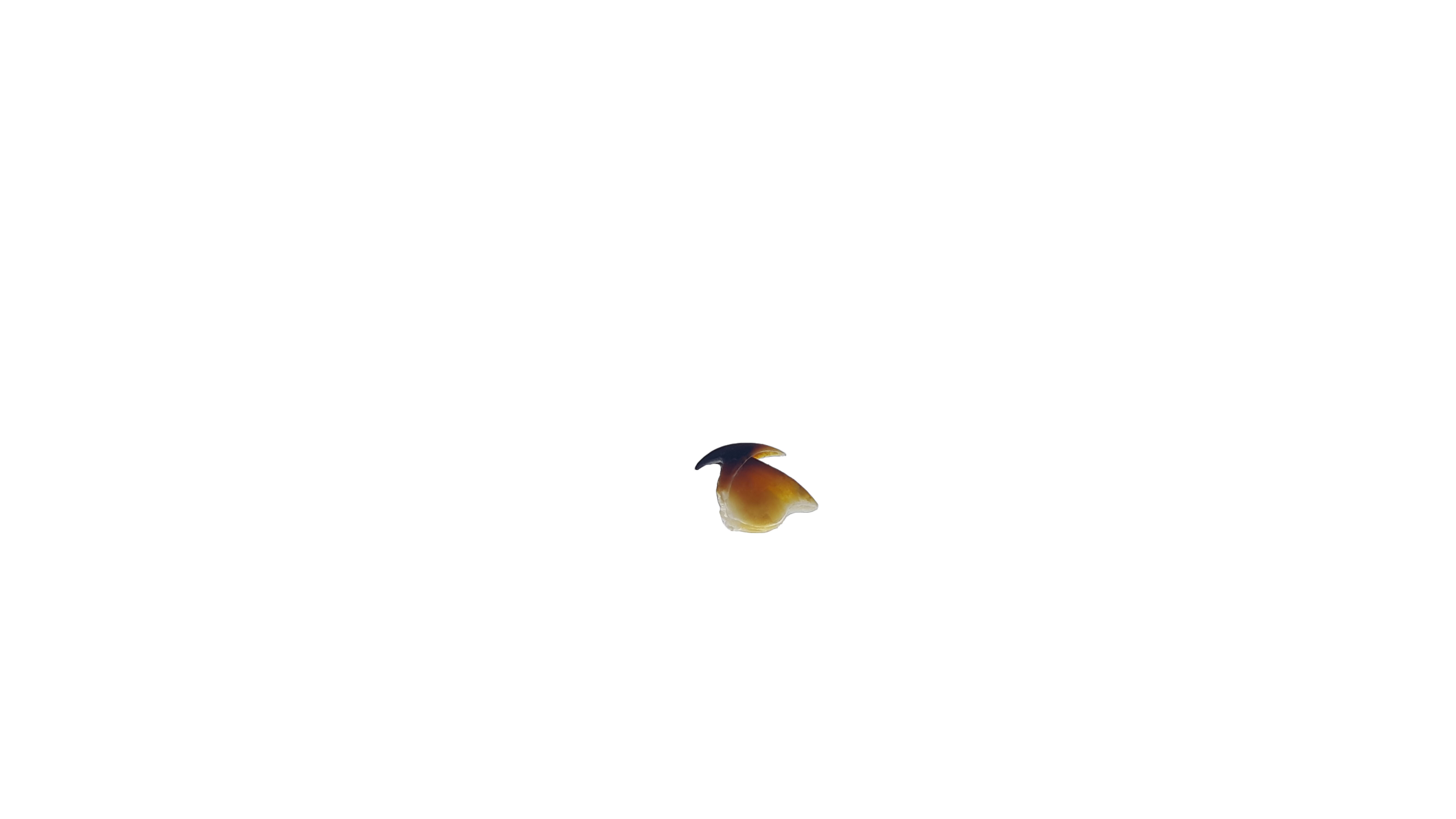

Supplement: Supplemental Information 2 — C2-Sepia aculeata, C3-Sepioteuthis lessoniana, C6-Sepia esculenta, O2-Amphioctopus aegina, S1-Loliolus uyii, S3-Uroteuthis chinensis, S4-Uroteuthis edulis [file peerj-09-11825-s002.zip › _Preprocessing_Upper_Beak/C2/U-l-C2-12.jpg]

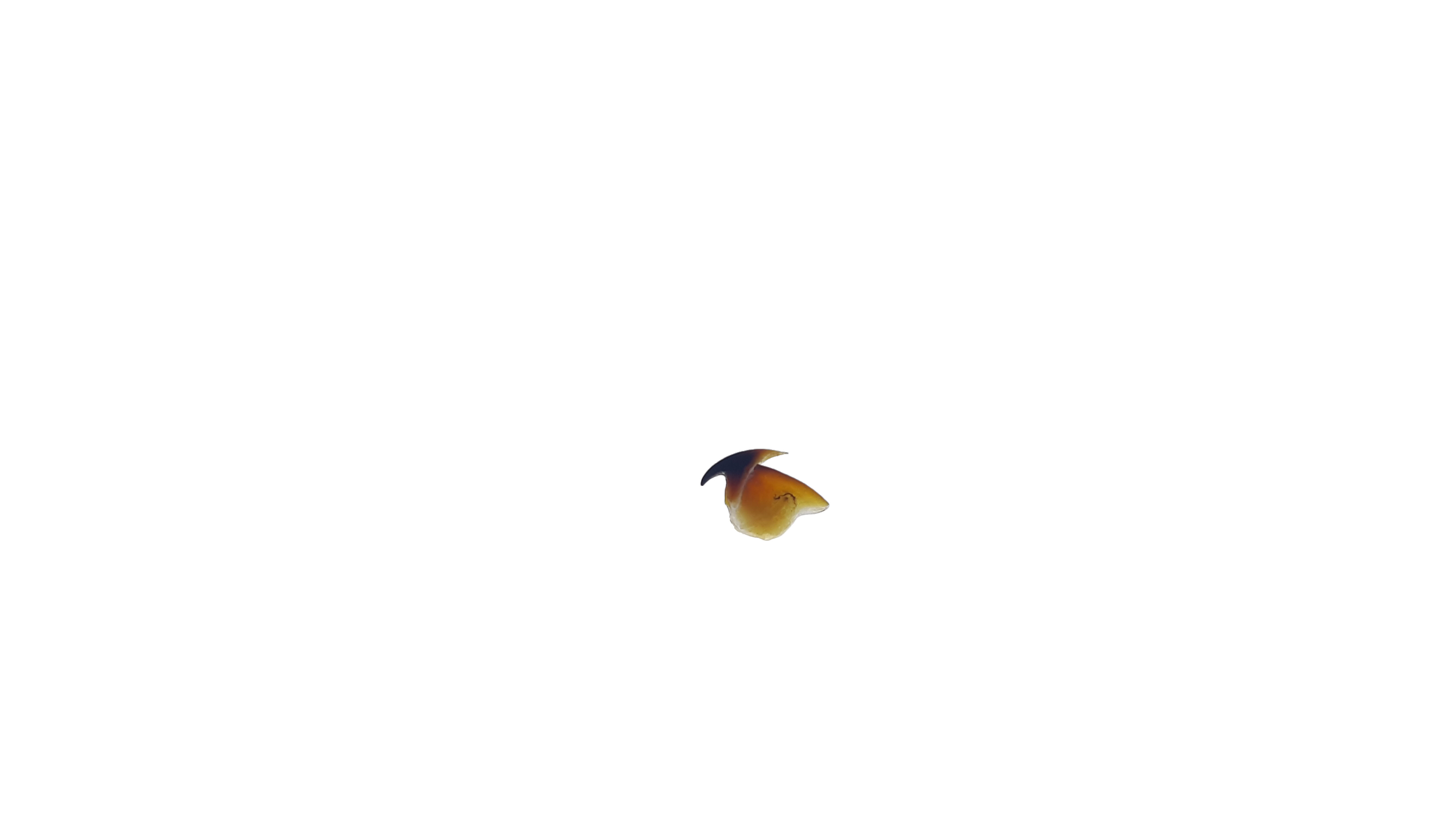

Supplement: Supplemental Information 2 — C2-Sepia aculeata, C3-Sepioteuthis lessoniana, C6-Sepia esculenta, O2-Amphioctopus aegina, S1-Loliolus uyii, S3-Uroteuthis chinensis, S4-Uroteuthis edulis [file peerj-09-11825-s002.zip › _Preprocessing_Upper_Beak/C2/U-l-C2-13.jpg]

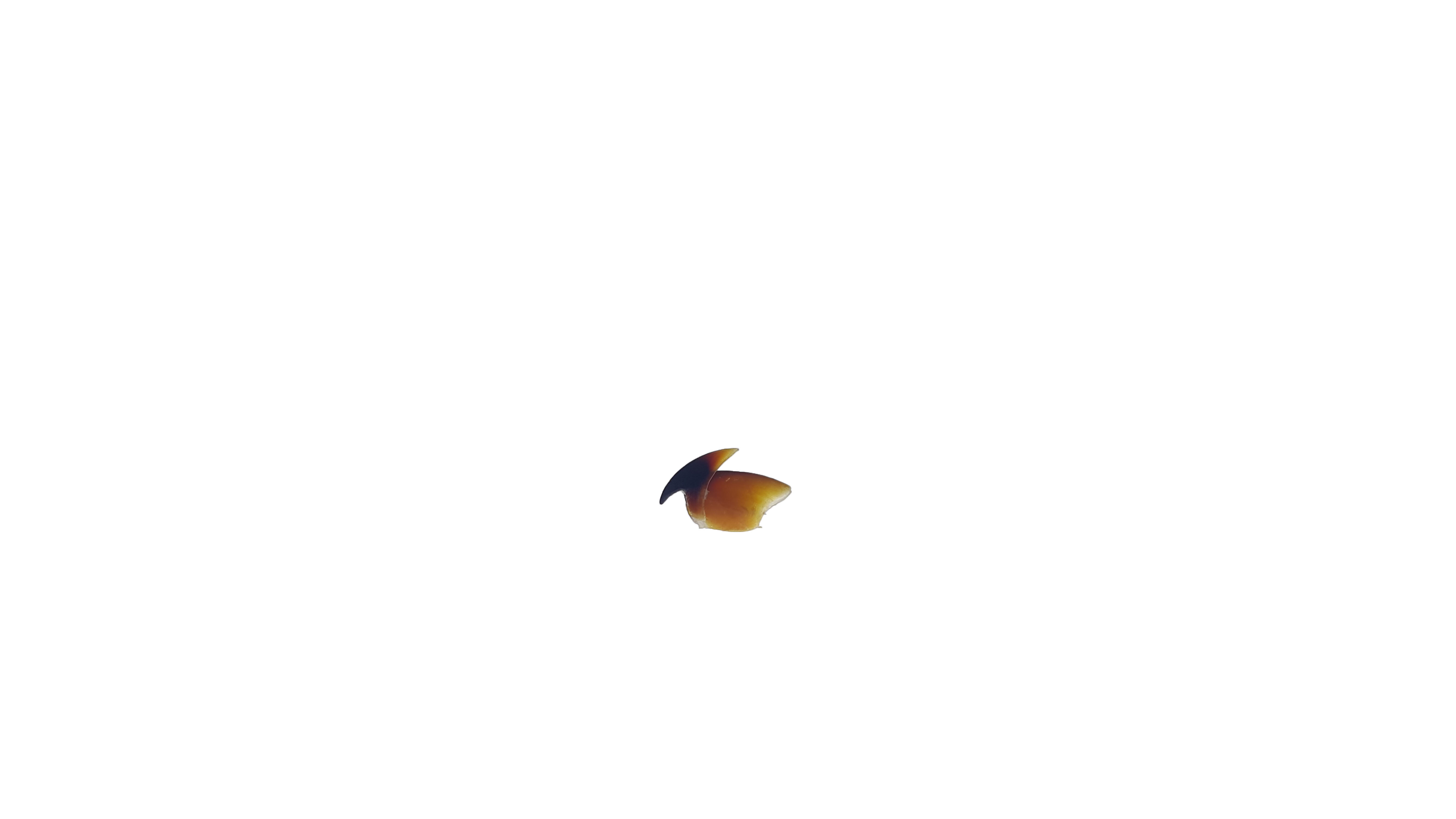

Supplement: Supplemental Information 2 — C2-Sepia aculeata, C3-Sepioteuthis lessoniana, C6-Sepia esculenta, O2-Amphioctopus aegina, S1-Loliolus uyii, S3-Uroteuthis chinensis, S4-Uroteuthis edulis [file peerj-09-11825-s002.zip › _Preprocessing_Upper_Beak/C2/U-l-C2-14.jpg]

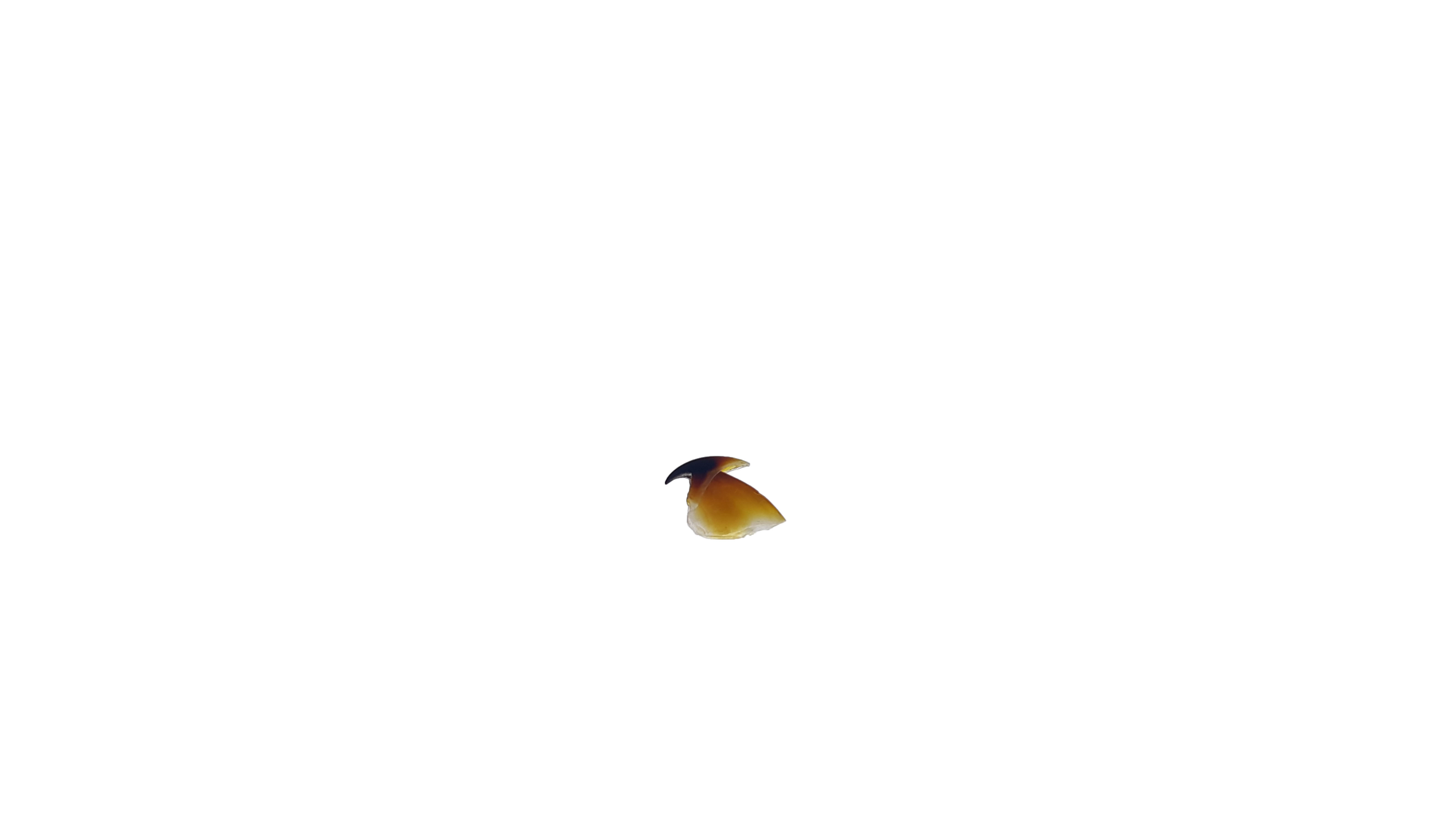

Supplement: Supplemental Information 2 — C2-Sepia aculeata, C3-Sepioteuthis lessoniana, C6-Sepia esculenta, O2-Amphioctopus aegina, S1-Loliolus uyii, S3-Uroteuthis chinensis, S4-Uroteuthis edulis [file peerj-09-11825-s002.zip › _Preprocessing_Upper_Beak/C2/U-l-C2-15.jpg]

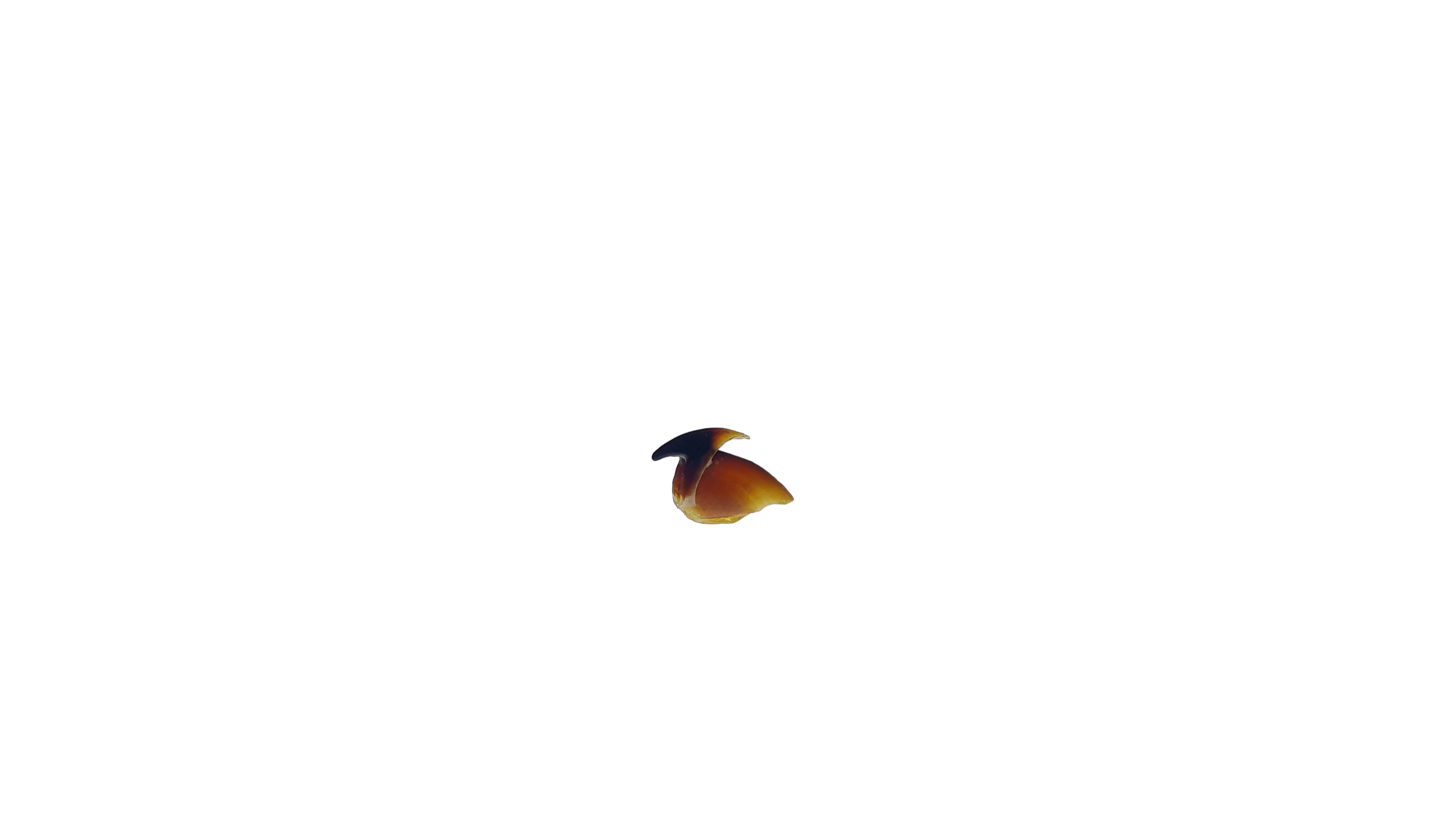

Supplement: Supplemental Information 2 — C2-Sepia aculeata, C3-Sepioteuthis lessoniana, C6-Sepia esculenta, O2-Amphioctopus aegina, S1-Loliolus uyii, S3-Uroteuthis chinensis, S4-Uroteuthis edulis [file peerj-09-11825-s002.zip › _Preprocessing_Upper_Beak/C2/U-l-C2-16.jpg]

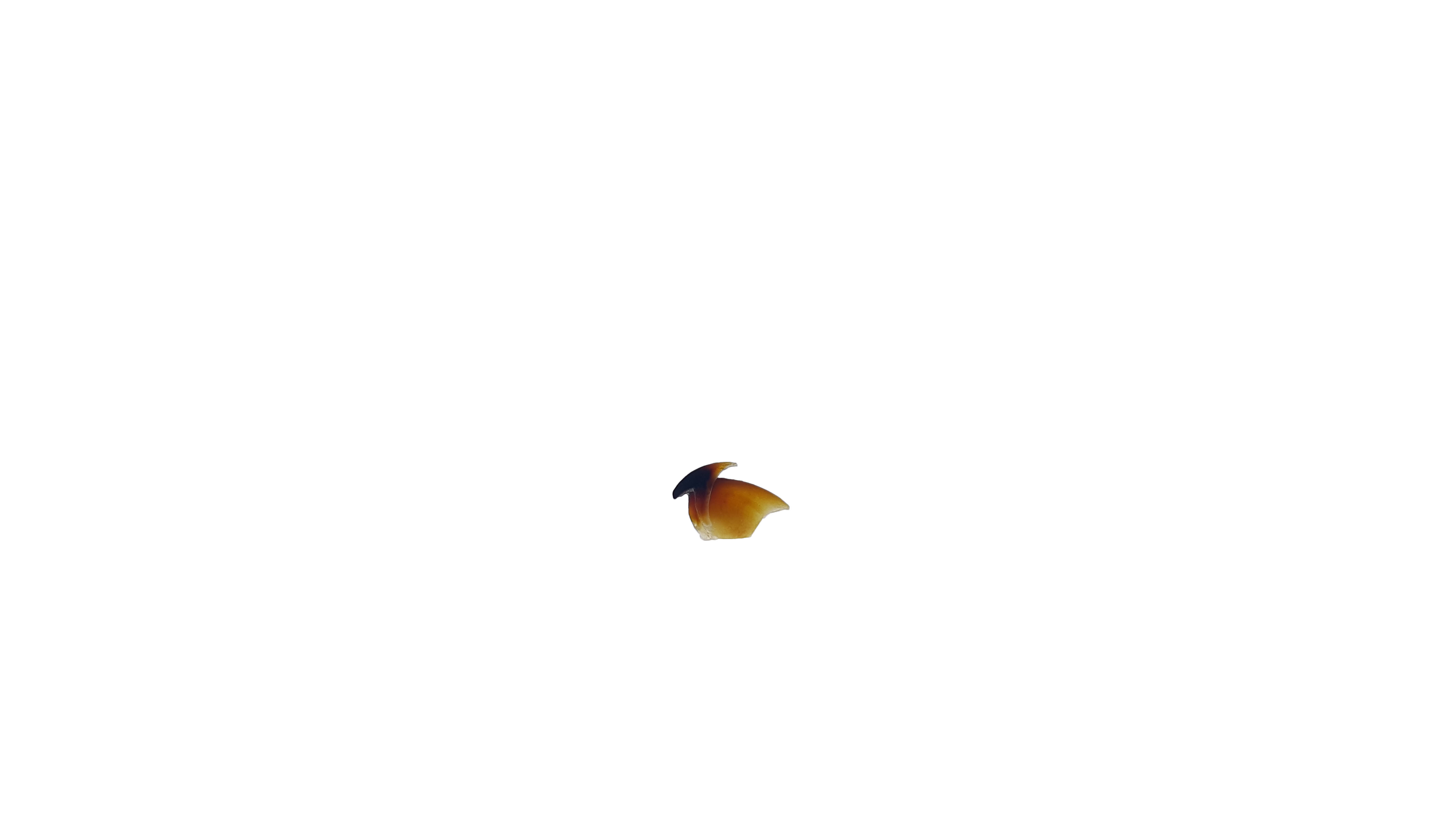

Supplement: Supplemental Information 2 — C2-Sepia aculeata, C3-Sepioteuthis lessoniana, C6-Sepia esculenta, O2-Amphioctopus aegina, S1-Loliolus uyii, S3-Uroteuthis chinensis, S4-Uroteuthis edulis [file peerj-09-11825-s002.zip › _Preprocessing_Upper_Beak/C2/U-l-C2-17.jpg]

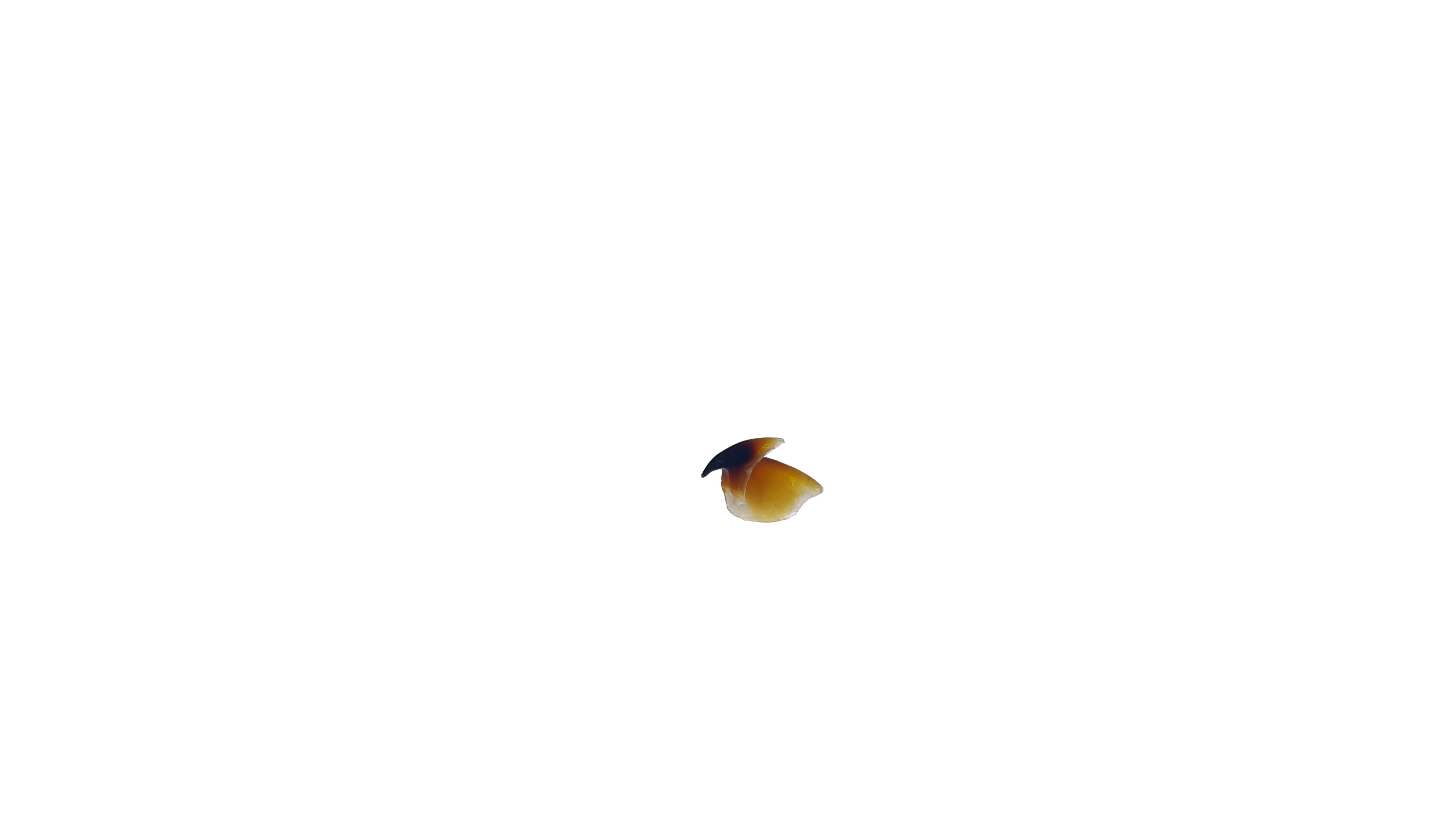

Supplement: Supplemental Information 2 — C2-Sepia aculeata, C3-Sepioteuthis lessoniana, C6-Sepia esculenta, O2-Amphioctopus aegina, S1-Loliolus uyii, S3-Uroteuthis chinensis, S4-Uroteuthis edulis [file peerj-09-11825-s002.zip › _Preprocessing_Upper_Beak/C2/U-l-C2-18.jpg]

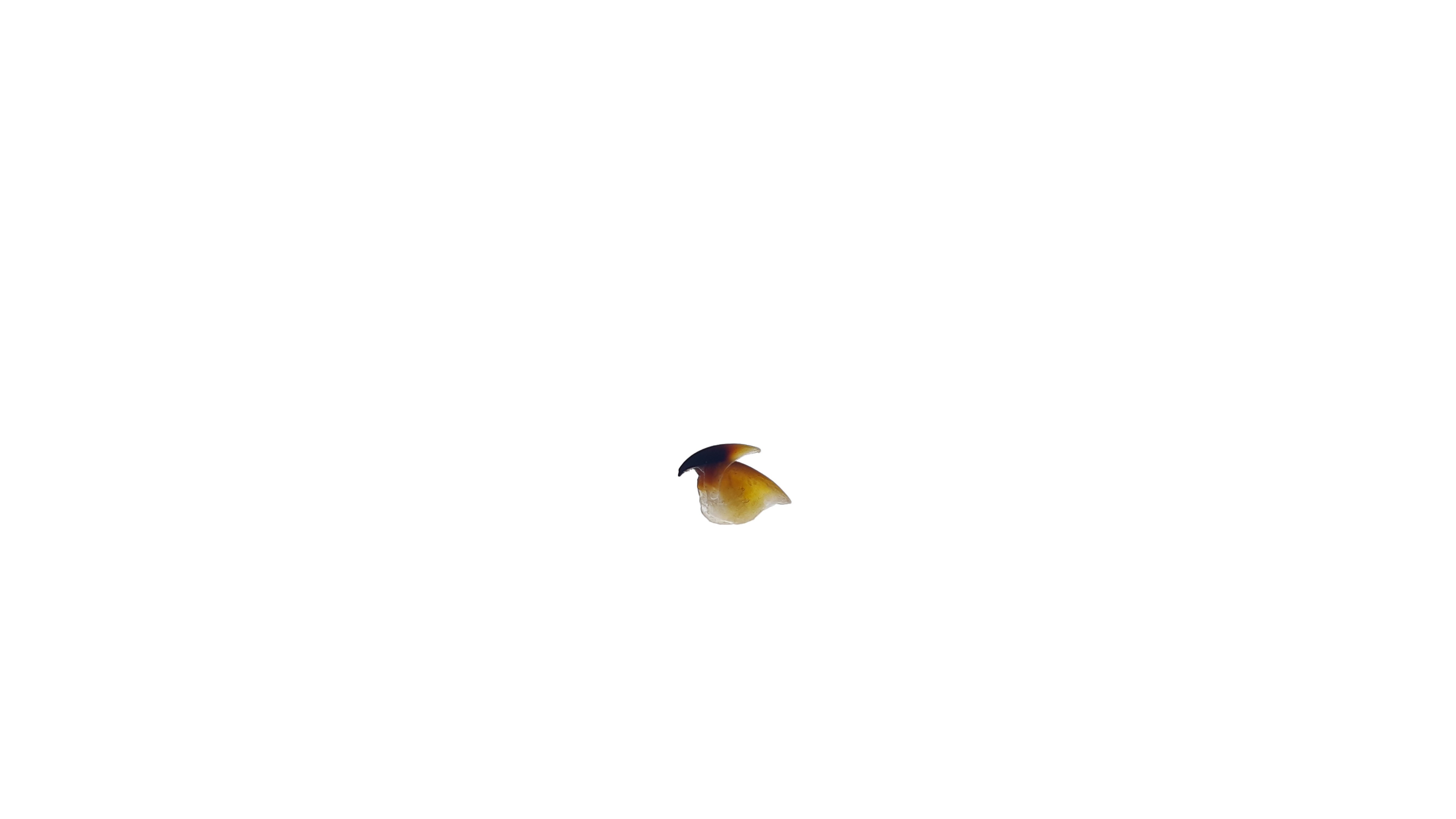

Supplement: Supplemental Information 2 — C2-Sepia aculeata, C3-Sepioteuthis lessoniana, C6-Sepia esculenta, O2-Amphioctopus aegina, S1-Loliolus uyii, S3-Uroteuthis chinensis, S4-Uroteuthis edulis [file peerj-09-11825-s002.zip › _Preprocessing_Upper_Beak/C2/U-l-C2-19.jpg]

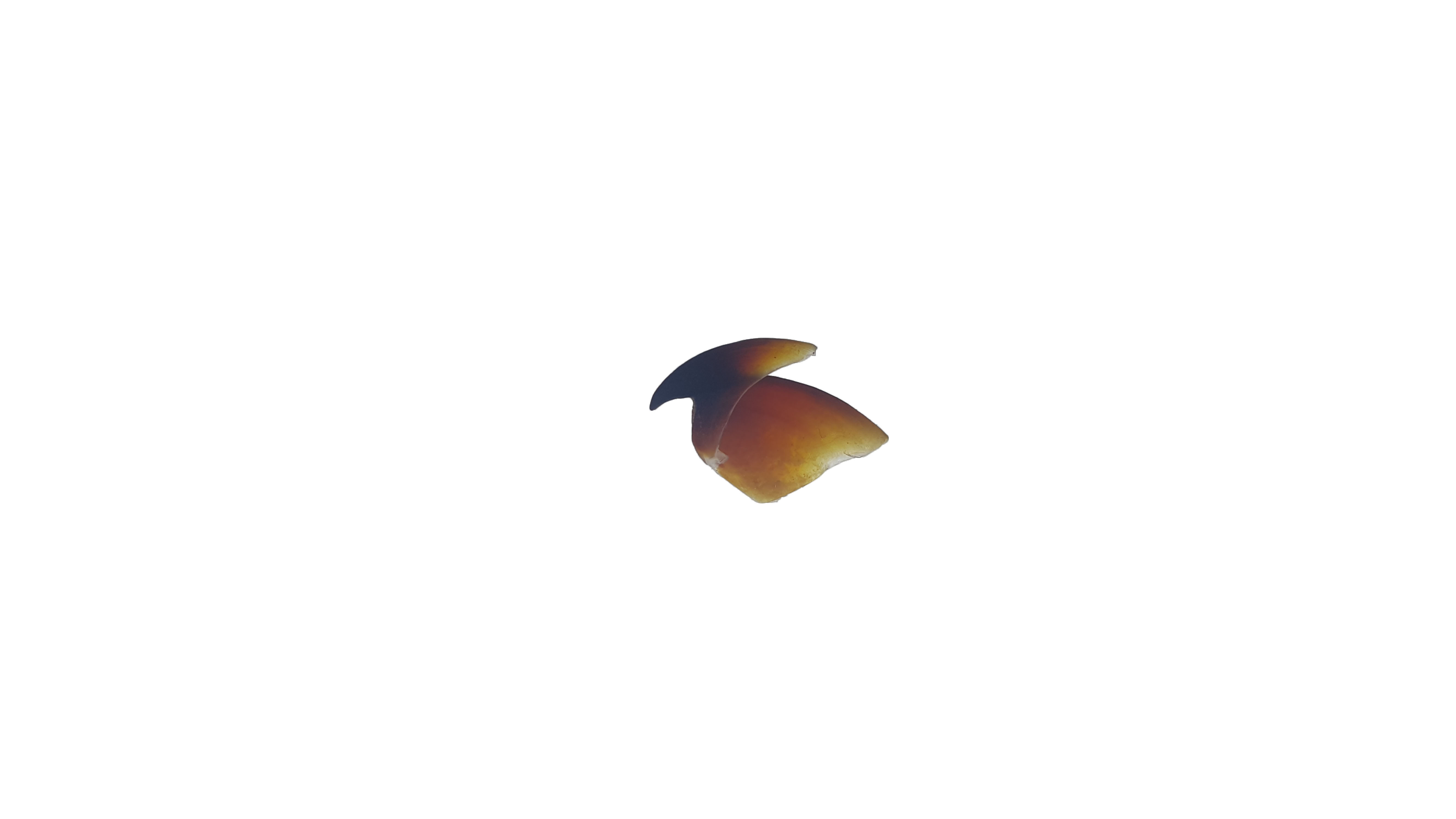

Supplement: Supplemental Information 2 — C2-Sepia aculeata, C3-Sepioteuthis lessoniana, C6-Sepia esculenta, O2-Amphioctopus aegina, S1-Loliolus uyii, S3-Uroteuthis chinensis, S4-Uroteuthis edulis [file peerj-09-11825-s002.zip › _Preprocessing_Upper_Beak/C2/U-l-C2-2.jpg]

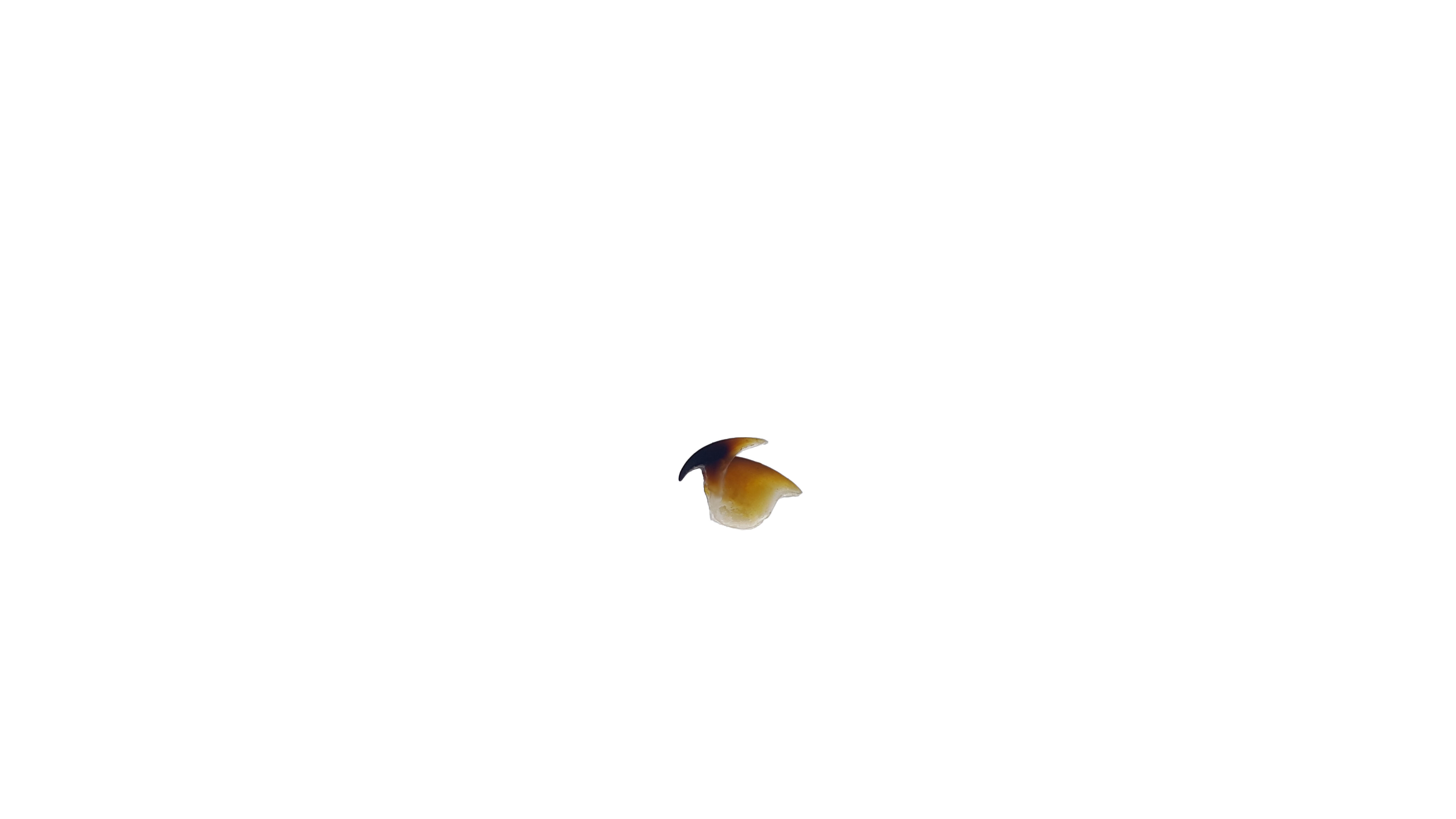

Supplement: Supplemental Information 2 — C2-Sepia aculeata, C3-Sepioteuthis lessoniana, C6-Sepia esculenta, O2-Amphioctopus aegina, S1-Loliolus uyii, S3-Uroteuthis chinensis, S4-Uroteuthis edulis [file peerj-09-11825-s002.zip › _Preprocessing_Upper_Beak/C2/U-l-C2-20.jpg]

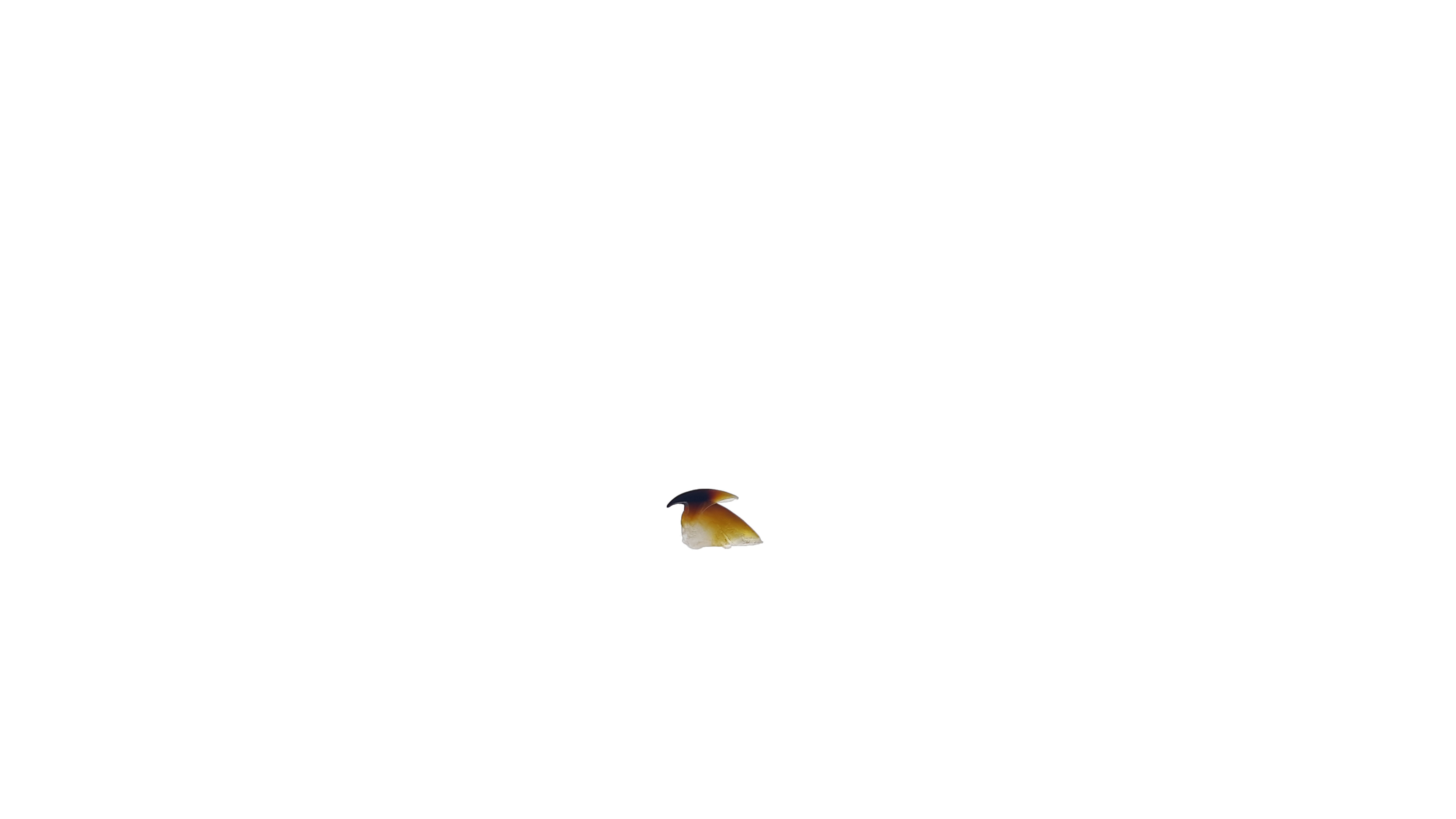

Supplement: Supplemental Information 2 — C2-Sepia aculeata, C3-Sepioteuthis lessoniana, C6-Sepia esculenta, O2-Amphioctopus aegina, S1-Loliolus uyii, S3-Uroteuthis chinensis, S4-Uroteuthis edulis [file peerj-09-11825-s002.zip › _Preprocessing_Upper_Beak/C2/U-l-C2-21.jpg]

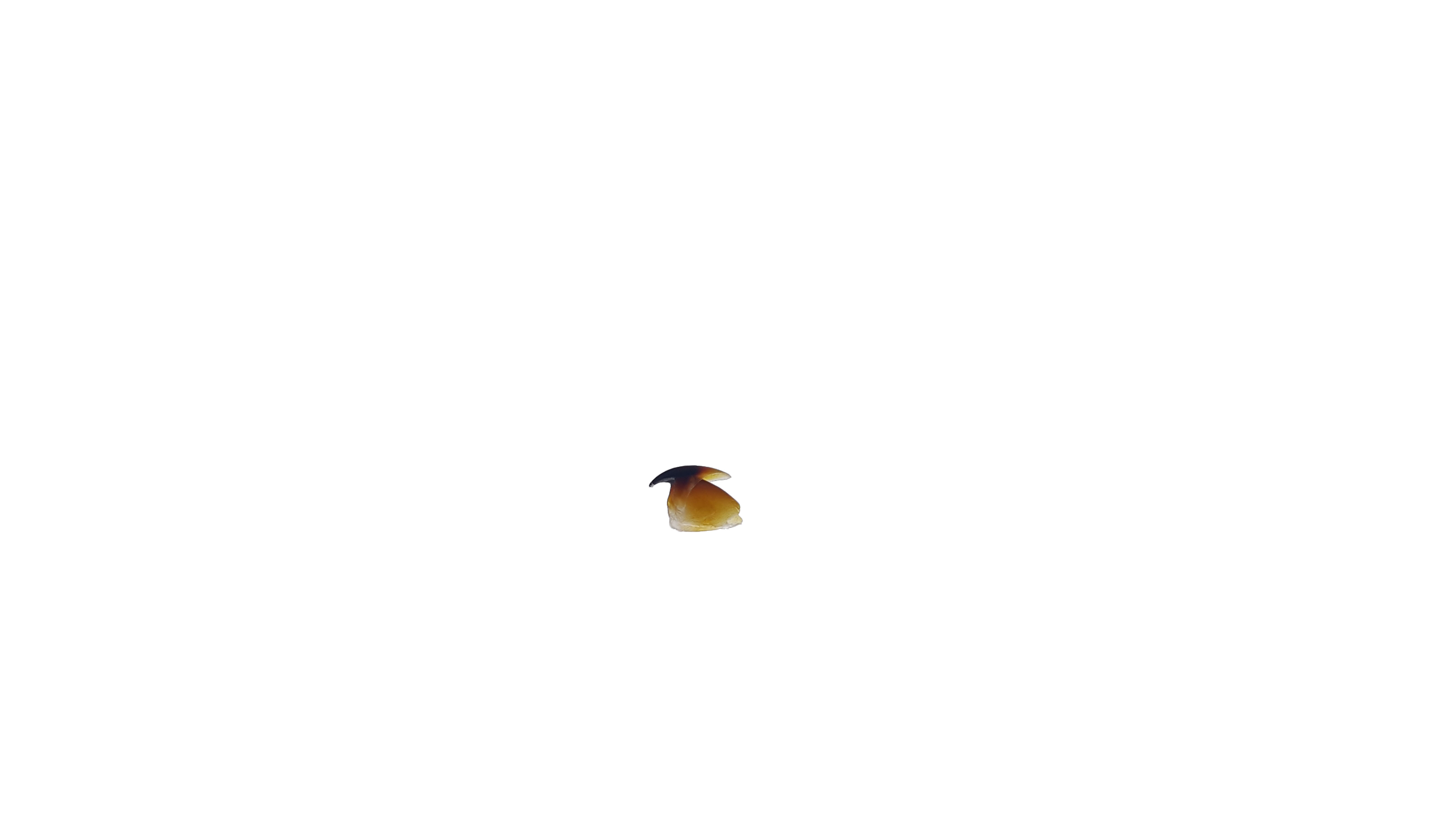

Supplement: Supplemental Information 2 — C2-Sepia aculeata, C3-Sepioteuthis lessoniana, C6-Sepia esculenta, O2-Amphioctopus aegina, S1-Loliolus uyii, S3-Uroteuthis chinensis, S4-Uroteuthis edulis [file peerj-09-11825-s002.zip › _Preprocessing_Upper_Beak/C2/U-l-C2-22.jpg]

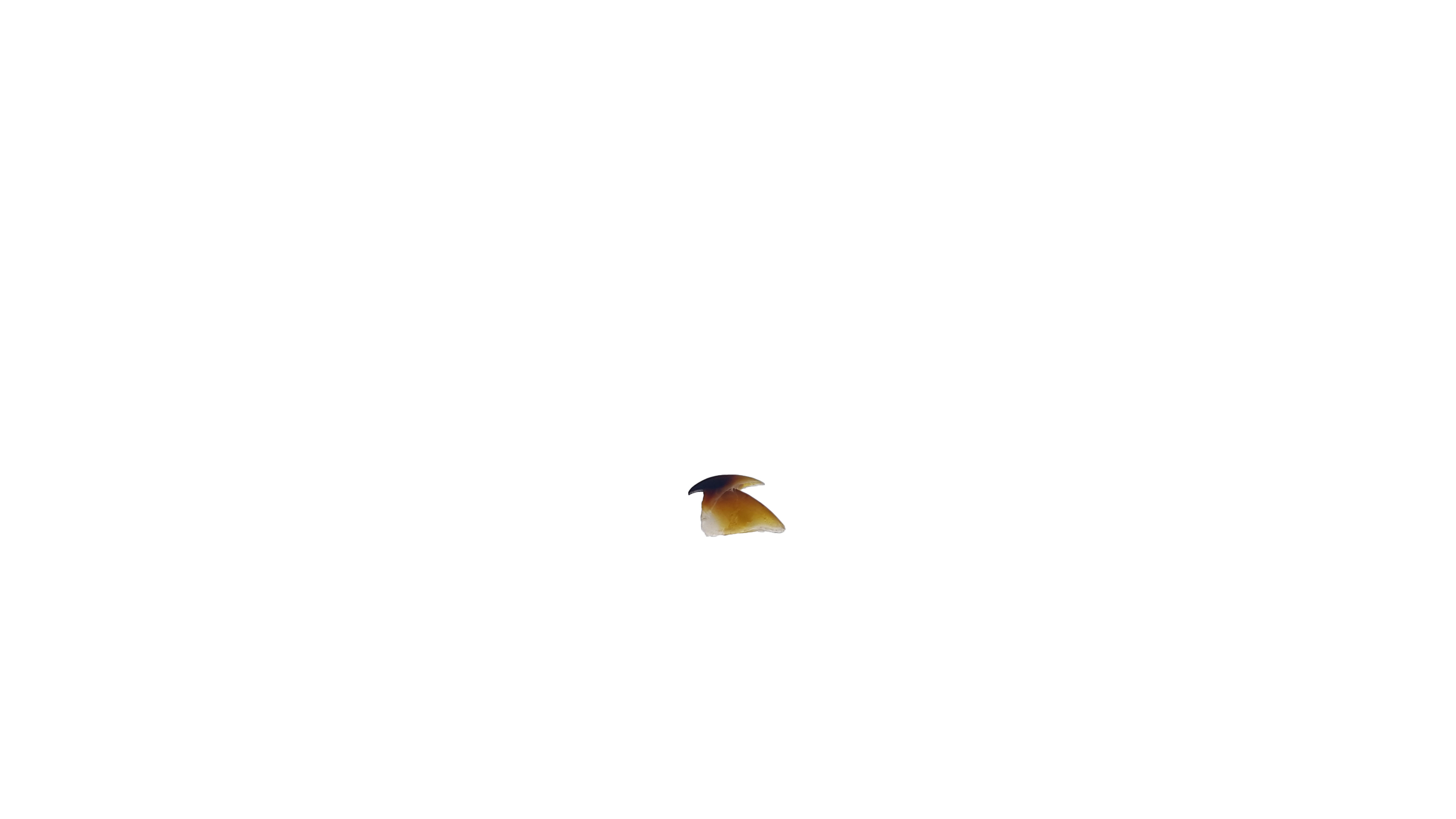

Supplement: Supplemental Information 2 — C2-Sepia aculeata, C3-Sepioteuthis lessoniana, C6-Sepia esculenta, O2-Amphioctopus aegina, S1-Loliolus uyii, S3-Uroteuthis chinensis, S4-Uroteuthis edulis [file peerj-09-11825-s002.zip › _Preprocessing_Upper_Beak/C2/U-l-C2-23.jpg]

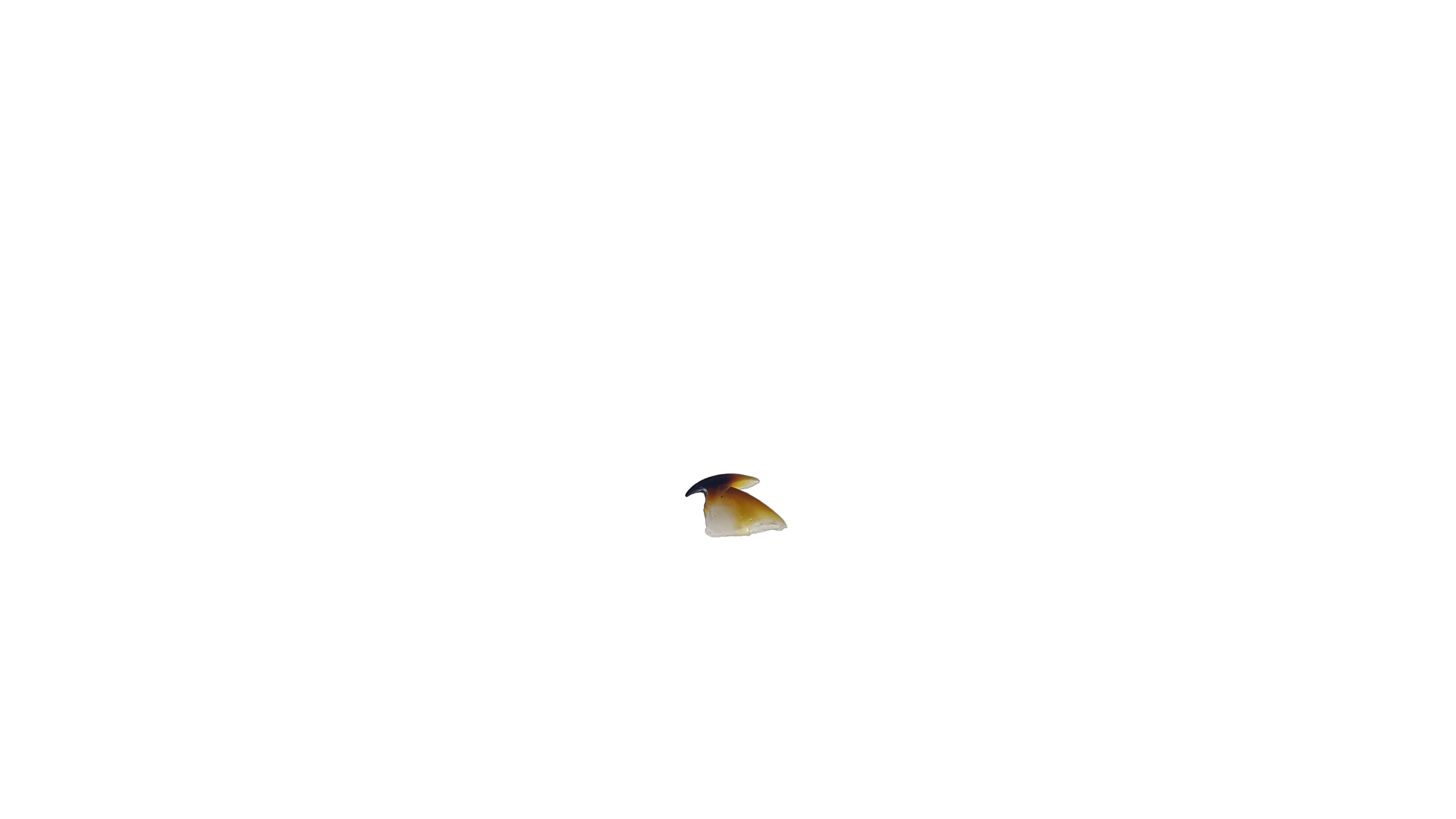

Supplement: Supplemental Information 2 — C2-Sepia aculeata, C3-Sepioteuthis lessoniana, C6-Sepia esculenta, O2-Amphioctopus aegina, S1-Loliolus uyii, S3-Uroteuthis chinensis, S4-Uroteuthis edulis [file peerj-09-11825-s002.zip › _Preprocessing_Upper_Beak/C2/U-l-C2-24.jpg]

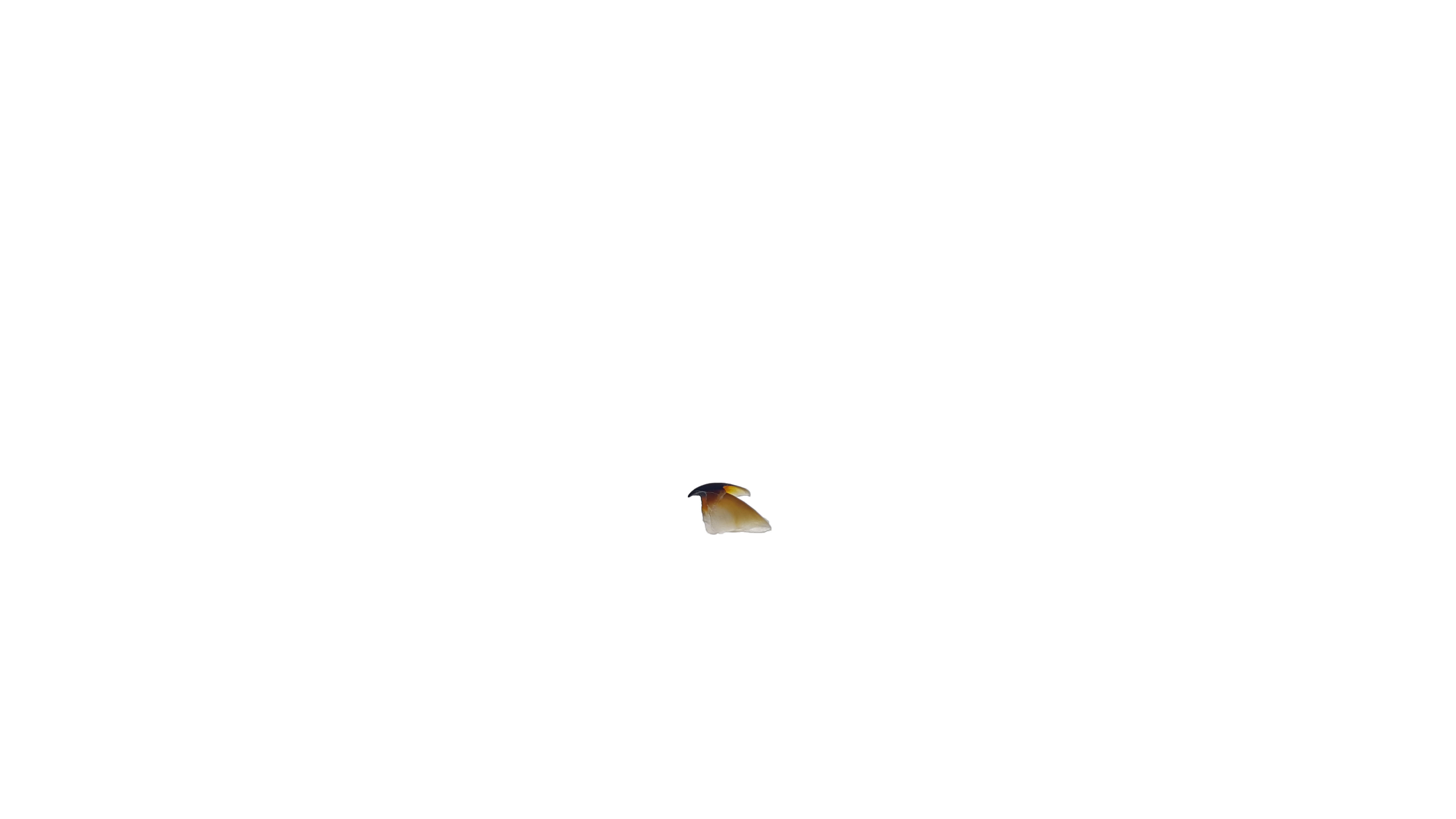

Supplement: Supplemental Information 2 — C2-Sepia aculeata, C3-Sepioteuthis lessoniana, C6-Sepia esculenta, O2-Amphioctopus aegina, S1-Loliolus uyii, S3-Uroteuthis chinensis, S4-Uroteuthis edulis [file peerj-09-11825-s002.zip › _Preprocessing_Upper_Beak/C2/U-l-C2-25.jpg]

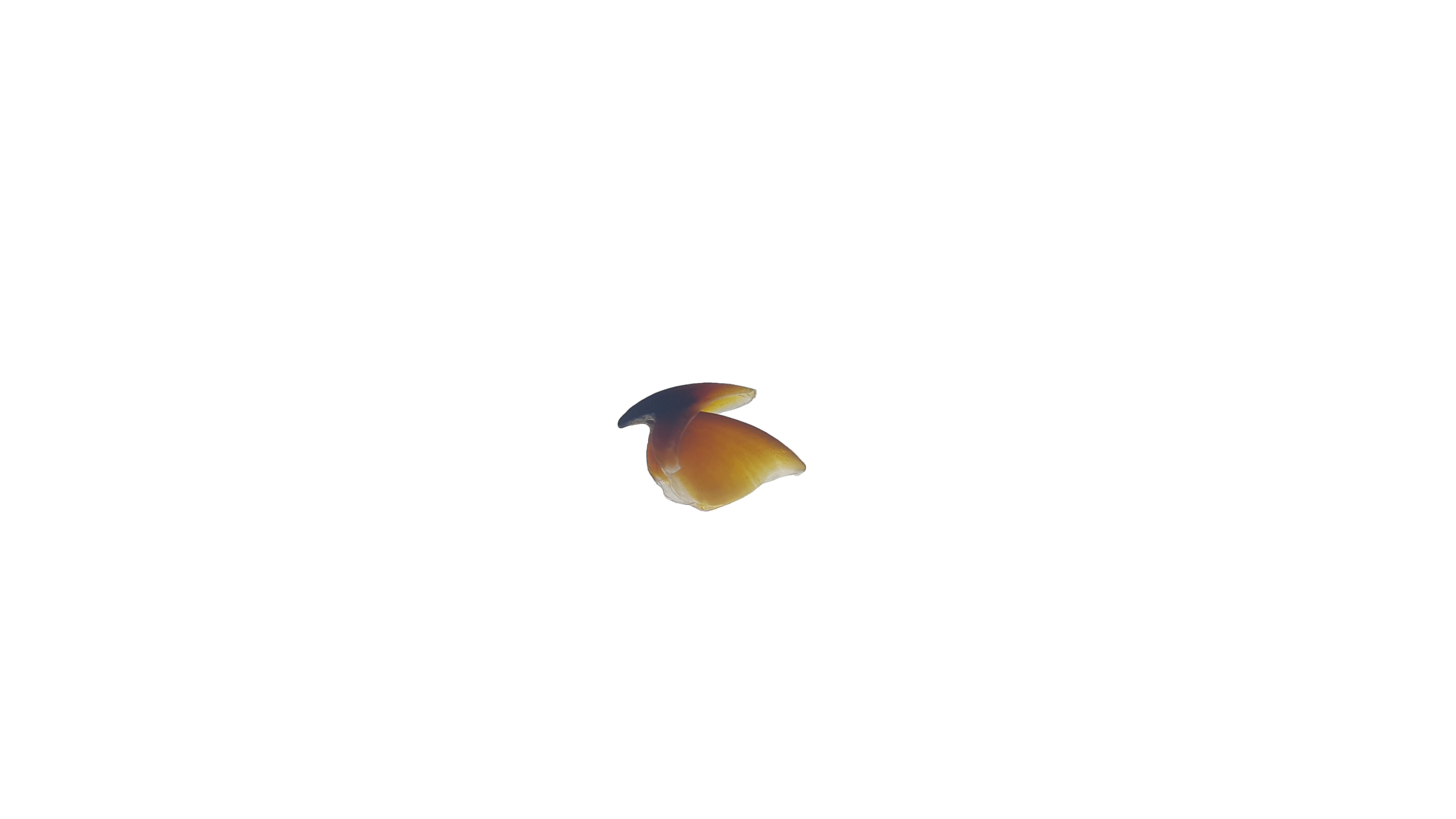

Supplement: Supplemental Information 2 — C2-Sepia aculeata, C3-Sepioteuthis lessoniana, C6-Sepia esculenta, O2-Amphioctopus aegina, S1-Loliolus uyii, S3-Uroteuthis chinensis, S4-Uroteuthis edulis [file peerj-09-11825-s002.zip › _Preprocessing_Upper_Beak/C2/U-l-C2-3.jpg]

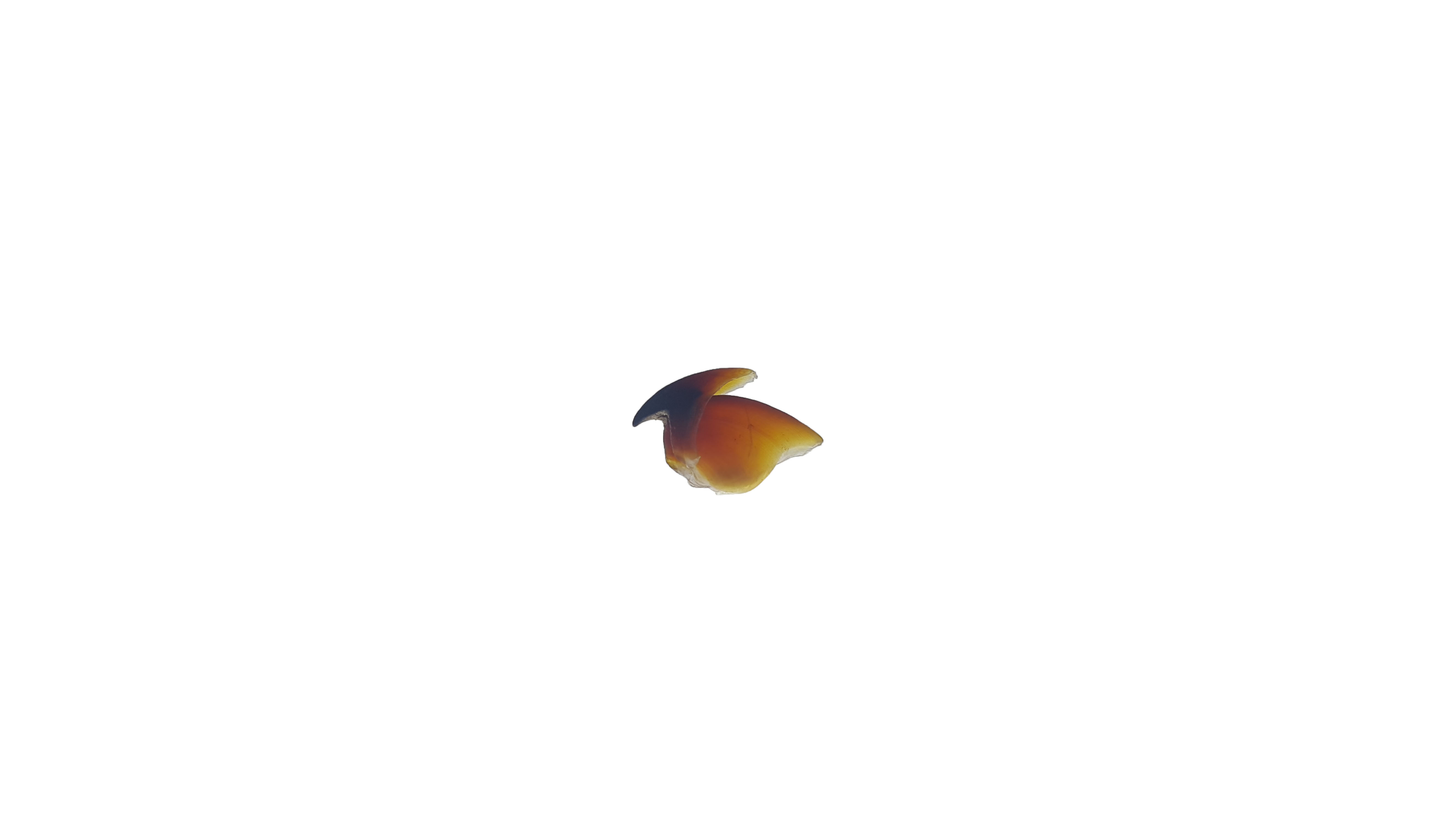

Supplement: Supplemental Information 2 — C2-Sepia aculeata, C3-Sepioteuthis lessoniana, C6-Sepia esculenta, O2-Amphioctopus aegina, S1-Loliolus uyii, S3-Uroteuthis chinensis, S4-Uroteuthis edulis [file peerj-09-11825-s002.zip › _Preprocessing_Upper_Beak/C2/U-l-C2-4.jpg]

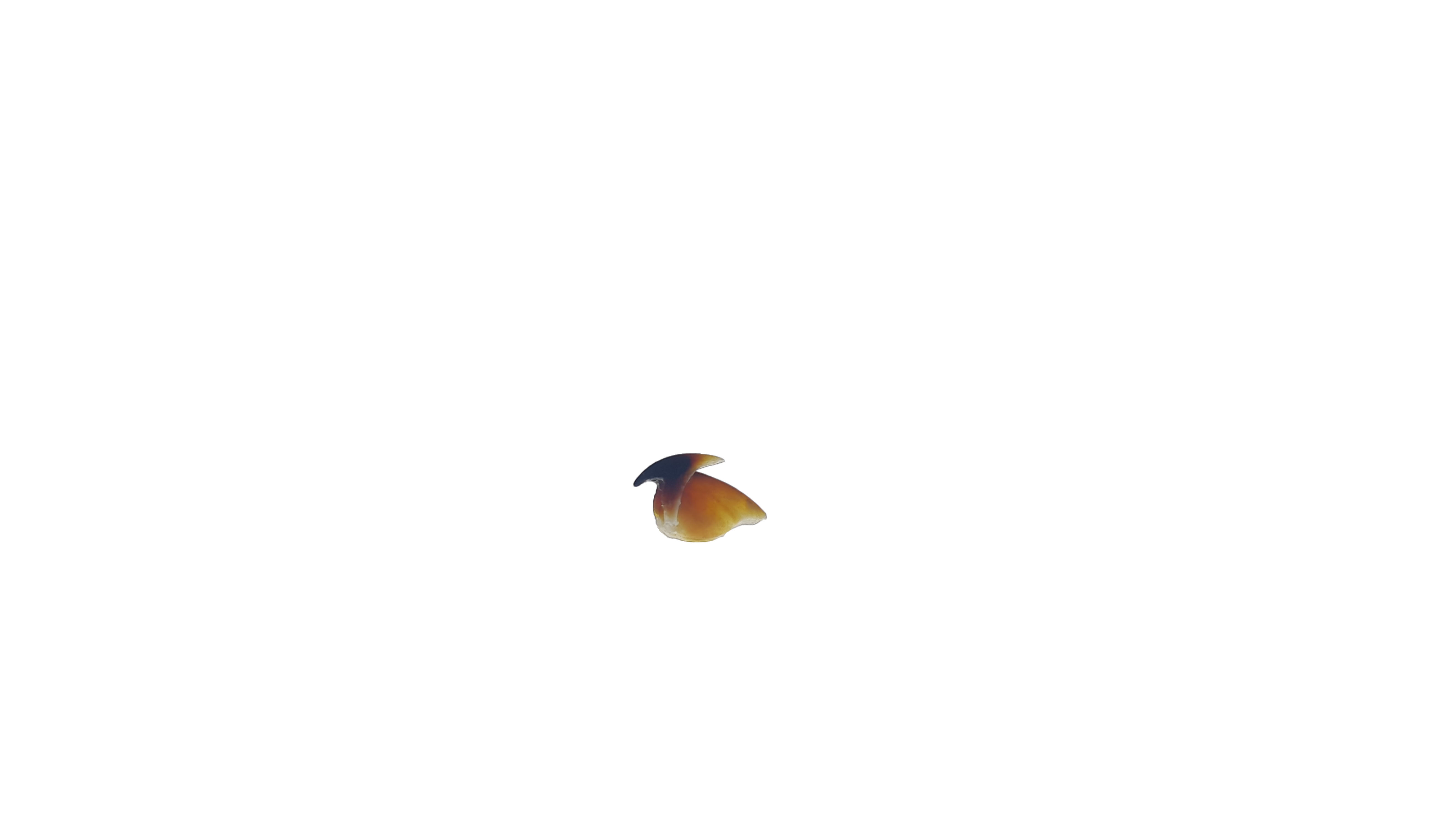

Supplement: Supplemental Information 2 — C2-Sepia aculeata, C3-Sepioteuthis lessoniana, C6-Sepia esculenta, O2-Amphioctopus aegina, S1-Loliolus uyii, S3-Uroteuthis chinensis, S4-Uroteuthis edulis [file peerj-09-11825-s002.zip › _Preprocessing_Upper_Beak/C2/U-l-C2-5.jpg]

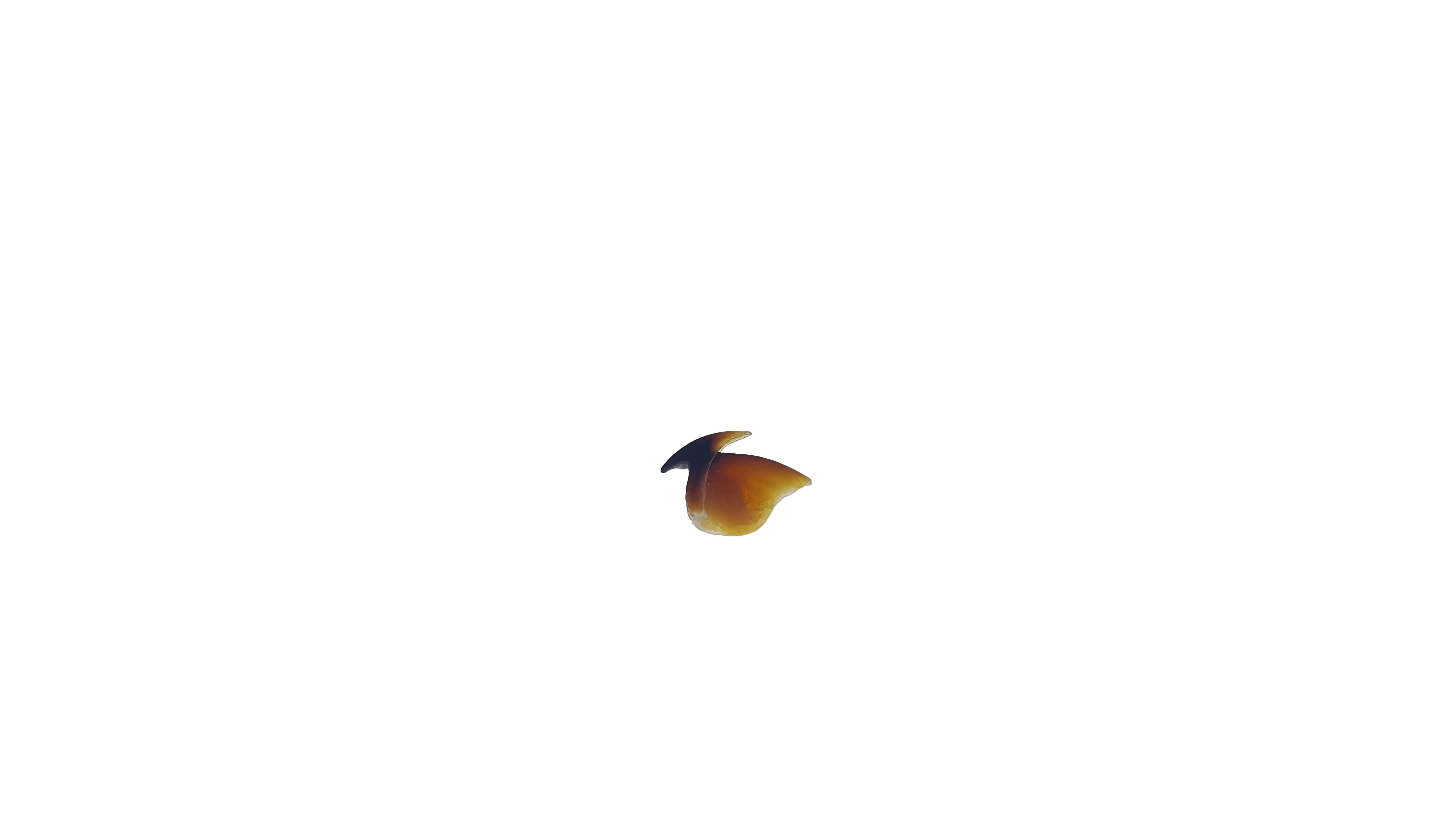

Supplement: Supplemental Information 2 — C2-Sepia aculeata, C3-Sepioteuthis lessoniana, C6-Sepia esculenta, O2-Amphioctopus aegina, S1-Loliolus uyii, S3-Uroteuthis chinensis, S4-Uroteuthis edulis [file peerj-09-11825-s002.zip › _Preprocessing_Upper_Beak/C2/U-l-C2-6.jpg]

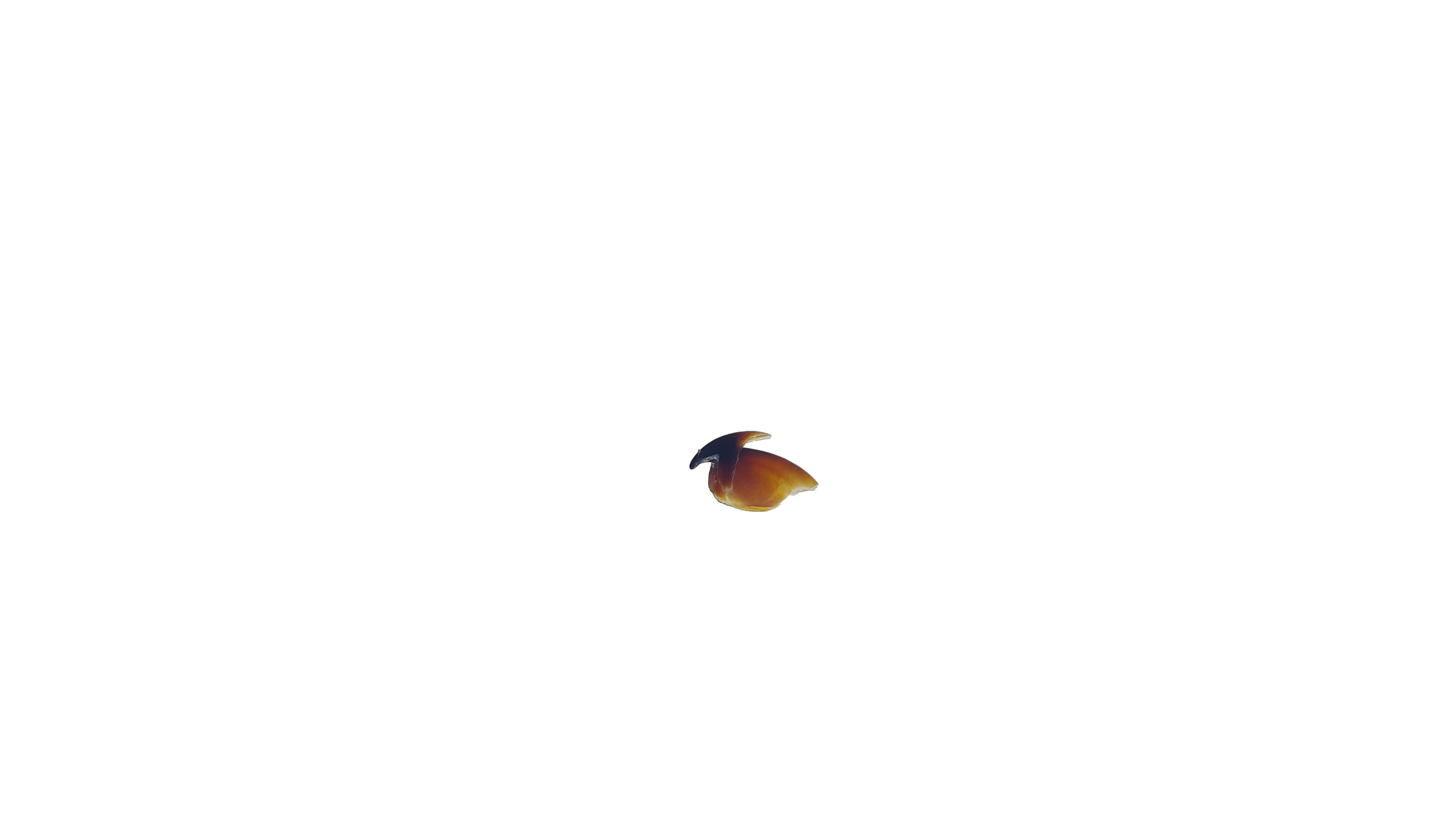

Supplement: Supplemental Information 2 — C2-Sepia aculeata, C3-Sepioteuthis lessoniana, C6-Sepia esculenta, O2-Amphioctopus aegina, S1-Loliolus uyii, S3-Uroteuthis chinensis, S4-Uroteuthis edulis [file peerj-09-11825-s002.zip › _Preprocessing_Upper_Beak/C2/U-l-C2-7.jpg]

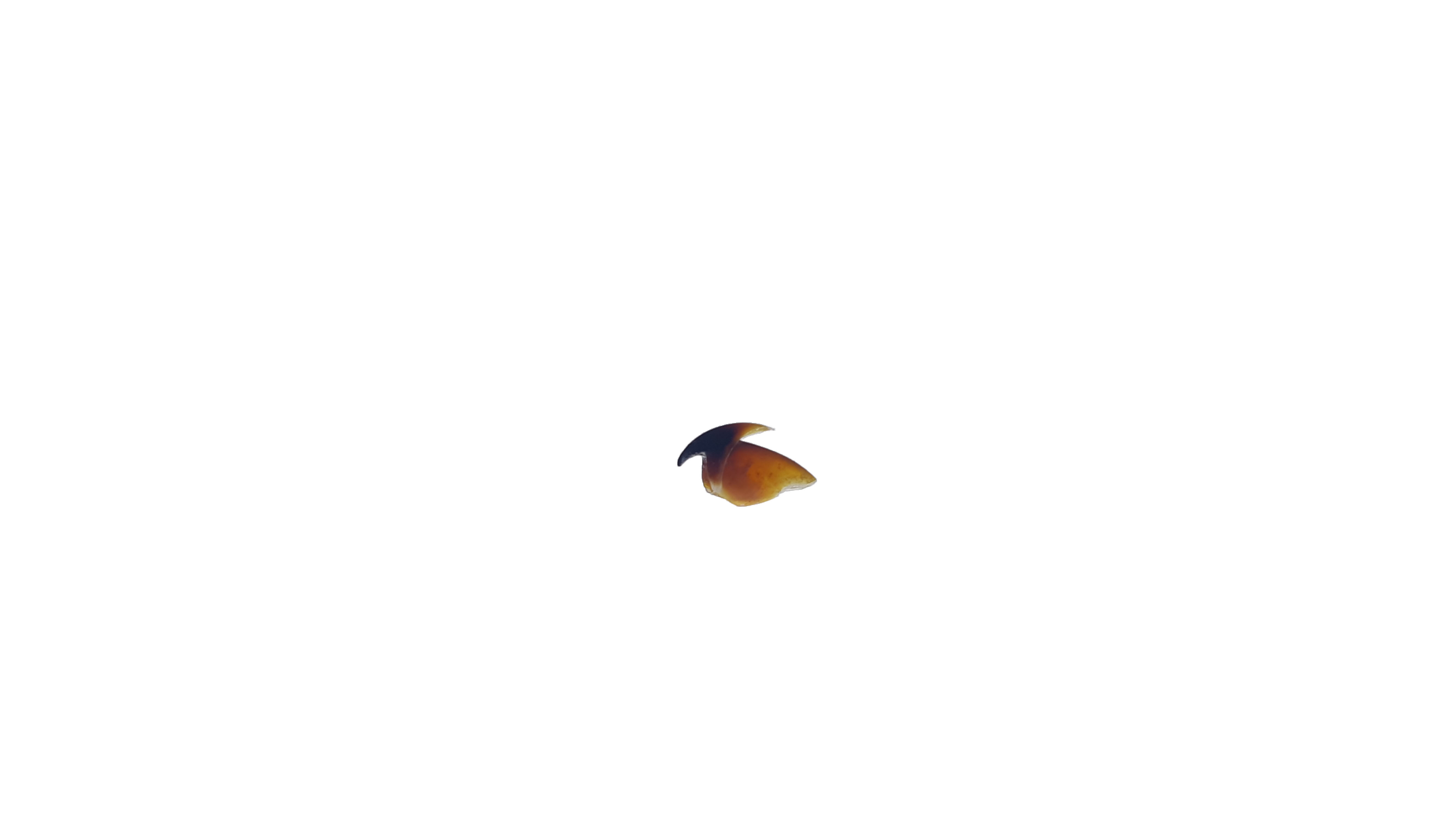

Supplement: Supplemental Information 2 — C2-Sepia aculeata, C3-Sepioteuthis lessoniana, C6-Sepia esculenta, O2-Amphioctopus aegina, S1-Loliolus uyii, S3-Uroteuthis chinensis, S4-Uroteuthis edulis [file peerj-09-11825-s002.zip › _Preprocessing_Upper_Beak/C2/U-l-C2-8.jpg]

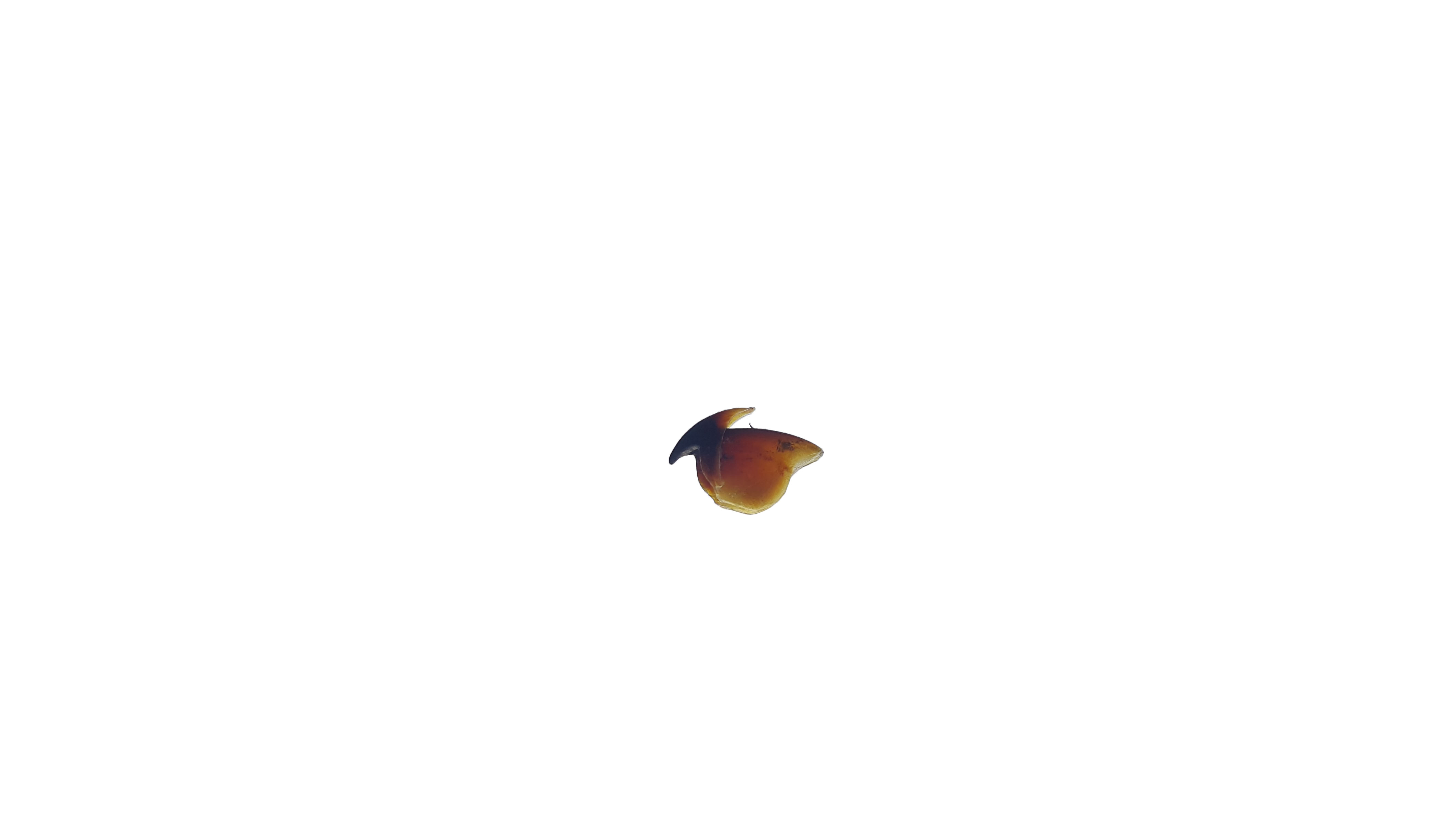

Supplement: Supplemental Information 2 — C2-Sepia aculeata, C3-Sepioteuthis lessoniana, C6-Sepia esculenta, O2-Amphioctopus aegina, S1-Loliolus uyii, S3-Uroteuthis chinensis, S4-Uroteuthis edulis [file peerj-09-11825-s002.zip › _Preprocessing_Upper_Beak/C2/U-l-C2-9.jpg]

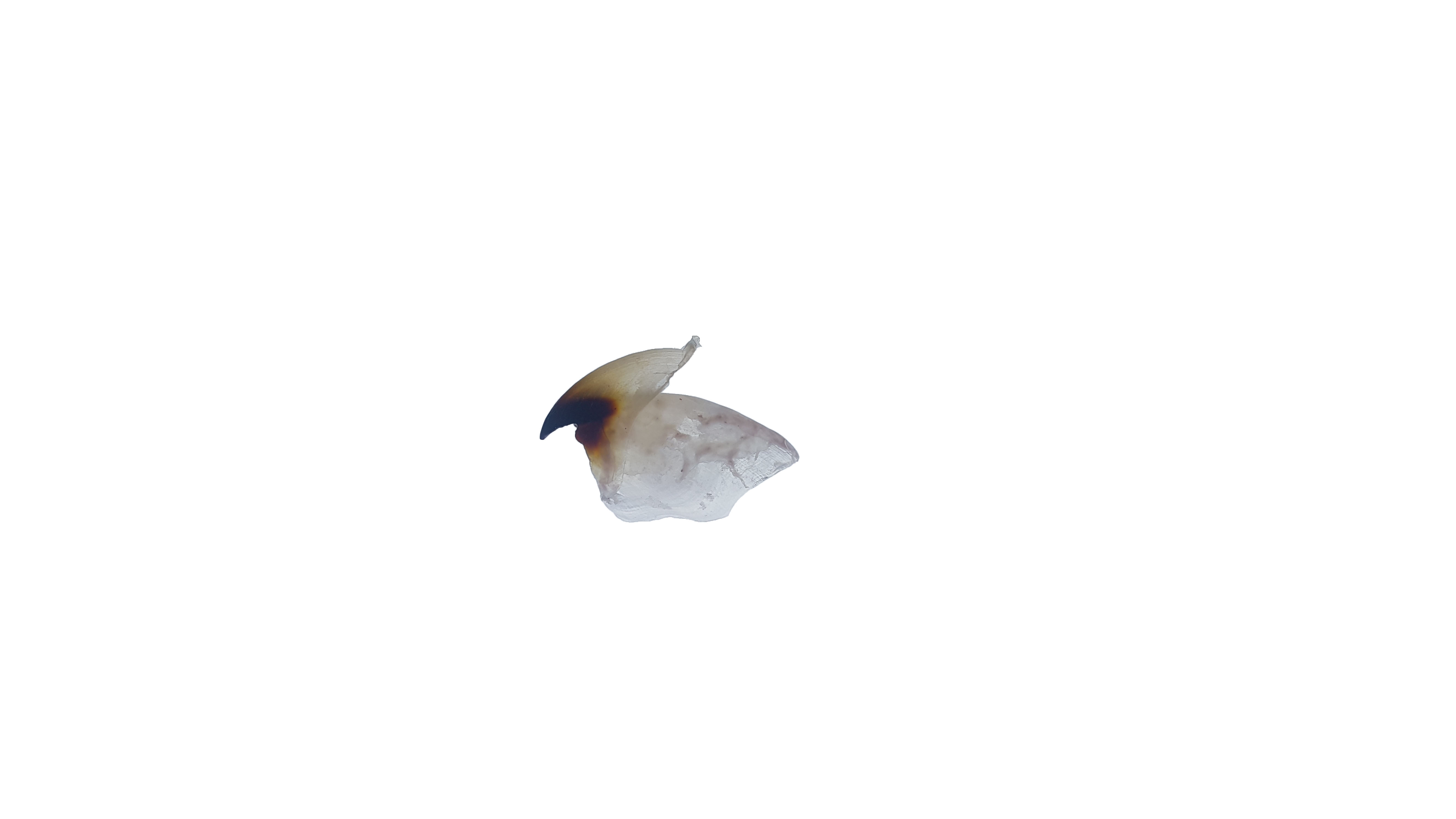

Supplement: Supplemental Information 2 — C2-Sepia aculeata, C3-Sepioteuthis lessoniana, C6-Sepia esculenta, O2-Amphioctopus aegina, S1-Loliolus uyii, S3-Uroteuthis chinensis, S4-Uroteuthis edulis [file peerj-09-11825-s002.zip › _Preprocessing_Upper_Beak/C3/U-l-C3-1.jpg]
