# Supplementary figures and images for: Cephalopod species identification using integrated analysis of machine learning and deep learning approaches (part 3 of 4)
Source: PeerJ. 2021 Aug 9;9:e11825. doi: 10.7717/peerj.11825 (PMC8359798; doi:10.7717/peerj.11825)

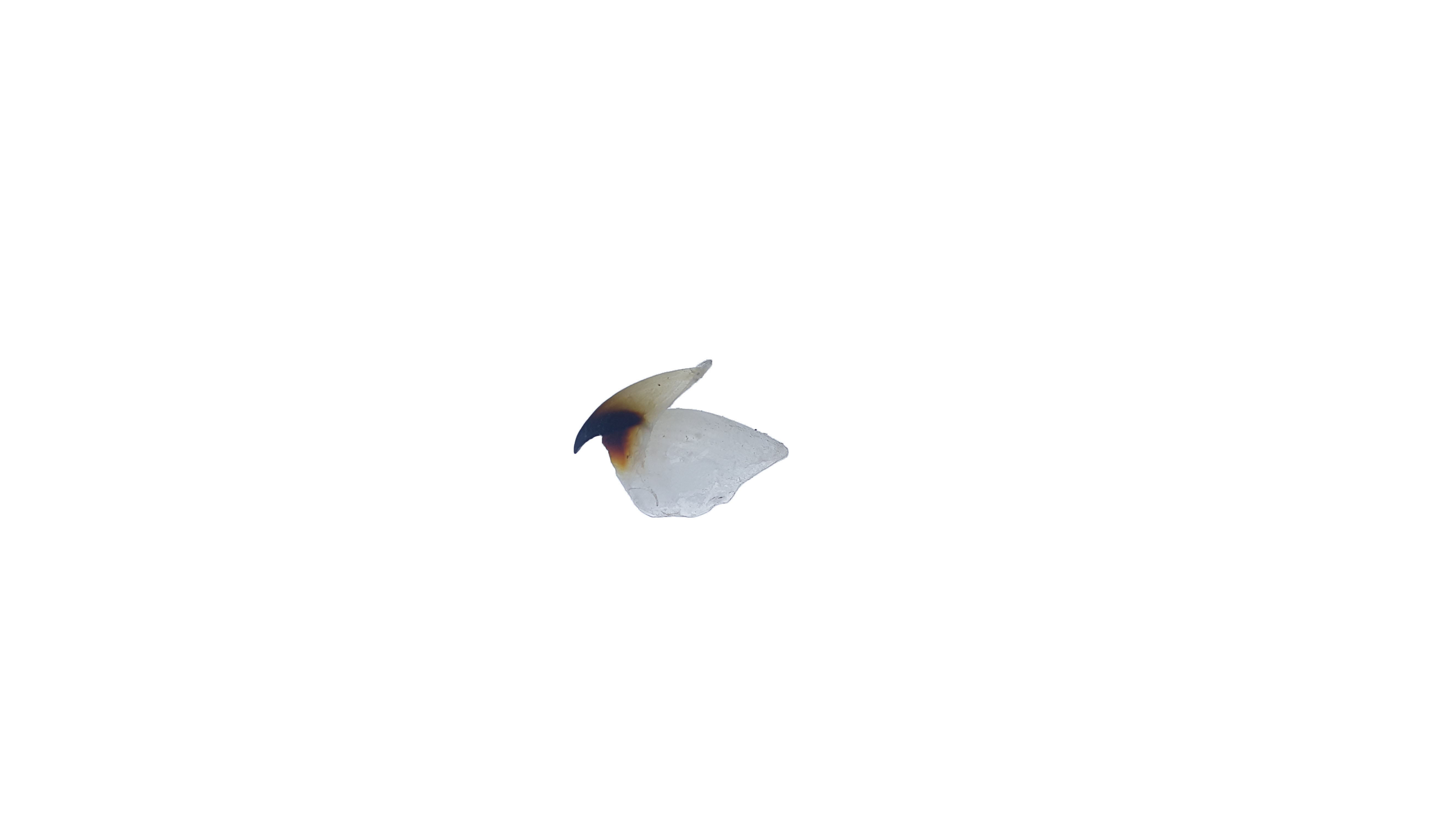

Supplement: Supplemental Information 2 — C2-Sepia aculeata, C3-Sepioteuthis lessoniana, C6-Sepia esculenta, O2-Amphioctopus aegina, S1-Loliolus uyii, S3-Uroteuthis chinensis, S4-Uroteuthis edulis [file peerj-09-11825-s002.zip › _Preprocessing_Upper_Beak/C3/U-l-C3-10.jpg]

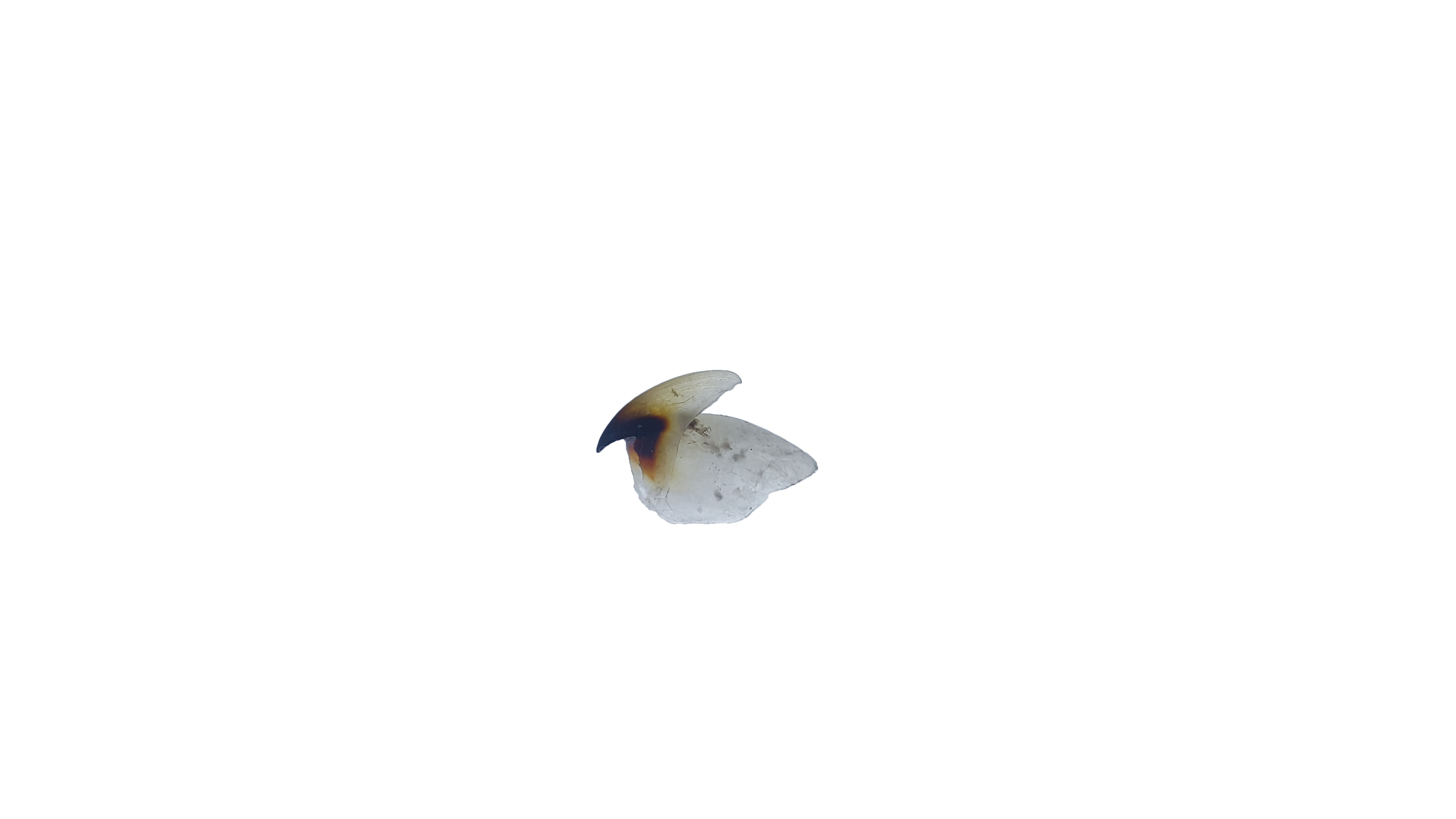

Supplement: Supplemental Information 2 — C2-Sepia aculeata, C3-Sepioteuthis lessoniana, C6-Sepia esculenta, O2-Amphioctopus aegina, S1-Loliolus uyii, S3-Uroteuthis chinensis, S4-Uroteuthis edulis [file peerj-09-11825-s002.zip › _Preprocessing_Upper_Beak/C3/U-l-C3-11.jpg]

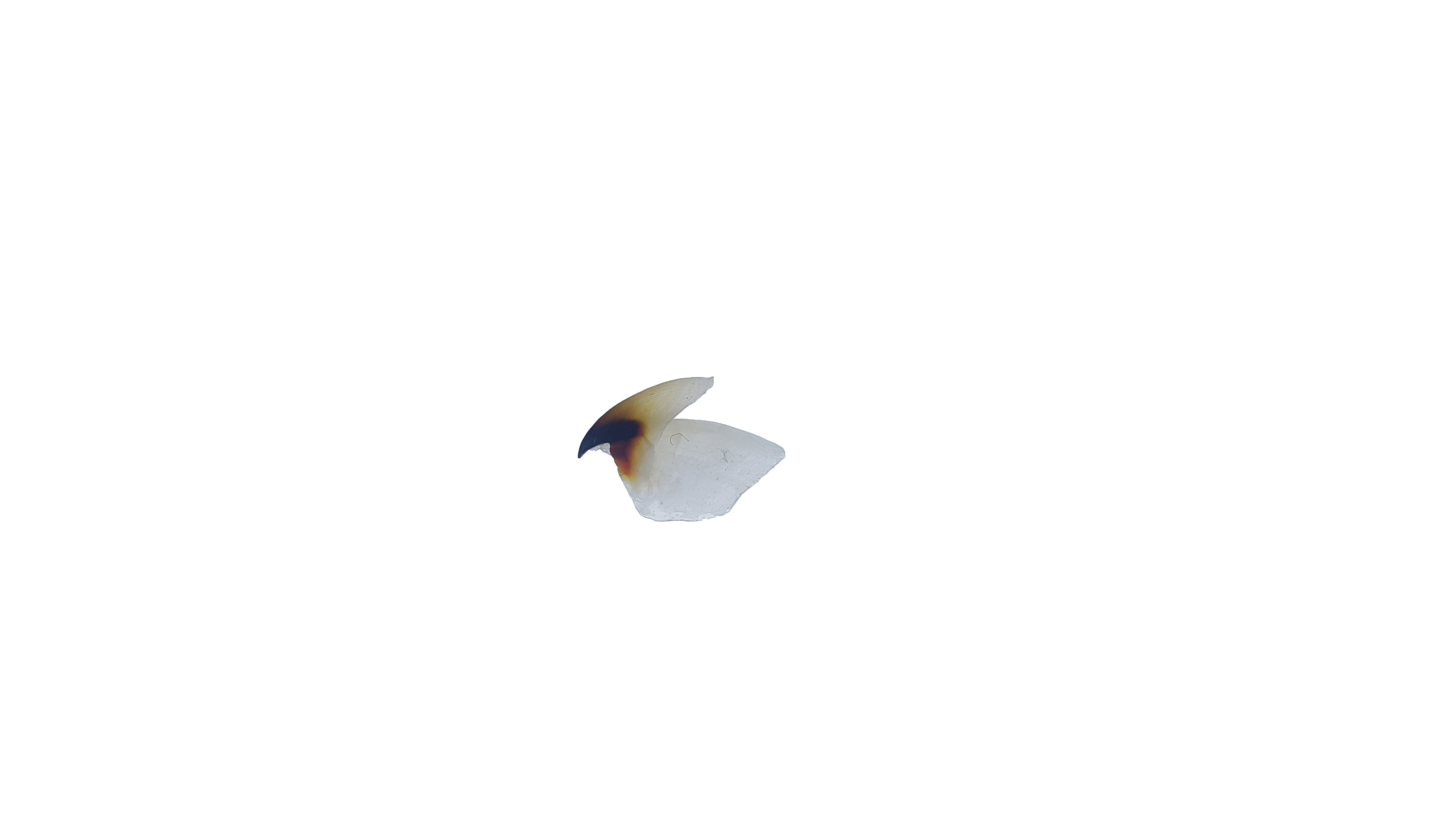

Supplement: Supplemental Information 2 — C2-Sepia aculeata, C3-Sepioteuthis lessoniana, C6-Sepia esculenta, O2-Amphioctopus aegina, S1-Loliolus uyii, S3-Uroteuthis chinensis, S4-Uroteuthis edulis [file peerj-09-11825-s002.zip › _Preprocessing_Upper_Beak/C3/U-l-C3-12.jpg]

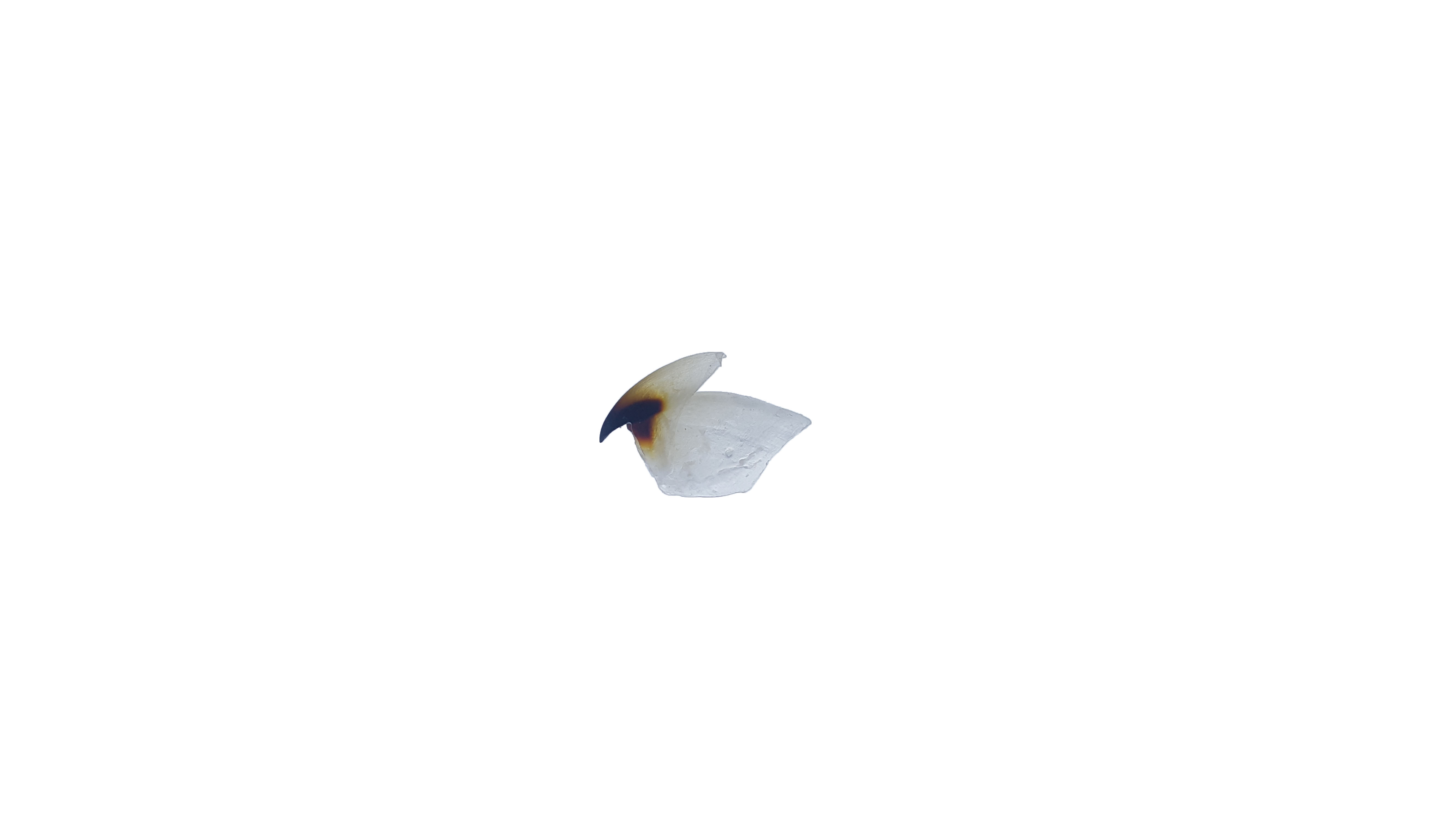

Supplement: Supplemental Information 2 — C2-Sepia aculeata, C3-Sepioteuthis lessoniana, C6-Sepia esculenta, O2-Amphioctopus aegina, S1-Loliolus uyii, S3-Uroteuthis chinensis, S4-Uroteuthis edulis [file peerj-09-11825-s002.zip › _Preprocessing_Upper_Beak/C3/U-l-C3-13.jpg]

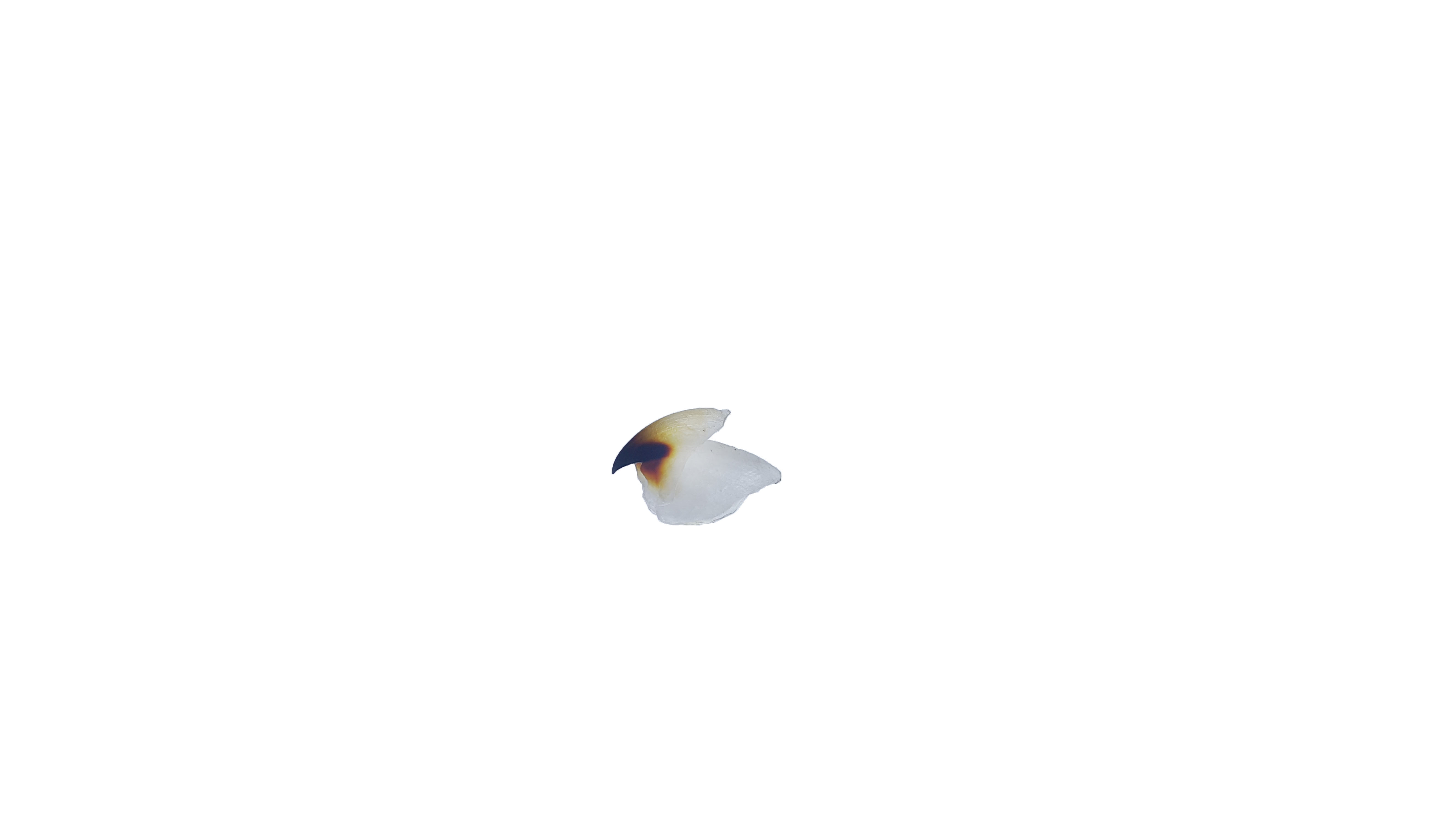

Supplement: Supplemental Information 2 — C2-Sepia aculeata, C3-Sepioteuthis lessoniana, C6-Sepia esculenta, O2-Amphioctopus aegina, S1-Loliolus uyii, S3-Uroteuthis chinensis, S4-Uroteuthis edulis [file peerj-09-11825-s002.zip › _Preprocessing_Upper_Beak/C3/U-l-C3-14.jpg]

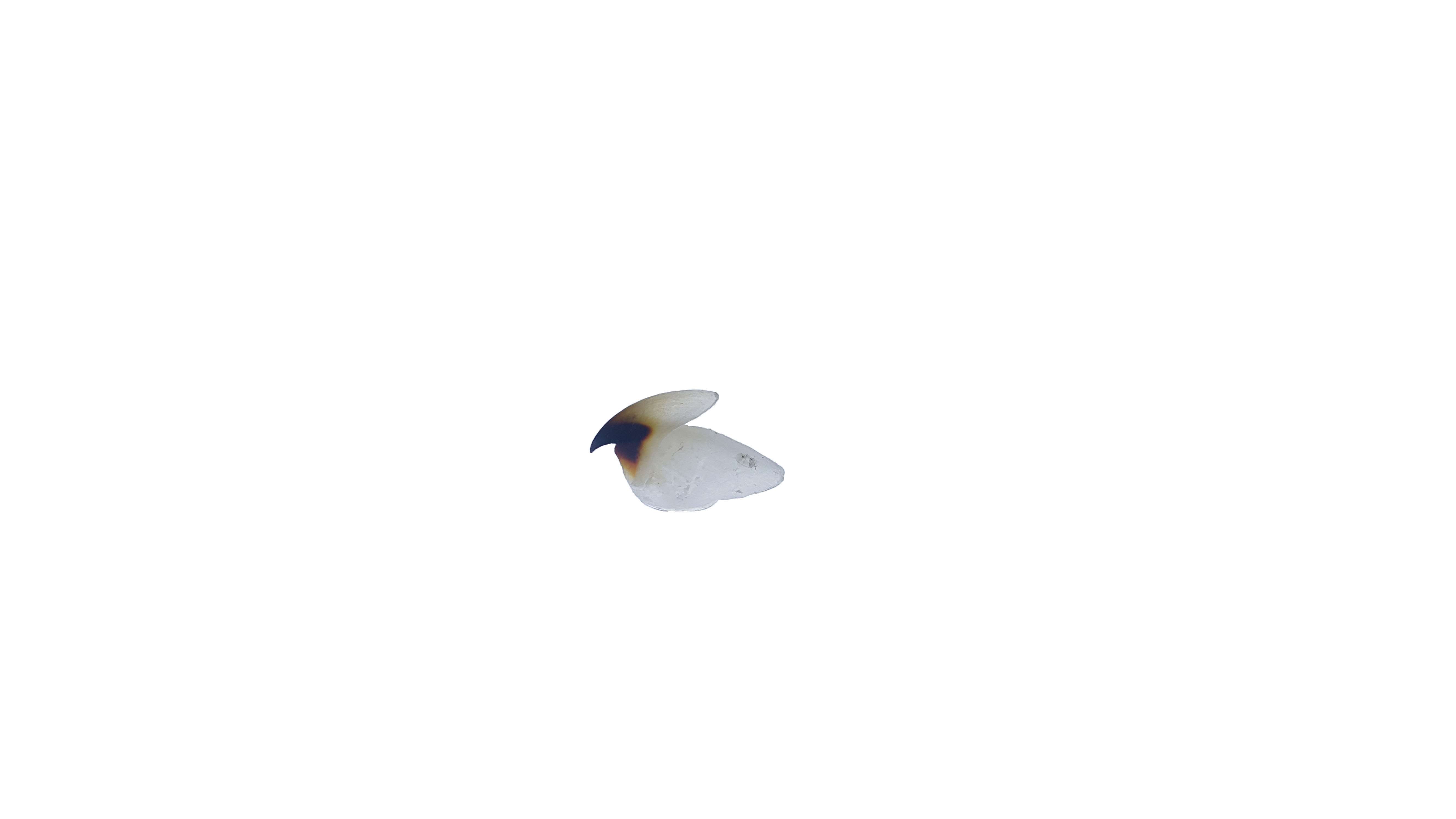

Supplement: Supplemental Information 2 — C2-Sepia aculeata, C3-Sepioteuthis lessoniana, C6-Sepia esculenta, O2-Amphioctopus aegina, S1-Loliolus uyii, S3-Uroteuthis chinensis, S4-Uroteuthis edulis [file peerj-09-11825-s002.zip › _Preprocessing_Upper_Beak/C3/U-l-C3-15.jpg]

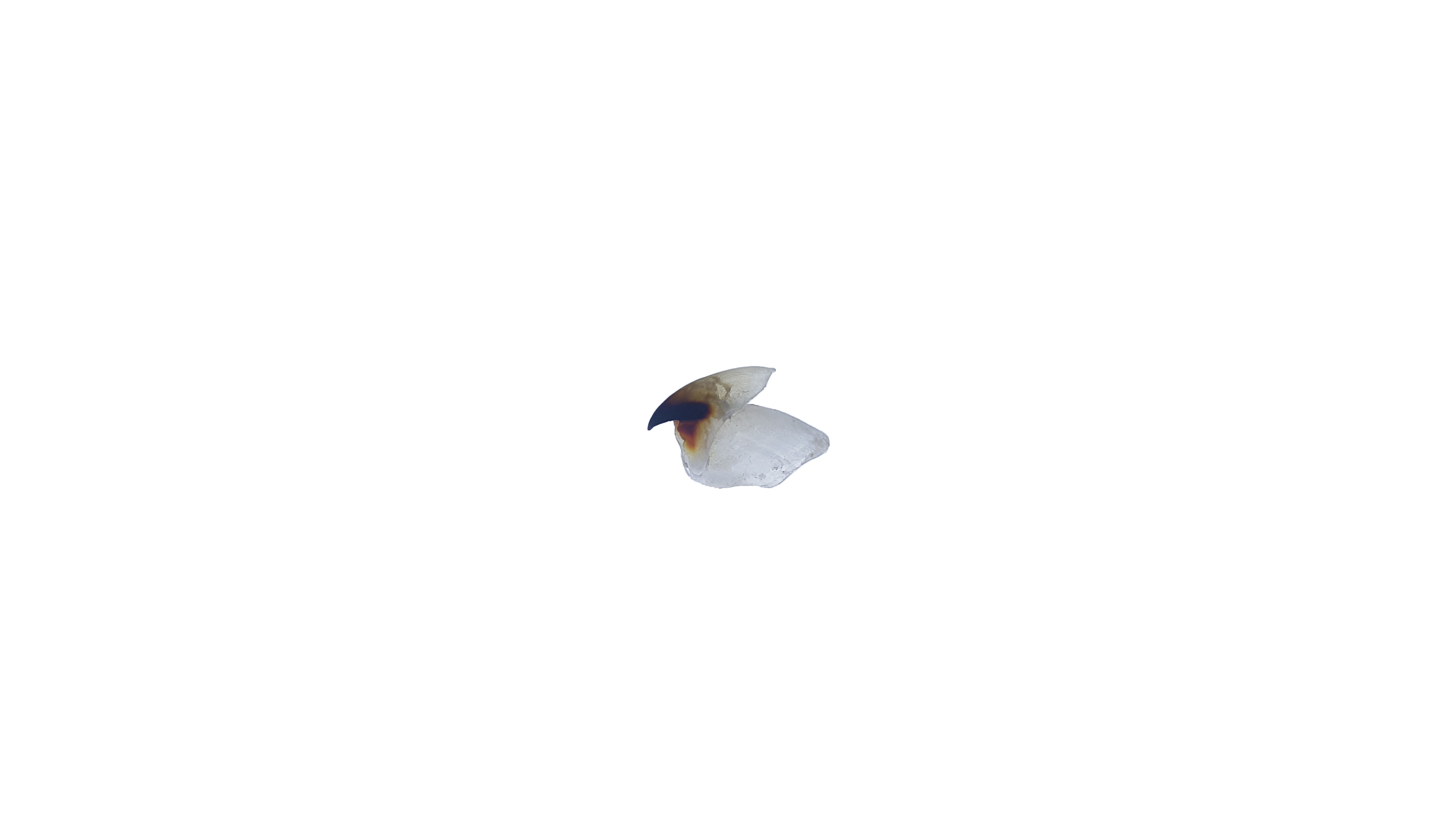

Supplement: Supplemental Information 2 — C2-Sepia aculeata, C3-Sepioteuthis lessoniana, C6-Sepia esculenta, O2-Amphioctopus aegina, S1-Loliolus uyii, S3-Uroteuthis chinensis, S4-Uroteuthis edulis [file peerj-09-11825-s002.zip › _Preprocessing_Upper_Beak/C3/U-l-C3-16.jpg]

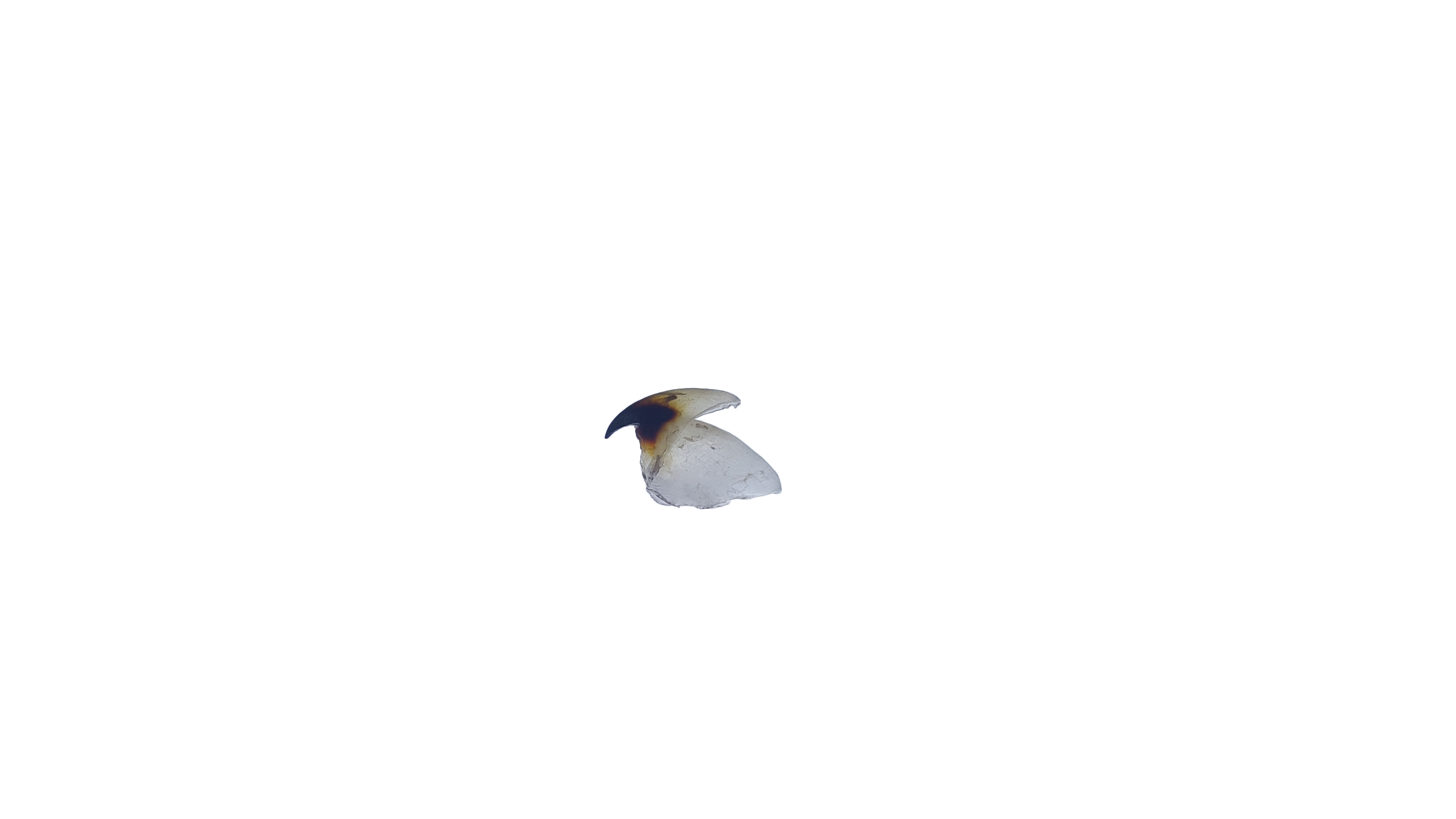

Supplement: Supplemental Information 2 — C2-Sepia aculeata, C3-Sepioteuthis lessoniana, C6-Sepia esculenta, O2-Amphioctopus aegina, S1-Loliolus uyii, S3-Uroteuthis chinensis, S4-Uroteuthis edulis [file peerj-09-11825-s002.zip › _Preprocessing_Upper_Beak/C3/U-l-C3-17.jpg]

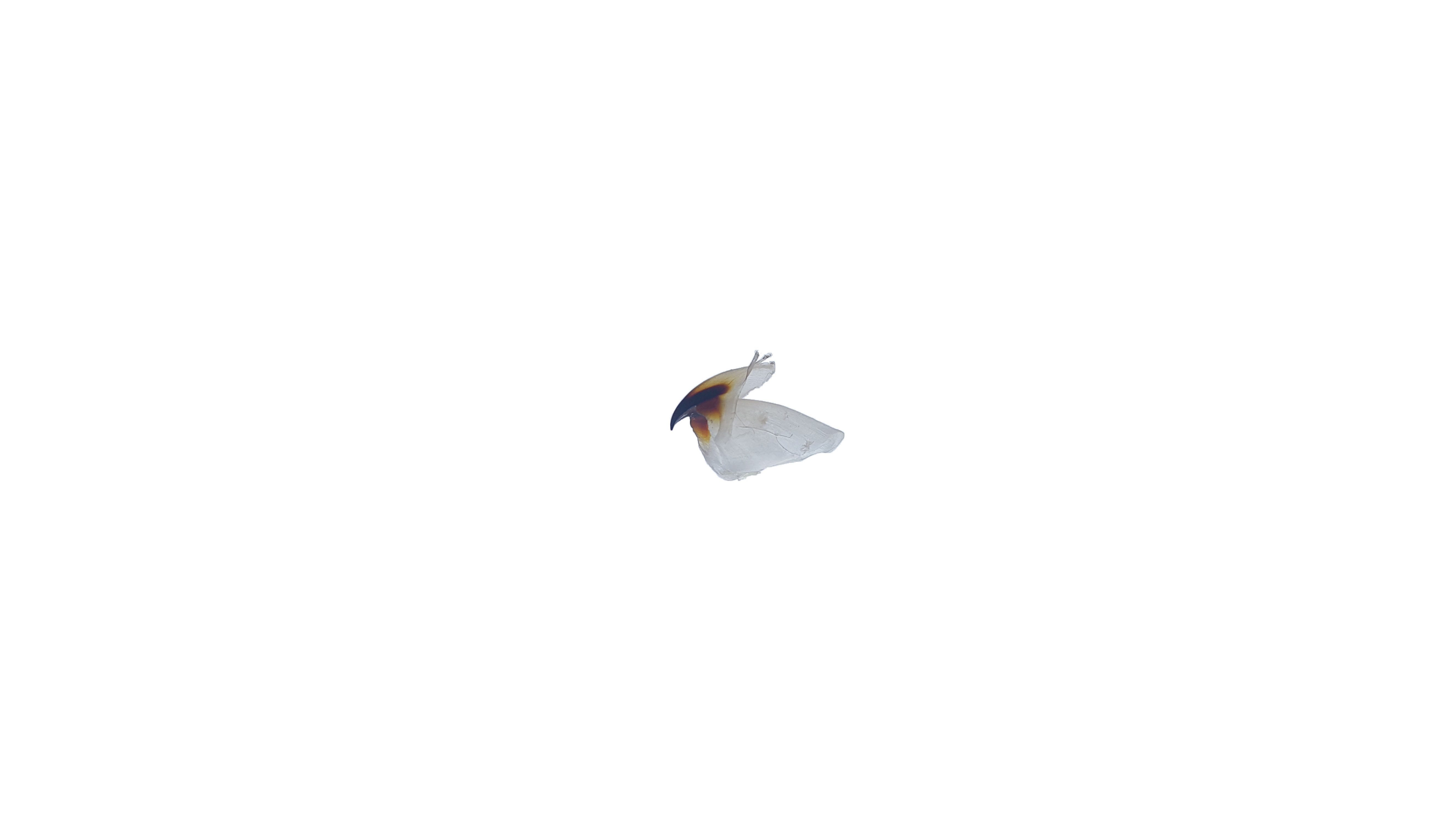

Supplement: Supplemental Information 2 — C2-Sepia aculeata, C3-Sepioteuthis lessoniana, C6-Sepia esculenta, O2-Amphioctopus aegina, S1-Loliolus uyii, S3-Uroteuthis chinensis, S4-Uroteuthis edulis [file peerj-09-11825-s002.zip › _Preprocessing_Upper_Beak/C3/U-l-C3-2.jpg]

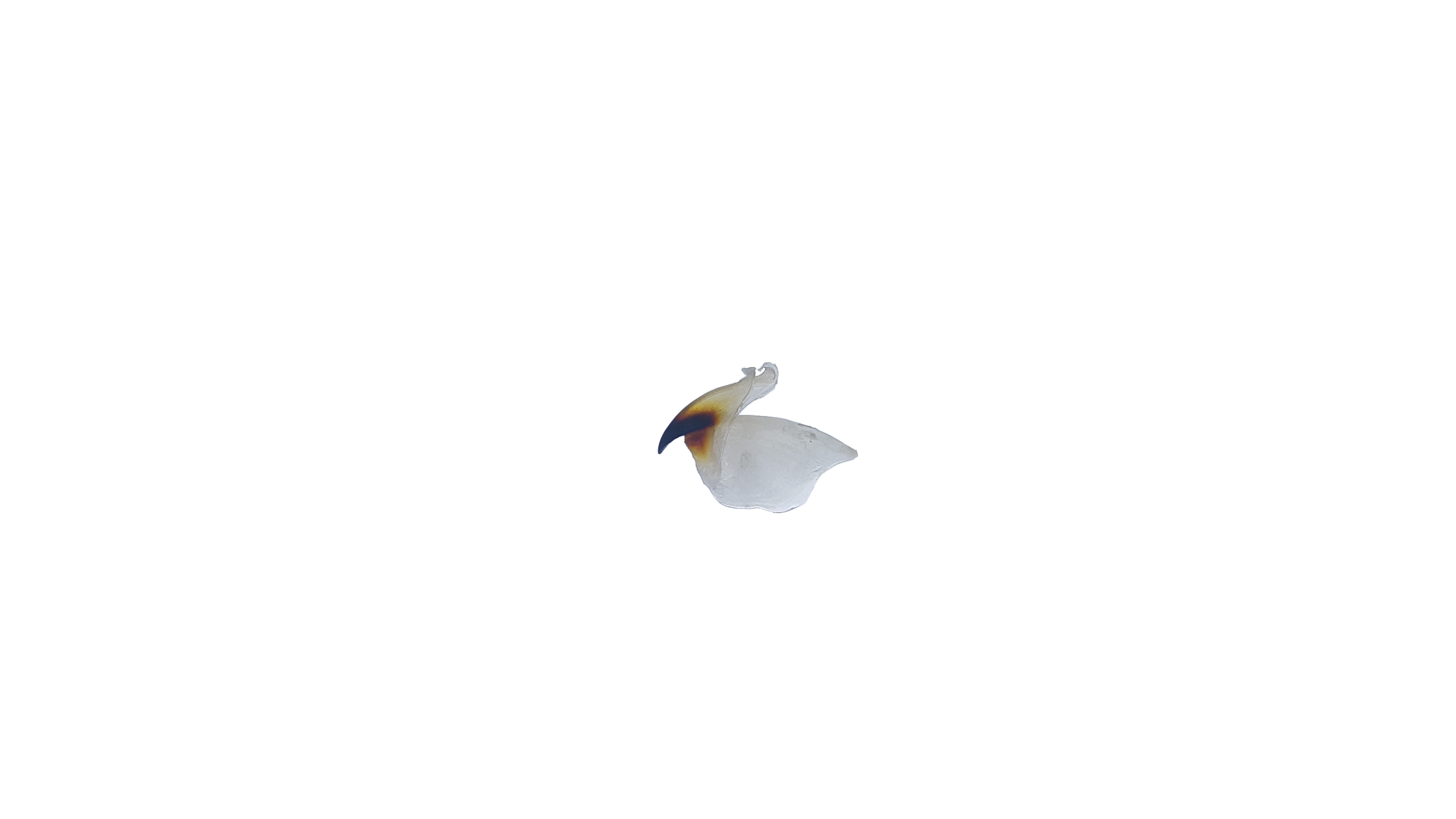

Supplement: Supplemental Information 2 — C2-Sepia aculeata, C3-Sepioteuthis lessoniana, C6-Sepia esculenta, O2-Amphioctopus aegina, S1-Loliolus uyii, S3-Uroteuthis chinensis, S4-Uroteuthis edulis [file peerj-09-11825-s002.zip › _Preprocessing_Upper_Beak/C3/U-l-C3-3.jpg]

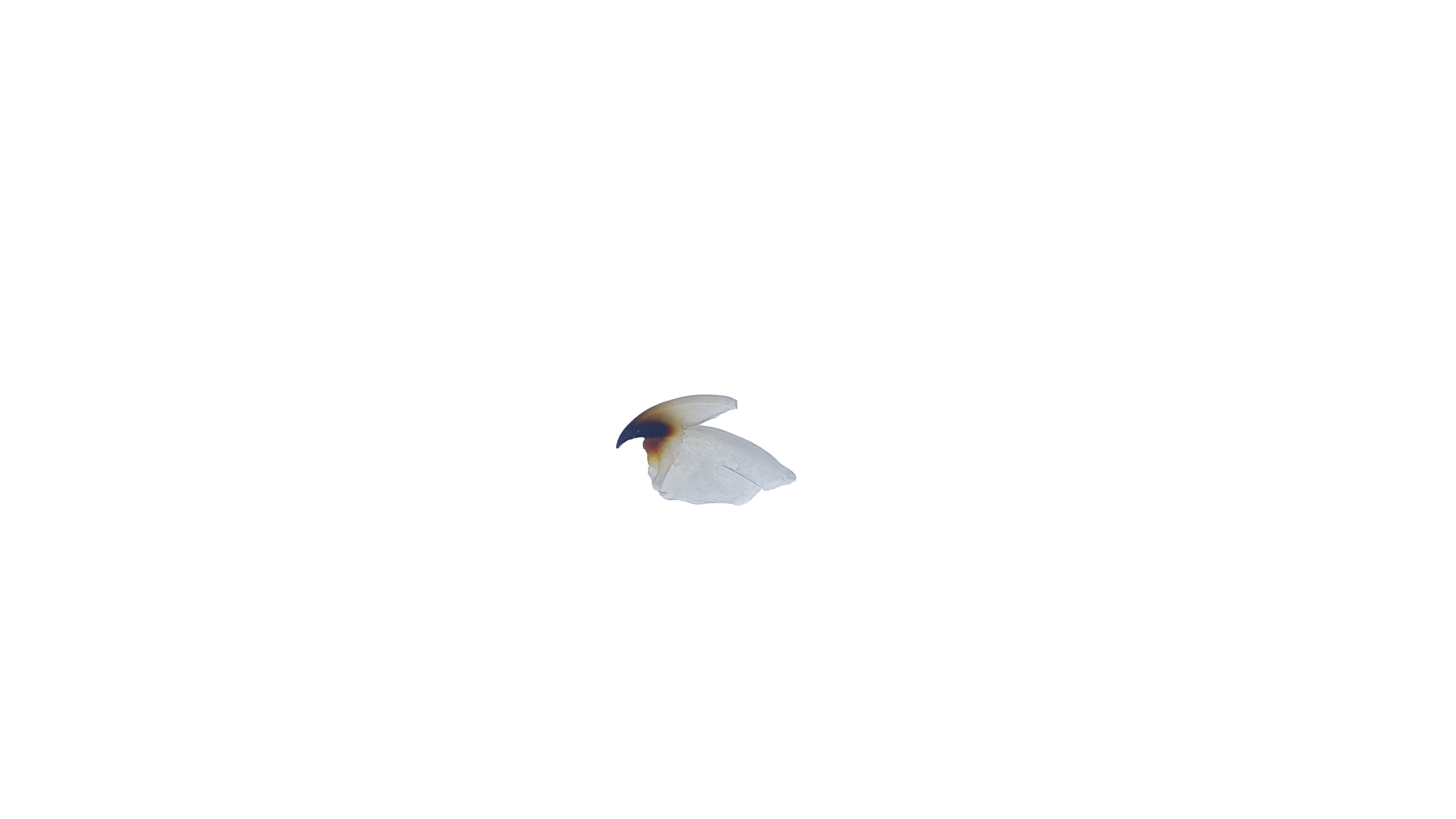

Supplement: Supplemental Information 2 — C2-Sepia aculeata, C3-Sepioteuthis lessoniana, C6-Sepia esculenta, O2-Amphioctopus aegina, S1-Loliolus uyii, S3-Uroteuthis chinensis, S4-Uroteuthis edulis [file peerj-09-11825-s002.zip › _Preprocessing_Upper_Beak/C3/U-l-C3-4.jpg]

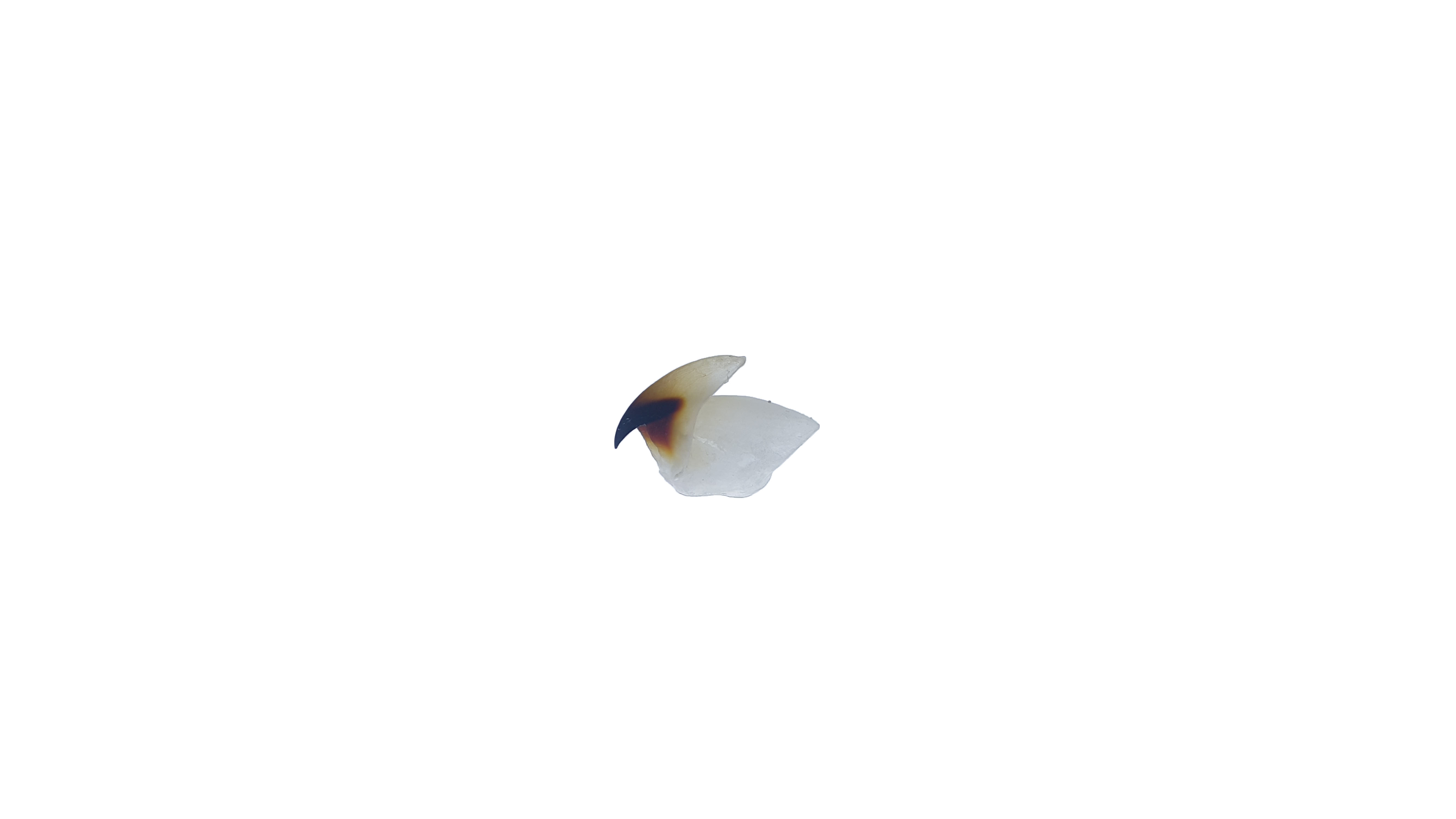

Supplement: Supplemental Information 2 — C2-Sepia aculeata, C3-Sepioteuthis lessoniana, C6-Sepia esculenta, O2-Amphioctopus aegina, S1-Loliolus uyii, S3-Uroteuthis chinensis, S4-Uroteuthis edulis [file peerj-09-11825-s002.zip › _Preprocessing_Upper_Beak/C3/U-l-C3-5.jpg]

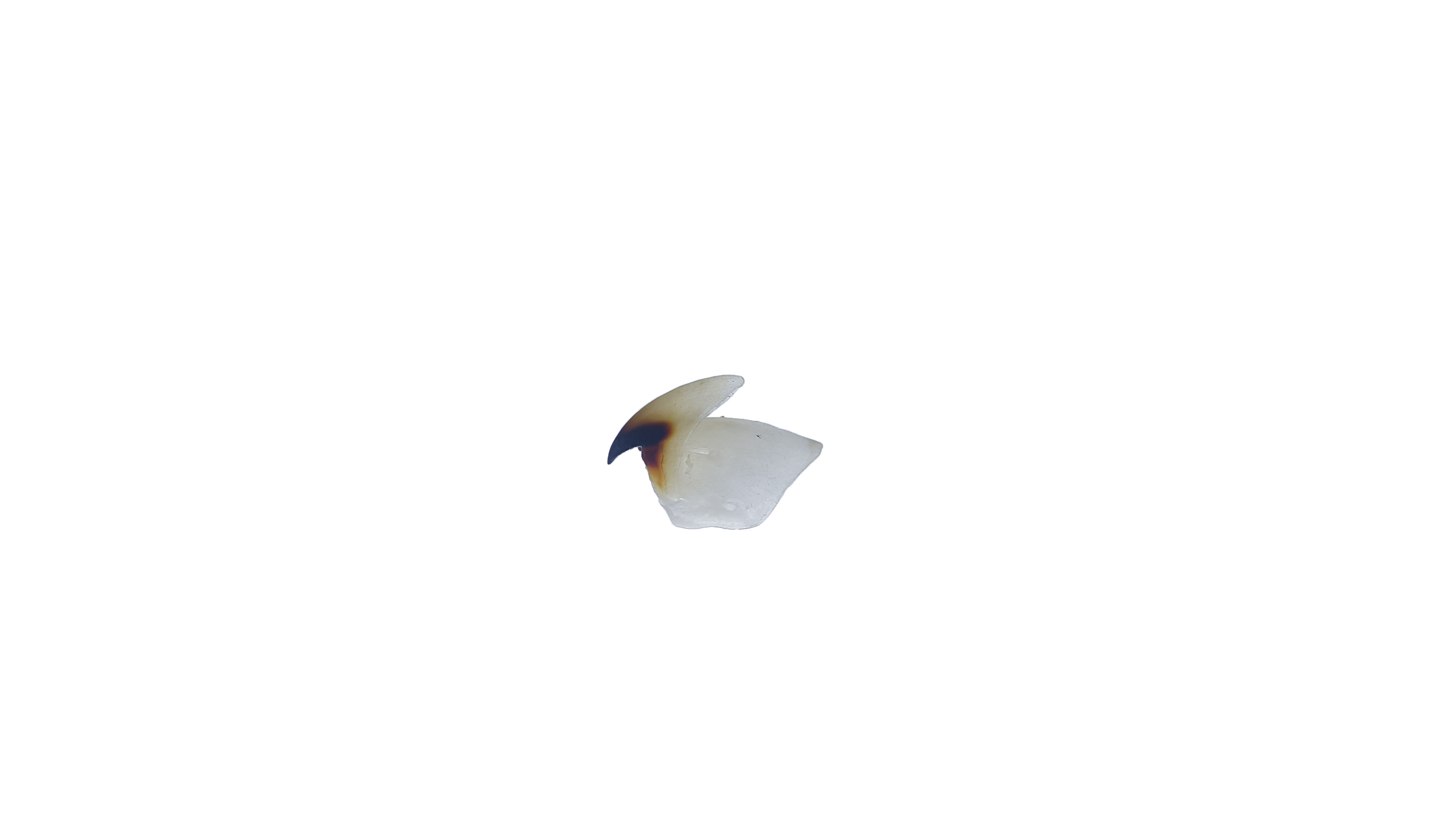

Supplement: Supplemental Information 2 — C2-Sepia aculeata, C3-Sepioteuthis lessoniana, C6-Sepia esculenta, O2-Amphioctopus aegina, S1-Loliolus uyii, S3-Uroteuthis chinensis, S4-Uroteuthis edulis [file peerj-09-11825-s002.zip › _Preprocessing_Upper_Beak/C3/U-l-C3-6.jpg]

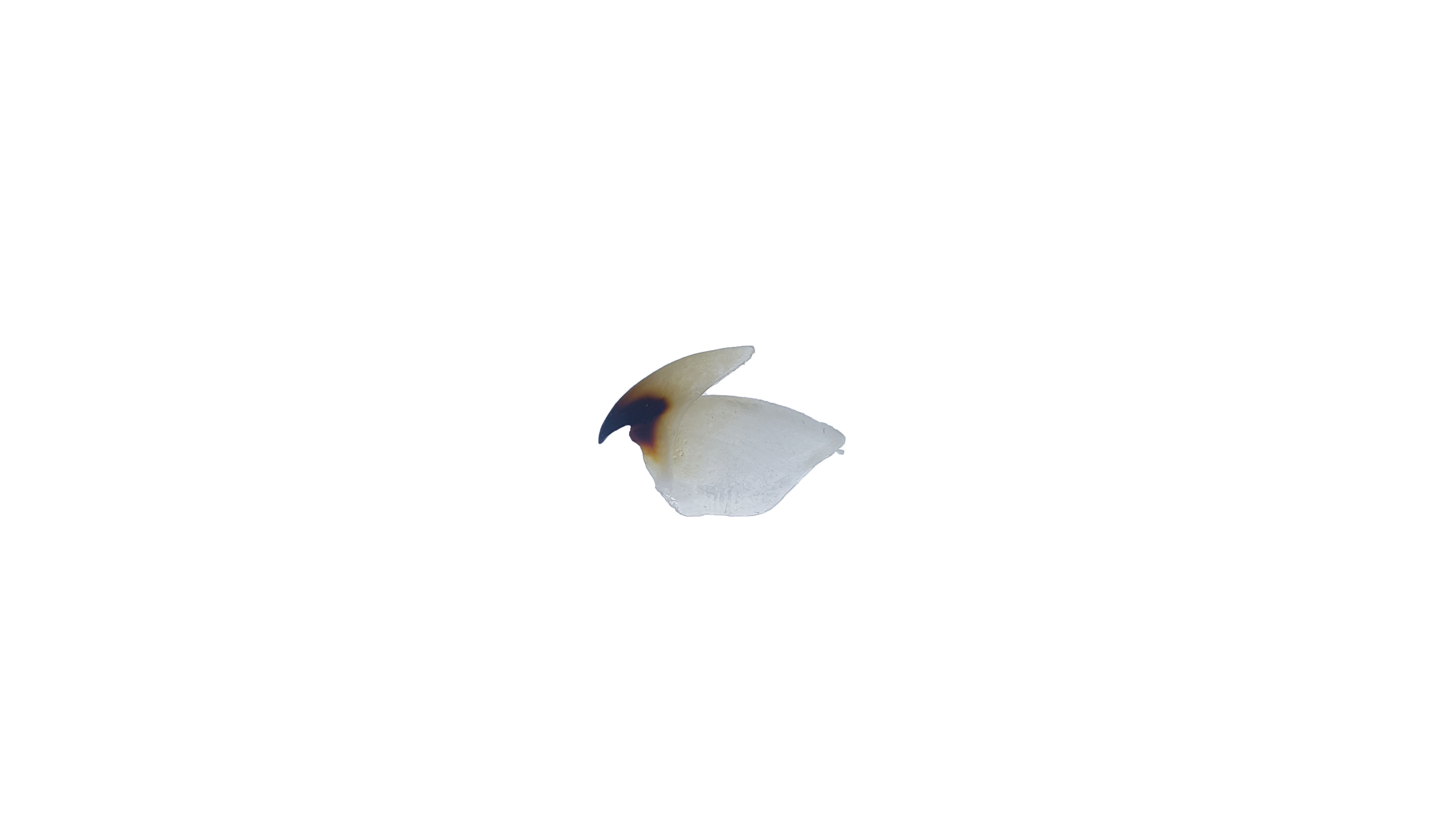

Supplement: Supplemental Information 2 — C2-Sepia aculeata, C3-Sepioteuthis lessoniana, C6-Sepia esculenta, O2-Amphioctopus aegina, S1-Loliolus uyii, S3-Uroteuthis chinensis, S4-Uroteuthis edulis [file peerj-09-11825-s002.zip › _Preprocessing_Upper_Beak/C3/U-l-C3-7.jpg]

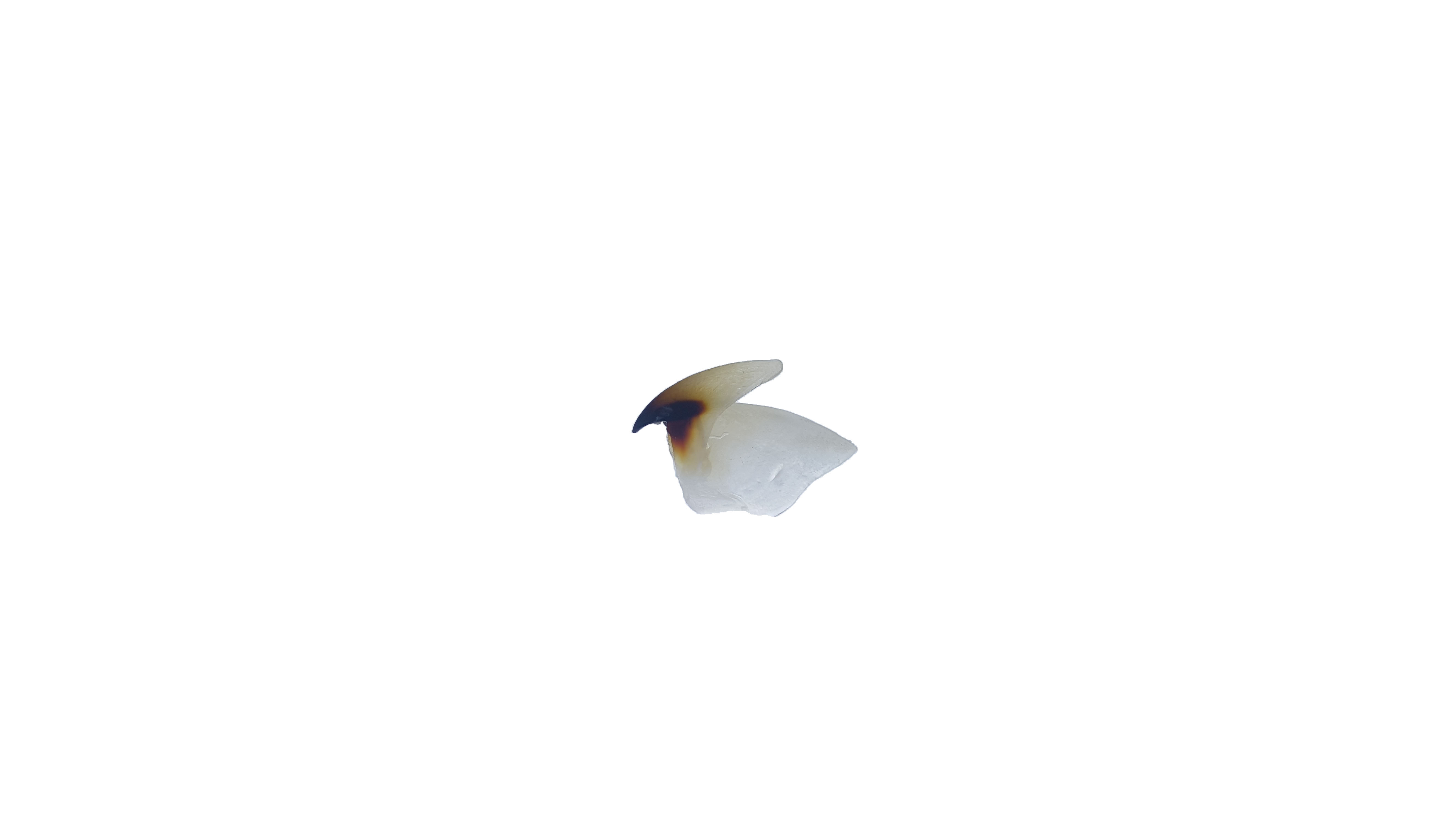

Supplement: Supplemental Information 2 — C2-Sepia aculeata, C3-Sepioteuthis lessoniana, C6-Sepia esculenta, O2-Amphioctopus aegina, S1-Loliolus uyii, S3-Uroteuthis chinensis, S4-Uroteuthis edulis [file peerj-09-11825-s002.zip › _Preprocessing_Upper_Beak/C3/U-l-C3-8.jpg]

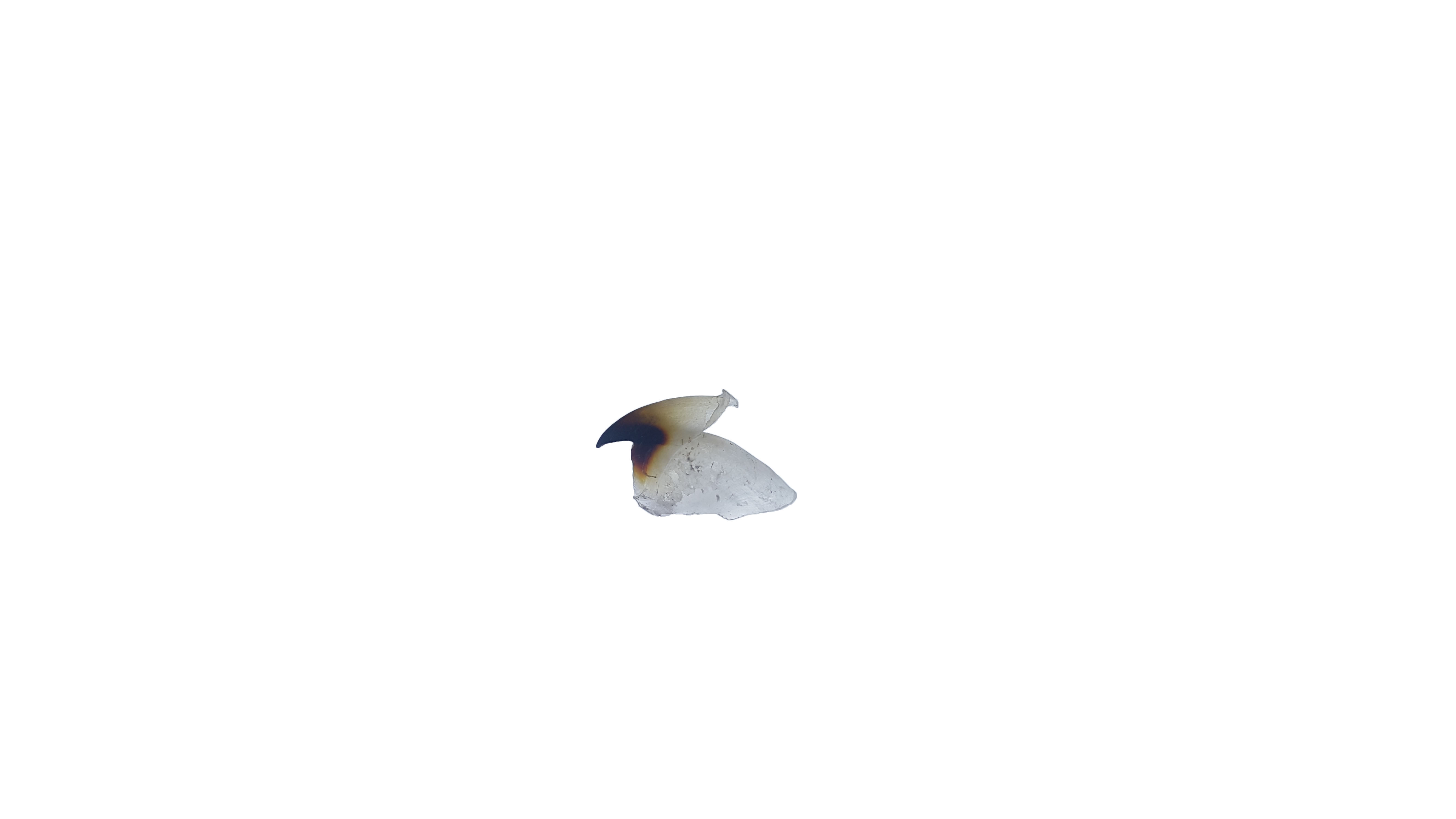

Supplement: Supplemental Information 2 — C2-Sepia aculeata, C3-Sepioteuthis lessoniana, C6-Sepia esculenta, O2-Amphioctopus aegina, S1-Loliolus uyii, S3-Uroteuthis chinensis, S4-Uroteuthis edulis [file peerj-09-11825-s002.zip › _Preprocessing_Upper_Beak/C3/U-l-C3-9.jpg]

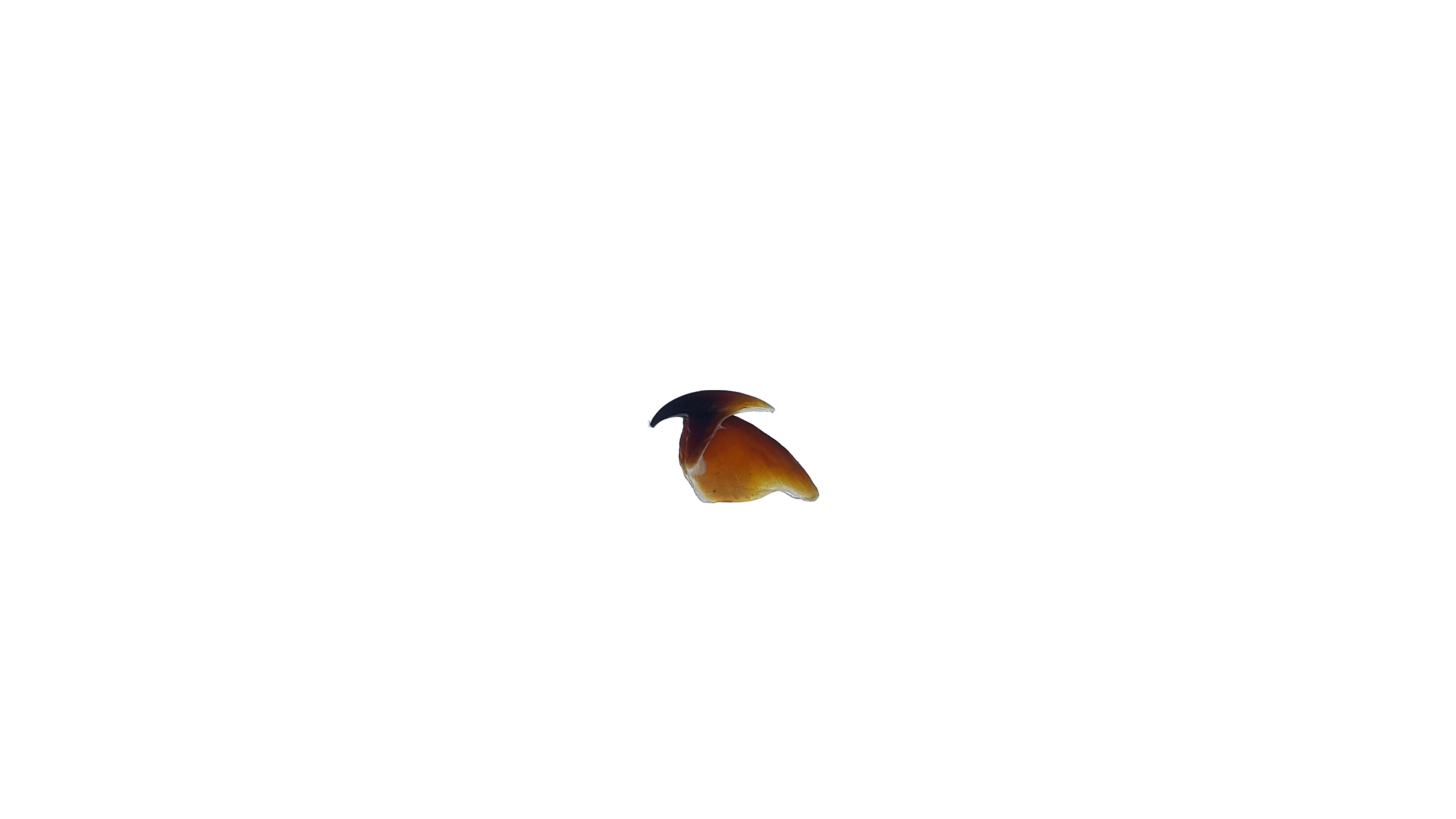

Supplement: Supplemental Information 2 — C2-Sepia aculeata, C3-Sepioteuthis lessoniana, C6-Sepia esculenta, O2-Amphioctopus aegina, S1-Loliolus uyii, S3-Uroteuthis chinensis, S4-Uroteuthis edulis [file peerj-09-11825-s002.zip › _Preprocessing_Upper_Beak/C6/U-l-C6-1.jpg]

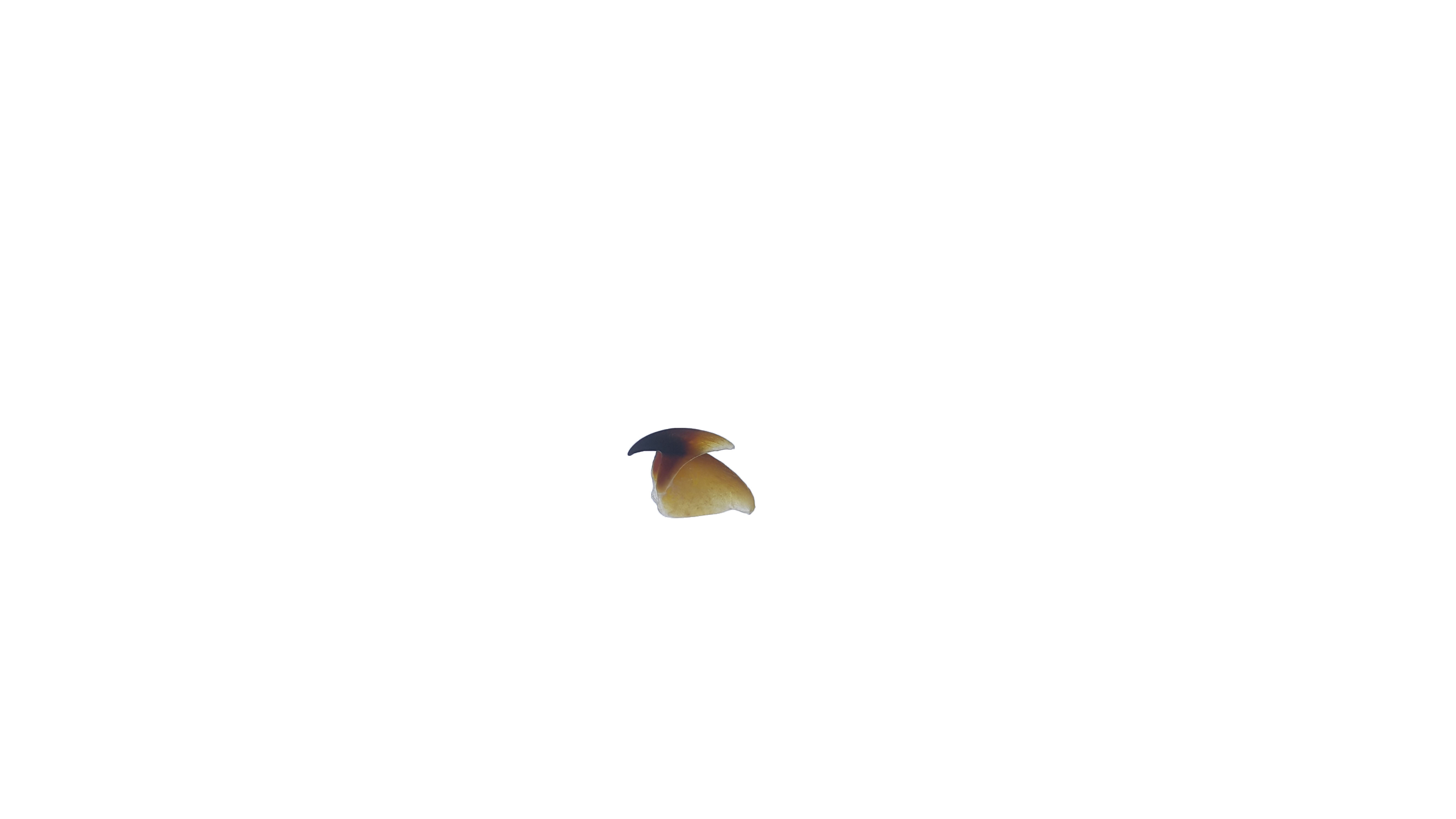

Supplement: Supplemental Information 2 — C2-Sepia aculeata, C3-Sepioteuthis lessoniana, C6-Sepia esculenta, O2-Amphioctopus aegina, S1-Loliolus uyii, S3-Uroteuthis chinensis, S4-Uroteuthis edulis [file peerj-09-11825-s002.zip › _Preprocessing_Upper_Beak/C6/U-l-C6-10.jpg]

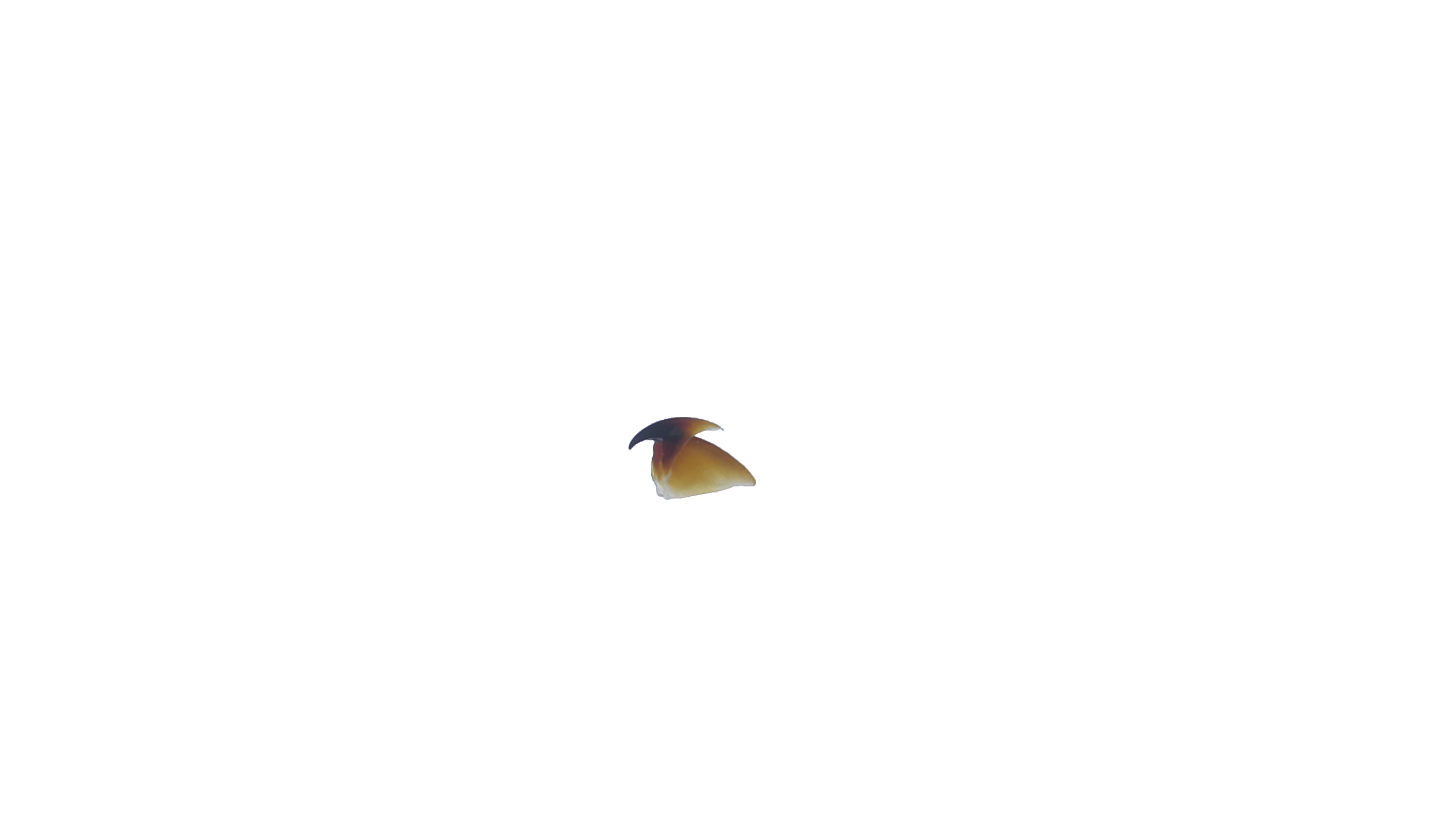

Supplement: Supplemental Information 2 — C2-Sepia aculeata, C3-Sepioteuthis lessoniana, C6-Sepia esculenta, O2-Amphioctopus aegina, S1-Loliolus uyii, S3-Uroteuthis chinensis, S4-Uroteuthis edulis [file peerj-09-11825-s002.zip › _Preprocessing_Upper_Beak/C6/U-l-C6-11.jpg]

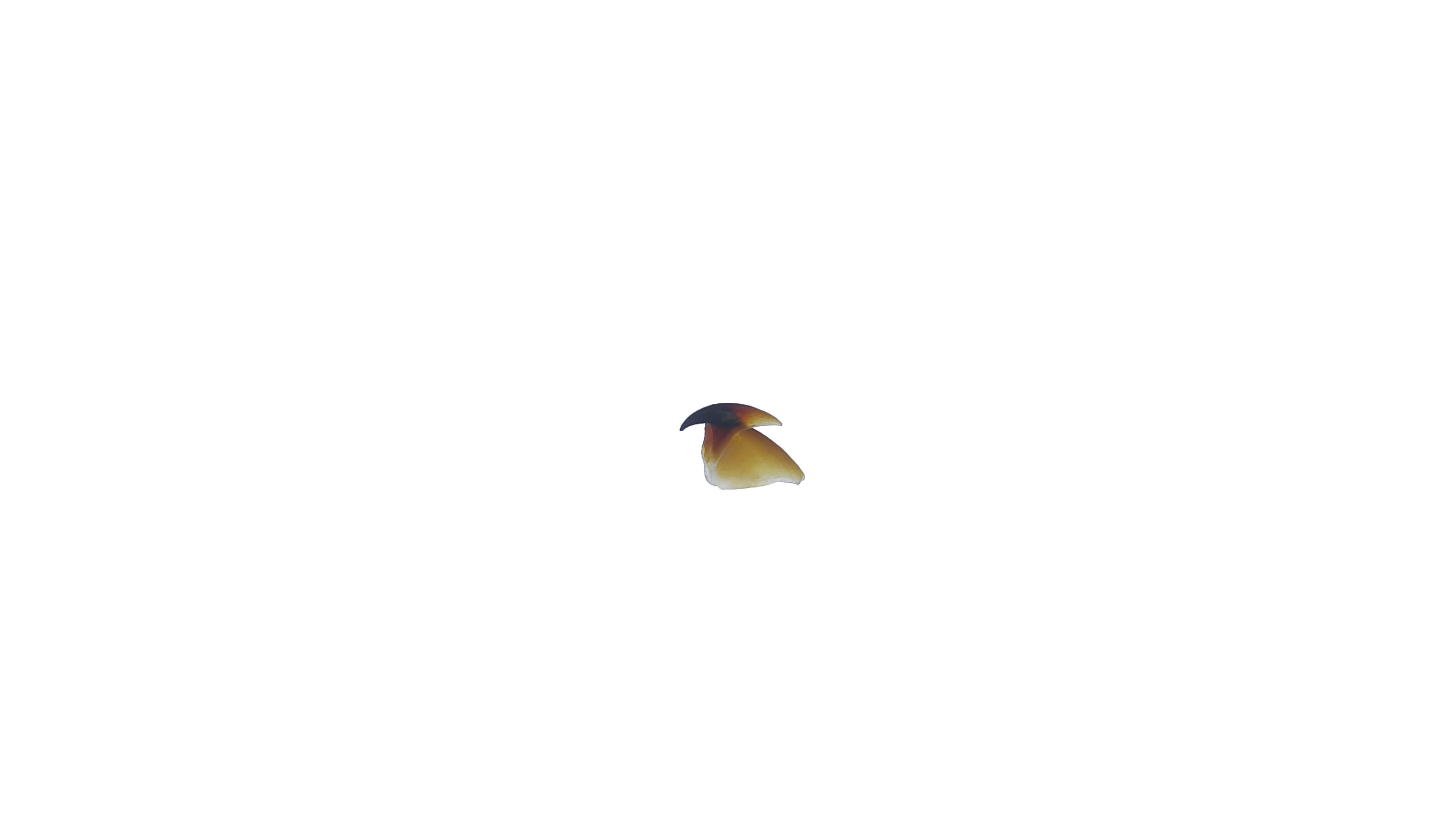

Supplement: Supplemental Information 2 — C2-Sepia aculeata, C3-Sepioteuthis lessoniana, C6-Sepia esculenta, O2-Amphioctopus aegina, S1-Loliolus uyii, S3-Uroteuthis chinensis, S4-Uroteuthis edulis [file peerj-09-11825-s002.zip › _Preprocessing_Upper_Beak/C6/U-l-C6-12.jpg]

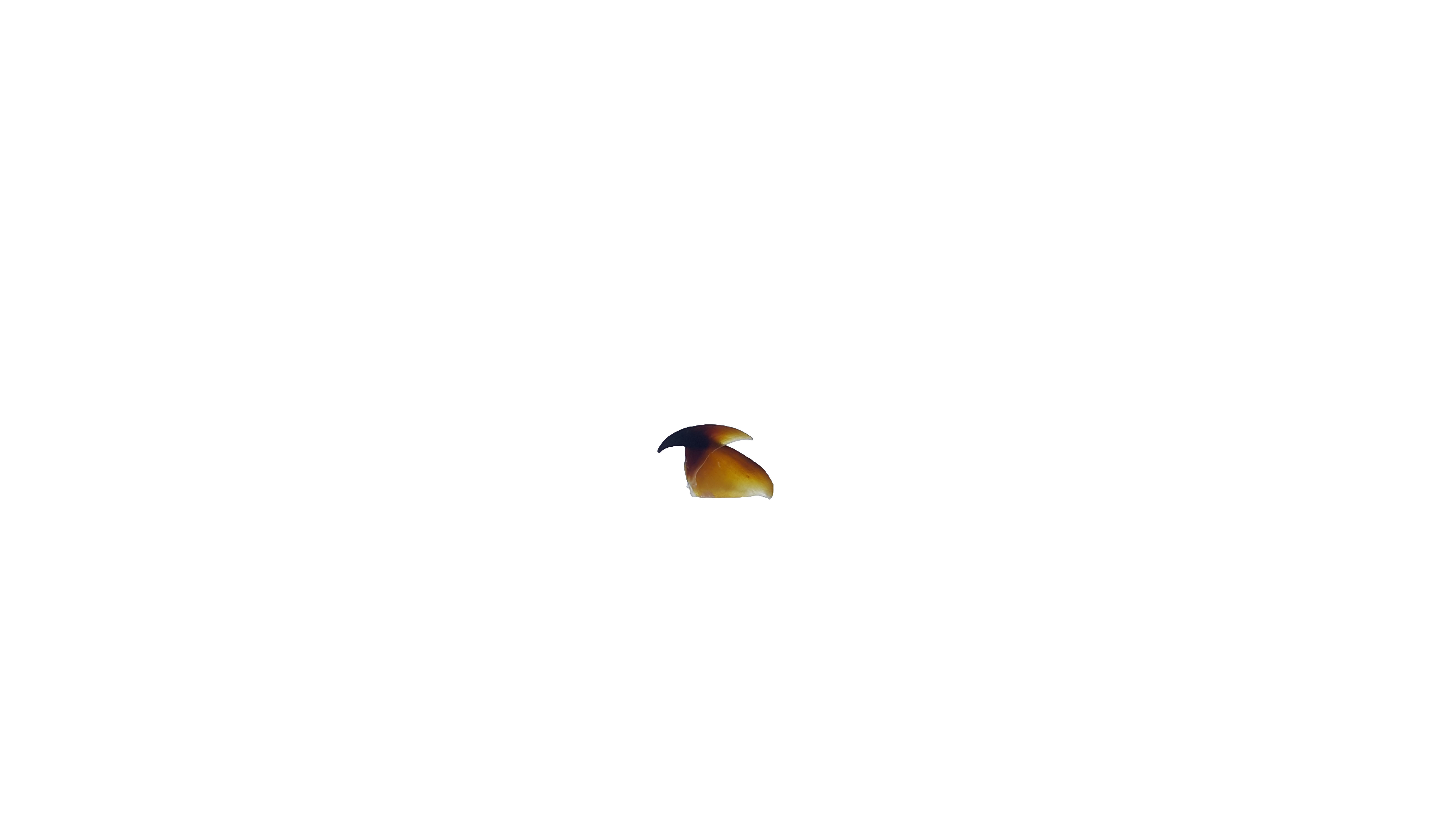

Supplement: Supplemental Information 2 — C2-Sepia aculeata, C3-Sepioteuthis lessoniana, C6-Sepia esculenta, O2-Amphioctopus aegina, S1-Loliolus uyii, S3-Uroteuthis chinensis, S4-Uroteuthis edulis [file peerj-09-11825-s002.zip › _Preprocessing_Upper_Beak/C6/U-l-C6-13.jpg]

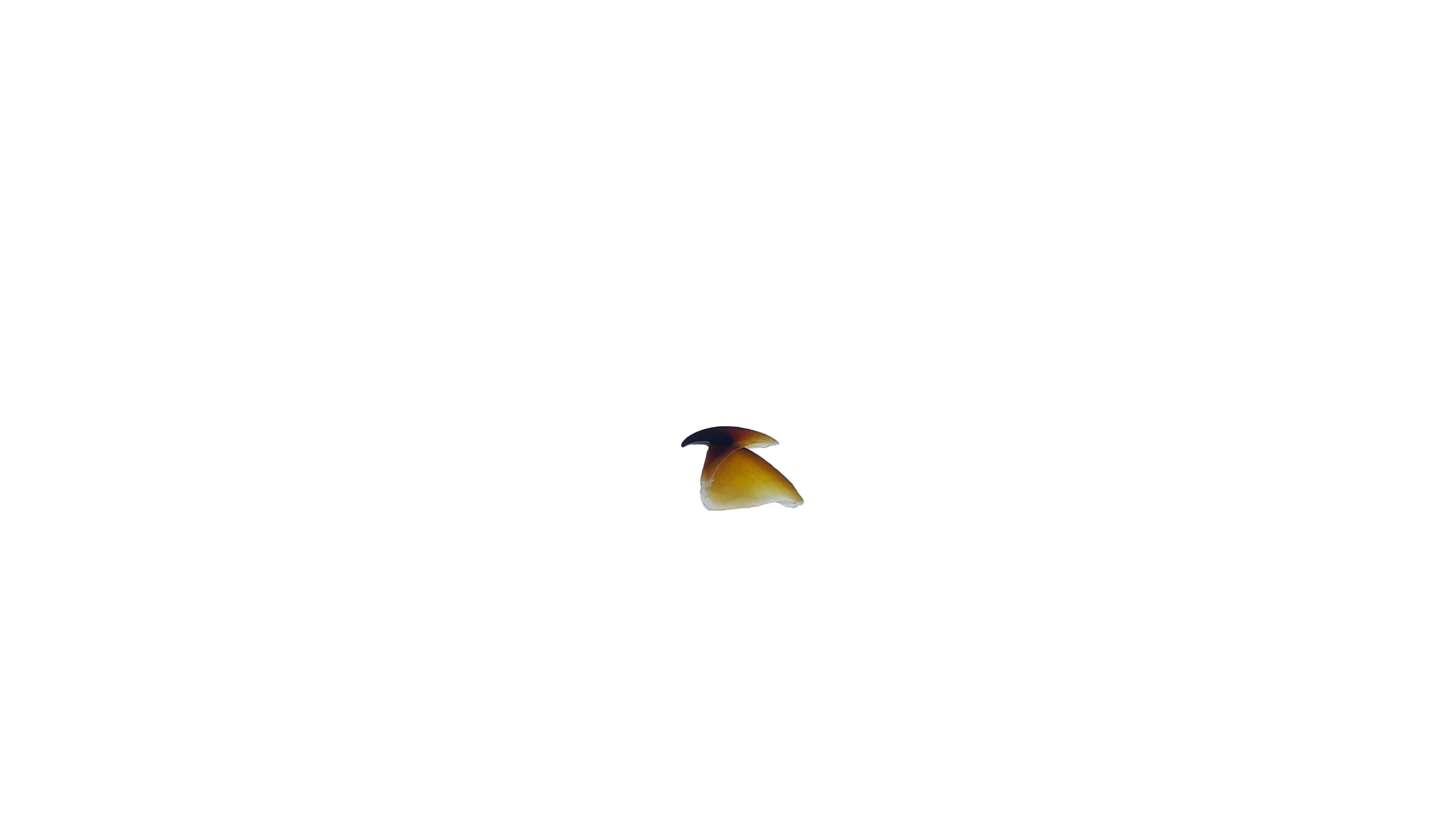

Supplement: Supplemental Information 2 — C2-Sepia aculeata, C3-Sepioteuthis lessoniana, C6-Sepia esculenta, O2-Amphioctopus aegina, S1-Loliolus uyii, S3-Uroteuthis chinensis, S4-Uroteuthis edulis [file peerj-09-11825-s002.zip › _Preprocessing_Upper_Beak/C6/U-l-C6-14.jpg]

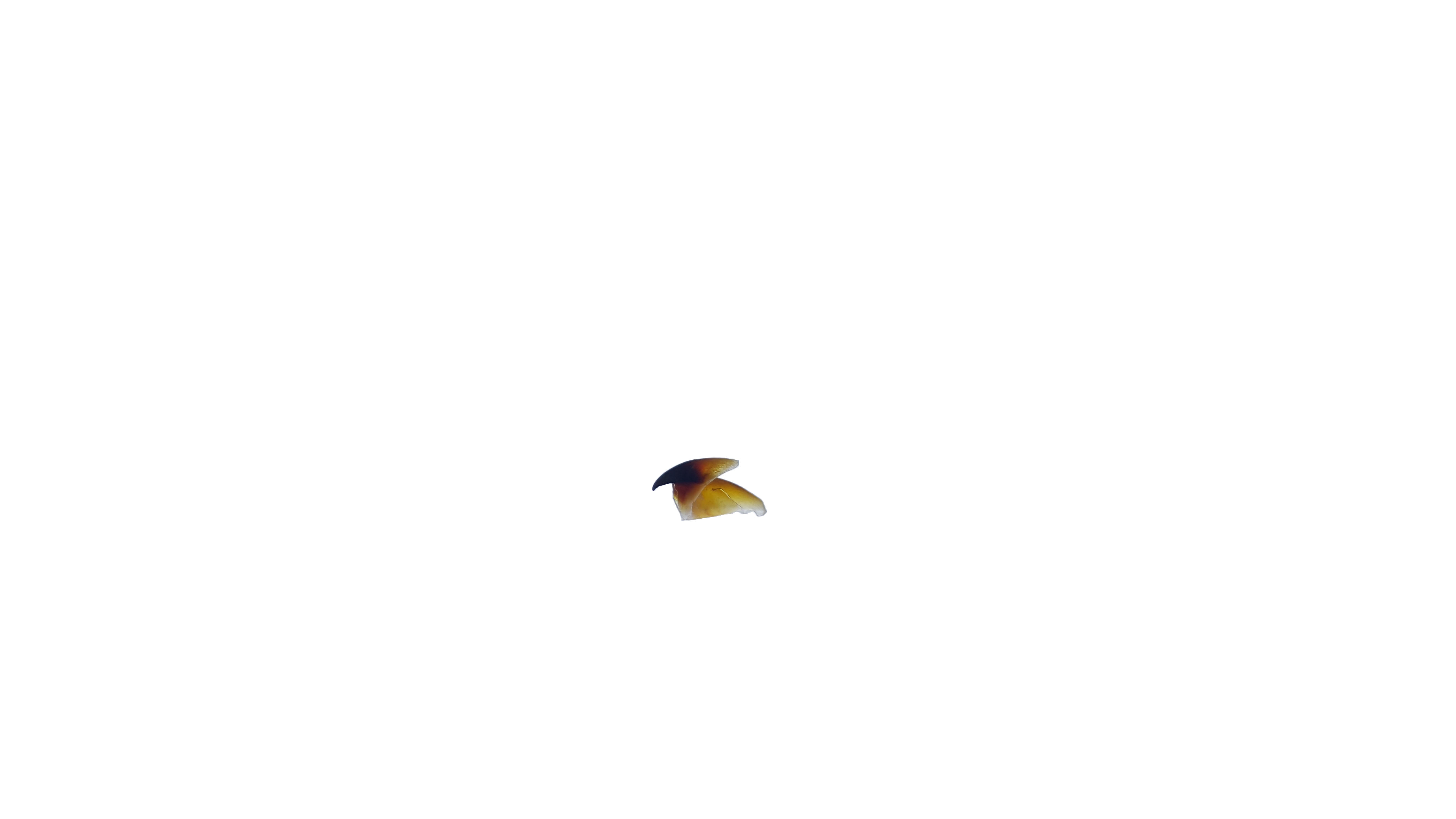

Supplement: Supplemental Information 2 — C2-Sepia aculeata, C3-Sepioteuthis lessoniana, C6-Sepia esculenta, O2-Amphioctopus aegina, S1-Loliolus uyii, S3-Uroteuthis chinensis, S4-Uroteuthis edulis [file peerj-09-11825-s002.zip › _Preprocessing_Upper_Beak/C6/U-l-C6-15.jpg]

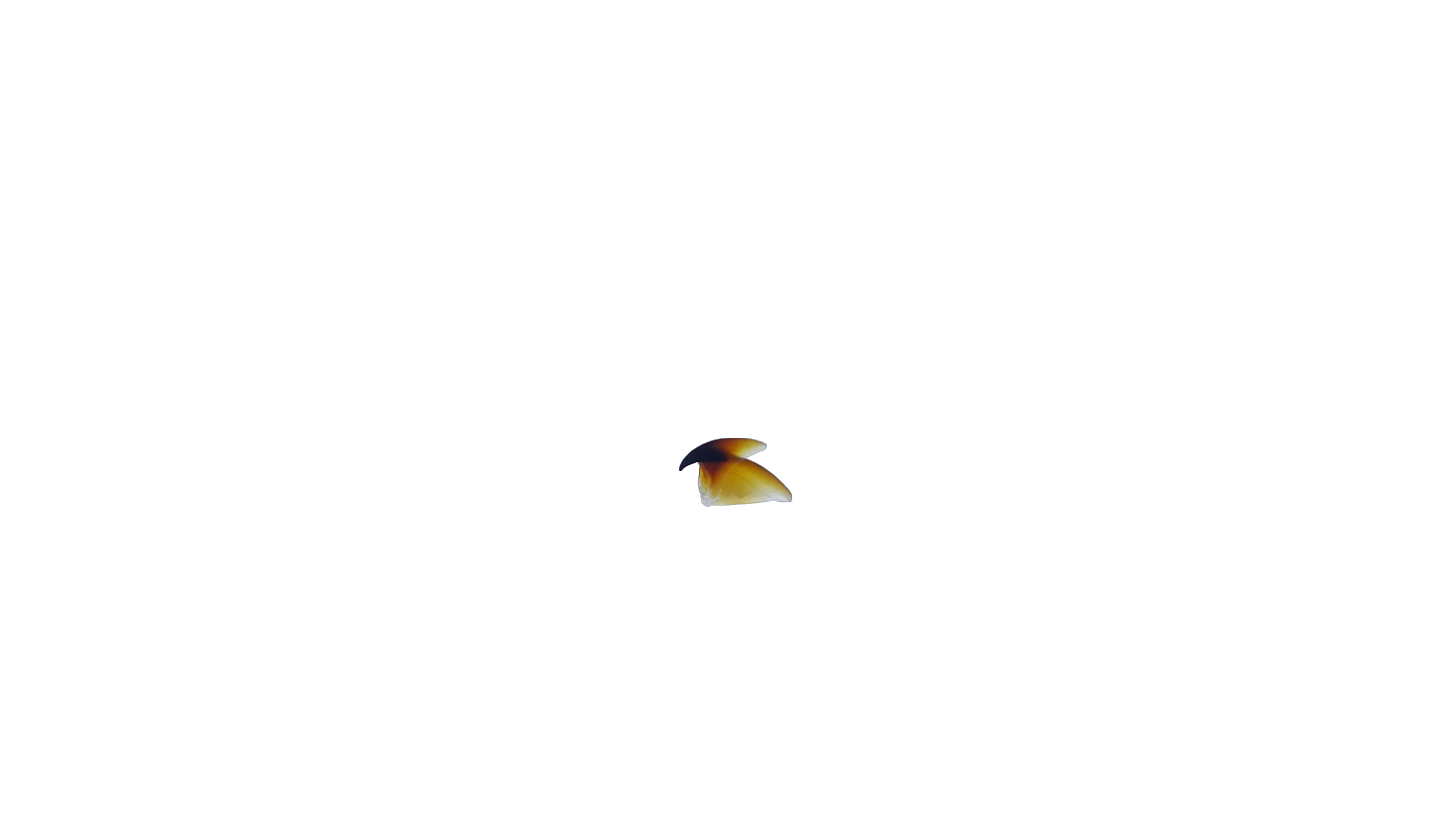

Supplement: Supplemental Information 2 — C2-Sepia aculeata, C3-Sepioteuthis lessoniana, C6-Sepia esculenta, O2-Amphioctopus aegina, S1-Loliolus uyii, S3-Uroteuthis chinensis, S4-Uroteuthis edulis [file peerj-09-11825-s002.zip › _Preprocessing_Upper_Beak/C6/U-l-C6-16.jpg]

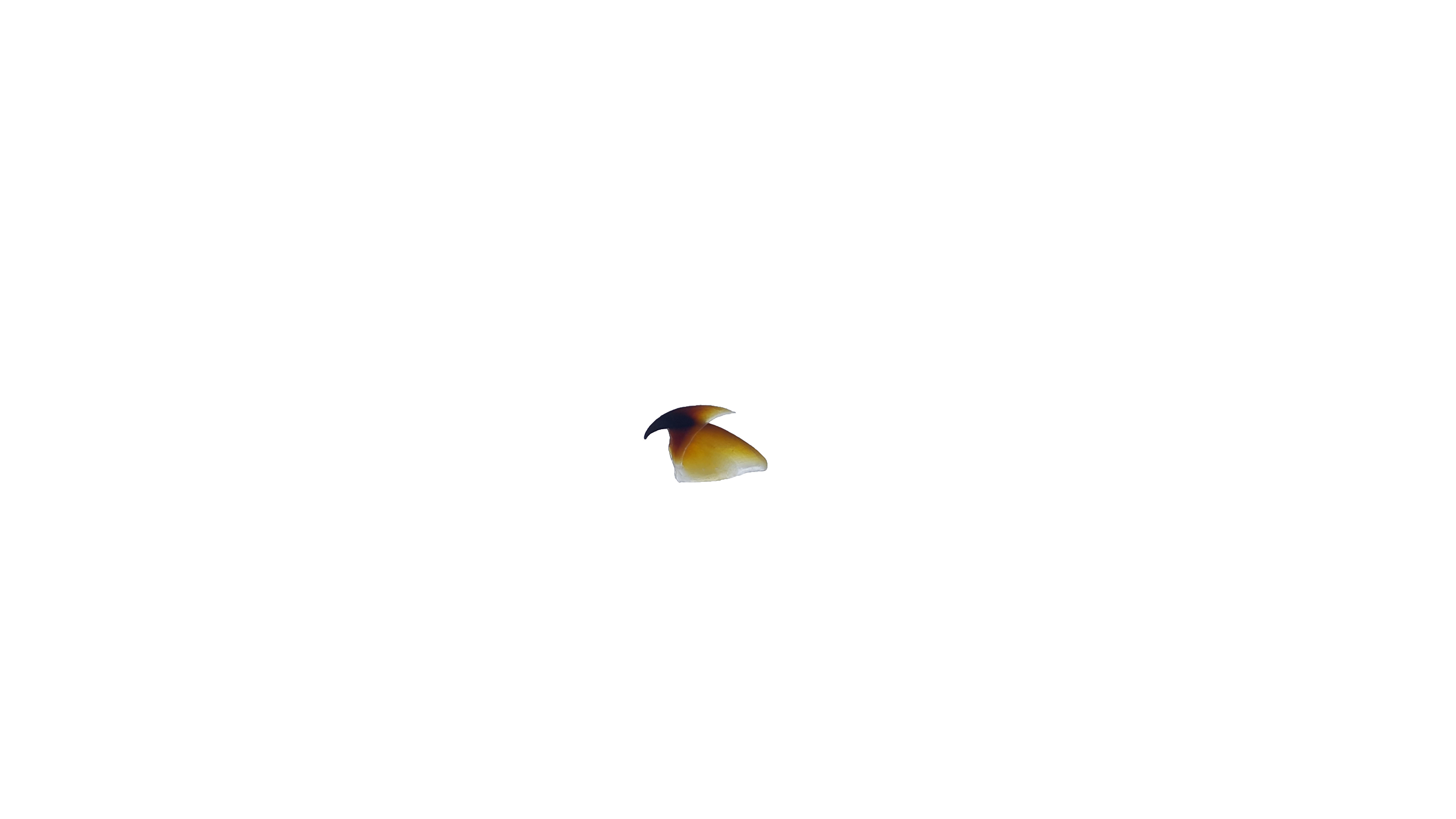

Supplement: Supplemental Information 2 — C2-Sepia aculeata, C3-Sepioteuthis lessoniana, C6-Sepia esculenta, O2-Amphioctopus aegina, S1-Loliolus uyii, S3-Uroteuthis chinensis, S4-Uroteuthis edulis [file peerj-09-11825-s002.zip › _Preprocessing_Upper_Beak/C6/U-l-C6-17.jpg]

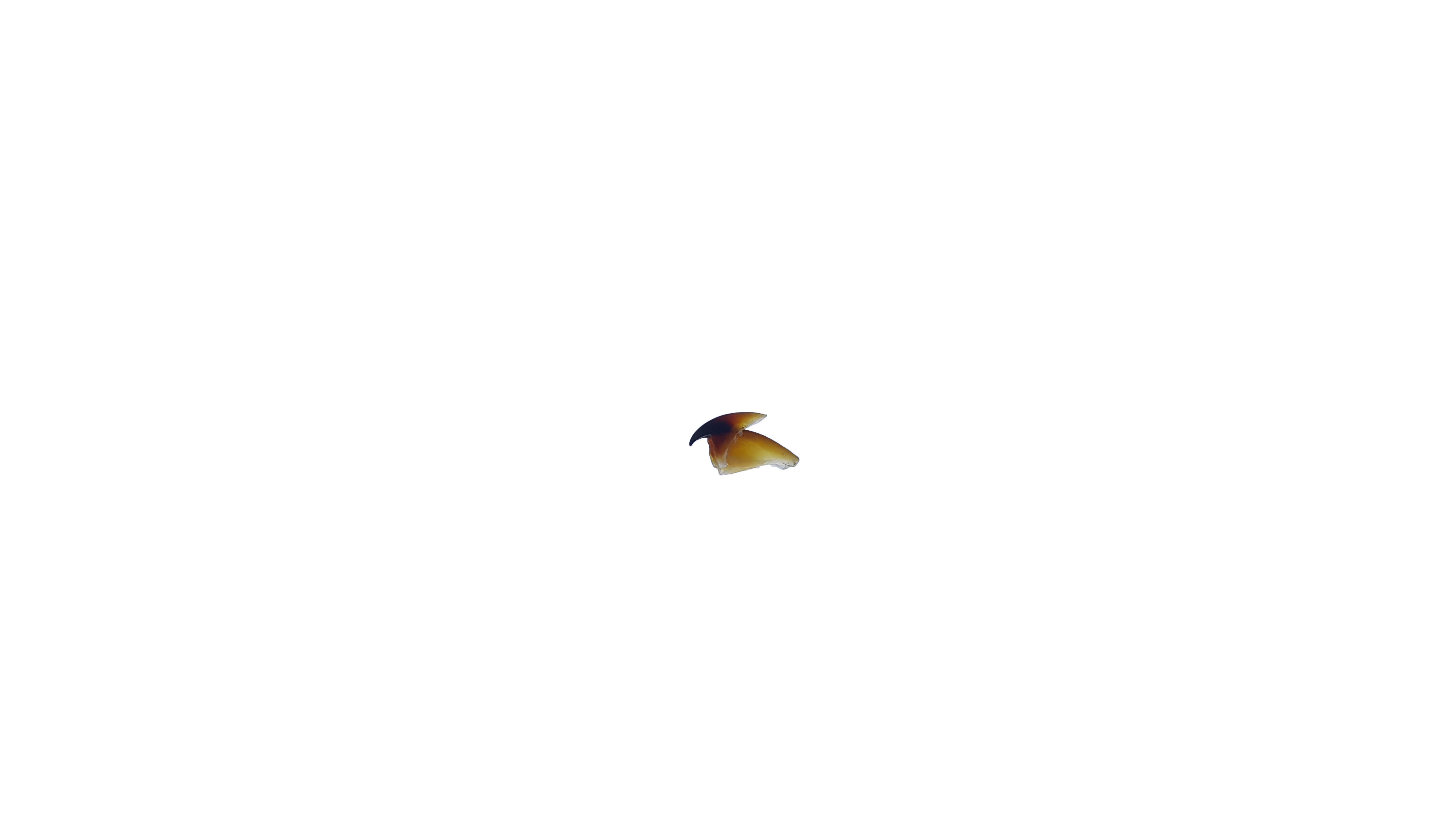

Supplement: Supplemental Information 2 — C2-Sepia aculeata, C3-Sepioteuthis lessoniana, C6-Sepia esculenta, O2-Amphioctopus aegina, S1-Loliolus uyii, S3-Uroteuthis chinensis, S4-Uroteuthis edulis [file peerj-09-11825-s002.zip › _Preprocessing_Upper_Beak/C6/U-l-C6-18.jpg]

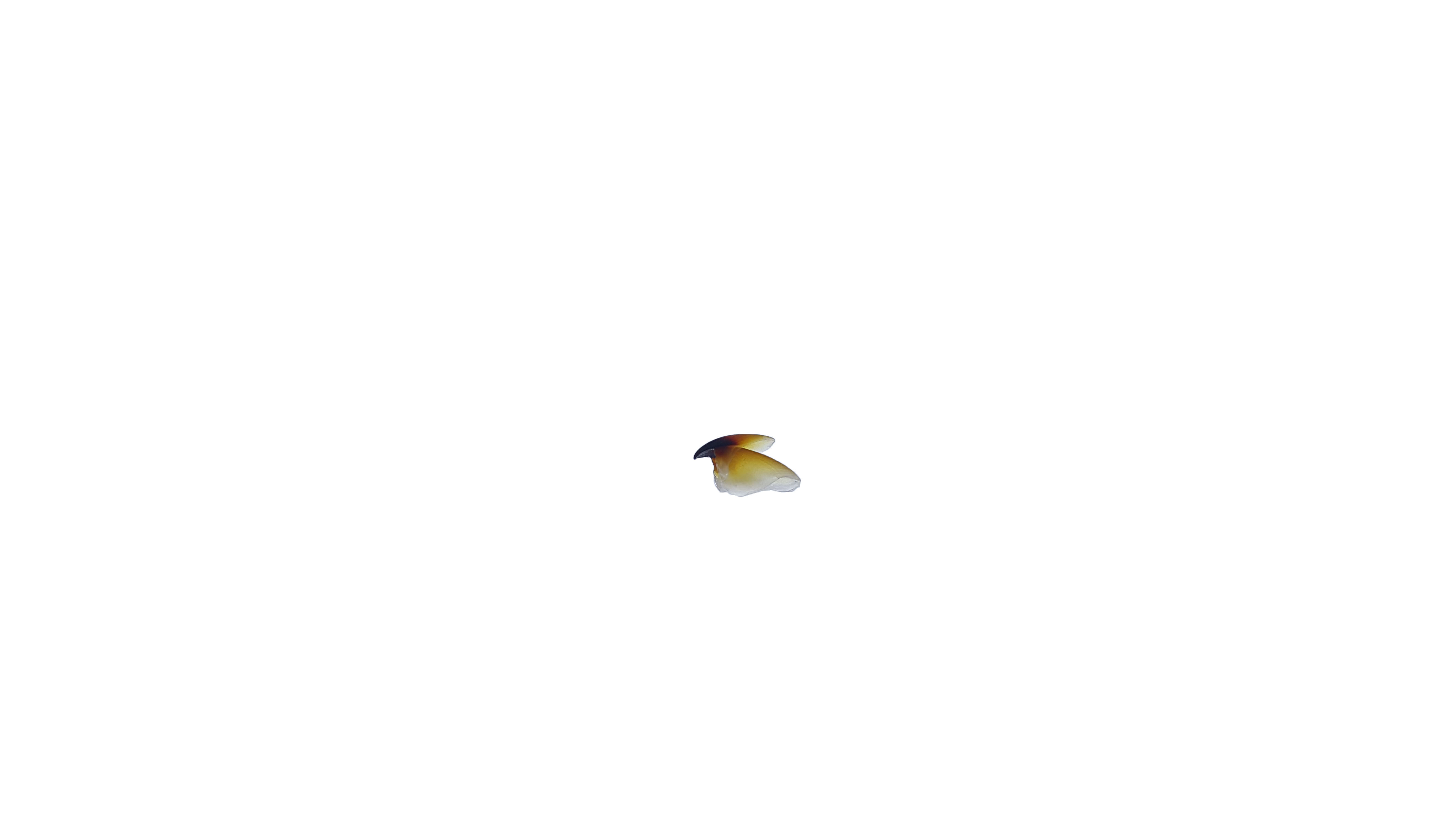

Supplement: Supplemental Information 2 — C2-Sepia aculeata, C3-Sepioteuthis lessoniana, C6-Sepia esculenta, O2-Amphioctopus aegina, S1-Loliolus uyii, S3-Uroteuthis chinensis, S4-Uroteuthis edulis [file peerj-09-11825-s002.zip › _Preprocessing_Upper_Beak/C6/U-l-C6-19.jpg]

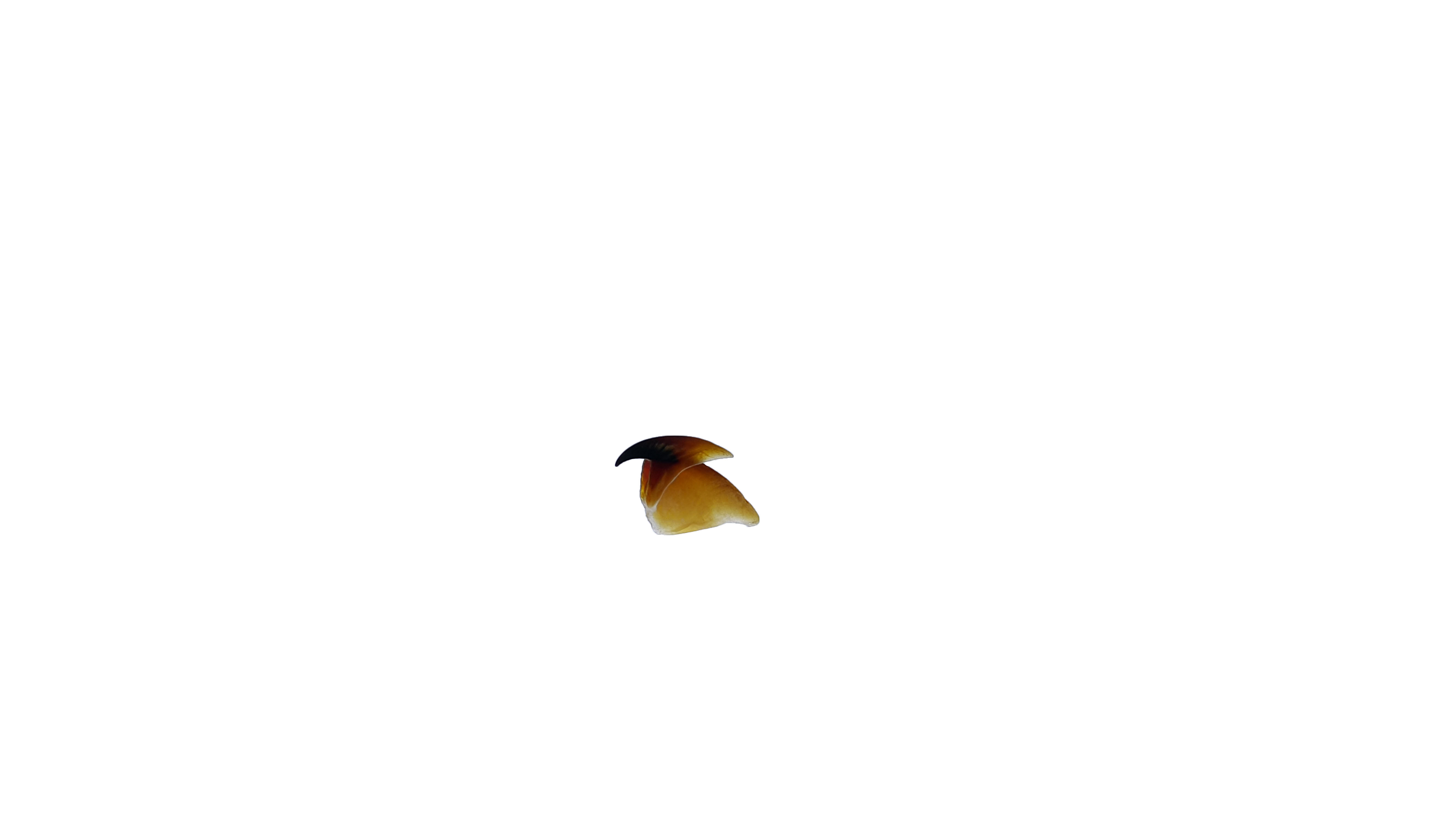

Supplement: Supplemental Information 2 — C2-Sepia aculeata, C3-Sepioteuthis lessoniana, C6-Sepia esculenta, O2-Amphioctopus aegina, S1-Loliolus uyii, S3-Uroteuthis chinensis, S4-Uroteuthis edulis [file peerj-09-11825-s002.zip › _Preprocessing_Upper_Beak/C6/U-l-C6-2.jpg]

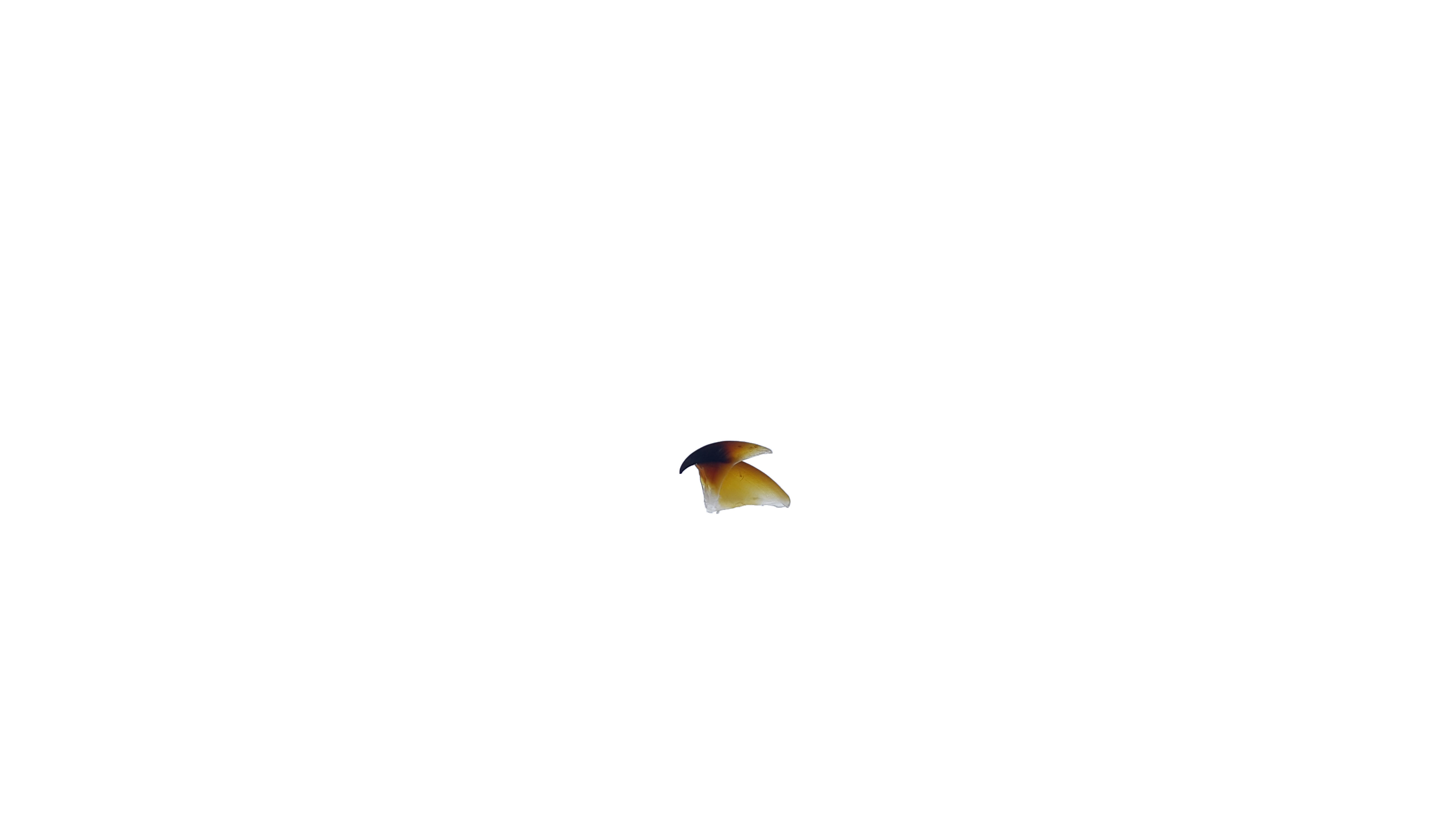

Supplement: Supplemental Information 2 — C2-Sepia aculeata, C3-Sepioteuthis lessoniana, C6-Sepia esculenta, O2-Amphioctopus aegina, S1-Loliolus uyii, S3-Uroteuthis chinensis, S4-Uroteuthis edulis [file peerj-09-11825-s002.zip › _Preprocessing_Upper_Beak/C6/U-l-C6-20.jpg]

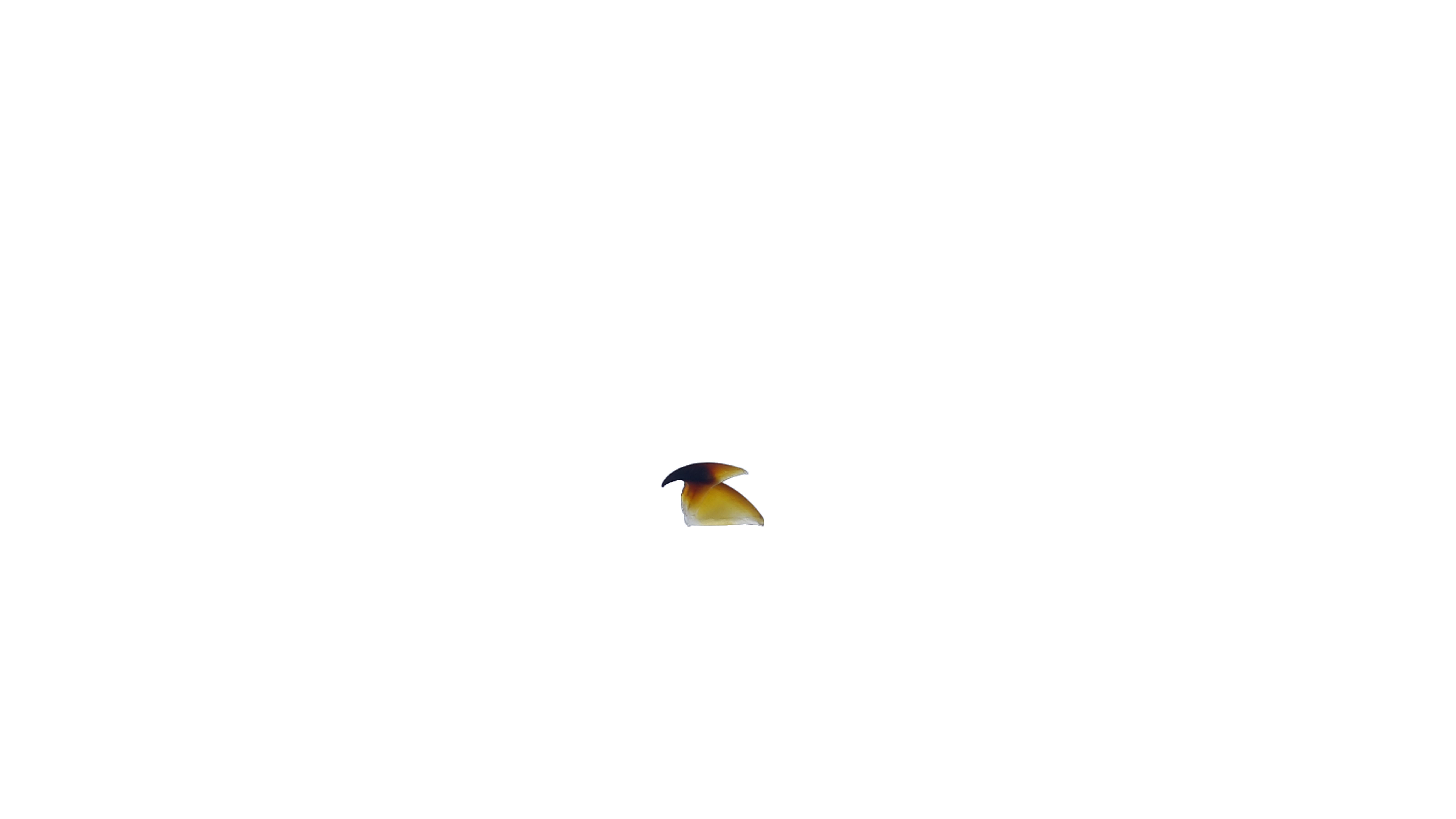

Supplement: Supplemental Information 2 — C2-Sepia aculeata, C3-Sepioteuthis lessoniana, C6-Sepia esculenta, O2-Amphioctopus aegina, S1-Loliolus uyii, S3-Uroteuthis chinensis, S4-Uroteuthis edulis [file peerj-09-11825-s002.zip › _Preprocessing_Upper_Beak/C6/U-l-C6-21.jpg]

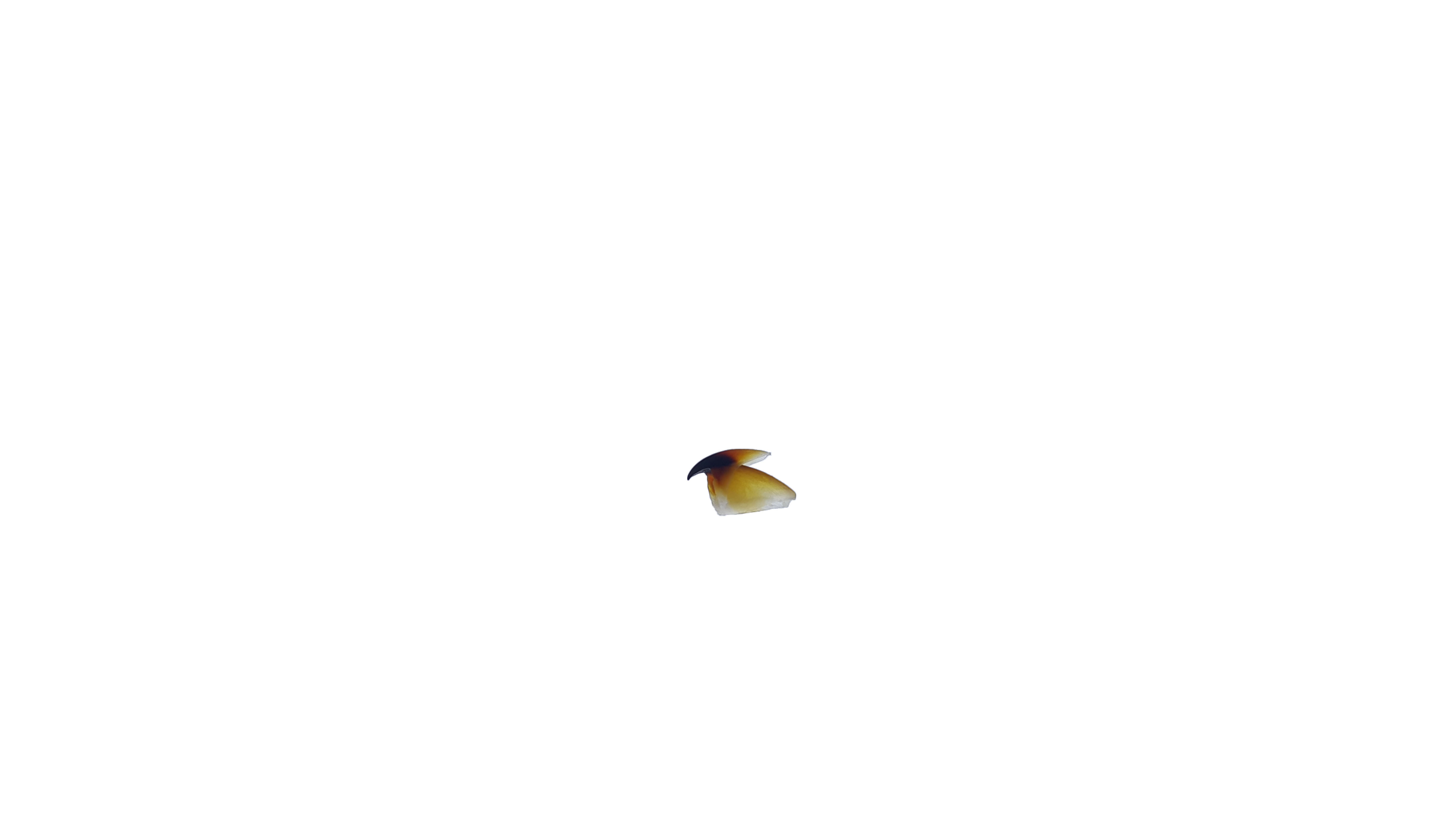

Supplement: Supplemental Information 2 — C2-Sepia aculeata, C3-Sepioteuthis lessoniana, C6-Sepia esculenta, O2-Amphioctopus aegina, S1-Loliolus uyii, S3-Uroteuthis chinensis, S4-Uroteuthis edulis [file peerj-09-11825-s002.zip › _Preprocessing_Upper_Beak/C6/U-l-C6-22.jpg]

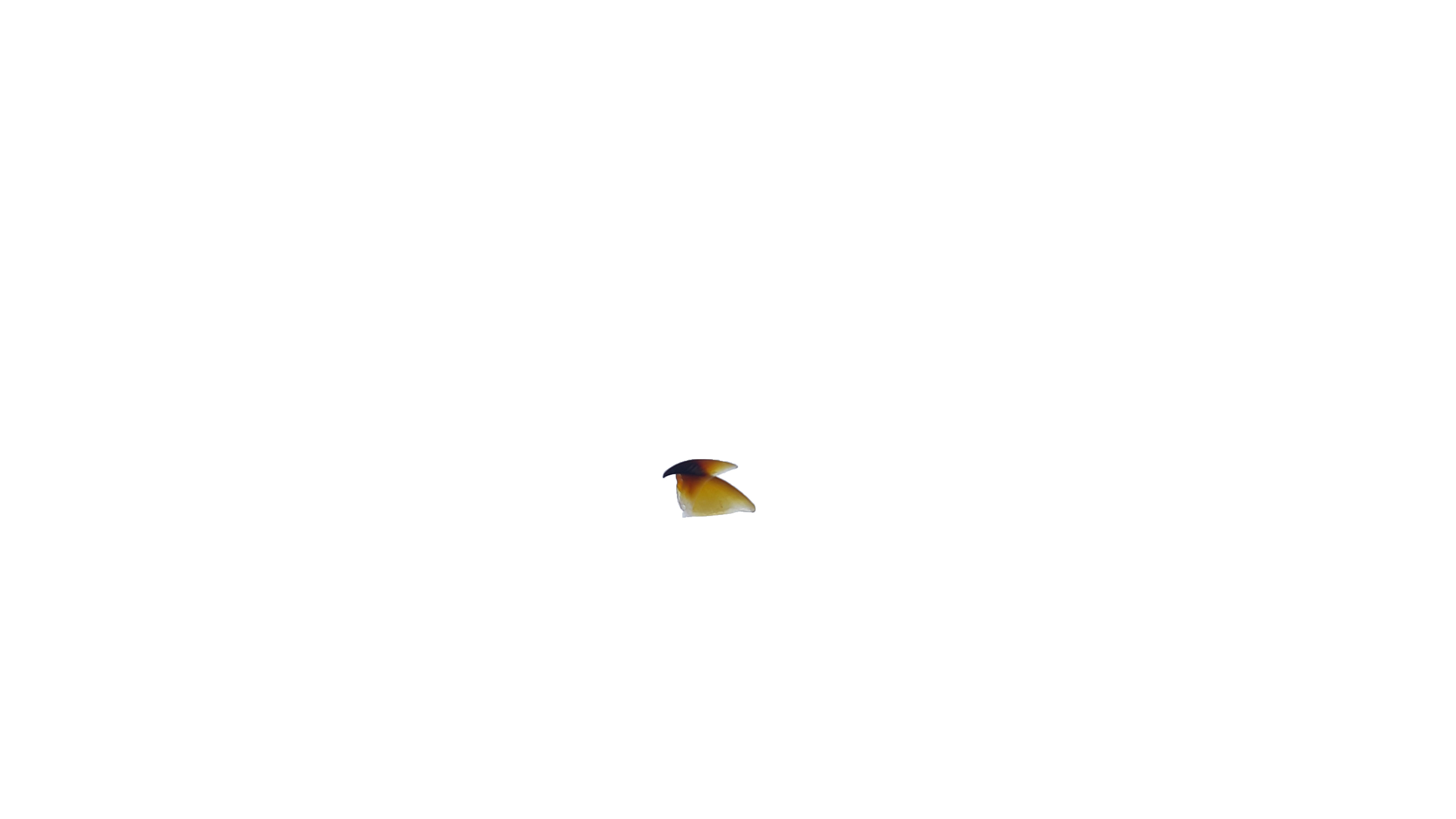

Supplement: Supplemental Information 2 — C2-Sepia aculeata, C3-Sepioteuthis lessoniana, C6-Sepia esculenta, O2-Amphioctopus aegina, S1-Loliolus uyii, S3-Uroteuthis chinensis, S4-Uroteuthis edulis [file peerj-09-11825-s002.zip › _Preprocessing_Upper_Beak/C6/U-l-C6-23.jpg]

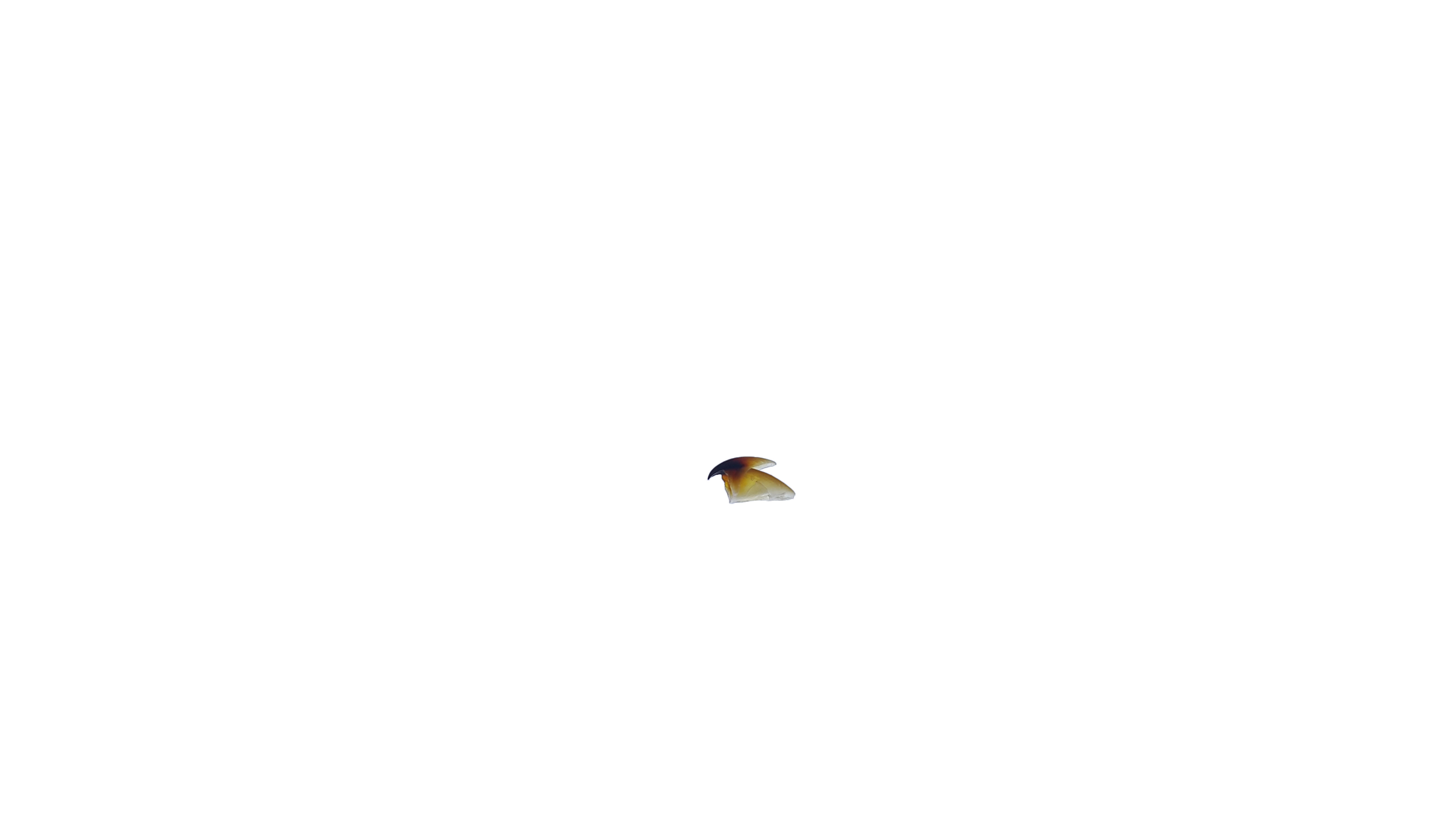

Supplement: Supplemental Information 2 — C2-Sepia aculeata, C3-Sepioteuthis lessoniana, C6-Sepia esculenta, O2-Amphioctopus aegina, S1-Loliolus uyii, S3-Uroteuthis chinensis, S4-Uroteuthis edulis [file peerj-09-11825-s002.zip › _Preprocessing_Upper_Beak/C6/U-l-C6-24.jpg]

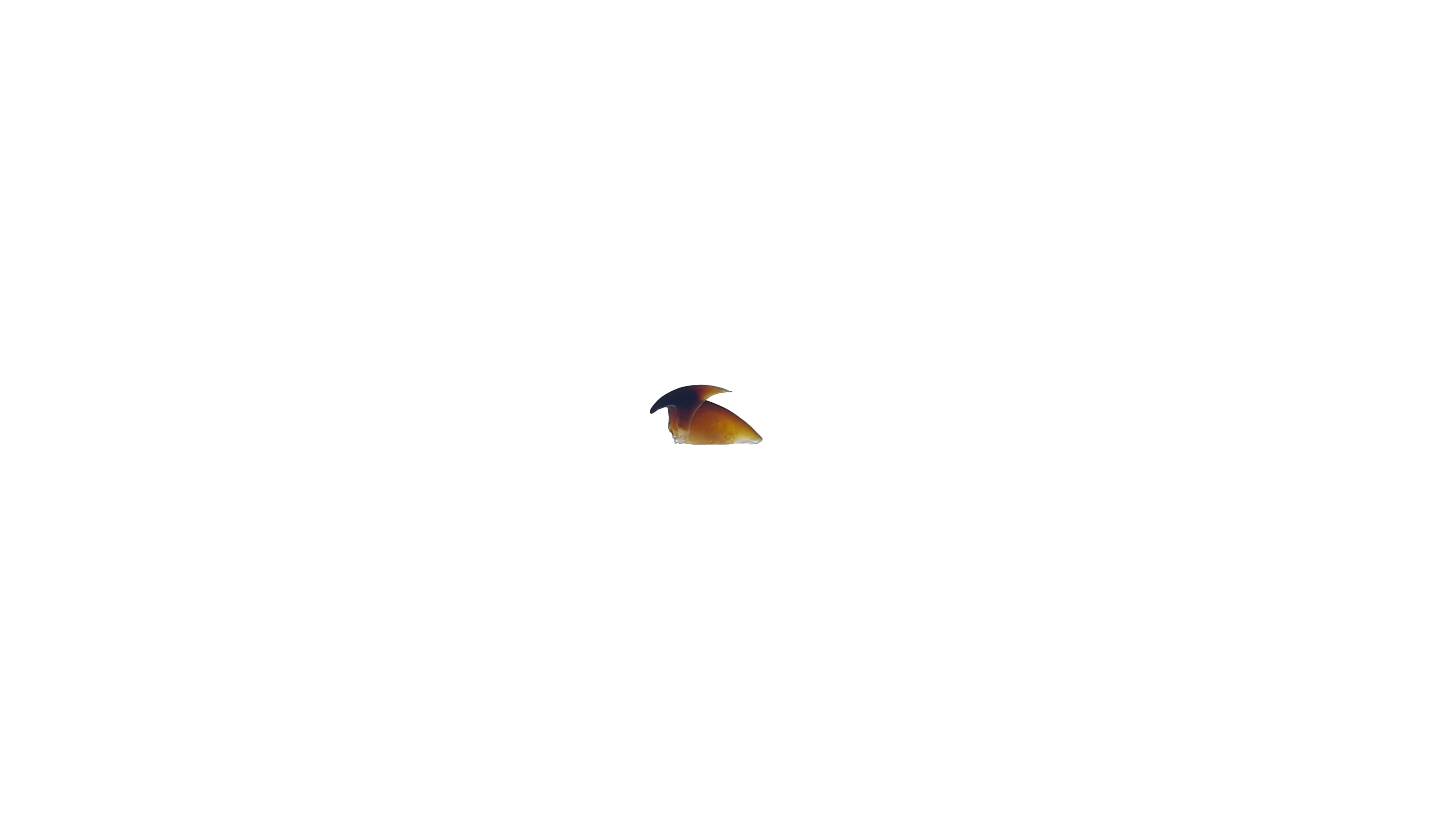

Supplement: Supplemental Information 2 — C2-Sepia aculeata, C3-Sepioteuthis lessoniana, C6-Sepia esculenta, O2-Amphioctopus aegina, S1-Loliolus uyii, S3-Uroteuthis chinensis, S4-Uroteuthis edulis [file peerj-09-11825-s002.zip › _Preprocessing_Upper_Beak/C6/U-l-C6-25.jpg]

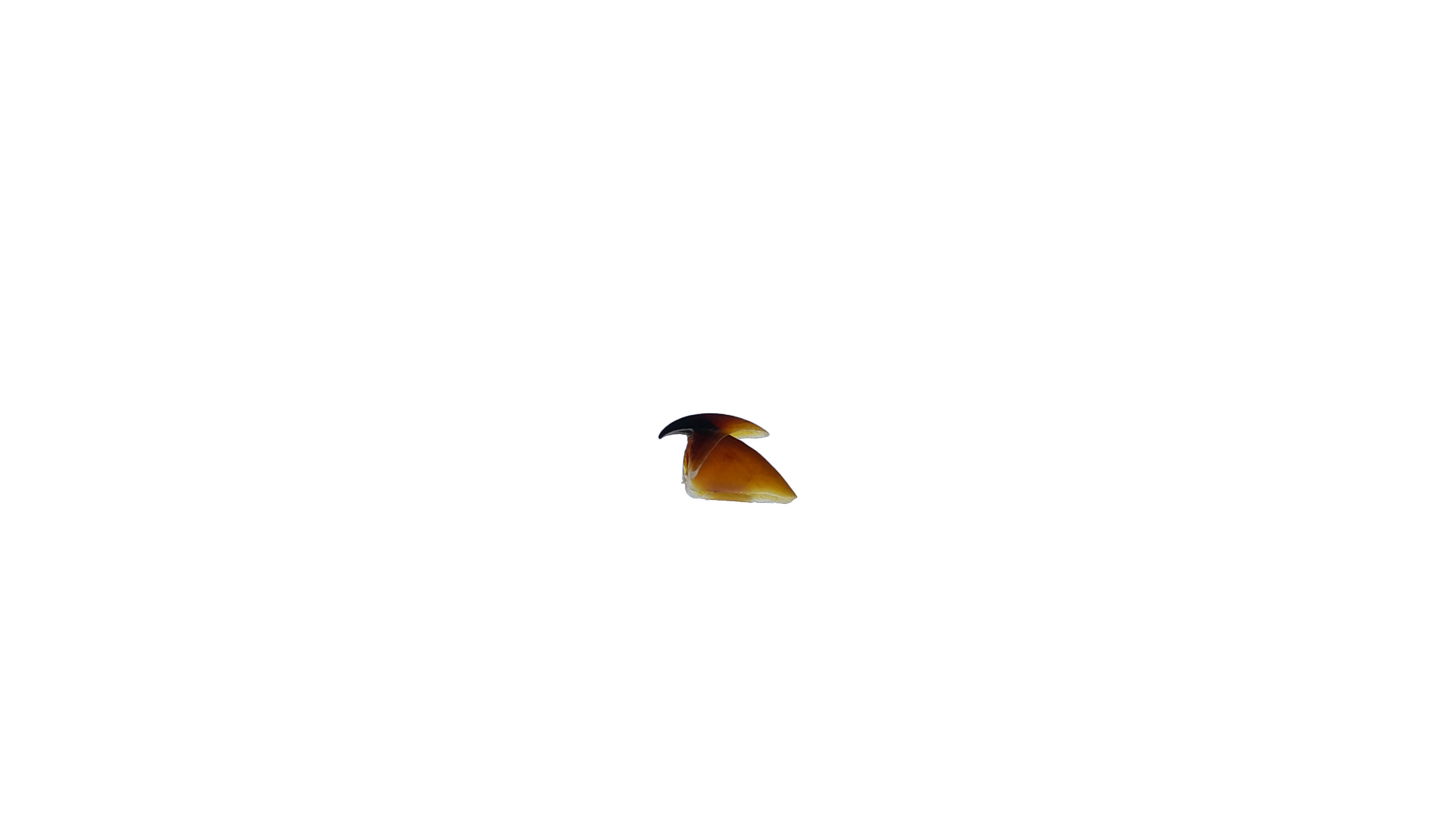

Supplement: Supplemental Information 2 — C2-Sepia aculeata, C3-Sepioteuthis lessoniana, C6-Sepia esculenta, O2-Amphioctopus aegina, S1-Loliolus uyii, S3-Uroteuthis chinensis, S4-Uroteuthis edulis [file peerj-09-11825-s002.zip › _Preprocessing_Upper_Beak/C6/U-l-C6-3.jpg]

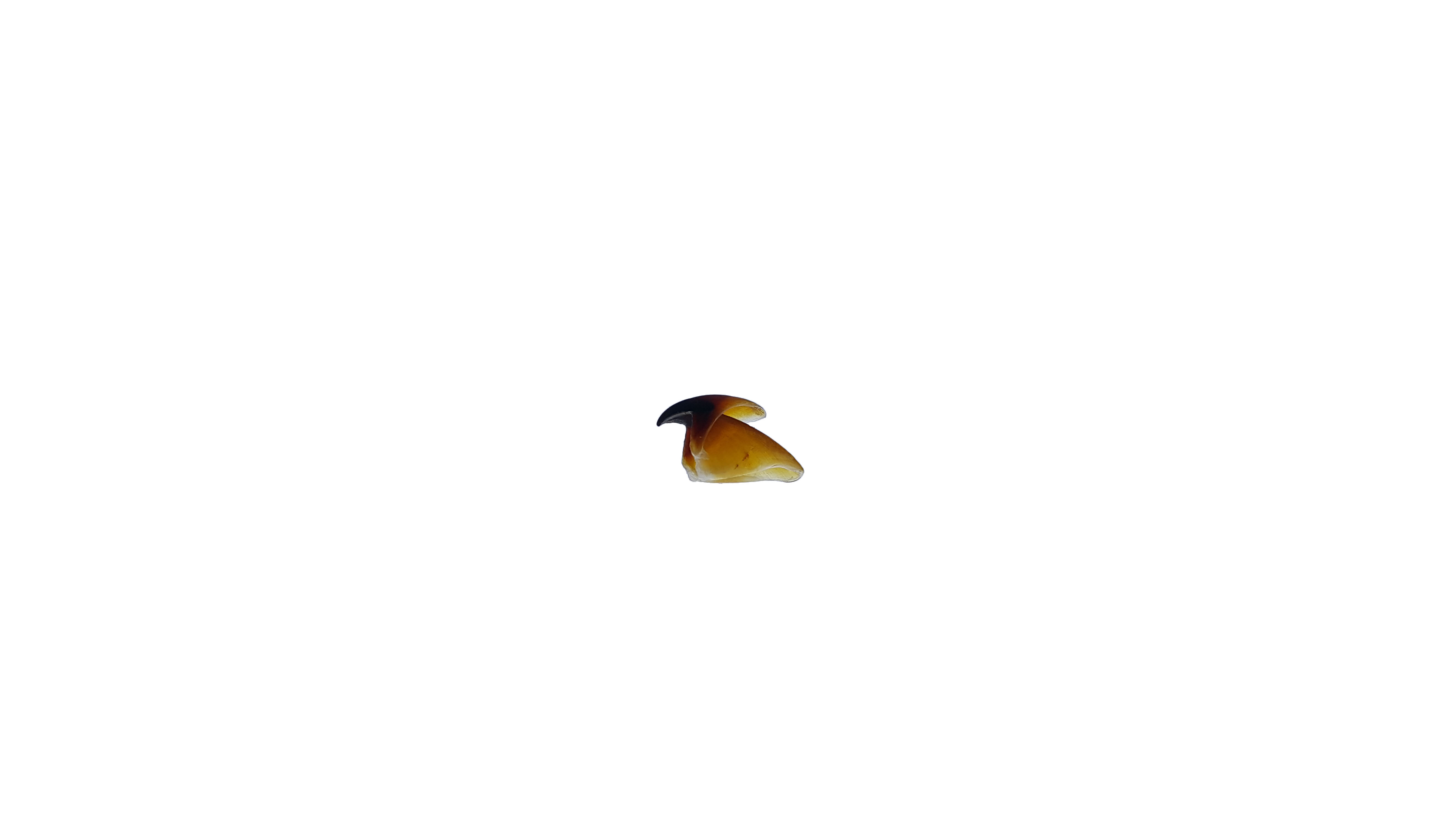

Supplement: Supplemental Information 2 — C2-Sepia aculeata, C3-Sepioteuthis lessoniana, C6-Sepia esculenta, O2-Amphioctopus aegina, S1-Loliolus uyii, S3-Uroteuthis chinensis, S4-Uroteuthis edulis [file peerj-09-11825-s002.zip › _Preprocessing_Upper_Beak/C6/U-l-C6-4.jpg]

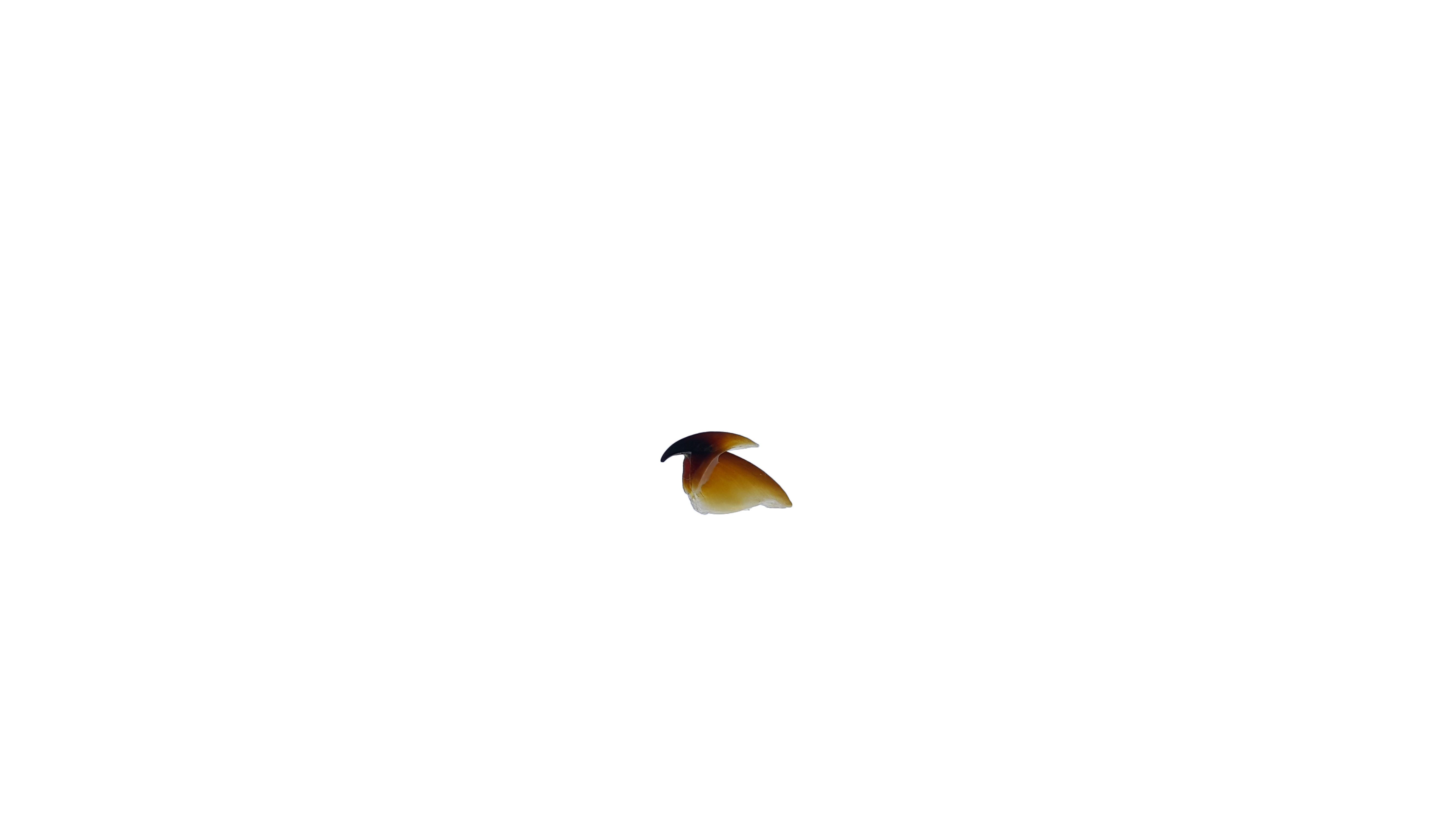

Supplement: Supplemental Information 2 — C2-Sepia aculeata, C3-Sepioteuthis lessoniana, C6-Sepia esculenta, O2-Amphioctopus aegina, S1-Loliolus uyii, S3-Uroteuthis chinensis, S4-Uroteuthis edulis [file peerj-09-11825-s002.zip › _Preprocessing_Upper_Beak/C6/U-l-C6-5.jpg]

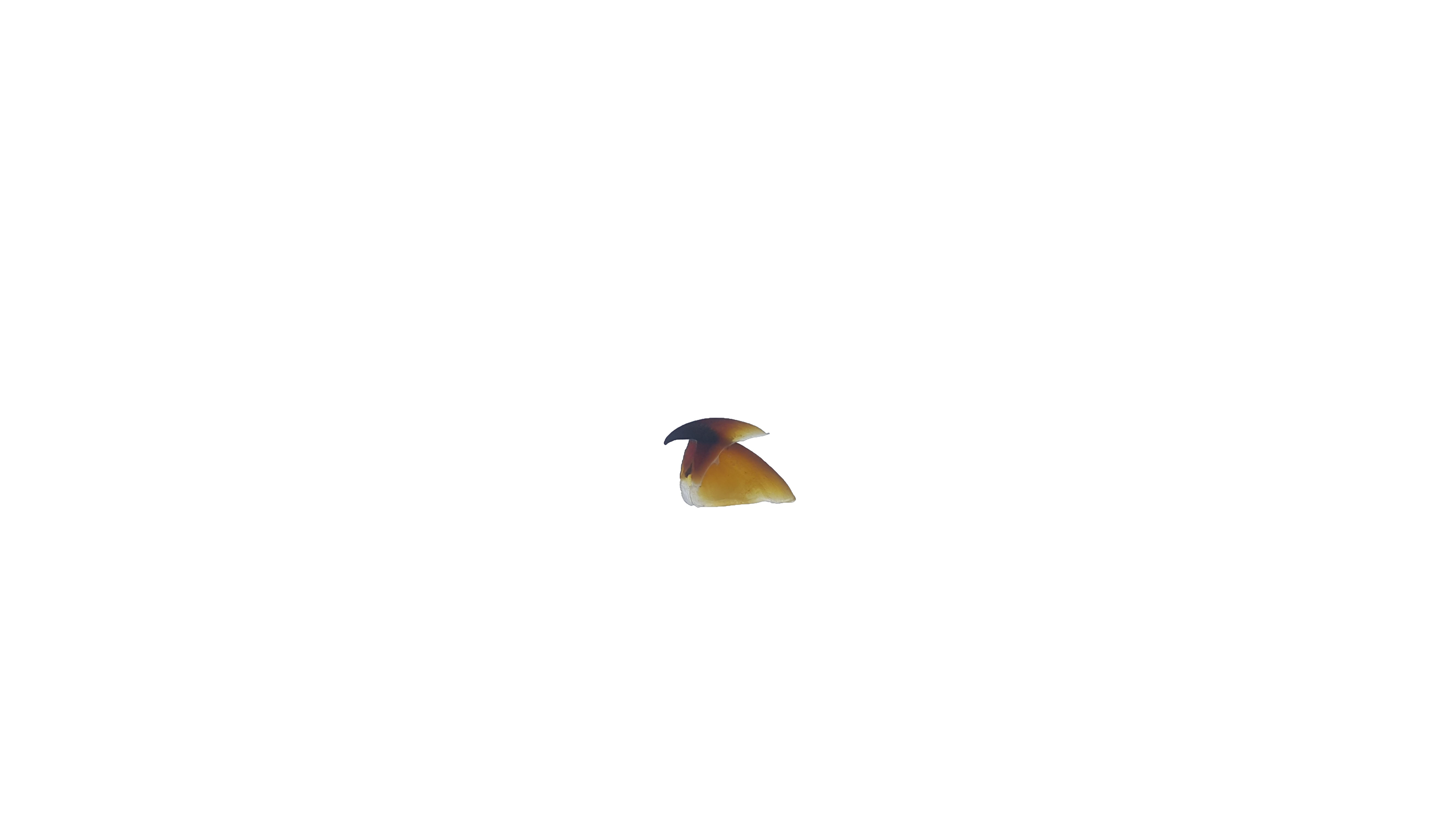

Supplement: Supplemental Information 2 — C2-Sepia aculeata, C3-Sepioteuthis lessoniana, C6-Sepia esculenta, O2-Amphioctopus aegina, S1-Loliolus uyii, S3-Uroteuthis chinensis, S4-Uroteuthis edulis [file peerj-09-11825-s002.zip › _Preprocessing_Upper_Beak/C6/U-l-C6-6.jpg]

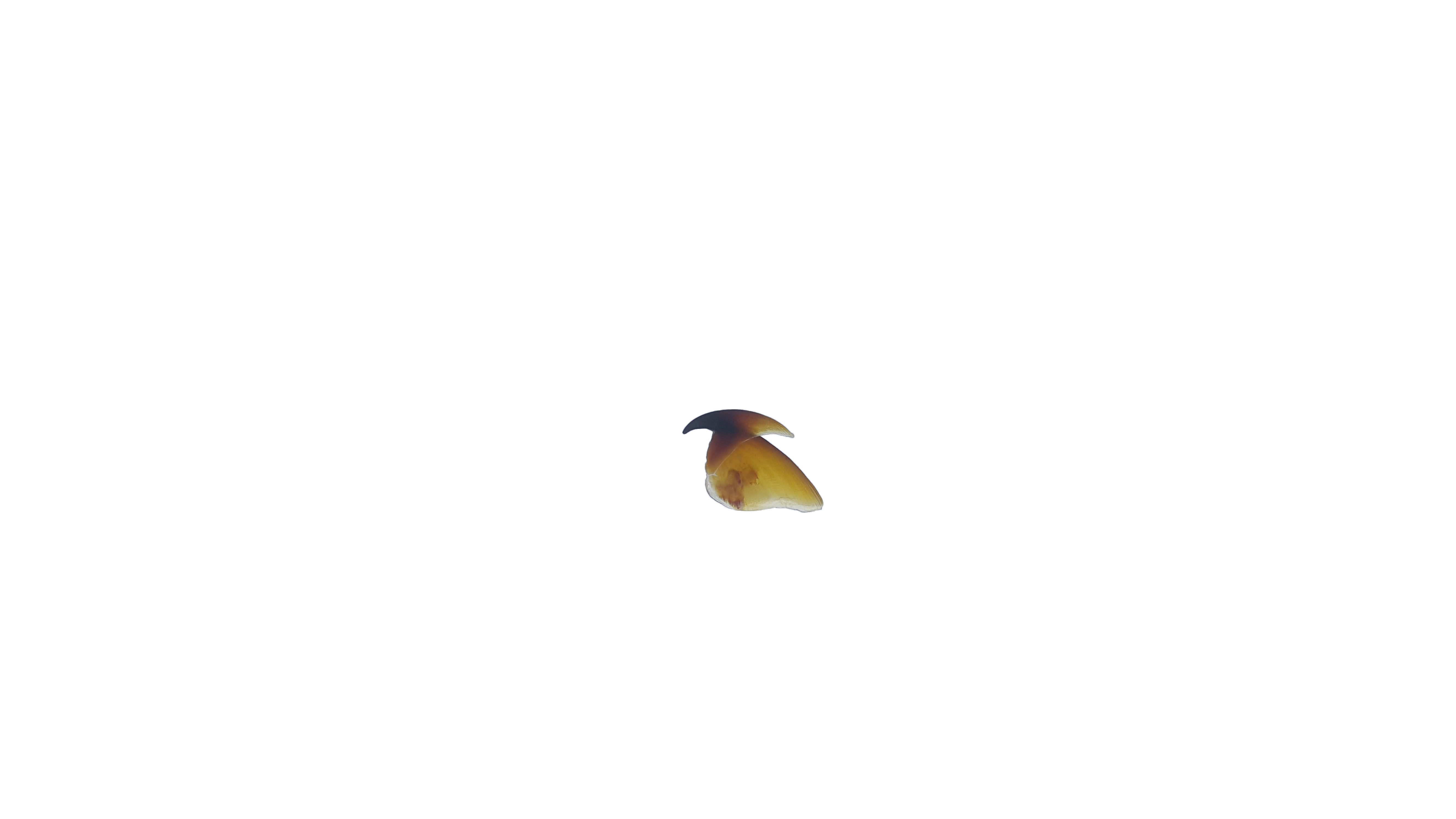

Supplement: Supplemental Information 2 — C2-Sepia aculeata, C3-Sepioteuthis lessoniana, C6-Sepia esculenta, O2-Amphioctopus aegina, S1-Loliolus uyii, S3-Uroteuthis chinensis, S4-Uroteuthis edulis [file peerj-09-11825-s002.zip › _Preprocessing_Upper_Beak/C6/U-l-C6-8.jpg]

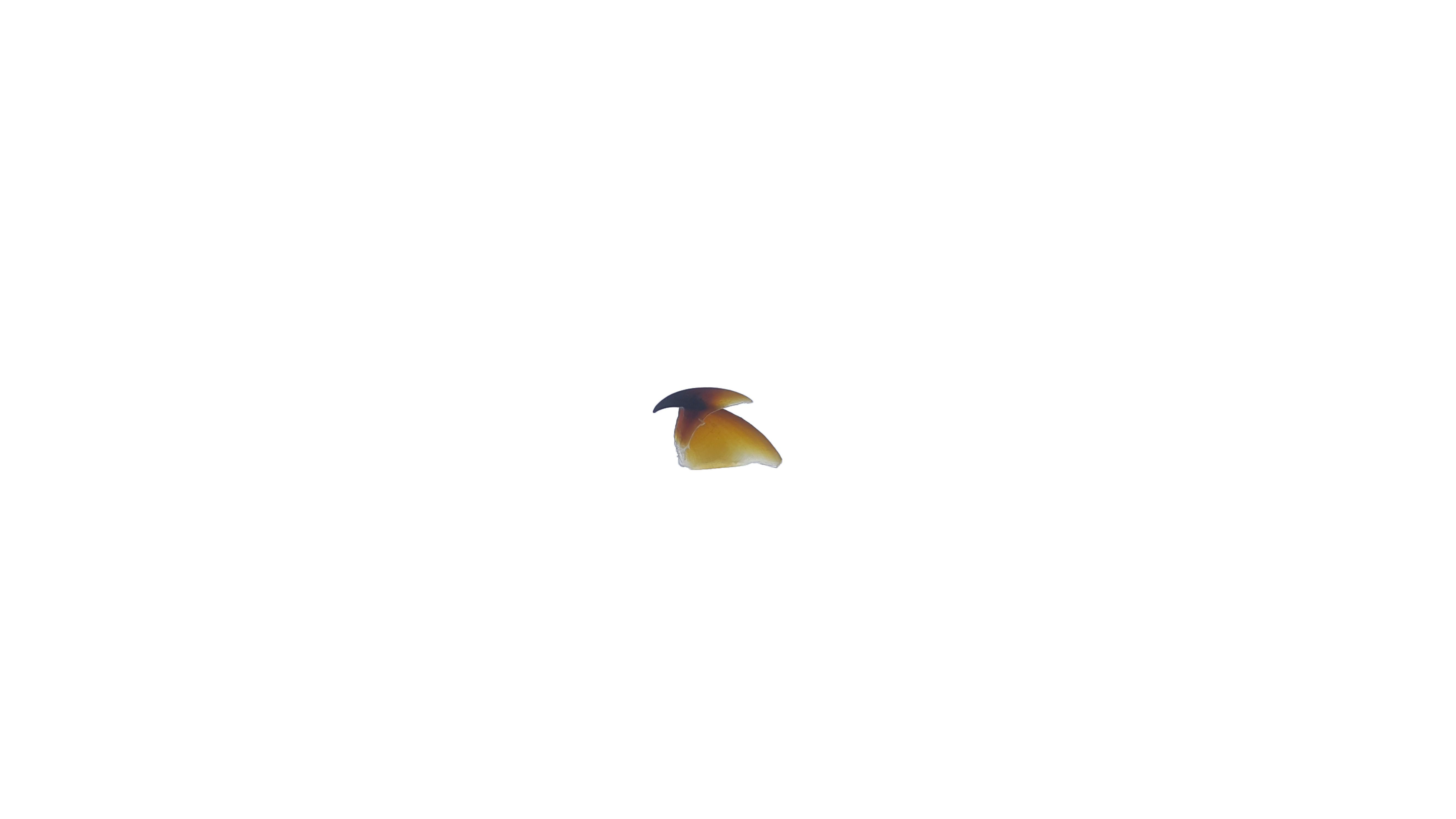

Supplement: Supplemental Information 2 — C2-Sepia aculeata, C3-Sepioteuthis lessoniana, C6-Sepia esculenta, O2-Amphioctopus aegina, S1-Loliolus uyii, S3-Uroteuthis chinensis, S4-Uroteuthis edulis [file peerj-09-11825-s002.zip › _Preprocessing_Upper_Beak/C6/U-l-C6-9.jpg]

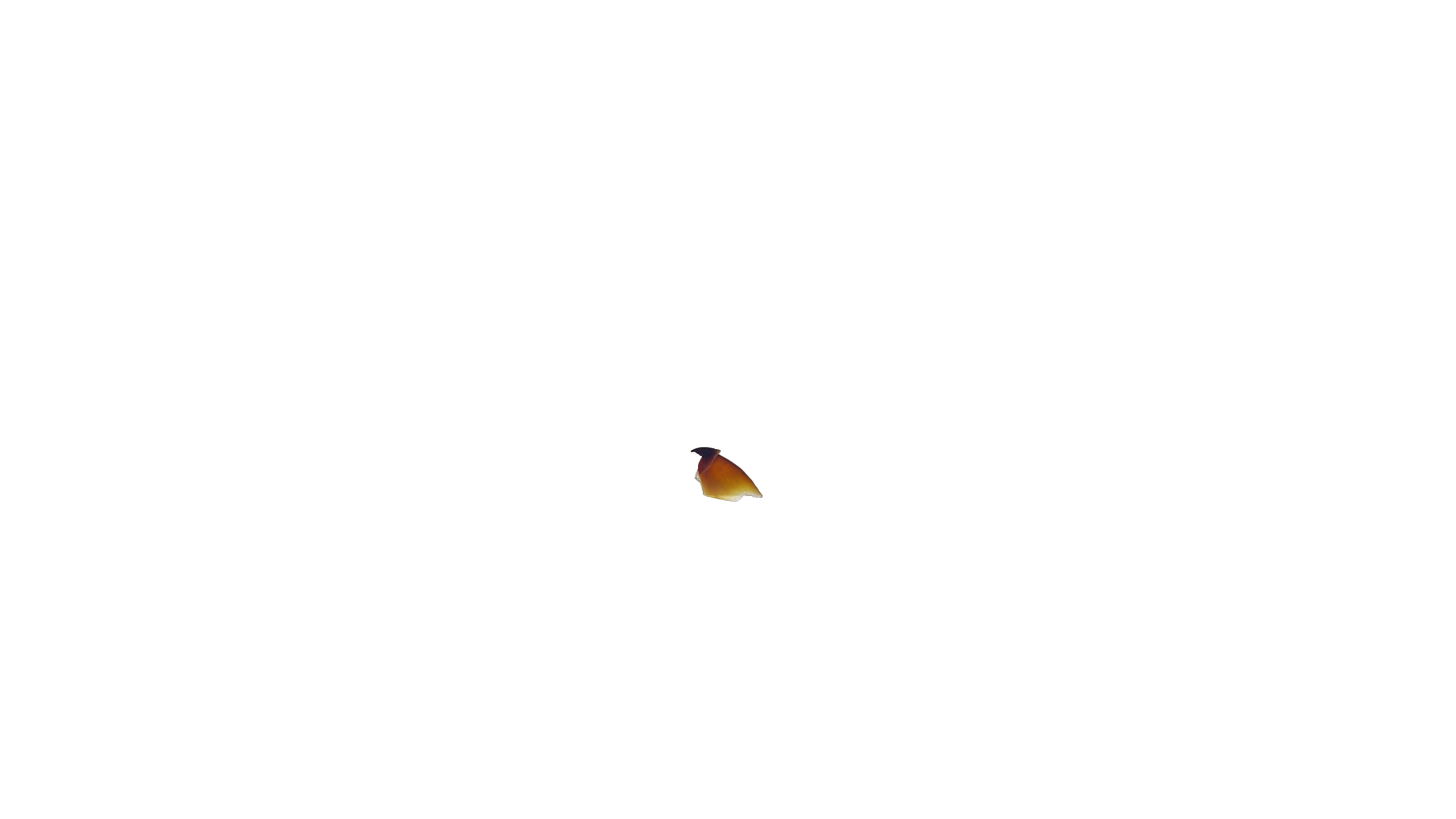

Supplement: Supplemental Information 2 — C2-Sepia aculeata, C3-Sepioteuthis lessoniana, C6-Sepia esculenta, O2-Amphioctopus aegina, S1-Loliolus uyii, S3-Uroteuthis chinensis, S4-Uroteuthis edulis [file peerj-09-11825-s002.zip › _Preprocessing_Upper_Beak/O2/U-l-O2-1.jpg]

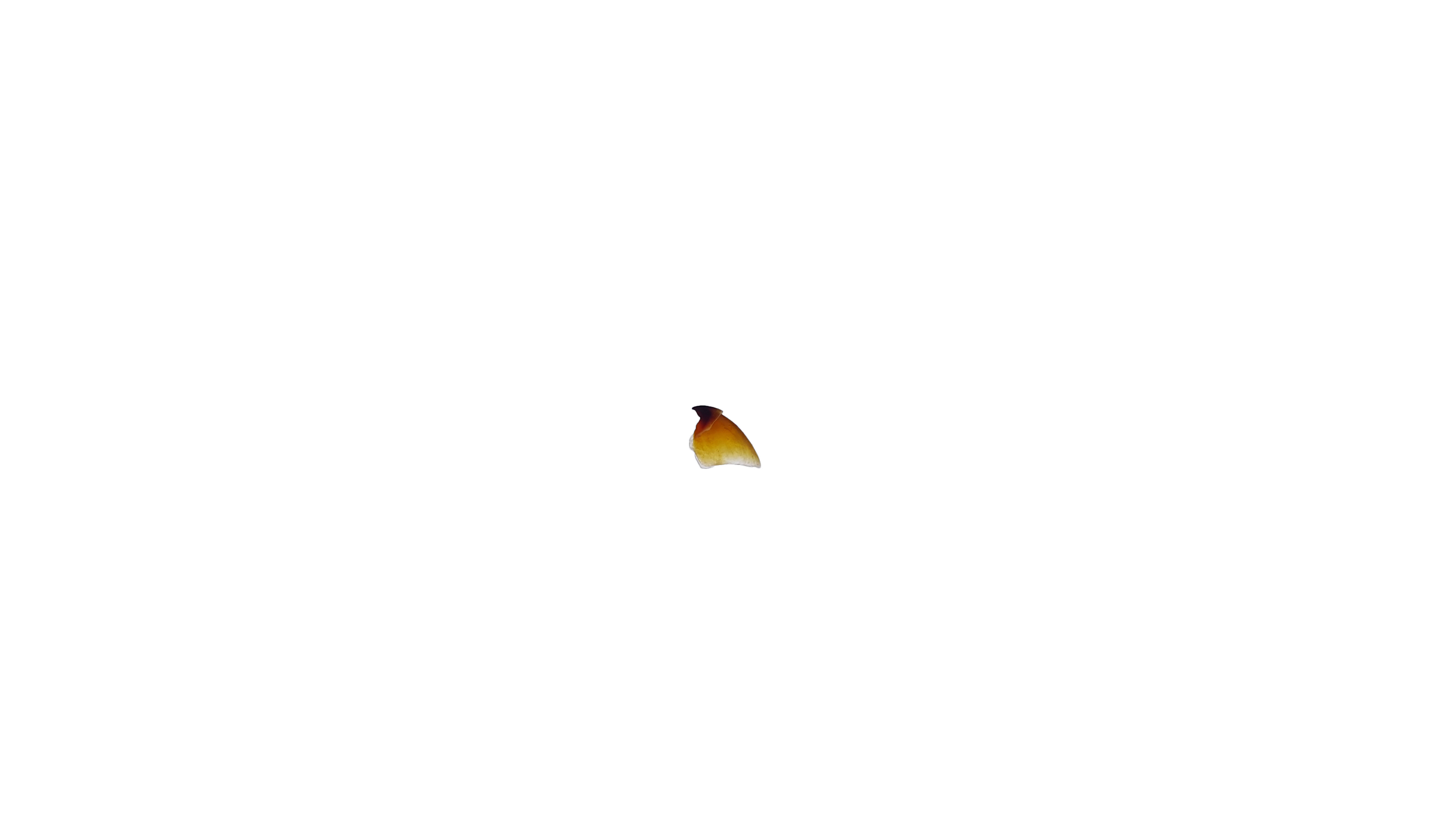

Supplement: Supplemental Information 2 — C2-Sepia aculeata, C3-Sepioteuthis lessoniana, C6-Sepia esculenta, O2-Amphioctopus aegina, S1-Loliolus uyii, S3-Uroteuthis chinensis, S4-Uroteuthis edulis [file peerj-09-11825-s002.zip › _Preprocessing_Upper_Beak/O2/U-l-O2-10.jpg]

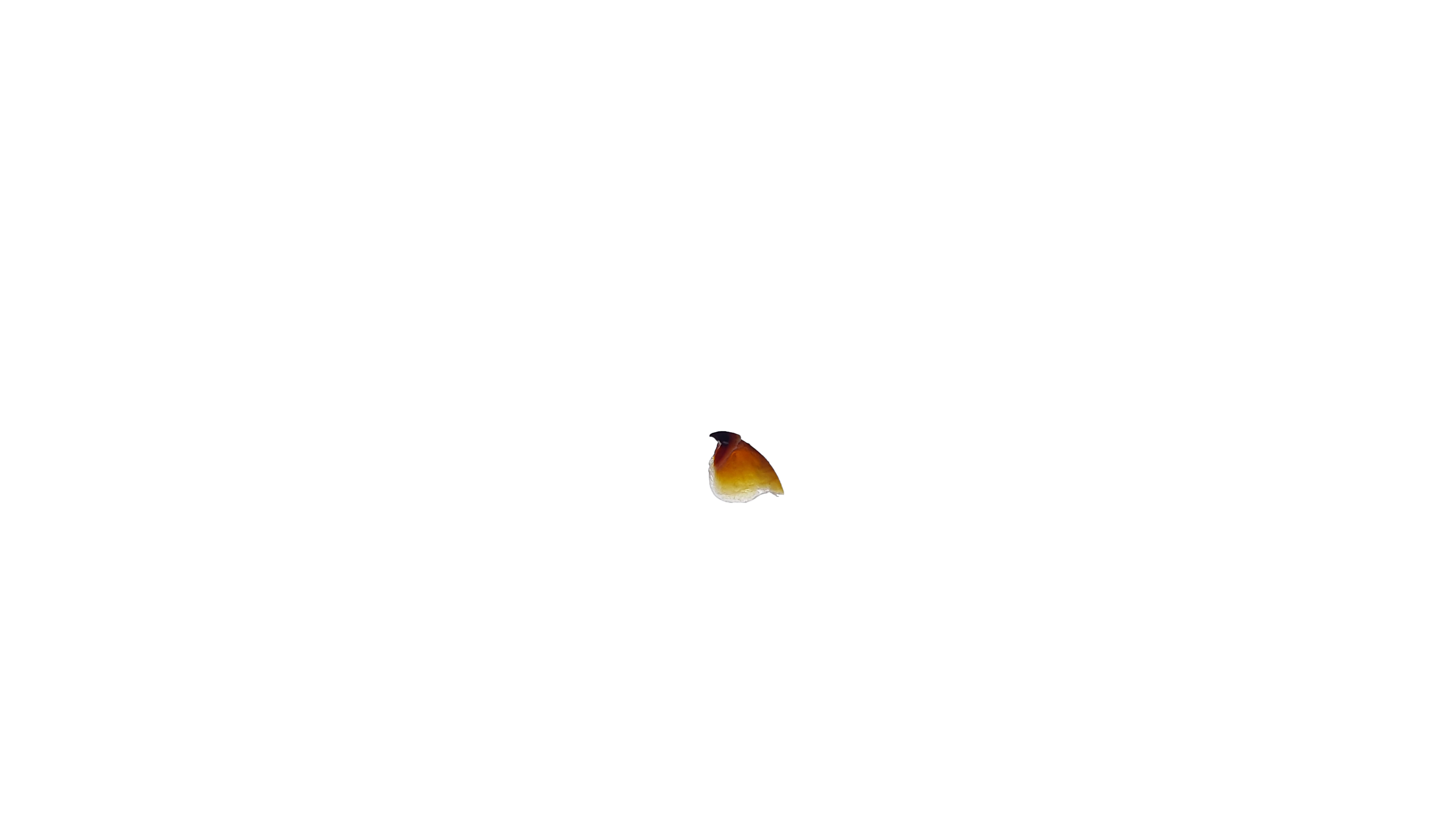

Supplement: Supplemental Information 2 — C2-Sepia aculeata, C3-Sepioteuthis lessoniana, C6-Sepia esculenta, O2-Amphioctopus aegina, S1-Loliolus uyii, S3-Uroteuthis chinensis, S4-Uroteuthis edulis [file peerj-09-11825-s002.zip › _Preprocessing_Upper_Beak/O2/U-l-O2-11.jpg]

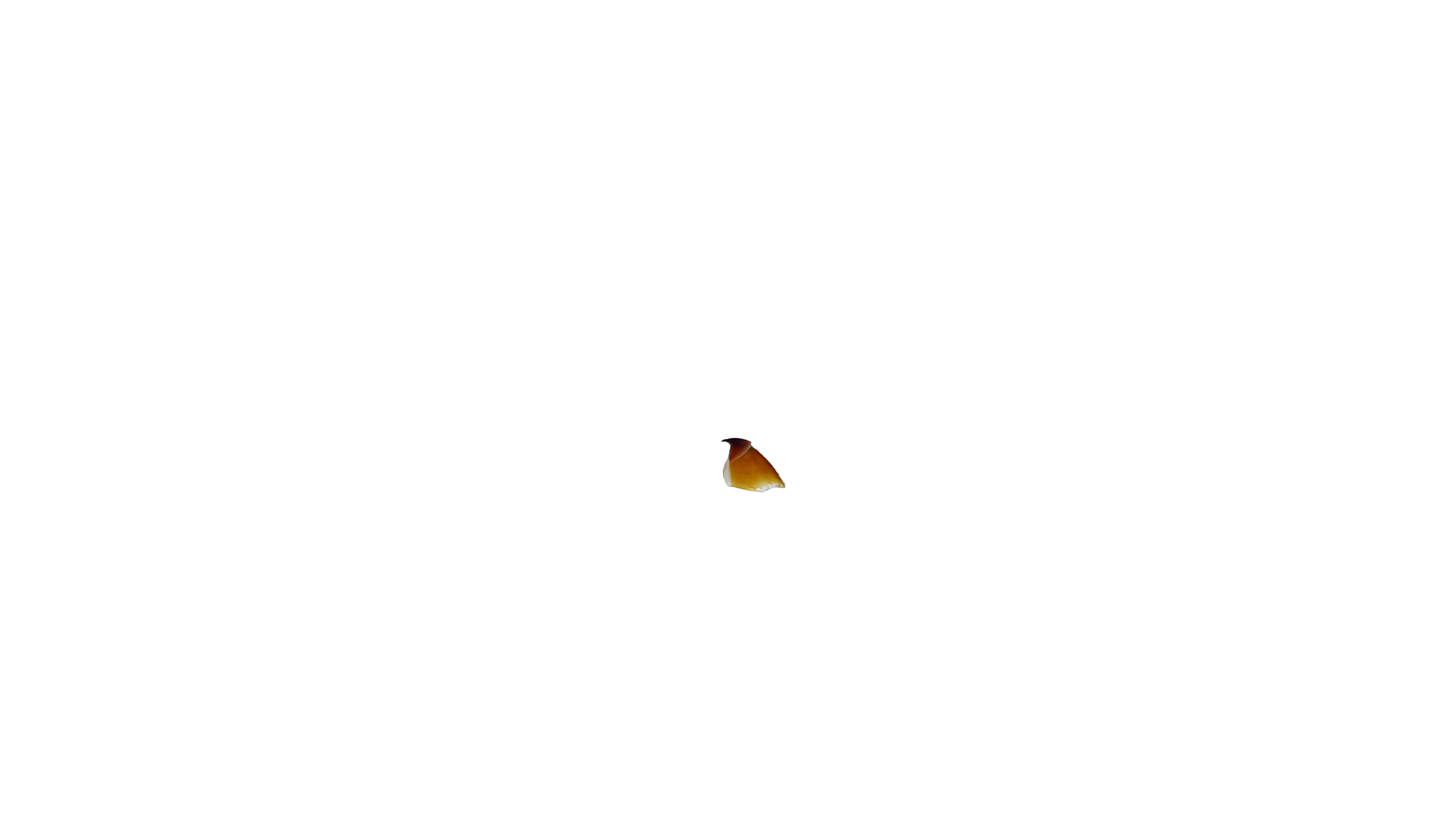

Supplement: Supplemental Information 2 — C2-Sepia aculeata, C3-Sepioteuthis lessoniana, C6-Sepia esculenta, O2-Amphioctopus aegina, S1-Loliolus uyii, S3-Uroteuthis chinensis, S4-Uroteuthis edulis [file peerj-09-11825-s002.zip › _Preprocessing_Upper_Beak/O2/U-l-O2-12.jpg]

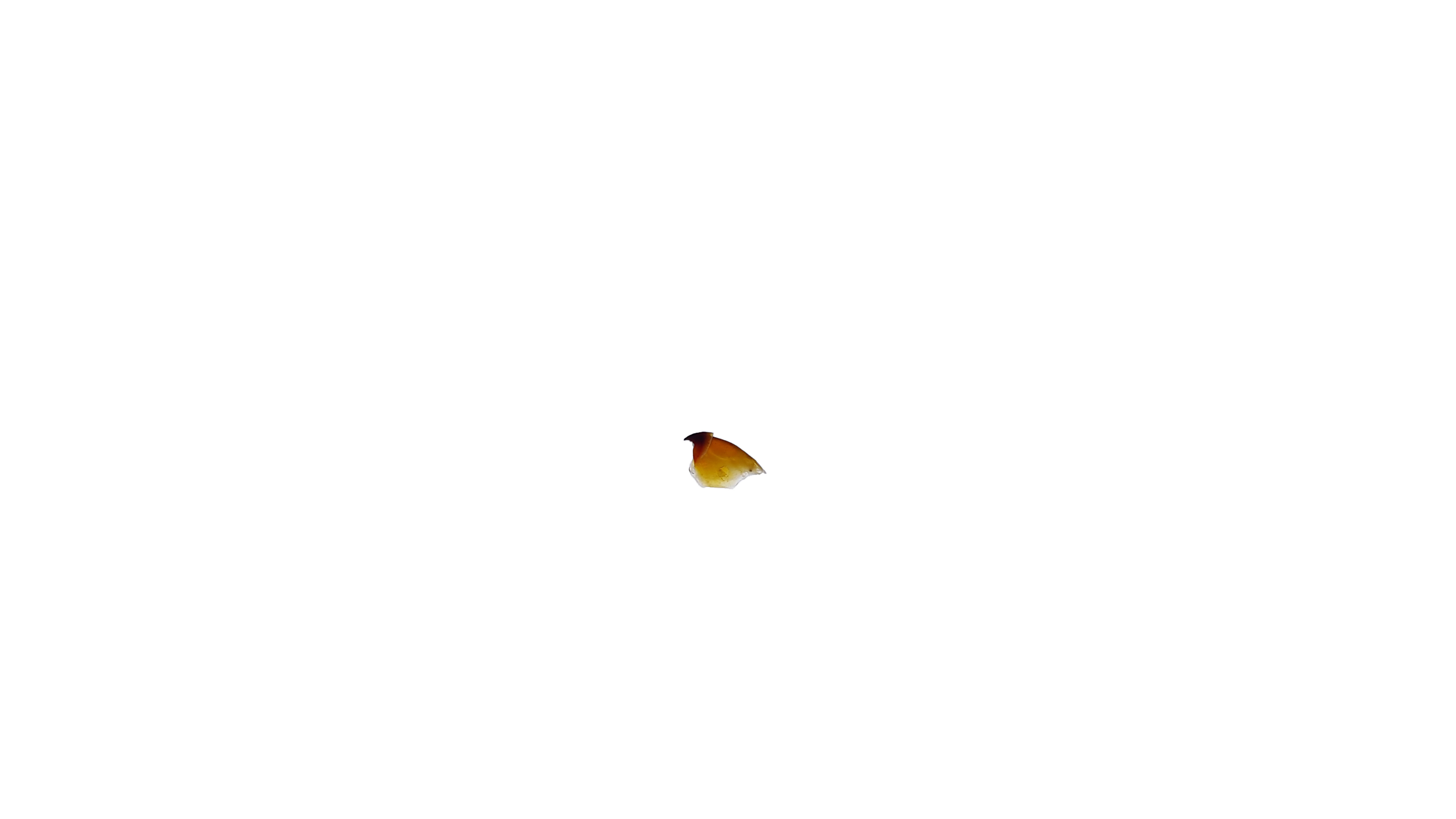

Supplement: Supplemental Information 2 — C2-Sepia aculeata, C3-Sepioteuthis lessoniana, C6-Sepia esculenta, O2-Amphioctopus aegina, S1-Loliolus uyii, S3-Uroteuthis chinensis, S4-Uroteuthis edulis [file peerj-09-11825-s002.zip › _Preprocessing_Upper_Beak/O2/U-l-O2-13.jpg]

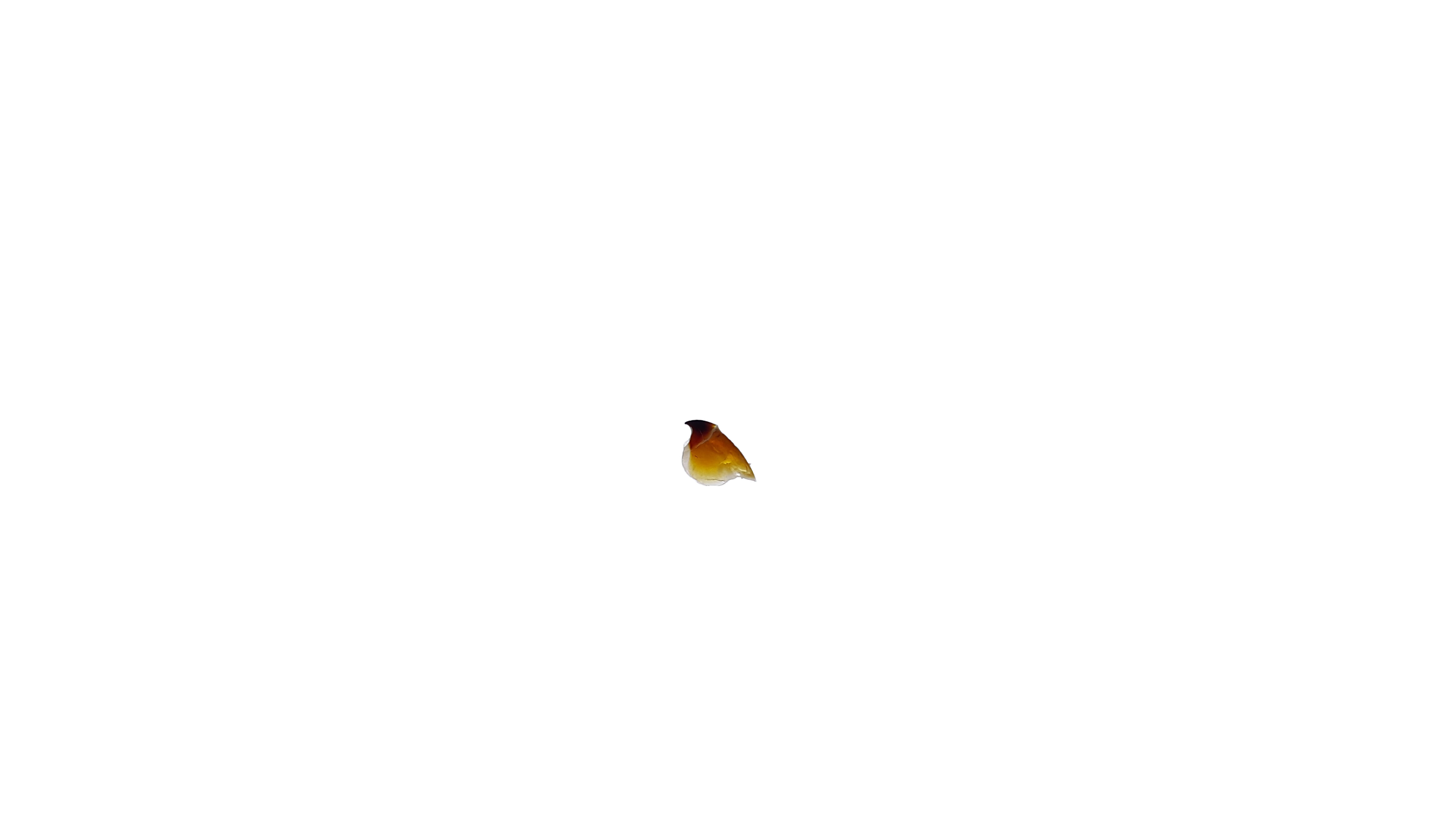

Supplement: Supplemental Information 2 — C2-Sepia aculeata, C3-Sepioteuthis lessoniana, C6-Sepia esculenta, O2-Amphioctopus aegina, S1-Loliolus uyii, S3-Uroteuthis chinensis, S4-Uroteuthis edulis [file peerj-09-11825-s002.zip › _Preprocessing_Upper_Beak/O2/U-l-O2-14.jpg]

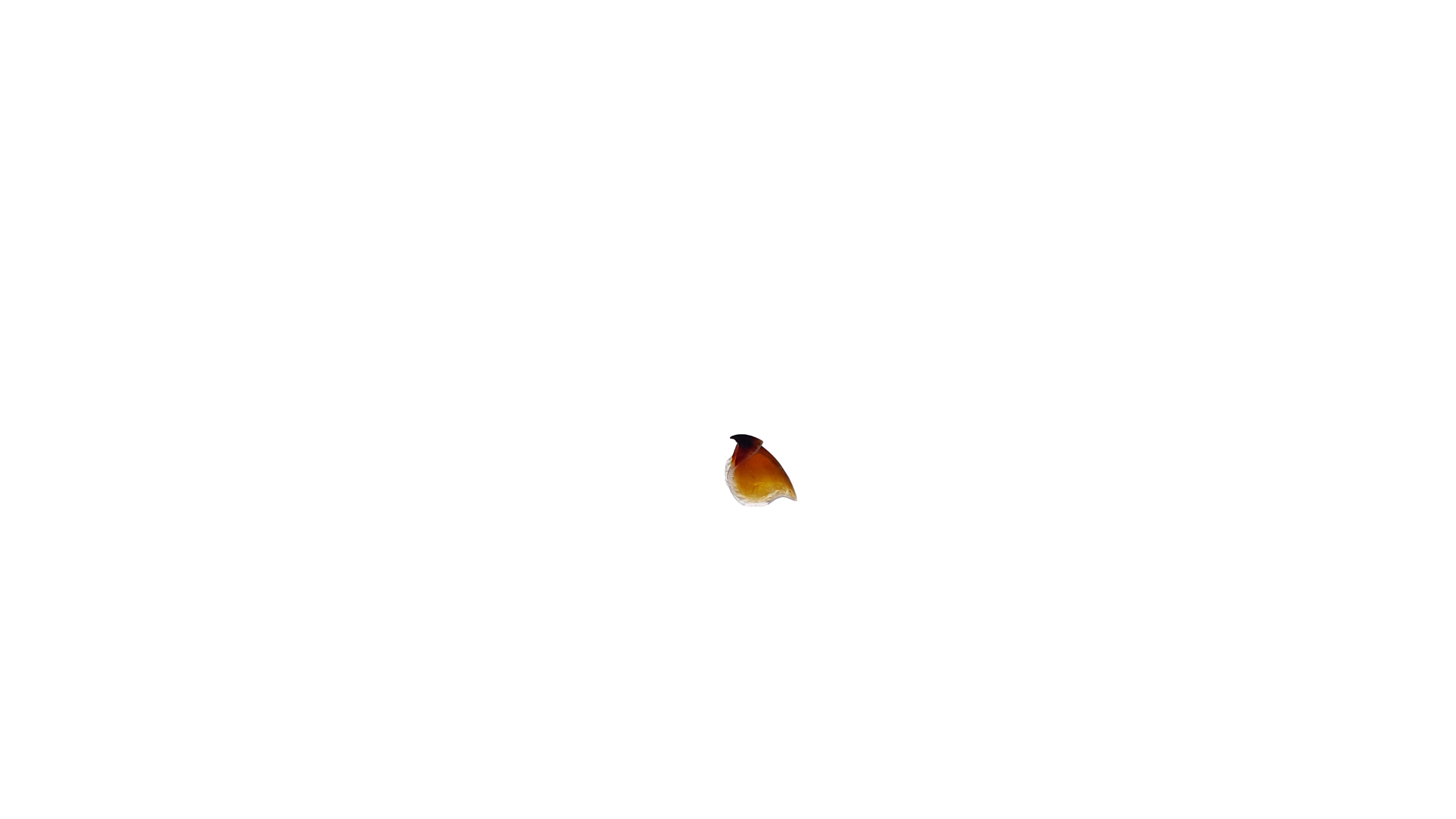

Supplement: Supplemental Information 2 — C2-Sepia aculeata, C3-Sepioteuthis lessoniana, C6-Sepia esculenta, O2-Amphioctopus aegina, S1-Loliolus uyii, S3-Uroteuthis chinensis, S4-Uroteuthis edulis [file peerj-09-11825-s002.zip › _Preprocessing_Upper_Beak/O2/U-l-O2-15.jpg]

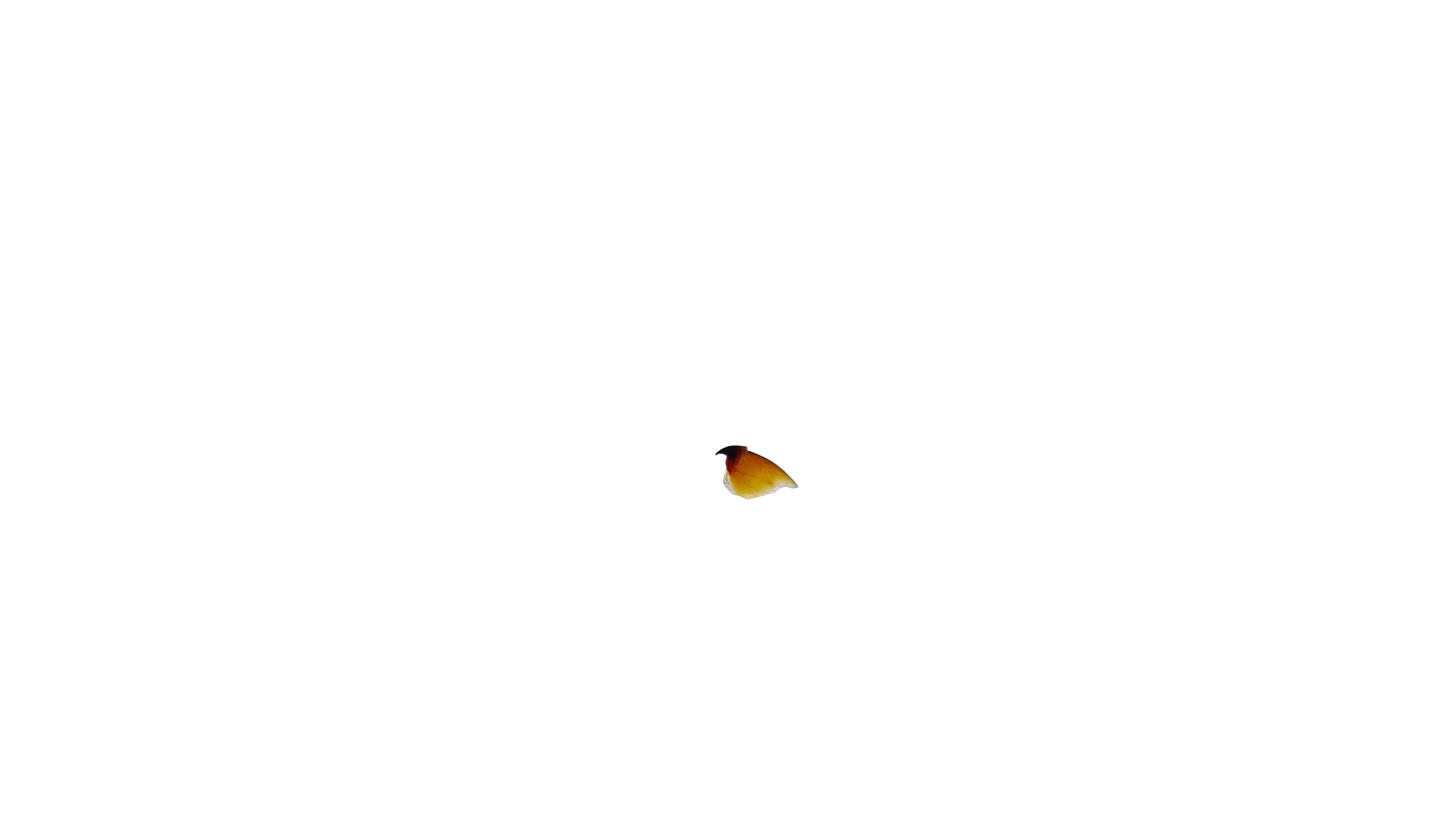

Supplement: Supplemental Information 2 — C2-Sepia aculeata, C3-Sepioteuthis lessoniana, C6-Sepia esculenta, O2-Amphioctopus aegina, S1-Loliolus uyii, S3-Uroteuthis chinensis, S4-Uroteuthis edulis [file peerj-09-11825-s002.zip › _Preprocessing_Upper_Beak/O2/U-l-O2-16.jpg]

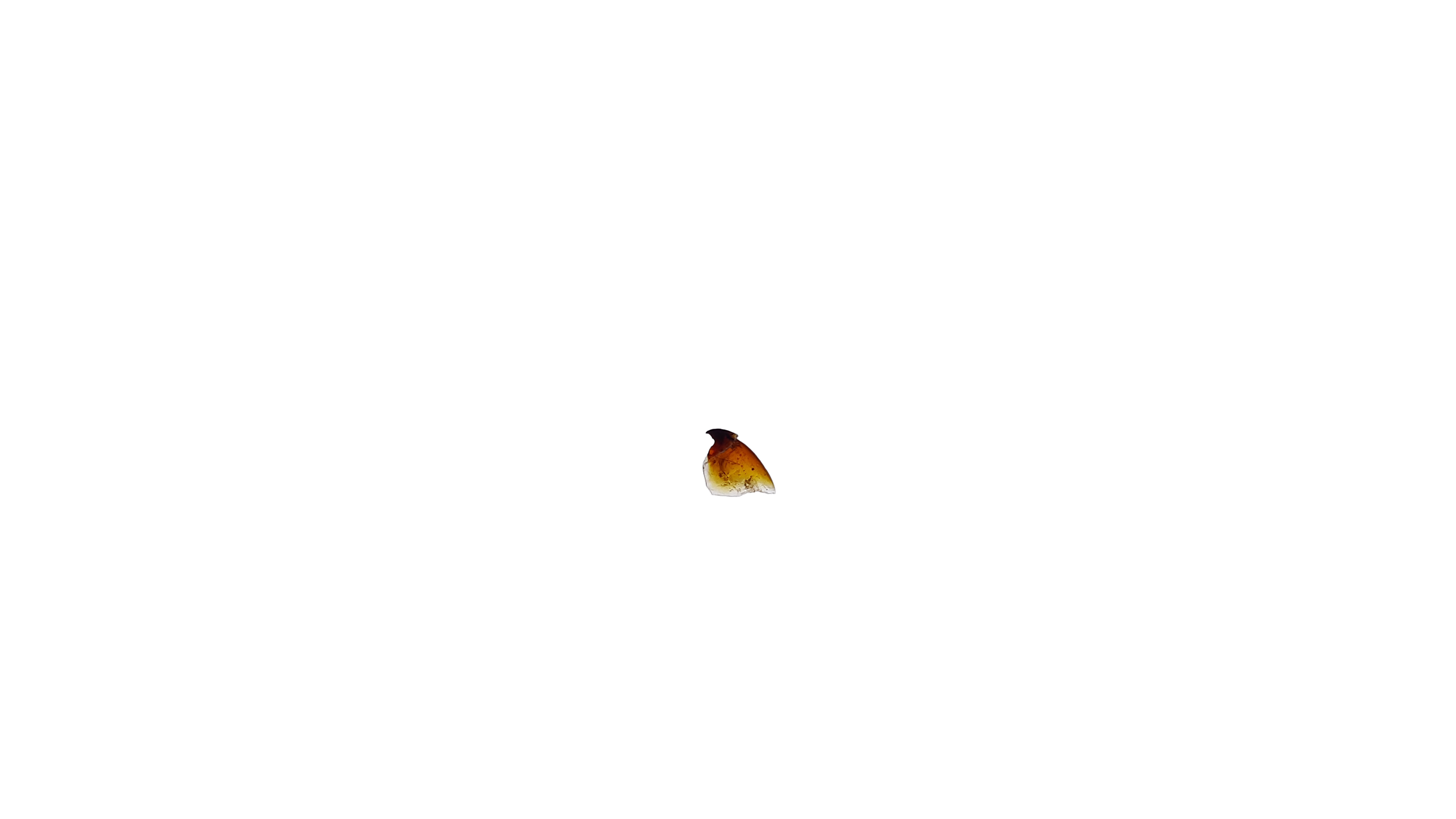

Supplement: Supplemental Information 2 — C2-Sepia aculeata, C3-Sepioteuthis lessoniana, C6-Sepia esculenta, O2-Amphioctopus aegina, S1-Loliolus uyii, S3-Uroteuthis chinensis, S4-Uroteuthis edulis [file peerj-09-11825-s002.zip › _Preprocessing_Upper_Beak/O2/U-l-O2-17.jpg]

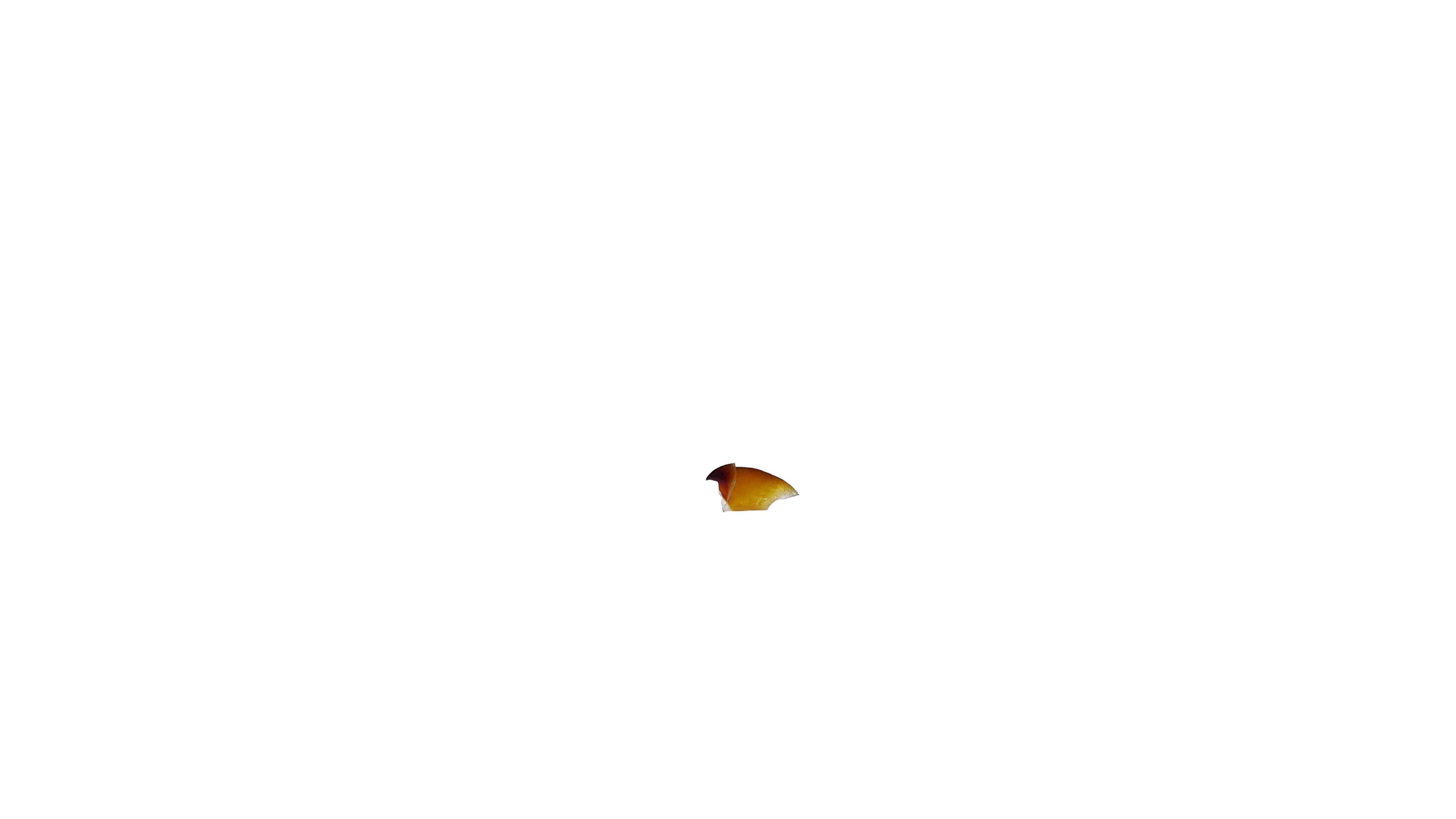

Supplement: Supplemental Information 2 — C2-Sepia aculeata, C3-Sepioteuthis lessoniana, C6-Sepia esculenta, O2-Amphioctopus aegina, S1-Loliolus uyii, S3-Uroteuthis chinensis, S4-Uroteuthis edulis [file peerj-09-11825-s002.zip › _Preprocessing_Upper_Beak/O2/U-l-O2-18.jpg]

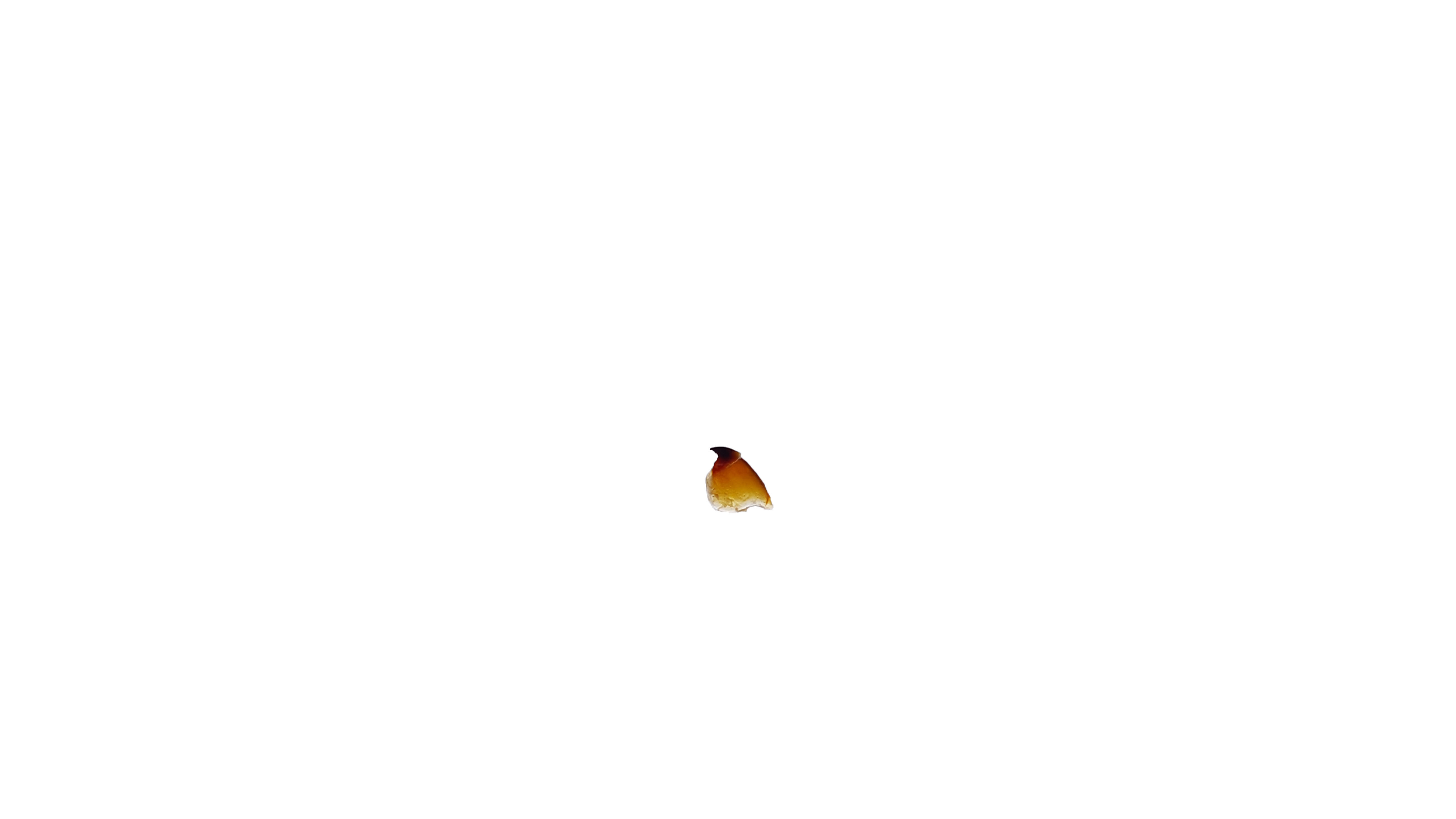

Supplement: Supplemental Information 2 — C2-Sepia aculeata, C3-Sepioteuthis lessoniana, C6-Sepia esculenta, O2-Amphioctopus aegina, S1-Loliolus uyii, S3-Uroteuthis chinensis, S4-Uroteuthis edulis [file peerj-09-11825-s002.zip › _Preprocessing_Upper_Beak/O2/U-l-O2-19.jpg]

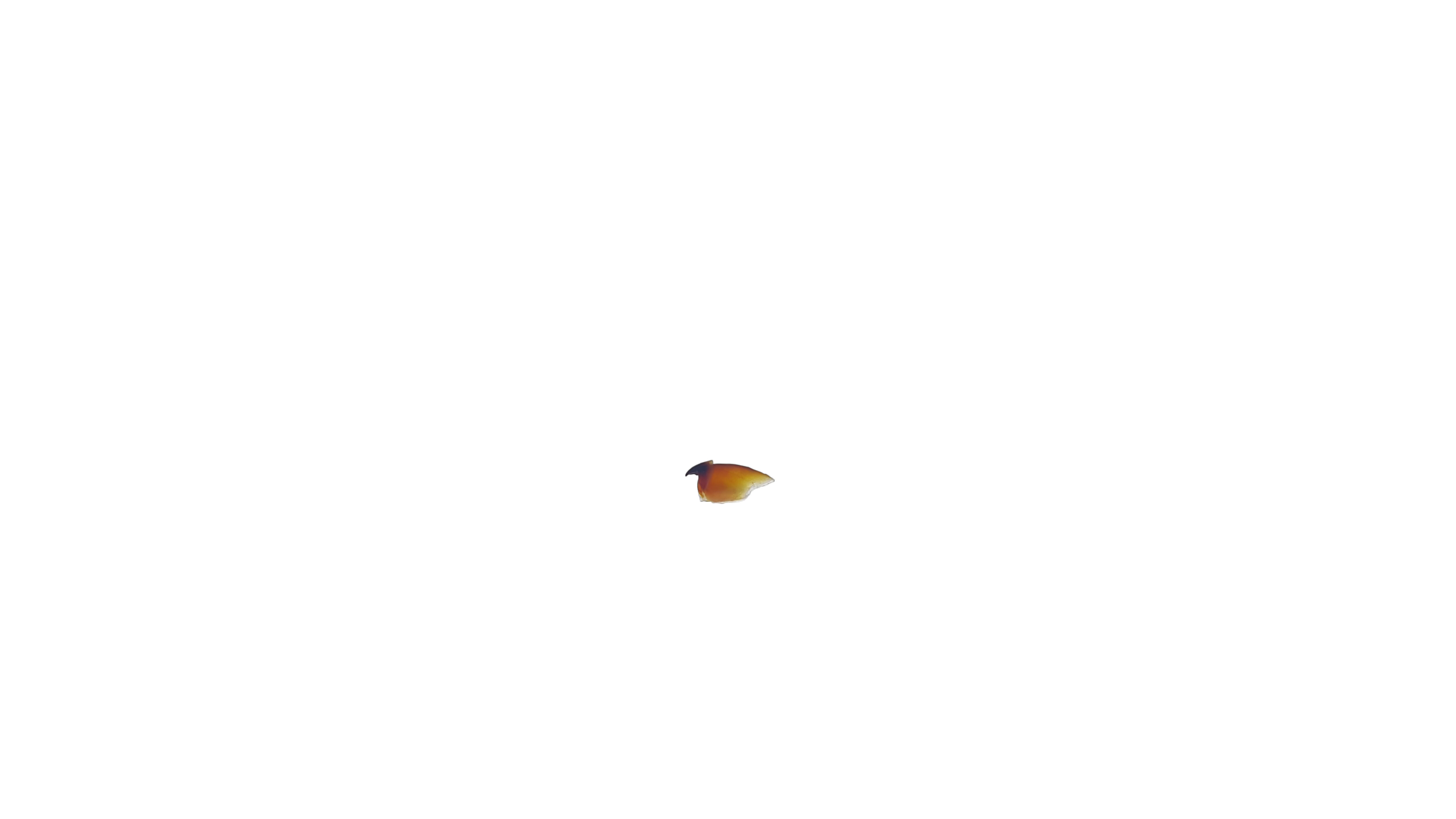

Supplement: Supplemental Information 2 — C2-Sepia aculeata, C3-Sepioteuthis lessoniana, C6-Sepia esculenta, O2-Amphioctopus aegina, S1-Loliolus uyii, S3-Uroteuthis chinensis, S4-Uroteuthis edulis [file peerj-09-11825-s002.zip › _Preprocessing_Upper_Beak/O2/U-l-O2-2.jpg]

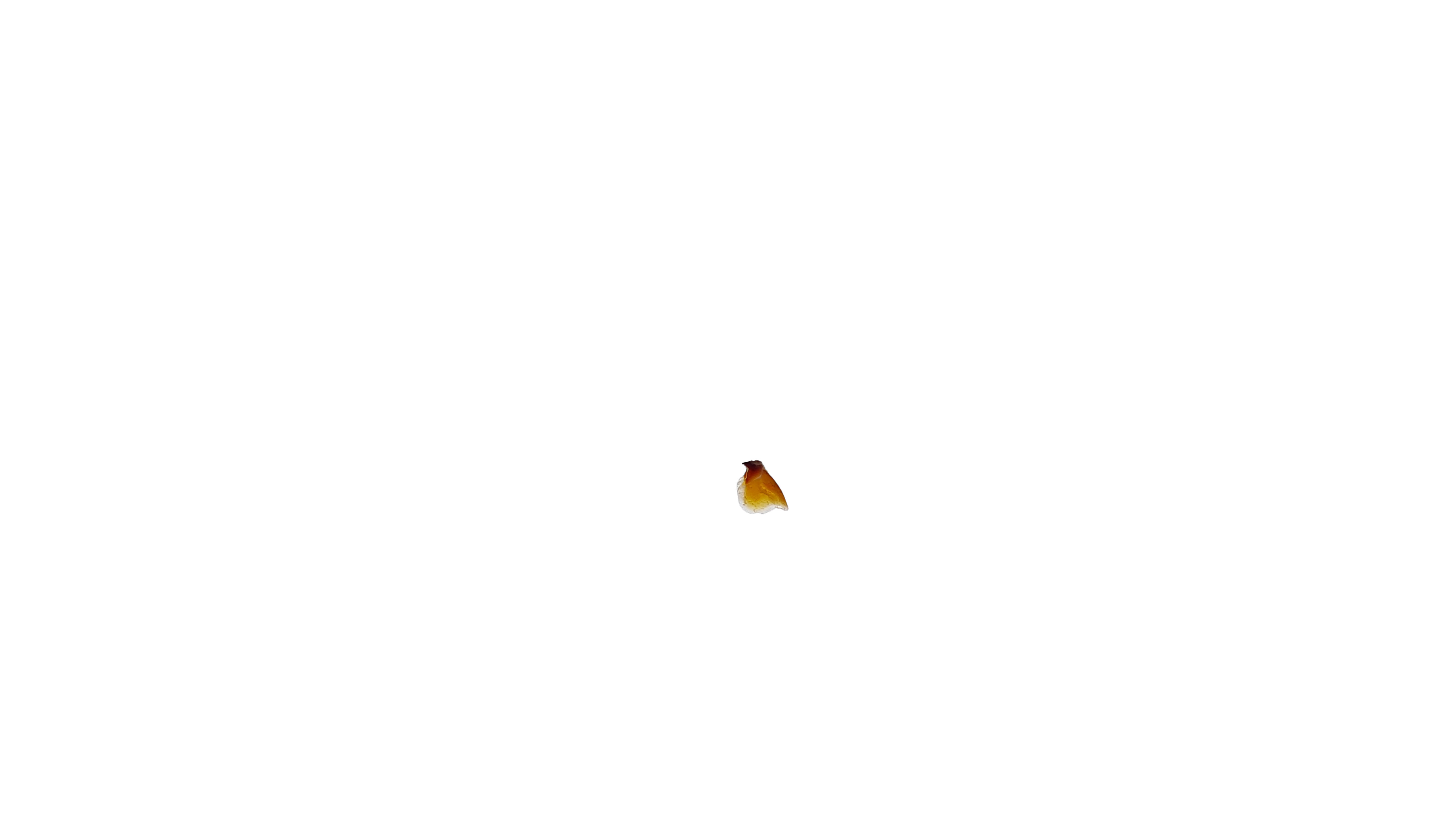

Supplement: Supplemental Information 2 — C2-Sepia aculeata, C3-Sepioteuthis lessoniana, C6-Sepia esculenta, O2-Amphioctopus aegina, S1-Loliolus uyii, S3-Uroteuthis chinensis, S4-Uroteuthis edulis [file peerj-09-11825-s002.zip › _Preprocessing_Upper_Beak/O2/U-l-O2-20.jpg]

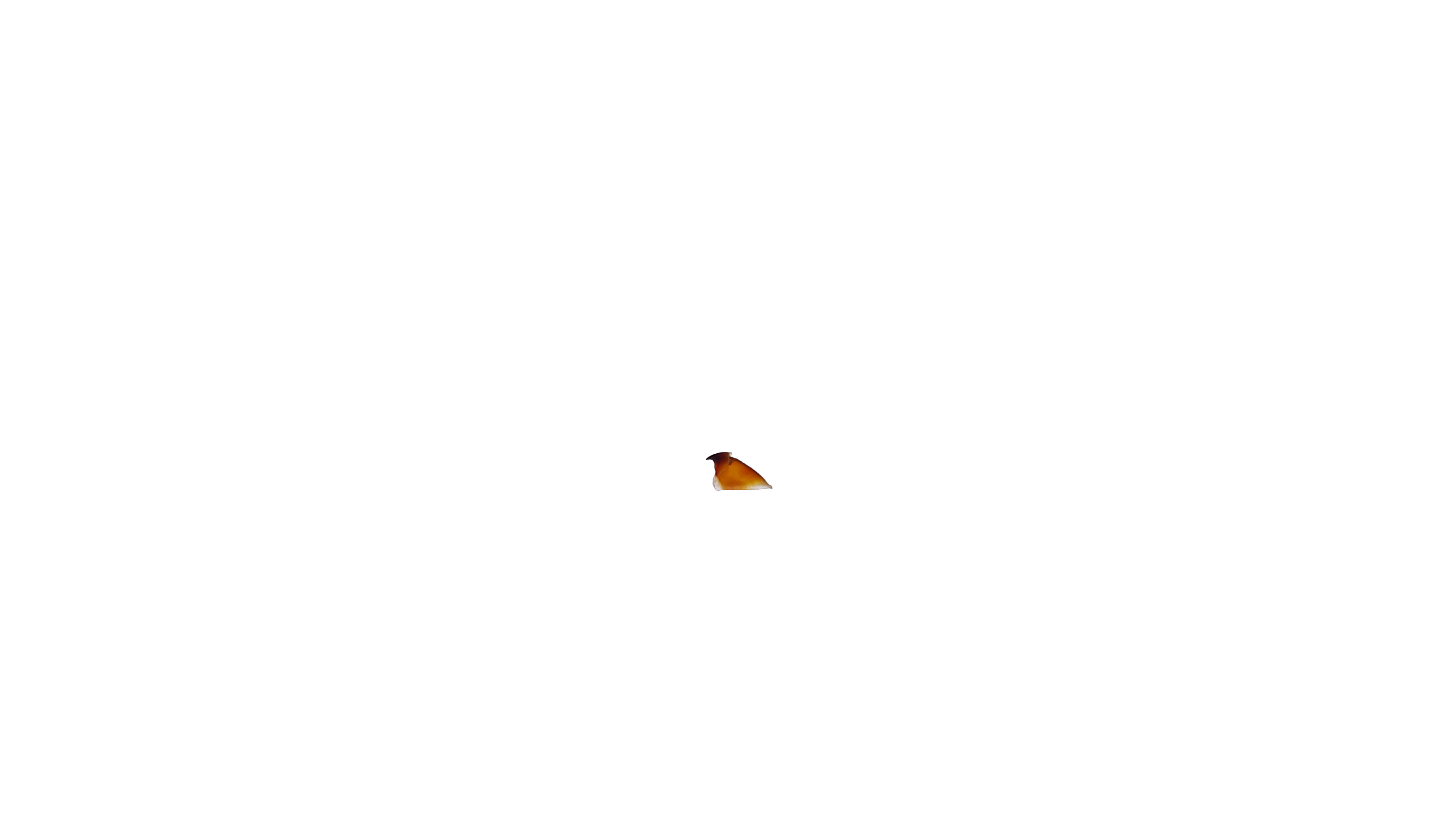

Supplement: Supplemental Information 2 — C2-Sepia aculeata, C3-Sepioteuthis lessoniana, C6-Sepia esculenta, O2-Amphioctopus aegina, S1-Loliolus uyii, S3-Uroteuthis chinensis, S4-Uroteuthis edulis [file peerj-09-11825-s002.zip › _Preprocessing_Upper_Beak/O2/U-l-O2-21.jpg]

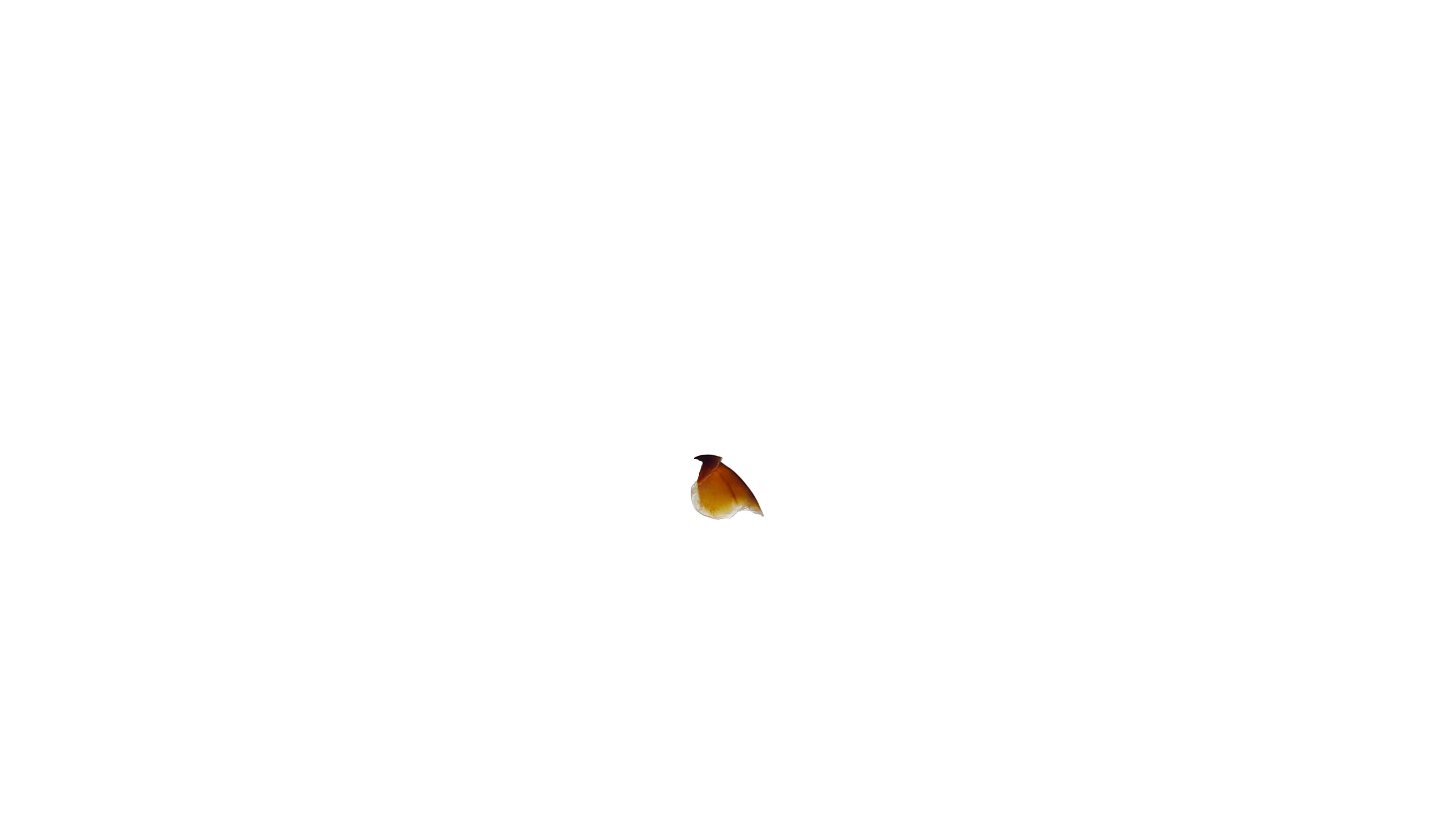

Supplement: Supplemental Information 2 — C2-Sepia aculeata, C3-Sepioteuthis lessoniana, C6-Sepia esculenta, O2-Amphioctopus aegina, S1-Loliolus uyii, S3-Uroteuthis chinensis, S4-Uroteuthis edulis [file peerj-09-11825-s002.zip › _Preprocessing_Upper_Beak/O2/U-l-O2-22.jpg]

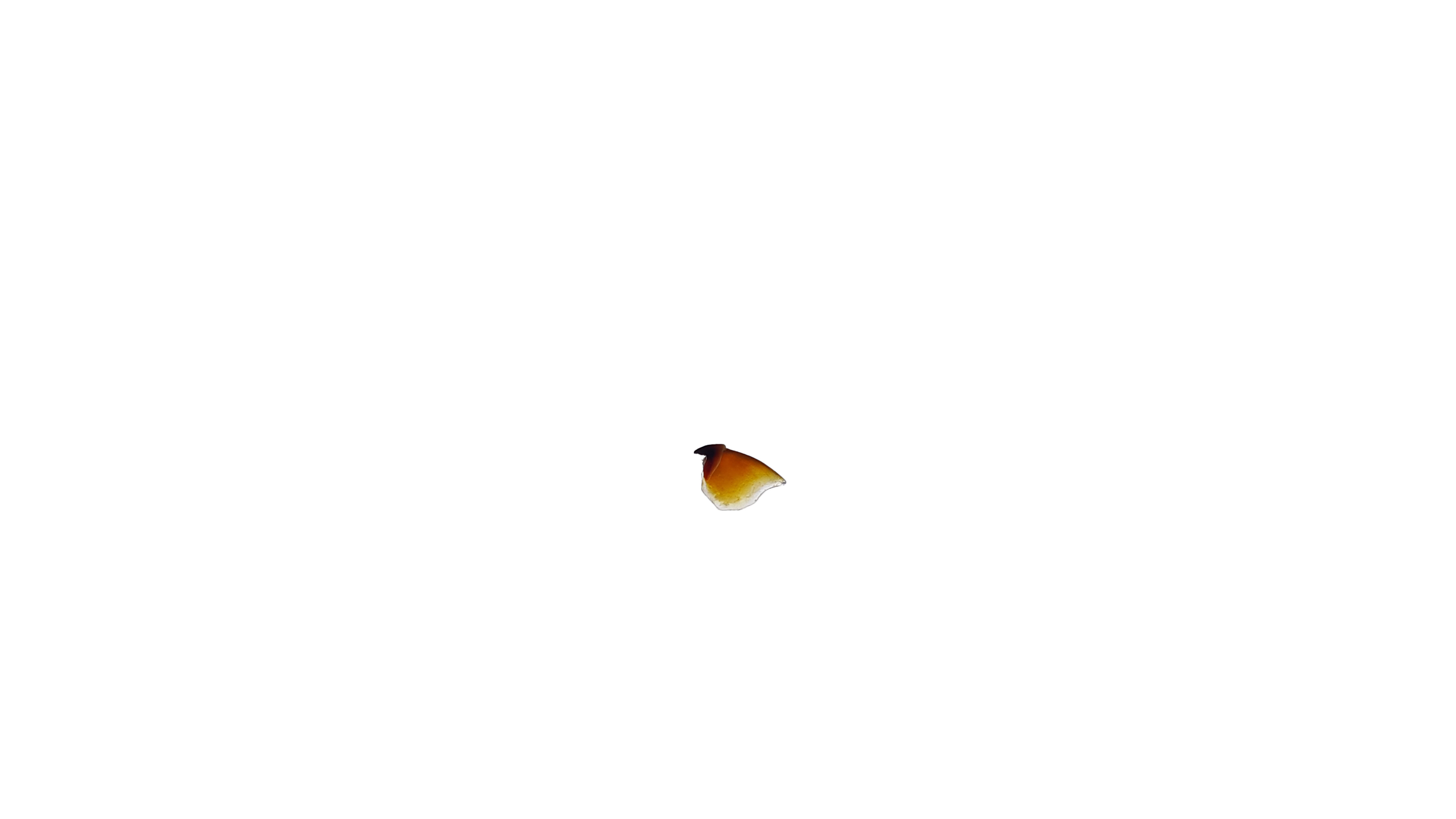

Supplement: Supplemental Information 2 — C2-Sepia aculeata, C3-Sepioteuthis lessoniana, C6-Sepia esculenta, O2-Amphioctopus aegina, S1-Loliolus uyii, S3-Uroteuthis chinensis, S4-Uroteuthis edulis [file peerj-09-11825-s002.zip › _Preprocessing_Upper_Beak/O2/U-l-O2-23.jpg]

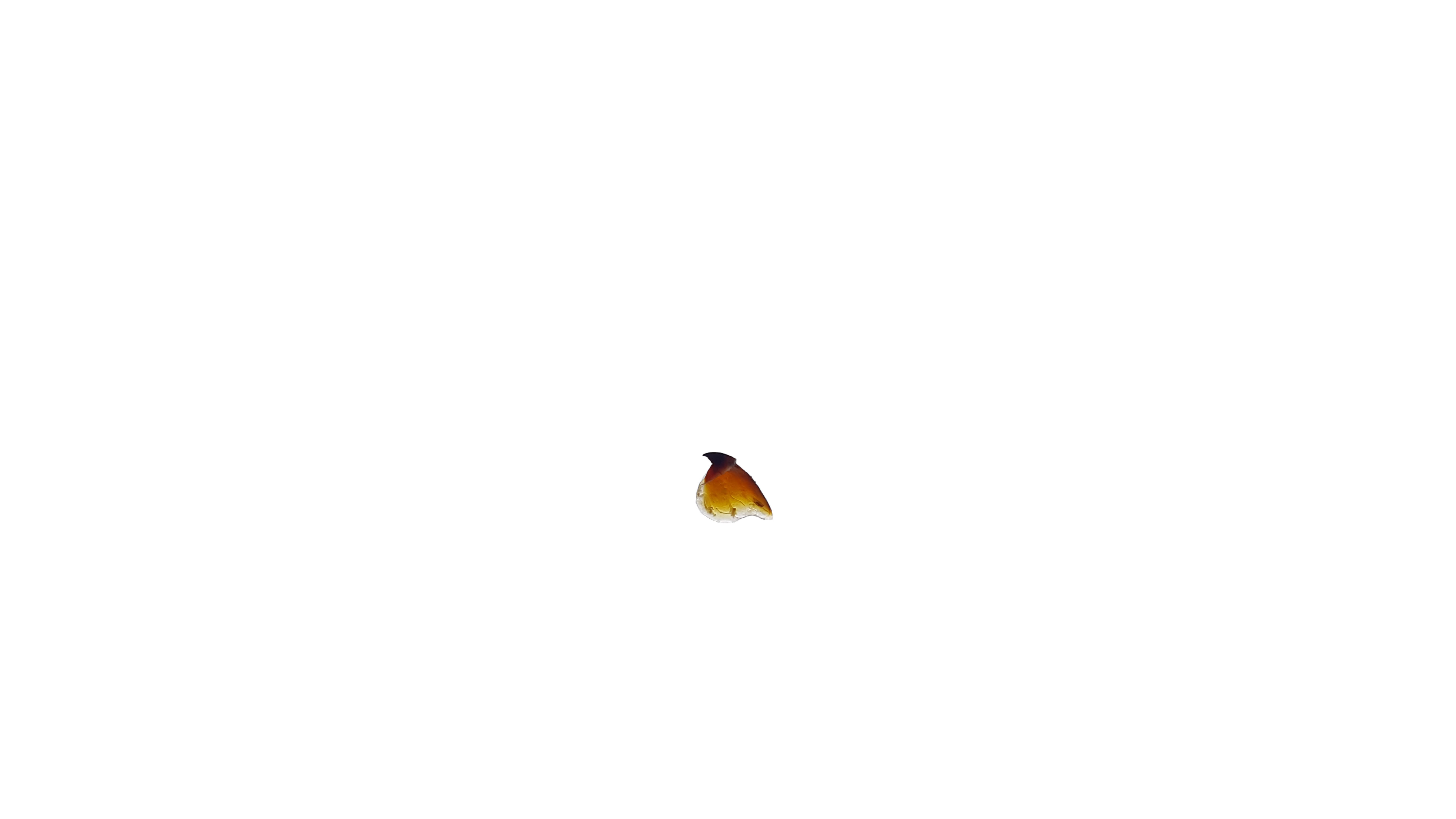

Supplement: Supplemental Information 2 — C2-Sepia aculeata, C3-Sepioteuthis lessoniana, C6-Sepia esculenta, O2-Amphioctopus aegina, S1-Loliolus uyii, S3-Uroteuthis chinensis, S4-Uroteuthis edulis [file peerj-09-11825-s002.zip › _Preprocessing_Upper_Beak/O2/U-l-O2-24.jpg]

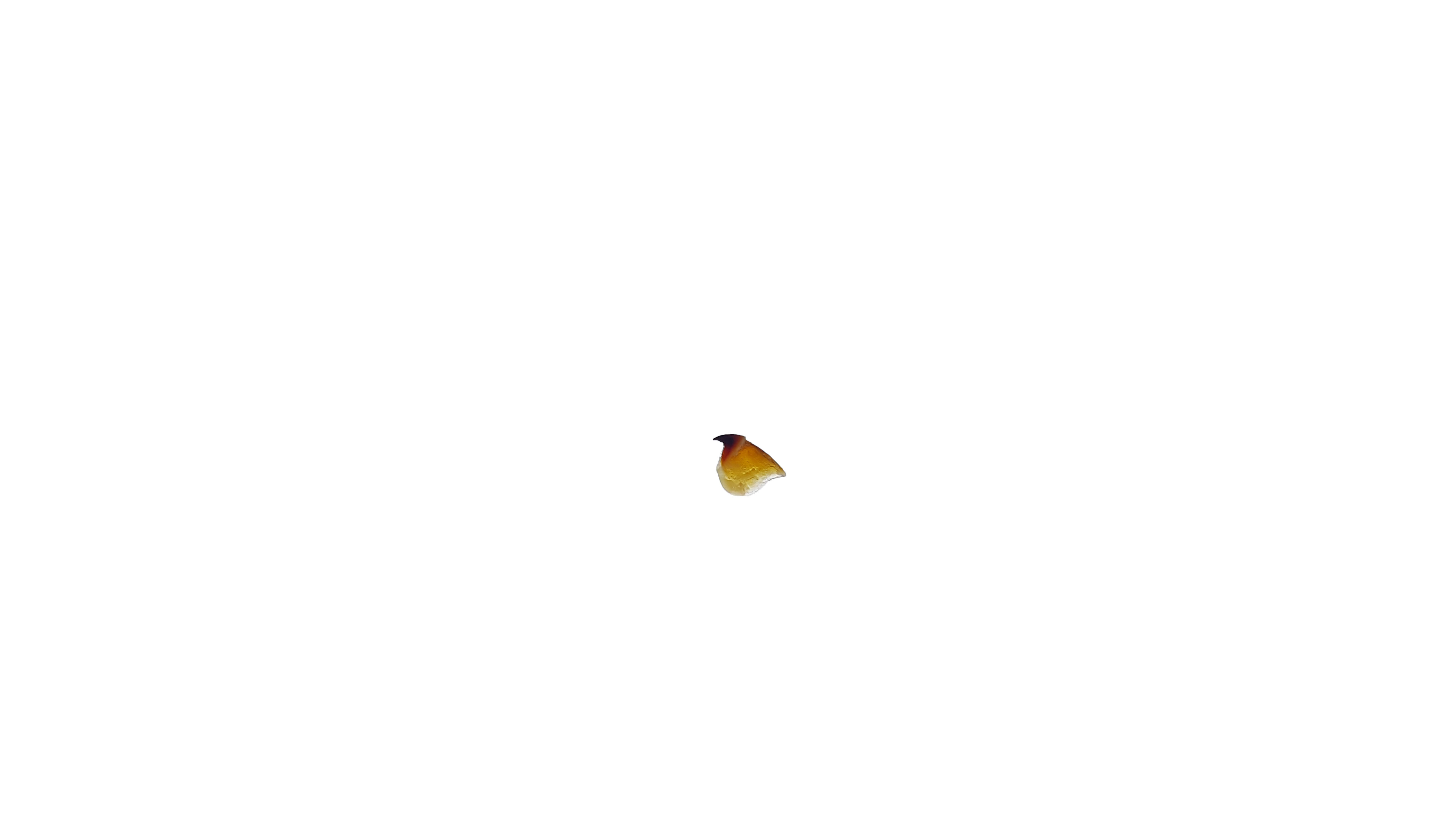

Supplement: Supplemental Information 2 — C2-Sepia aculeata, C3-Sepioteuthis lessoniana, C6-Sepia esculenta, O2-Amphioctopus aegina, S1-Loliolus uyii, S3-Uroteuthis chinensis, S4-Uroteuthis edulis [file peerj-09-11825-s002.zip › _Preprocessing_Upper_Beak/O2/U-l-O2-25.jpg]

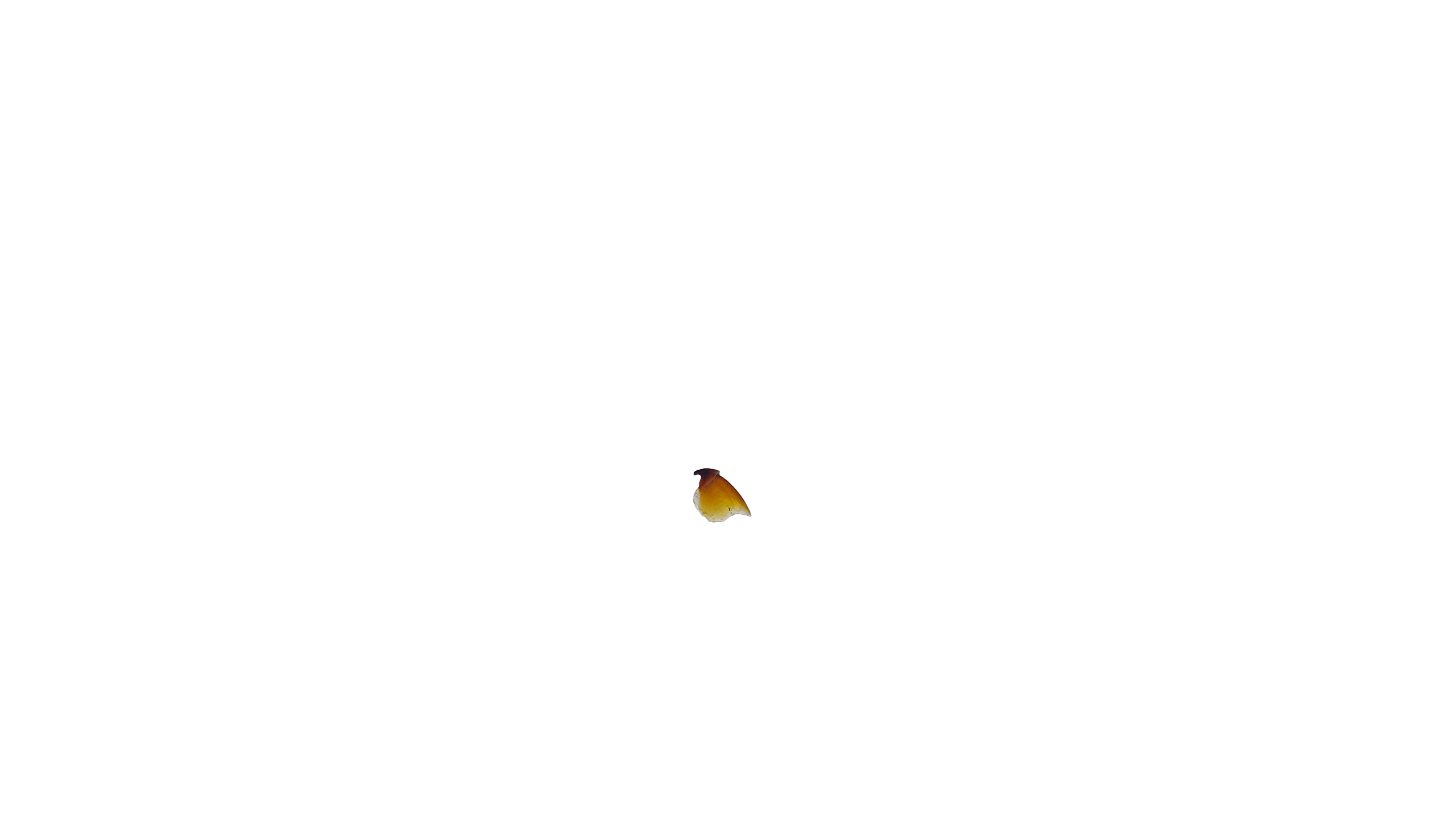

Supplement: Supplemental Information 2 — C2-Sepia aculeata, C3-Sepioteuthis lessoniana, C6-Sepia esculenta, O2-Amphioctopus aegina, S1-Loliolus uyii, S3-Uroteuthis chinensis, S4-Uroteuthis edulis [file peerj-09-11825-s002.zip › _Preprocessing_Upper_Beak/O2/U-l-O2-26.jpg]

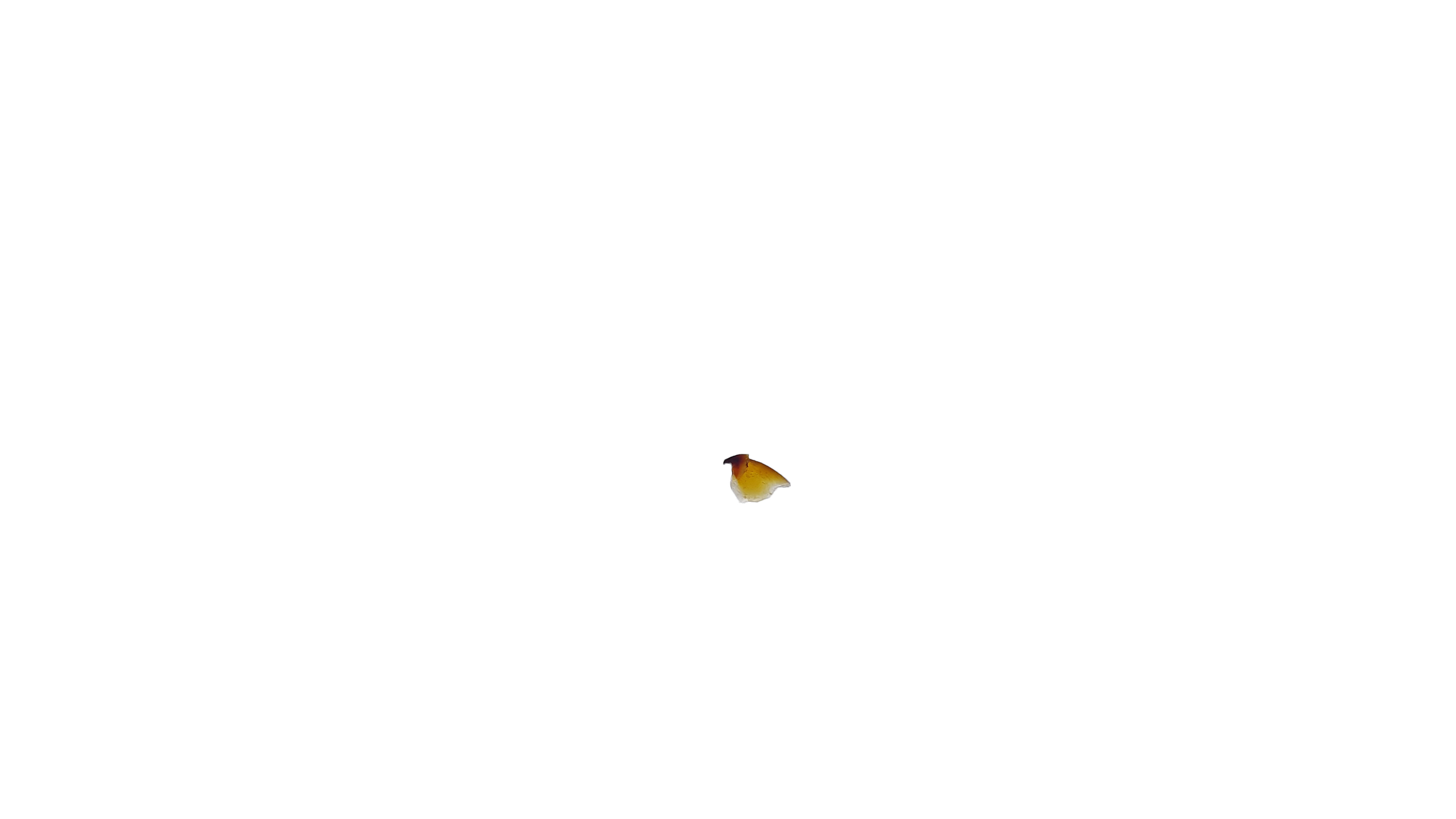

Supplement: Supplemental Information 2 — C2-Sepia aculeata, C3-Sepioteuthis lessoniana, C6-Sepia esculenta, O2-Amphioctopus aegina, S1-Loliolus uyii, S3-Uroteuthis chinensis, S4-Uroteuthis edulis [file peerj-09-11825-s002.zip › _Preprocessing_Upper_Beak/O2/U-l-O2-27.jpg]

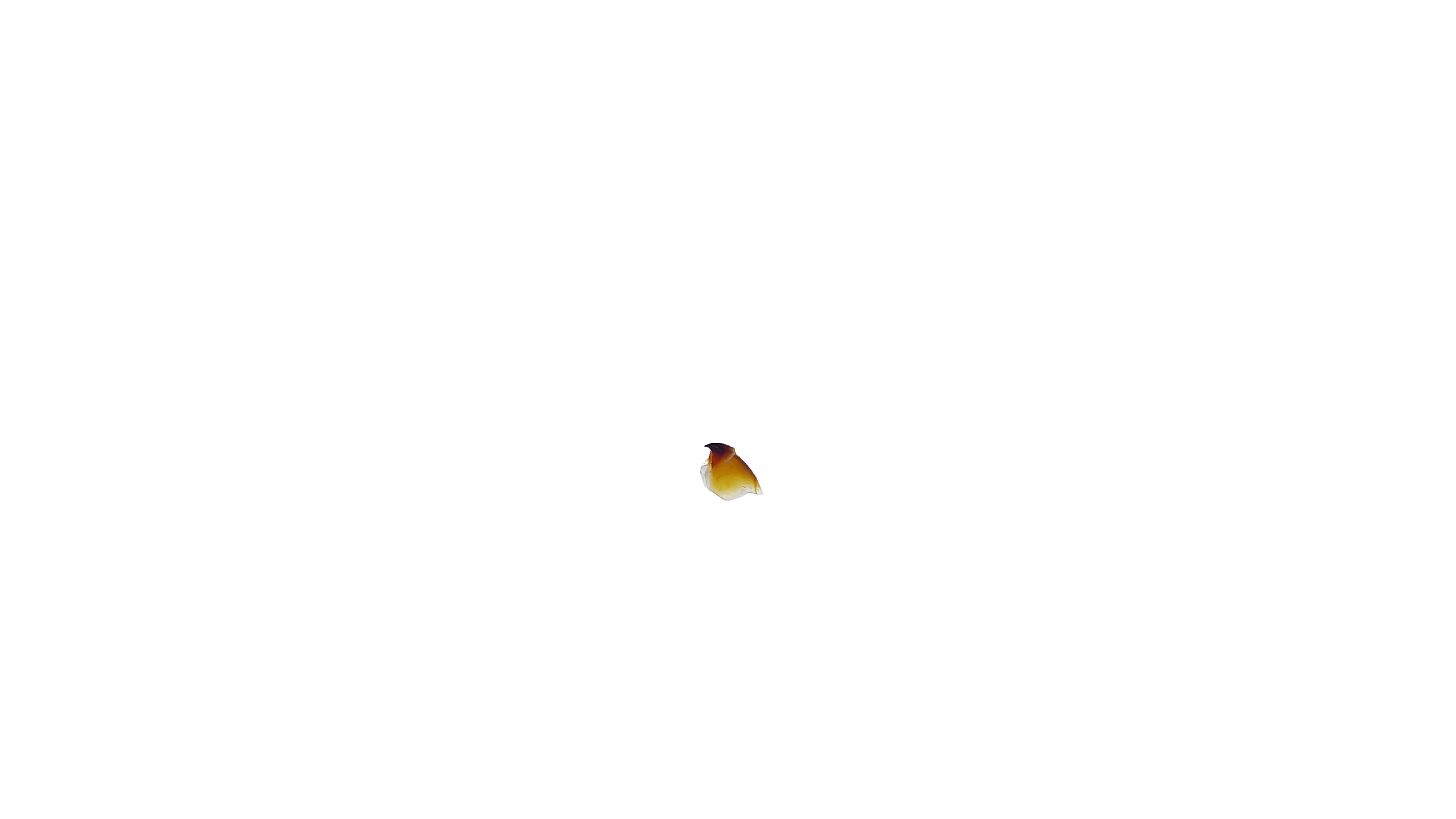

Supplement: Supplemental Information 2 — C2-Sepia aculeata, C3-Sepioteuthis lessoniana, C6-Sepia esculenta, O2-Amphioctopus aegina, S1-Loliolus uyii, S3-Uroteuthis chinensis, S4-Uroteuthis edulis [file peerj-09-11825-s002.zip › _Preprocessing_Upper_Beak/O2/U-l-O2-28.jpg]

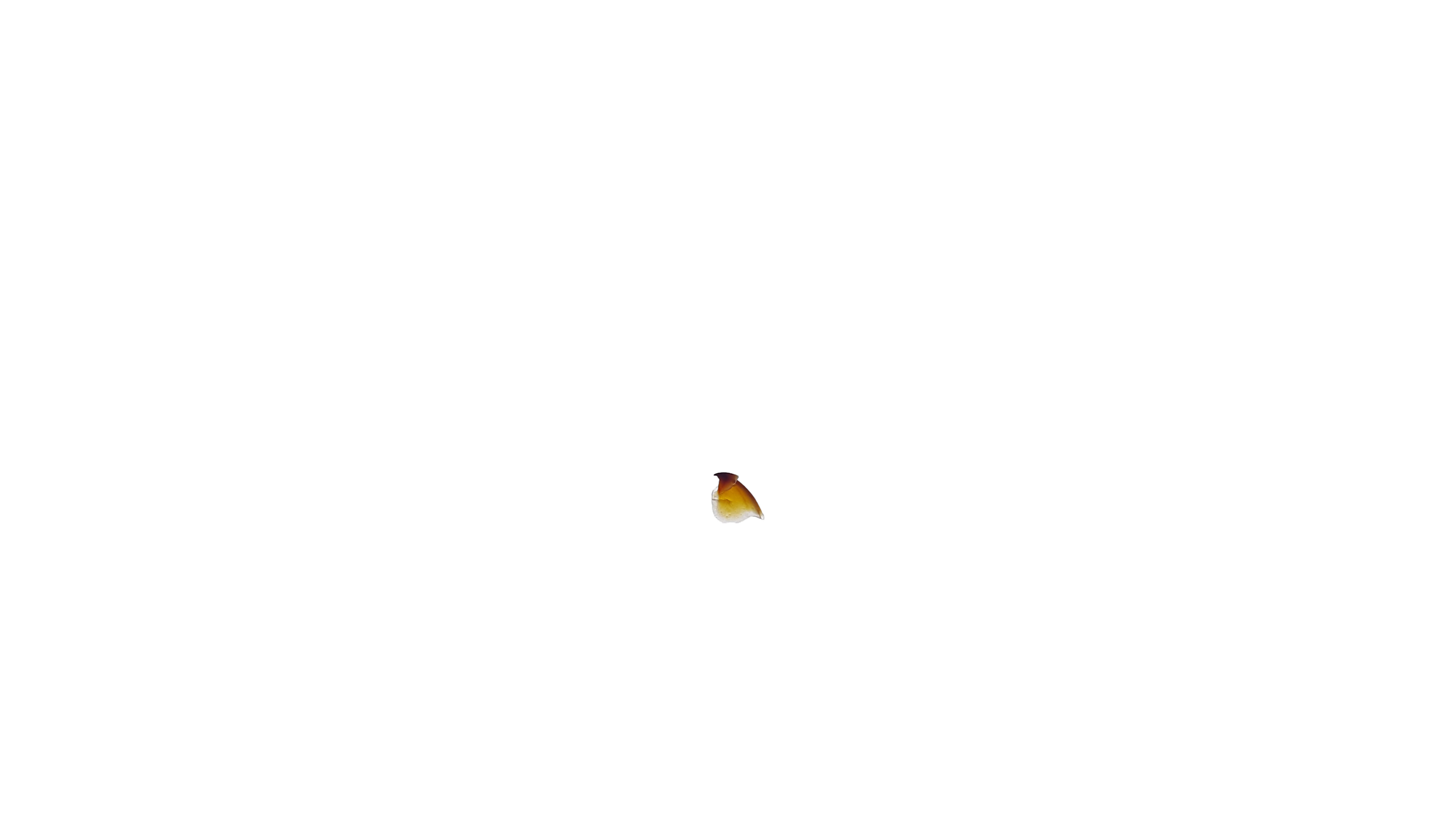

Supplement: Supplemental Information 2 — C2-Sepia aculeata, C3-Sepioteuthis lessoniana, C6-Sepia esculenta, O2-Amphioctopus aegina, S1-Loliolus uyii, S3-Uroteuthis chinensis, S4-Uroteuthis edulis [file peerj-09-11825-s002.zip › _Preprocessing_Upper_Beak/O2/U-l-O2-29.jpg]

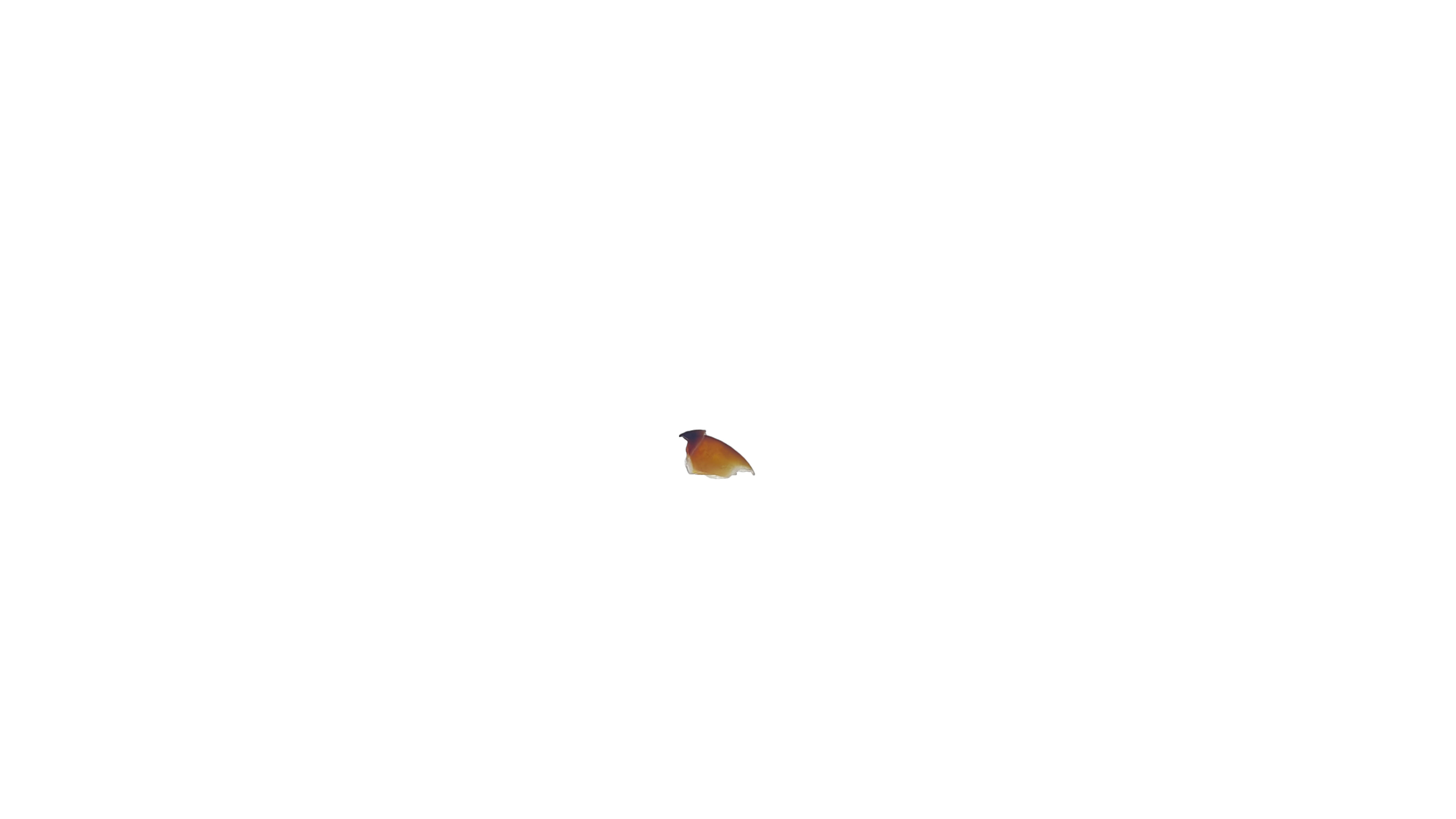

Supplement: Supplemental Information 2 — C2-Sepia aculeata, C3-Sepioteuthis lessoniana, C6-Sepia esculenta, O2-Amphioctopus aegina, S1-Loliolus uyii, S3-Uroteuthis chinensis, S4-Uroteuthis edulis [file peerj-09-11825-s002.zip › _Preprocessing_Upper_Beak/O2/U-l-O2-3.jpg]

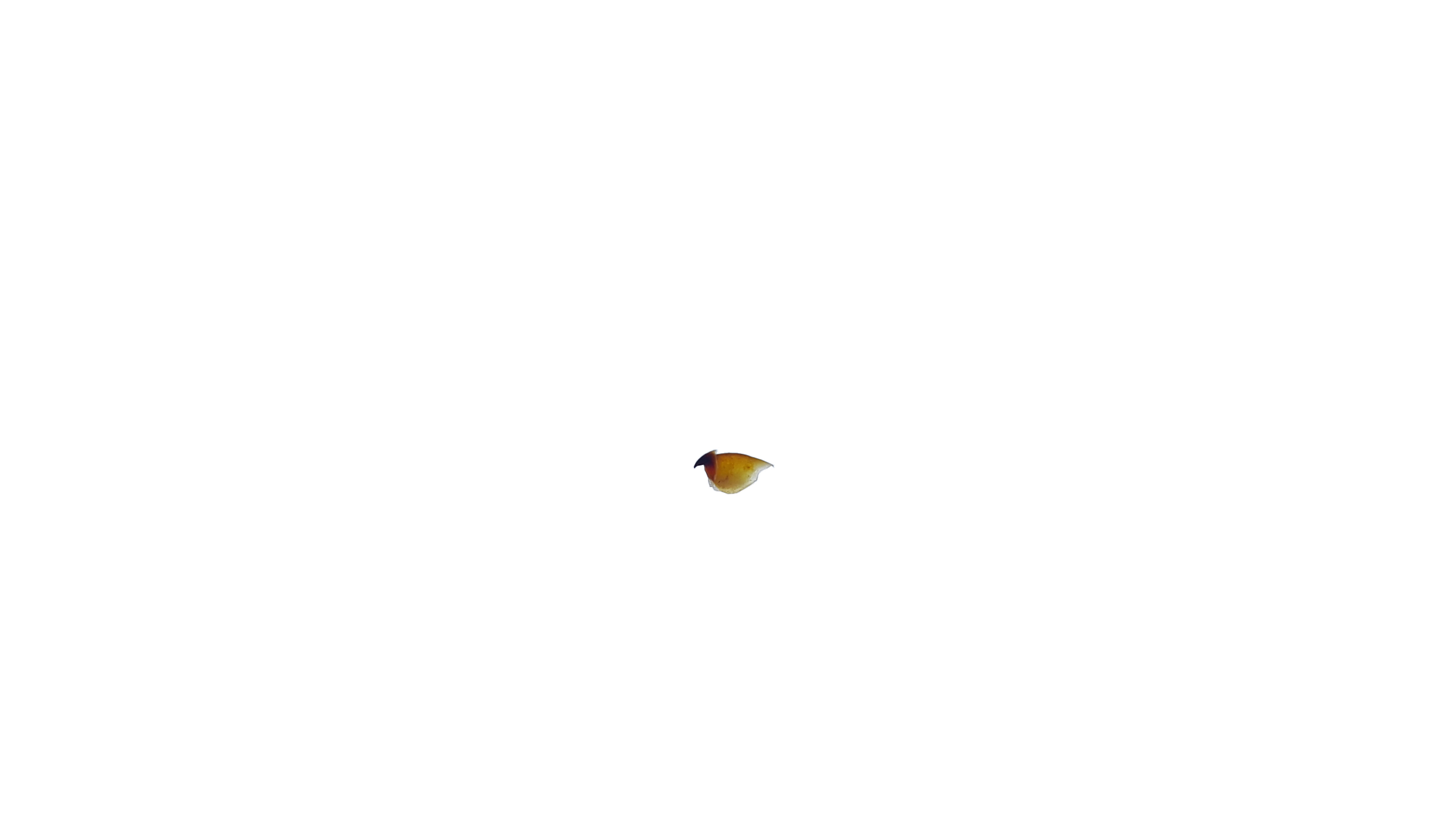

Supplement: Supplemental Information 2 — C2-Sepia aculeata, C3-Sepioteuthis lessoniana, C6-Sepia esculenta, O2-Amphioctopus aegina, S1-Loliolus uyii, S3-Uroteuthis chinensis, S4-Uroteuthis edulis [file peerj-09-11825-s002.zip › _Preprocessing_Upper_Beak/O2/U-l-O2-4.jpg]

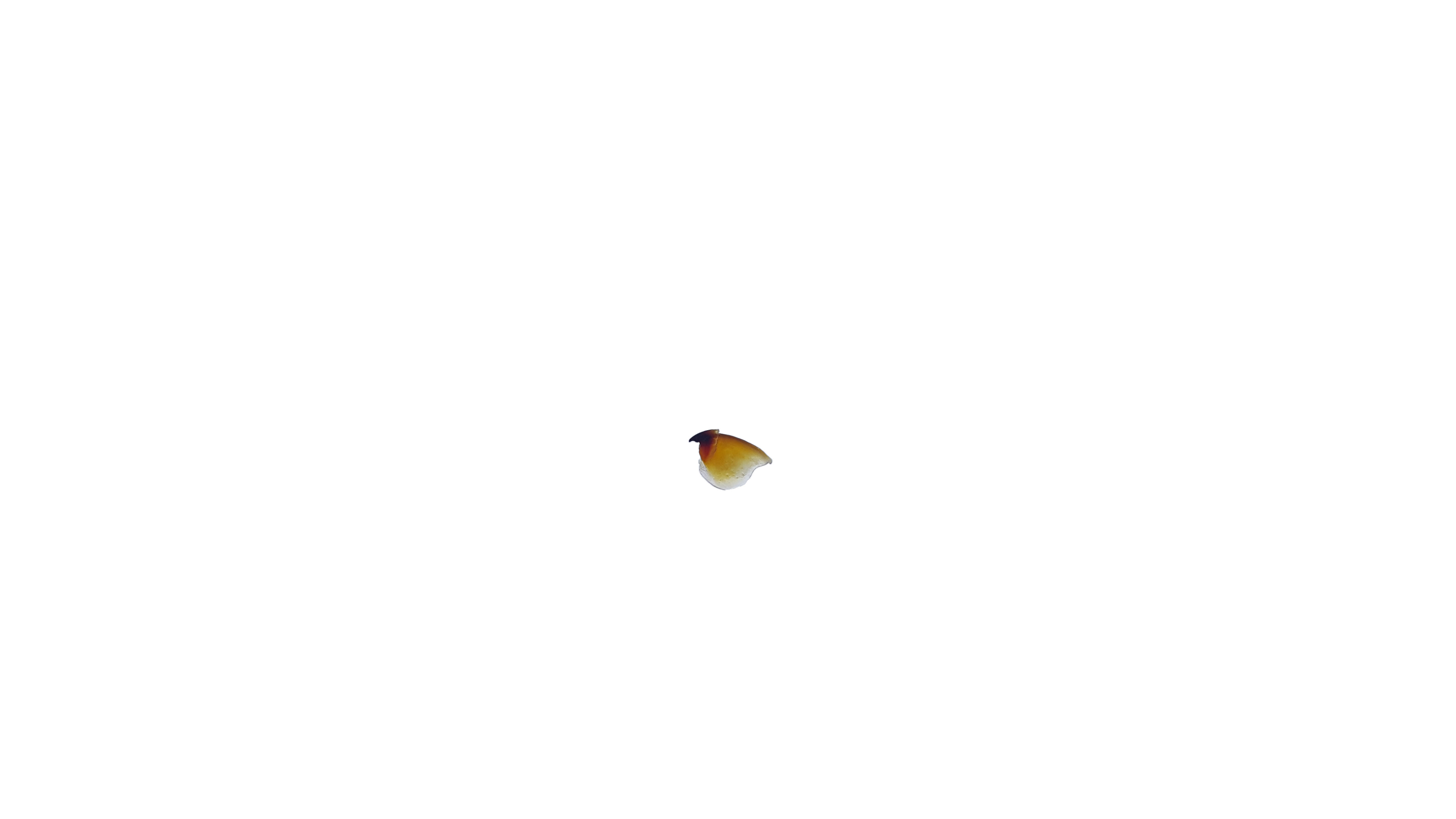

Supplement: Supplemental Information 2 — C2-Sepia aculeata, C3-Sepioteuthis lessoniana, C6-Sepia esculenta, O2-Amphioctopus aegina, S1-Loliolus uyii, S3-Uroteuthis chinensis, S4-Uroteuthis edulis [file peerj-09-11825-s002.zip › _Preprocessing_Upper_Beak/O2/U-l-O2-5.jpg]

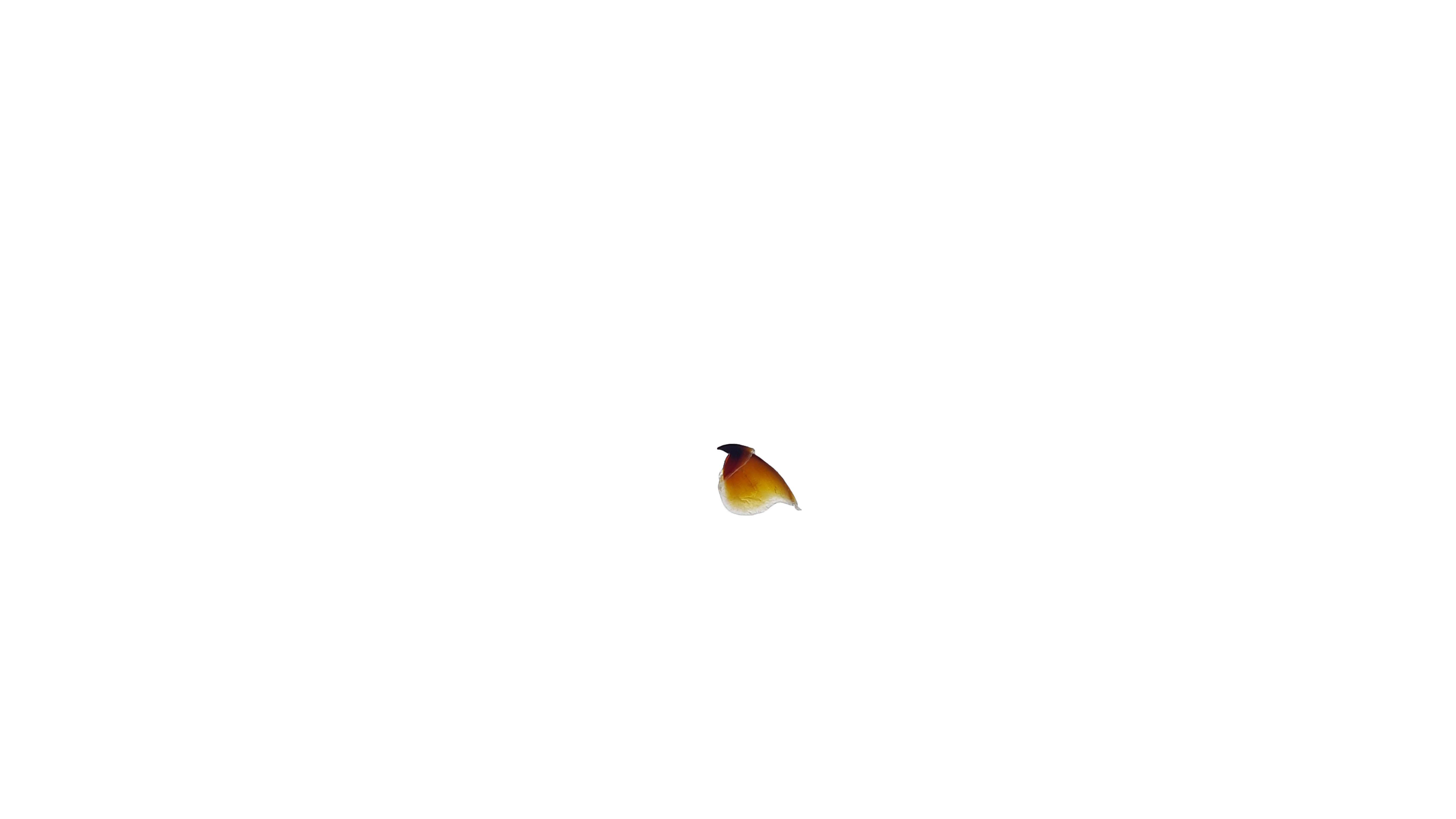

Supplement: Supplemental Information 2 — C2-Sepia aculeata, C3-Sepioteuthis lessoniana, C6-Sepia esculenta, O2-Amphioctopus aegina, S1-Loliolus uyii, S3-Uroteuthis chinensis, S4-Uroteuthis edulis [file peerj-09-11825-s002.zip › _Preprocessing_Upper_Beak/O2/U-l-O2-6.jpg]

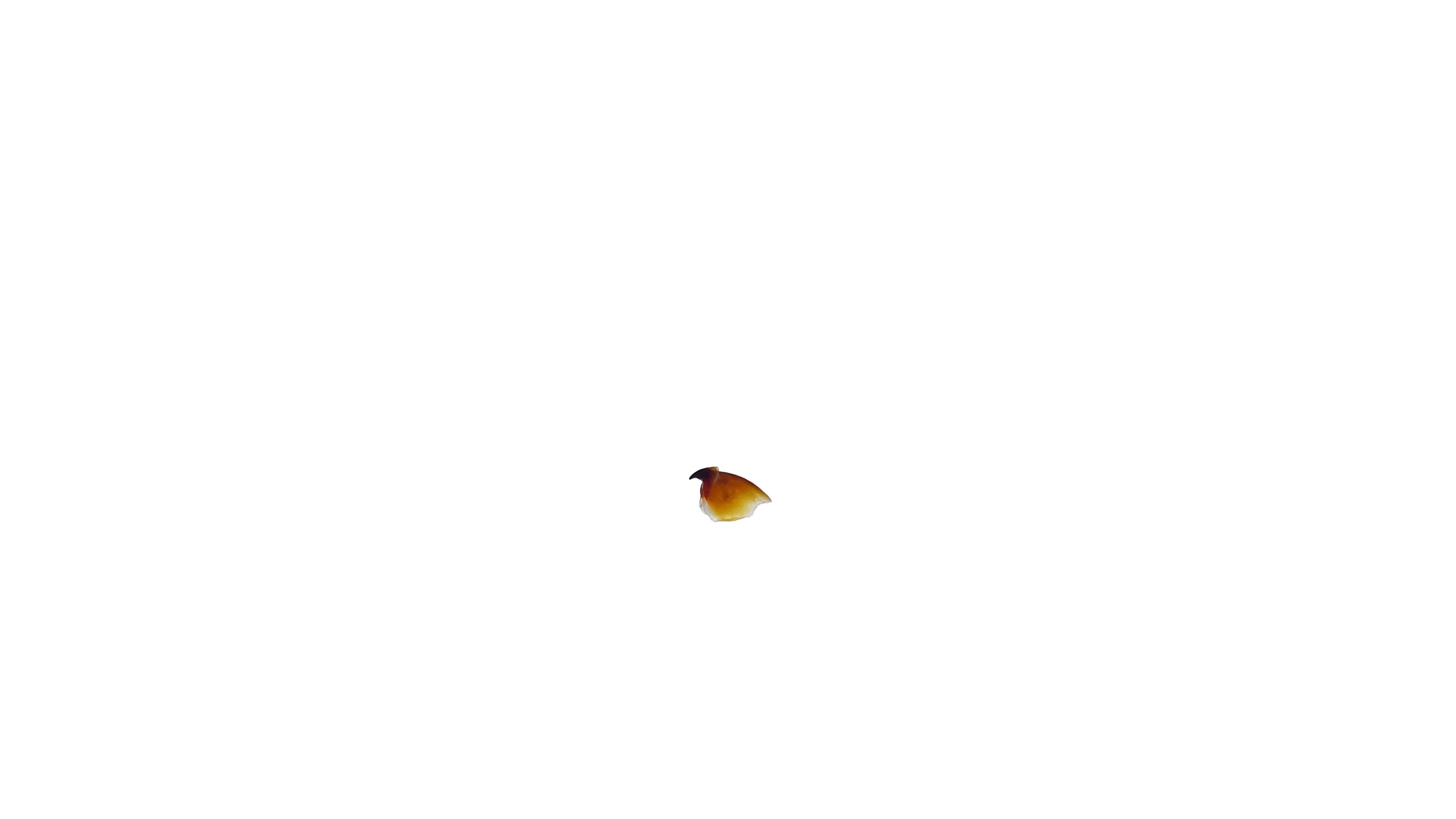

Supplement: Supplemental Information 2 — C2-Sepia aculeata, C3-Sepioteuthis lessoniana, C6-Sepia esculenta, O2-Amphioctopus aegina, S1-Loliolus uyii, S3-Uroteuthis chinensis, S4-Uroteuthis edulis [file peerj-09-11825-s002.zip › _Preprocessing_Upper_Beak/O2/U-l-O2-7.jpg]

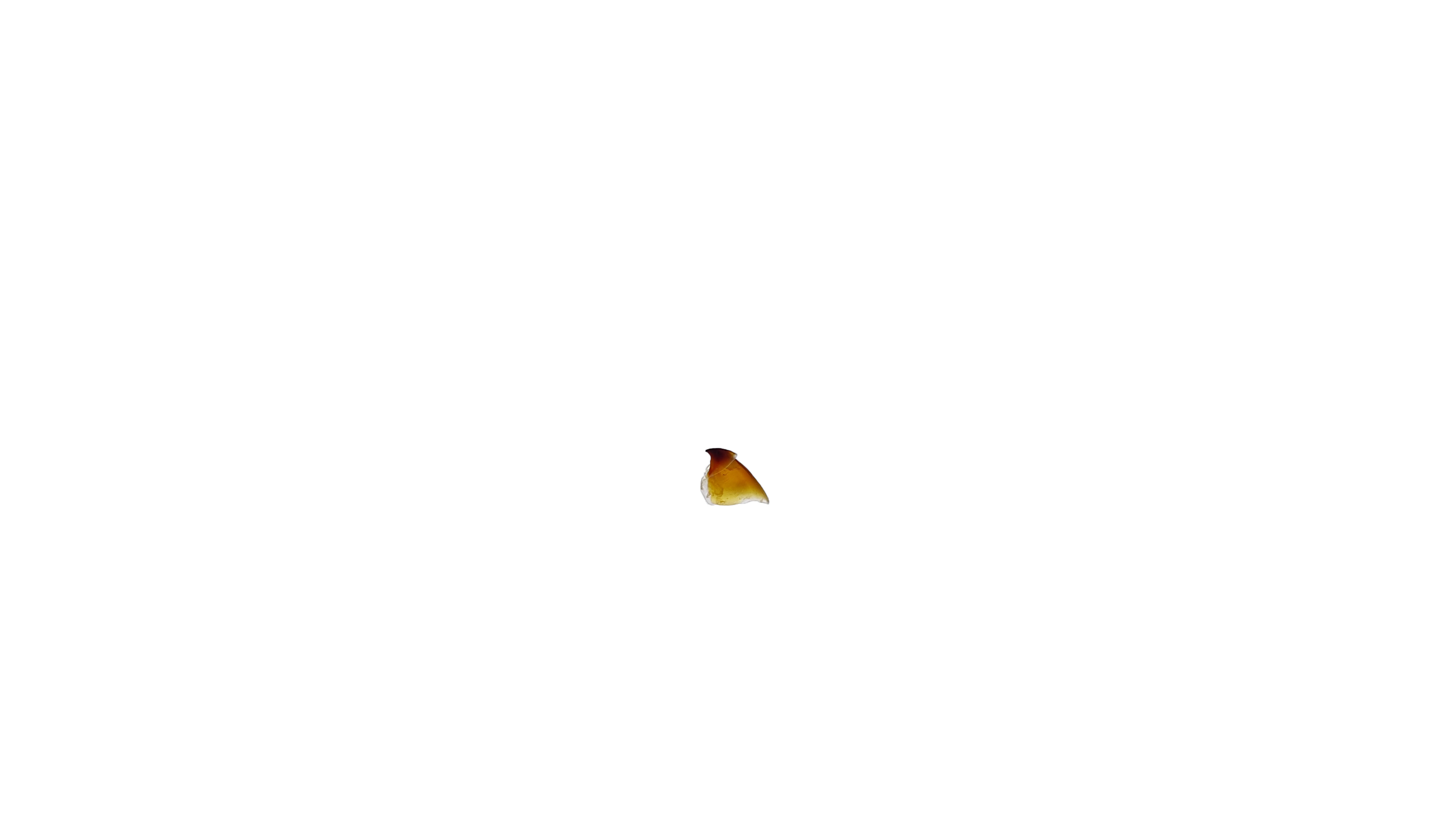

Supplement: Supplemental Information 2 — C2-Sepia aculeata, C3-Sepioteuthis lessoniana, C6-Sepia esculenta, O2-Amphioctopus aegina, S1-Loliolus uyii, S3-Uroteuthis chinensis, S4-Uroteuthis edulis [file peerj-09-11825-s002.zip › _Preprocessing_Upper_Beak/O2/U-l-O2-8.jpg]

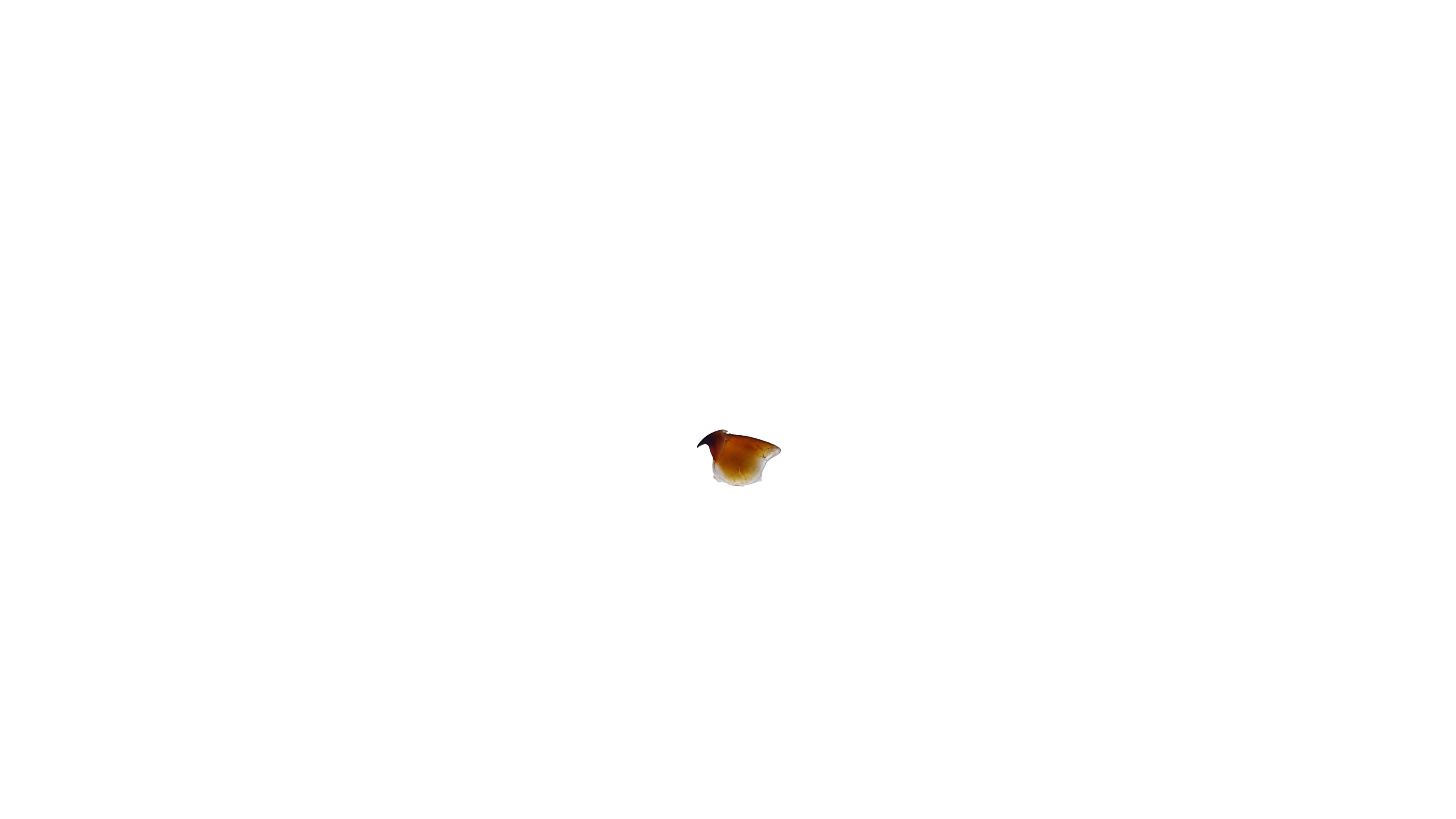

Supplement: Supplemental Information 2 — C2-Sepia aculeata, C3-Sepioteuthis lessoniana, C6-Sepia esculenta, O2-Amphioctopus aegina, S1-Loliolus uyii, S3-Uroteuthis chinensis, S4-Uroteuthis edulis [file peerj-09-11825-s002.zip › _Preprocessing_Upper_Beak/O2/U-l-O2-9.jpg]

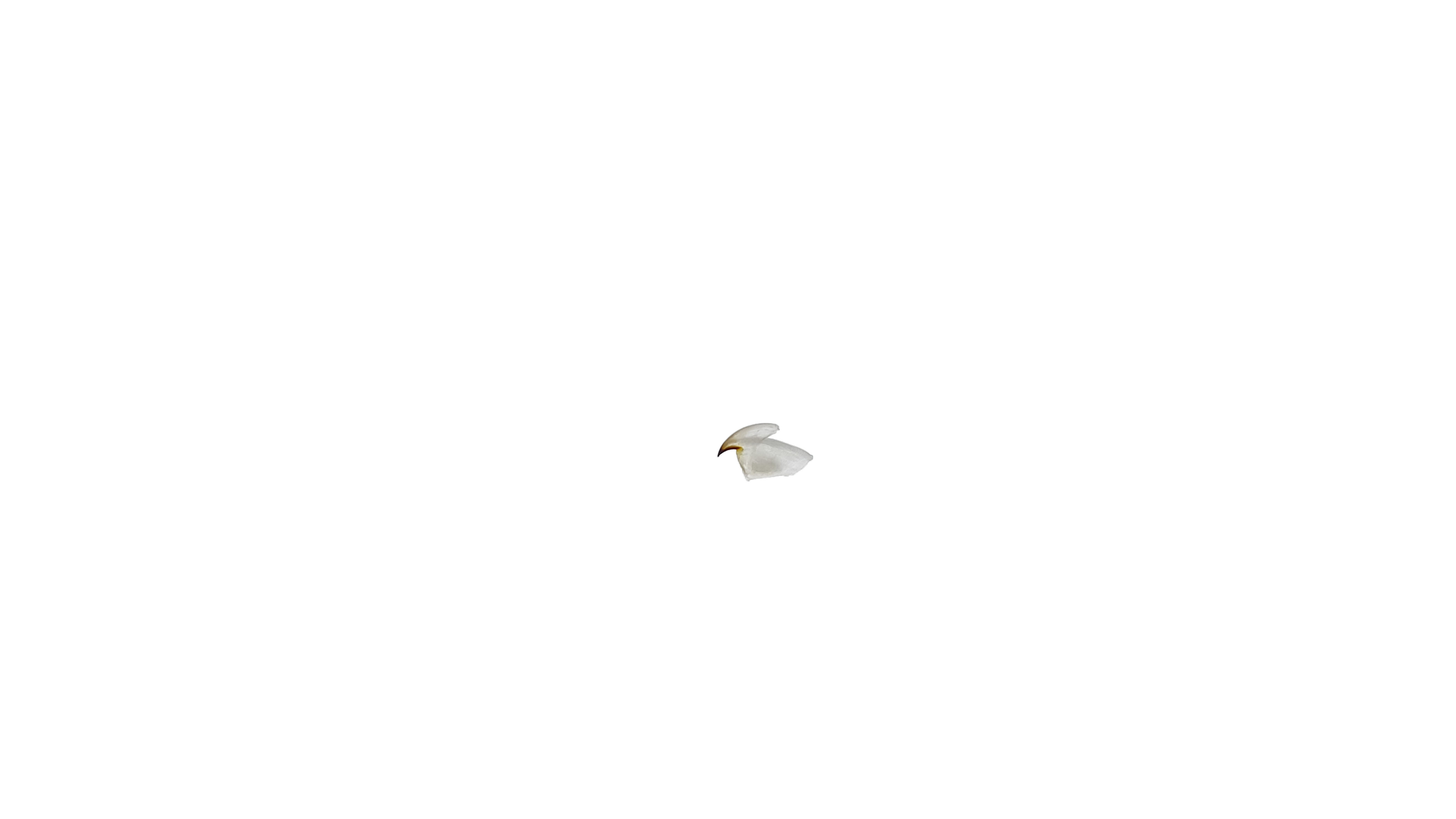

Supplement: Supplemental Information 2 — C2-Sepia aculeata, C3-Sepioteuthis lessoniana, C6-Sepia esculenta, O2-Amphioctopus aegina, S1-Loliolus uyii, S3-Uroteuthis chinensis, S4-Uroteuthis edulis [file peerj-09-11825-s002.zip › _Preprocessing_Upper_Beak/S1/U-l-S1-1.jpg]

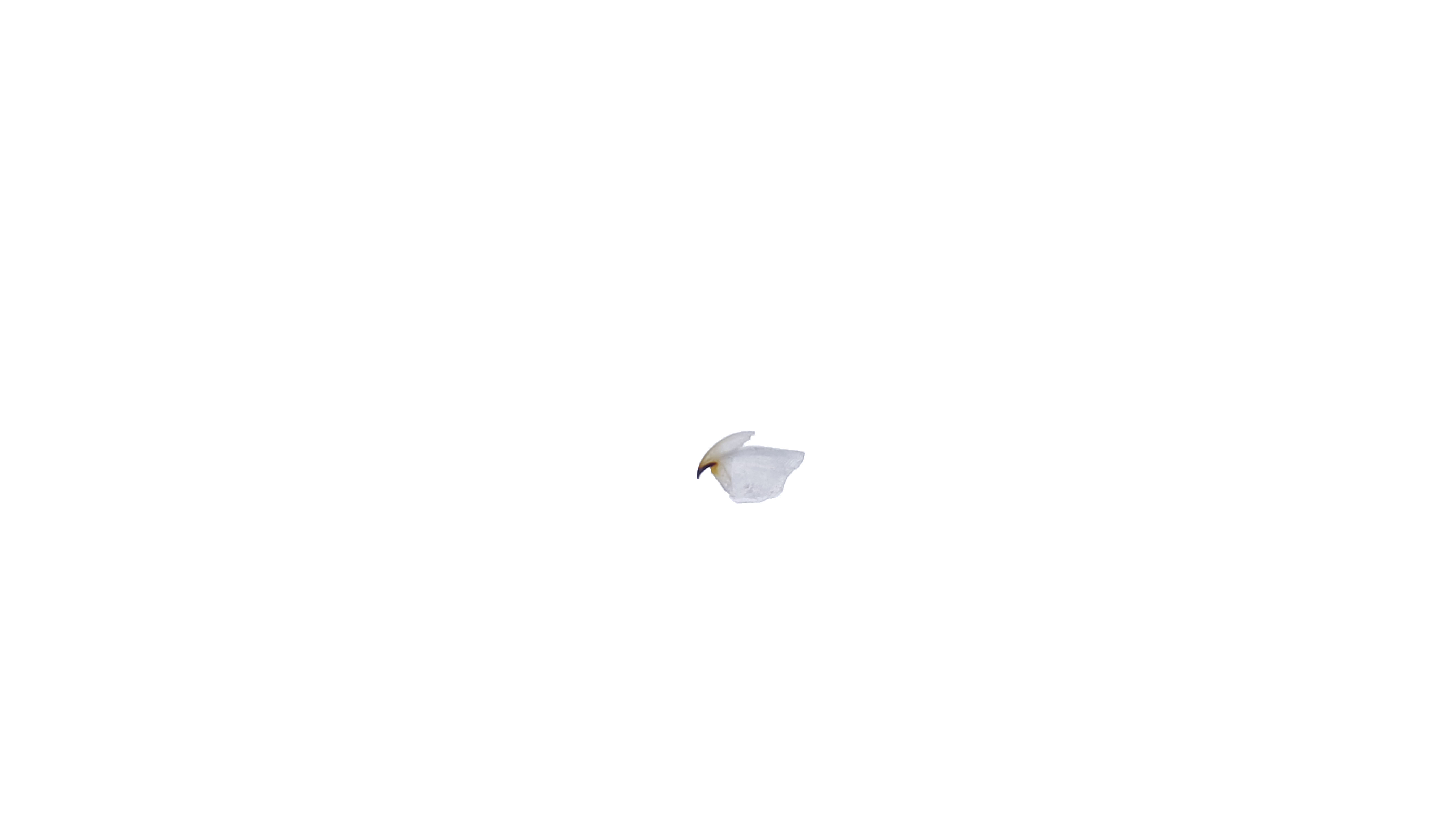

Supplement: Supplemental Information 2 — C2-Sepia aculeata, C3-Sepioteuthis lessoniana, C6-Sepia esculenta, O2-Amphioctopus aegina, S1-Loliolus uyii, S3-Uroteuthis chinensis, S4-Uroteuthis edulis [file peerj-09-11825-s002.zip › _Preprocessing_Upper_Beak/S1/U-l-S1-10.jpg]

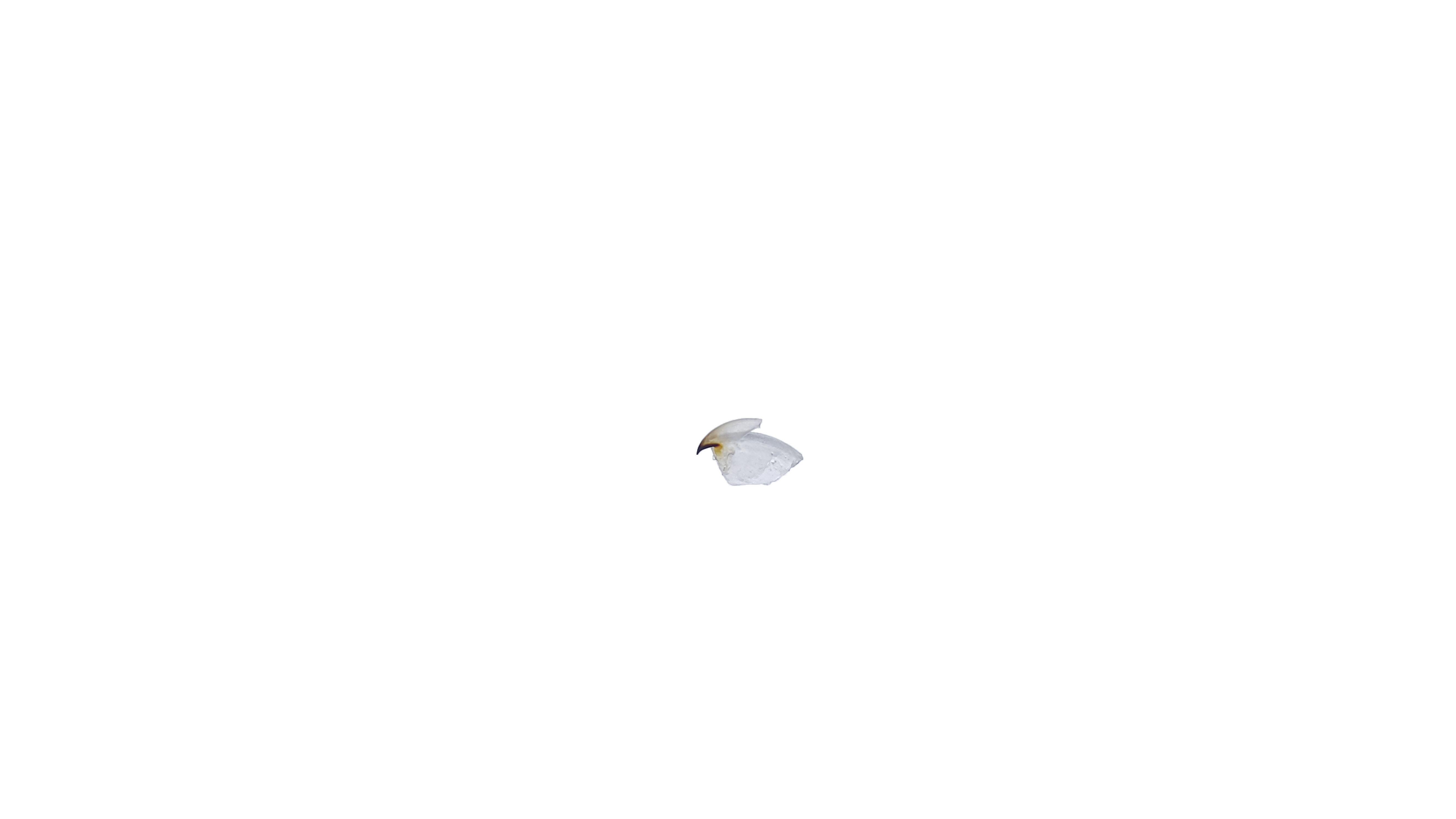

Supplement: Supplemental Information 2 — C2-Sepia aculeata, C3-Sepioteuthis lessoniana, C6-Sepia esculenta, O2-Amphioctopus aegina, S1-Loliolus uyii, S3-Uroteuthis chinensis, S4-Uroteuthis edulis [file peerj-09-11825-s002.zip › _Preprocessing_Upper_Beak/S1/U-l-S1-11.jpg]

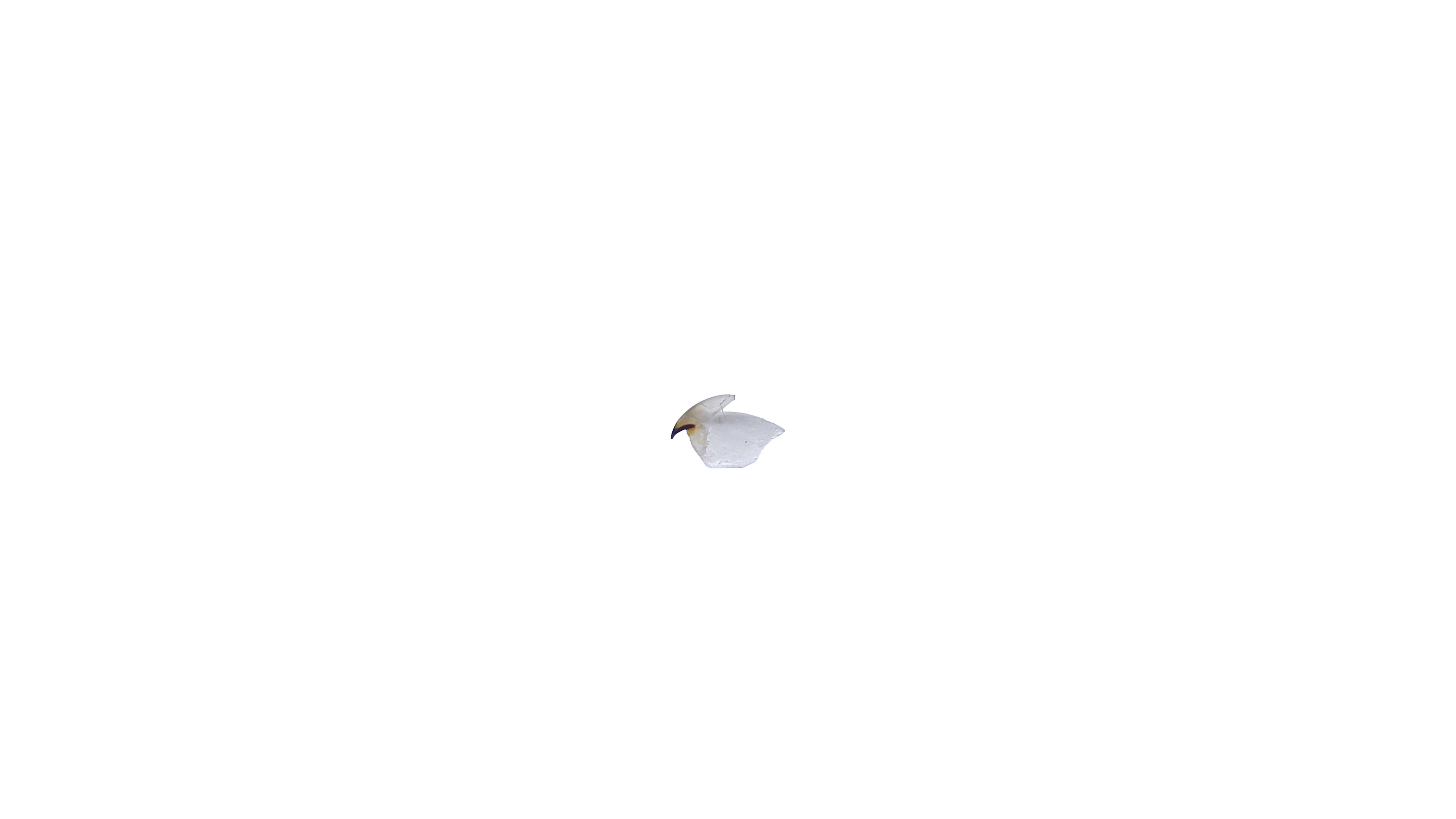

Supplement: Supplemental Information 2 — C2-Sepia aculeata, C3-Sepioteuthis lessoniana, C6-Sepia esculenta, O2-Amphioctopus aegina, S1-Loliolus uyii, S3-Uroteuthis chinensis, S4-Uroteuthis edulis [file peerj-09-11825-s002.zip › _Preprocessing_Upper_Beak/S1/U-l-S1-12.jpg]

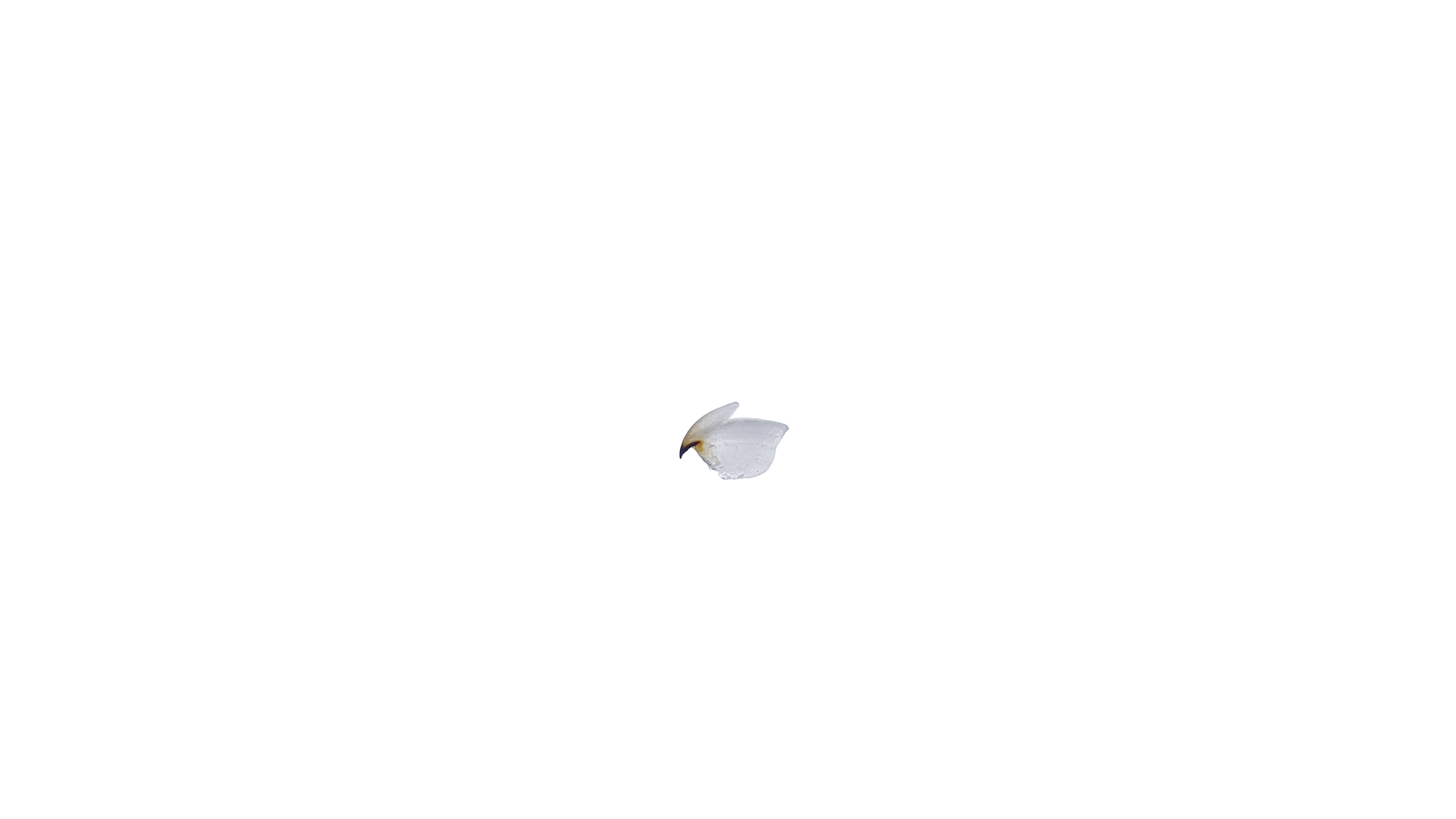

Supplement: Supplemental Information 2 — C2-Sepia aculeata, C3-Sepioteuthis lessoniana, C6-Sepia esculenta, O2-Amphioctopus aegina, S1-Loliolus uyii, S3-Uroteuthis chinensis, S4-Uroteuthis edulis [file peerj-09-11825-s002.zip › _Preprocessing_Upper_Beak/S1/U-l-S1-13.jpg]

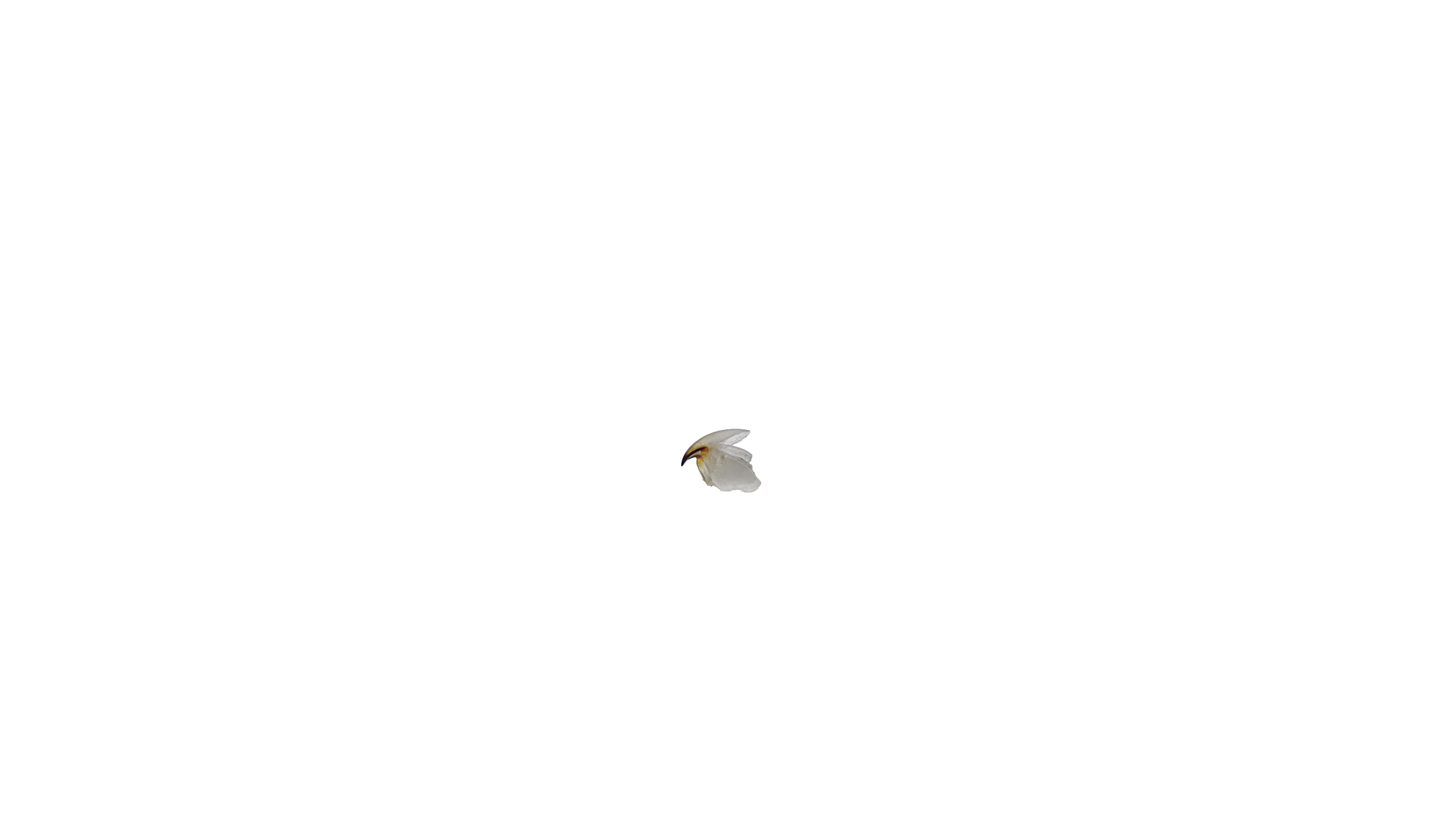

Supplement: Supplemental Information 2 — C2-Sepia aculeata, C3-Sepioteuthis lessoniana, C6-Sepia esculenta, O2-Amphioctopus aegina, S1-Loliolus uyii, S3-Uroteuthis chinensis, S4-Uroteuthis edulis [file peerj-09-11825-s002.zip › _Preprocessing_Upper_Beak/S1/U-l-S1-14.jpg]

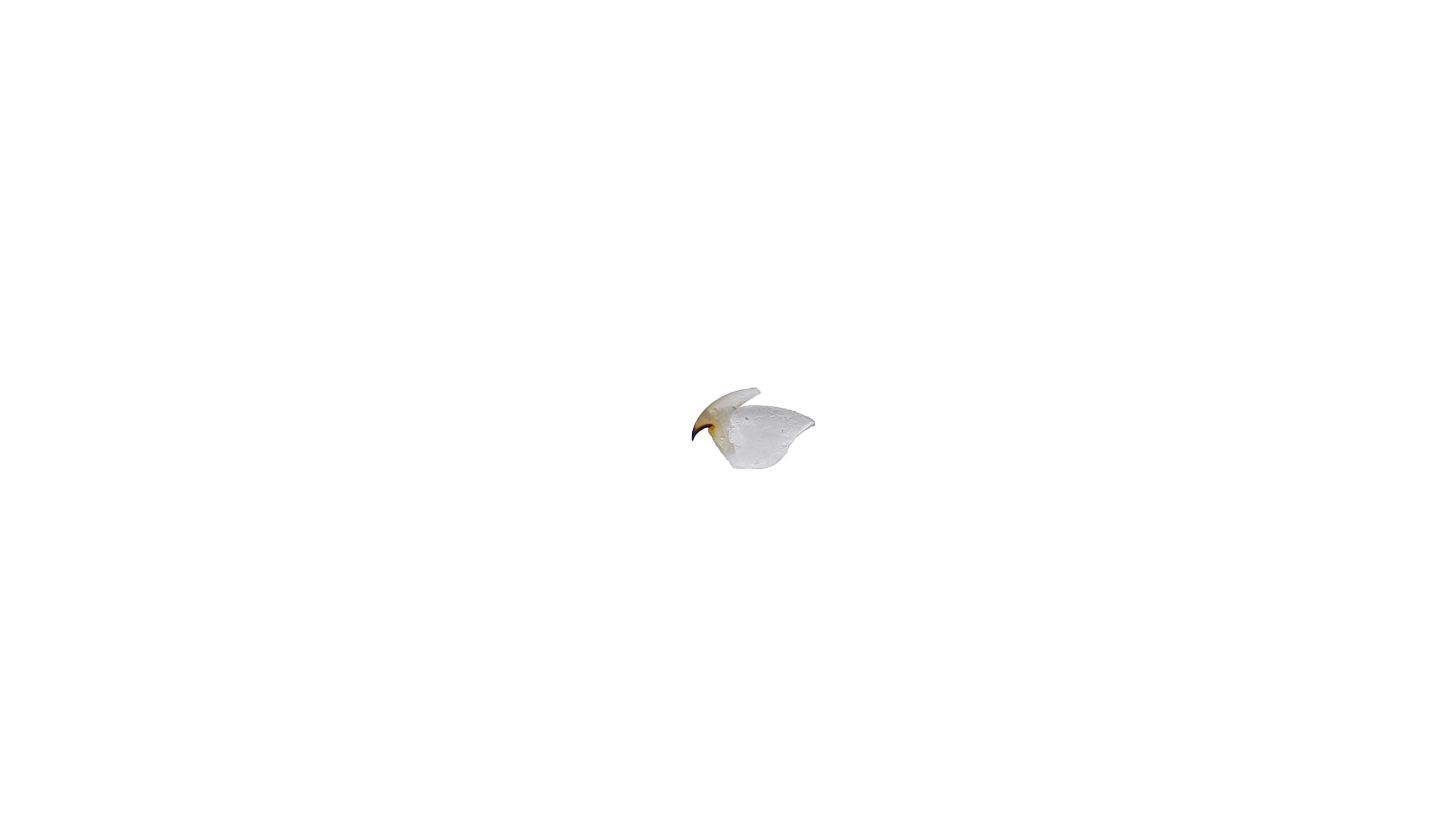

Supplement: Supplemental Information 2 — C2-Sepia aculeata, C3-Sepioteuthis lessoniana, C6-Sepia esculenta, O2-Amphioctopus aegina, S1-Loliolus uyii, S3-Uroteuthis chinensis, S4-Uroteuthis edulis [file peerj-09-11825-s002.zip › _Preprocessing_Upper_Beak/S1/U-l-S1-15.jpg]

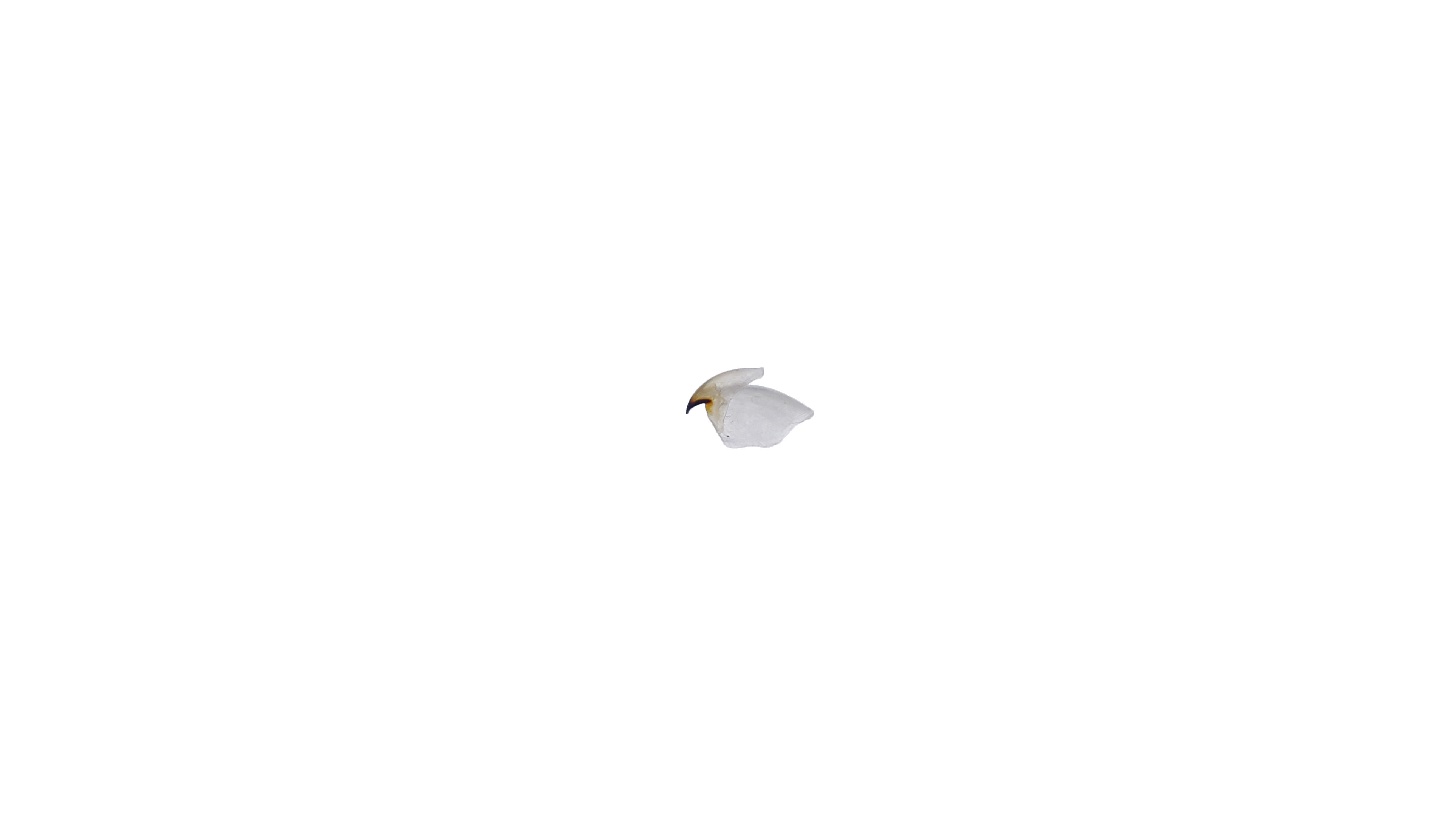

Supplement: Supplemental Information 2 — C2-Sepia aculeata, C3-Sepioteuthis lessoniana, C6-Sepia esculenta, O2-Amphioctopus aegina, S1-Loliolus uyii, S3-Uroteuthis chinensis, S4-Uroteuthis edulis [file peerj-09-11825-s002.zip › _Preprocessing_Upper_Beak/S1/U-l-S1-16.jpg]

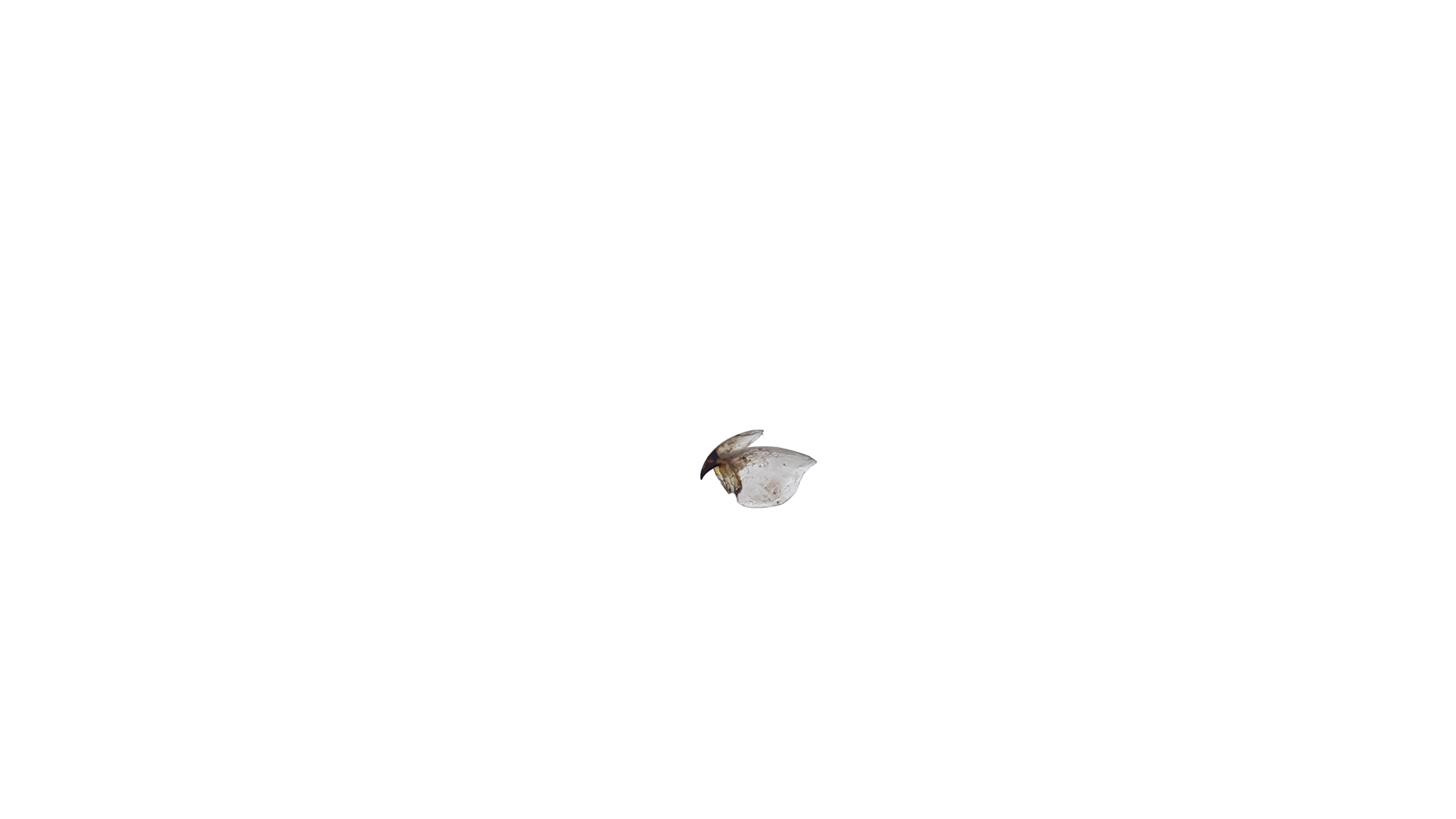

Supplement: Supplemental Information 2 — C2-Sepia aculeata, C3-Sepioteuthis lessoniana, C6-Sepia esculenta, O2-Amphioctopus aegina, S1-Loliolus uyii, S3-Uroteuthis chinensis, S4-Uroteuthis edulis [file peerj-09-11825-s002.zip › _Preprocessing_Upper_Beak/S1/U-l-S1-17.jpg]

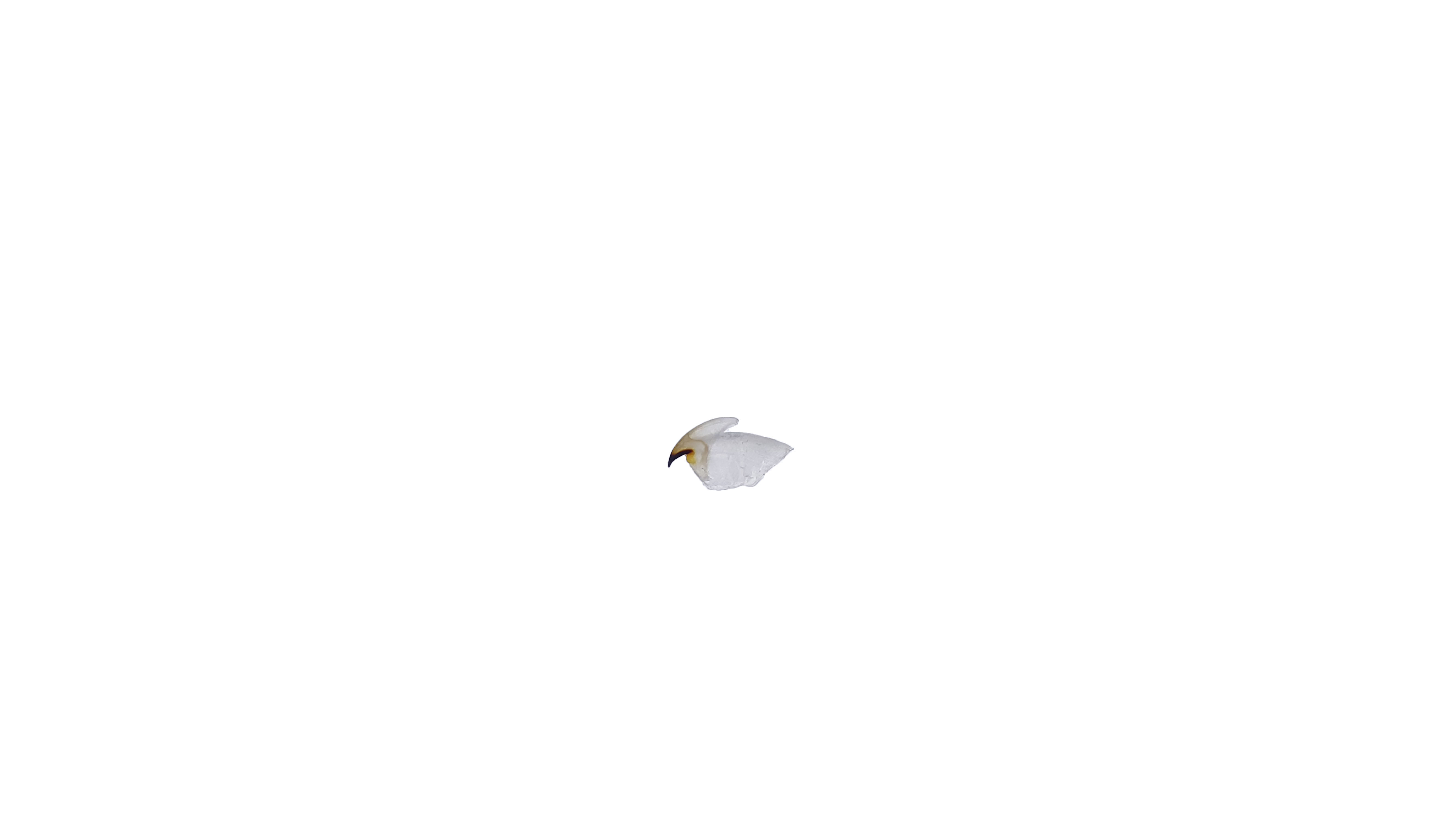

Supplement: Supplemental Information 2 — C2-Sepia aculeata, C3-Sepioteuthis lessoniana, C6-Sepia esculenta, O2-Amphioctopus aegina, S1-Loliolus uyii, S3-Uroteuthis chinensis, S4-Uroteuthis edulis [file peerj-09-11825-s002.zip › _Preprocessing_Upper_Beak/S1/U-l-S1-18.jpg]

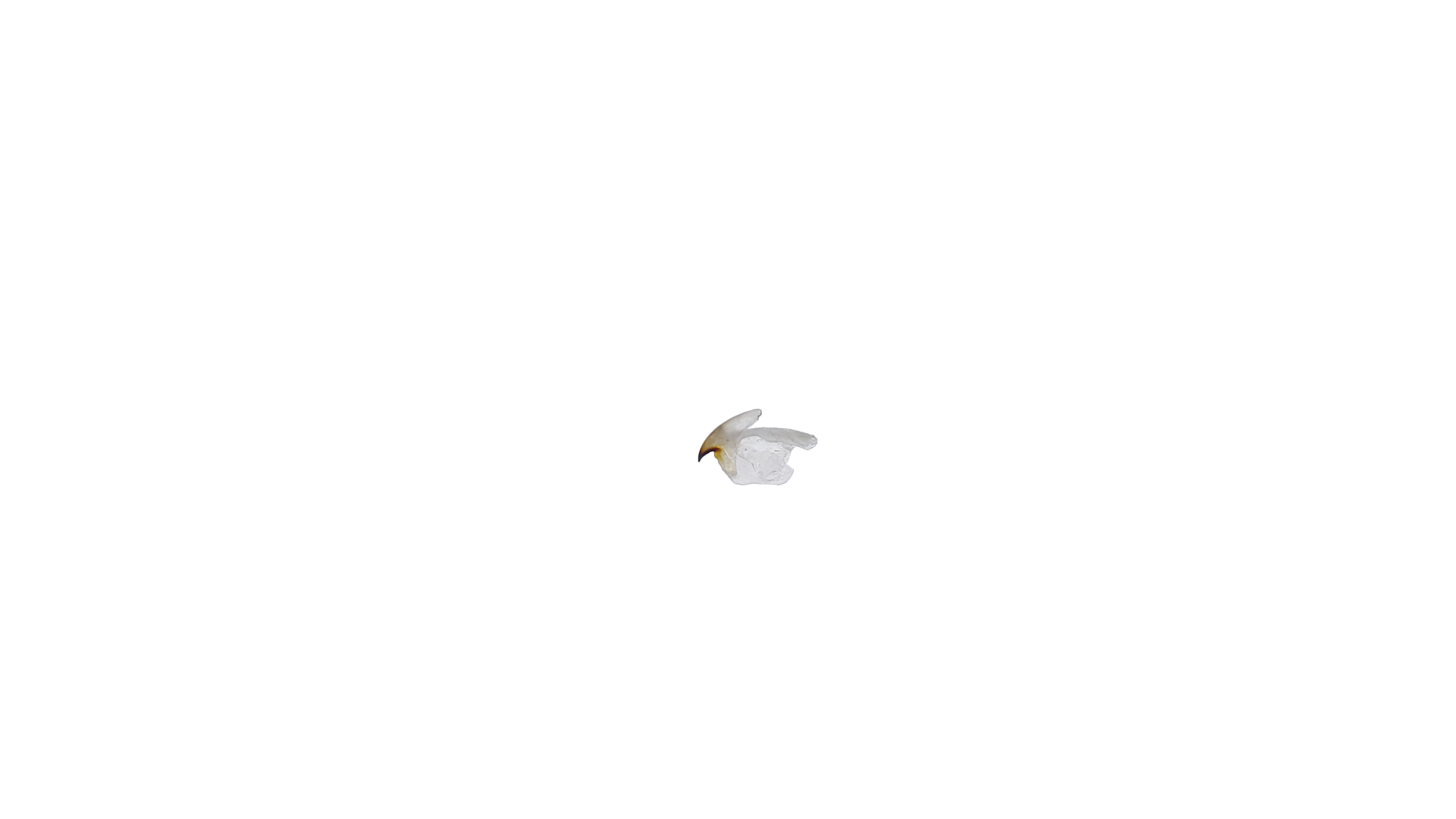

Supplement: Supplemental Information 2 — C2-Sepia aculeata, C3-Sepioteuthis lessoniana, C6-Sepia esculenta, O2-Amphioctopus aegina, S1-Loliolus uyii, S3-Uroteuthis chinensis, S4-Uroteuthis edulis [file peerj-09-11825-s002.zip › _Preprocessing_Upper_Beak/S1/U-l-S1-19.jpg]

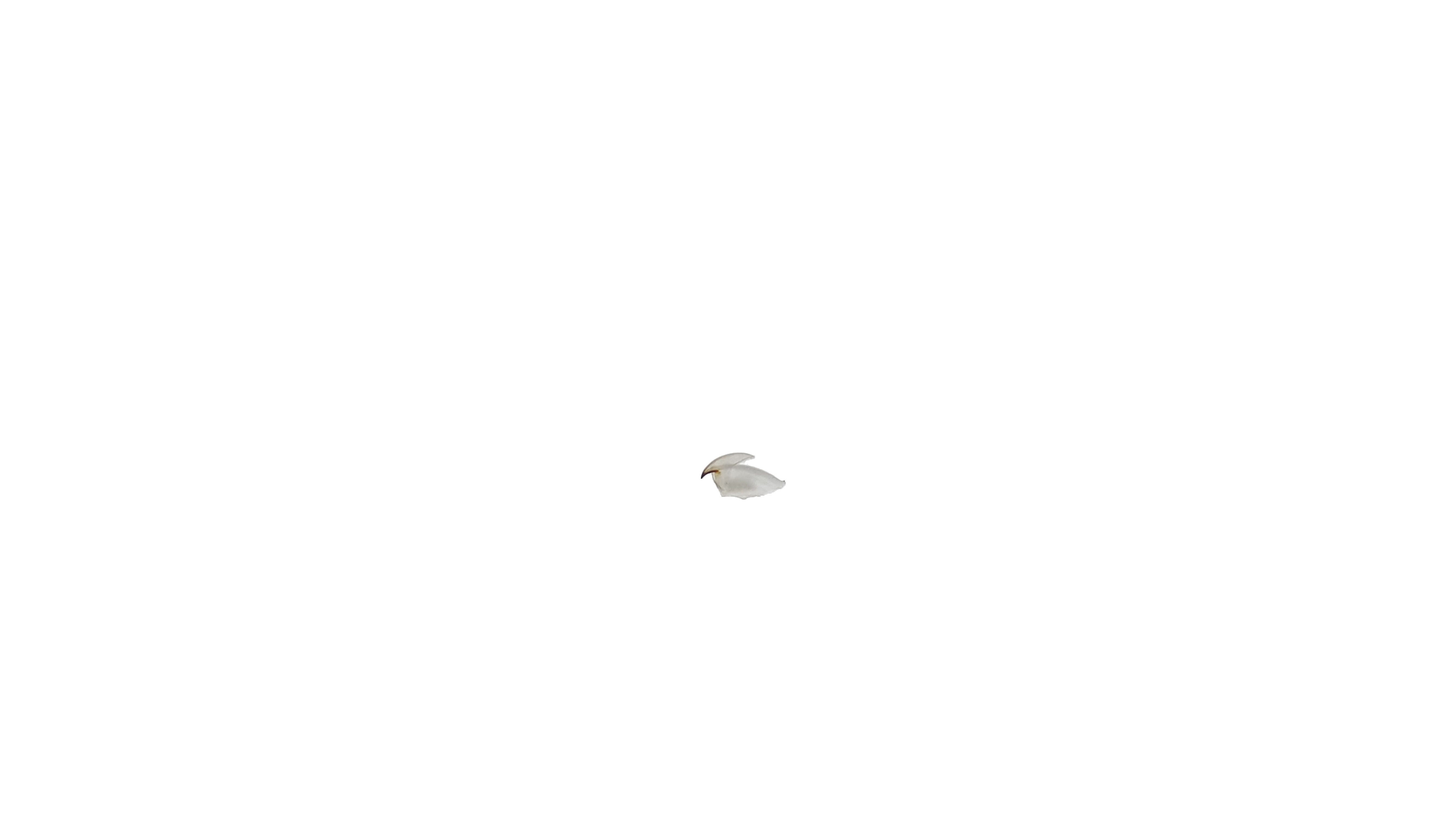

Supplement: Supplemental Information 2 — C2-Sepia aculeata, C3-Sepioteuthis lessoniana, C6-Sepia esculenta, O2-Amphioctopus aegina, S1-Loliolus uyii, S3-Uroteuthis chinensis, S4-Uroteuthis edulis [file peerj-09-11825-s002.zip › _Preprocessing_Upper_Beak/S1/U-l-S1-2.jpg]

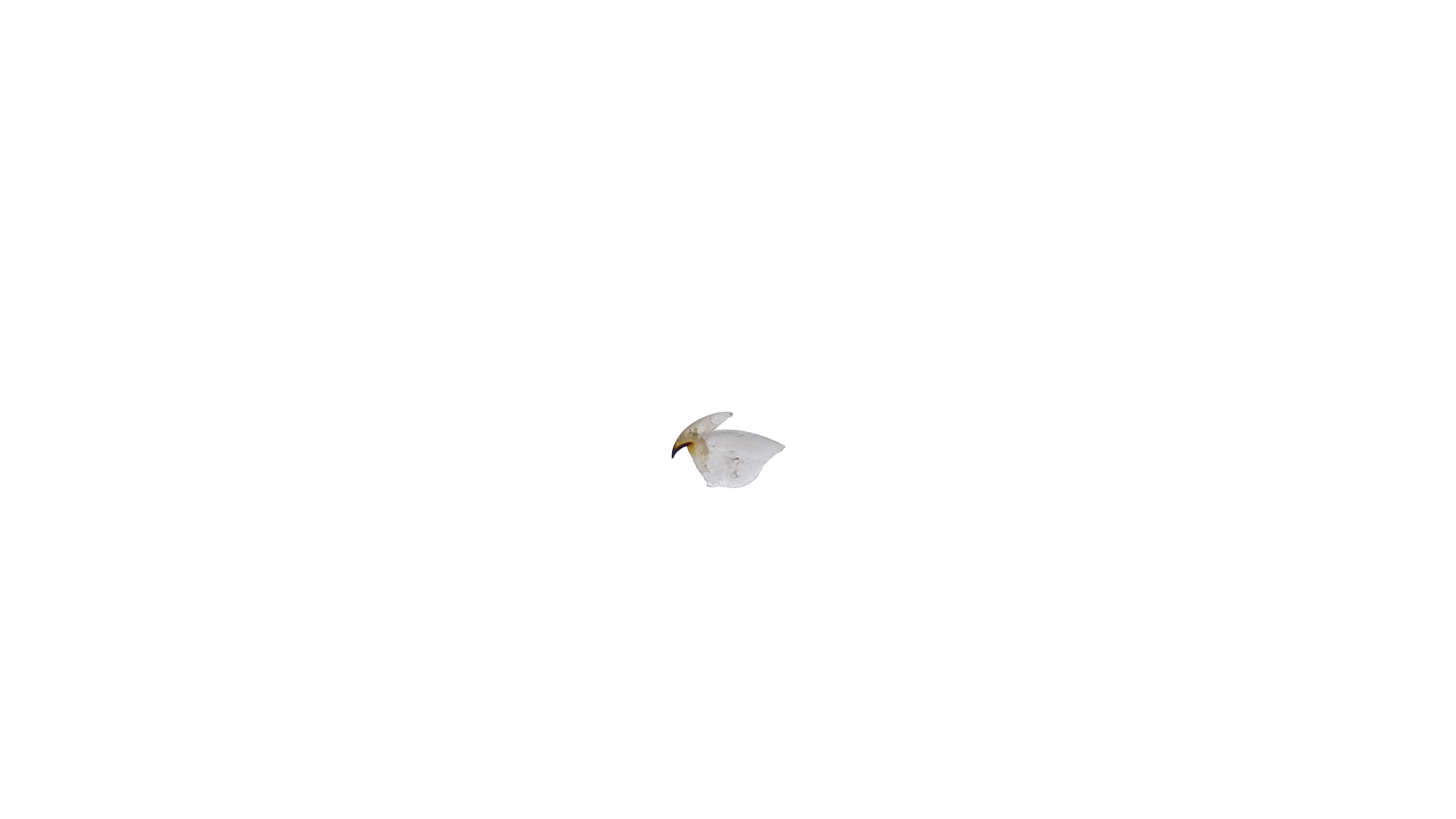

Supplement: Supplemental Information 2 — C2-Sepia aculeata, C3-Sepioteuthis lessoniana, C6-Sepia esculenta, O2-Amphioctopus aegina, S1-Loliolus uyii, S3-Uroteuthis chinensis, S4-Uroteuthis edulis [file peerj-09-11825-s002.zip › _Preprocessing_Upper_Beak/S1/U-l-S1-20.jpg]

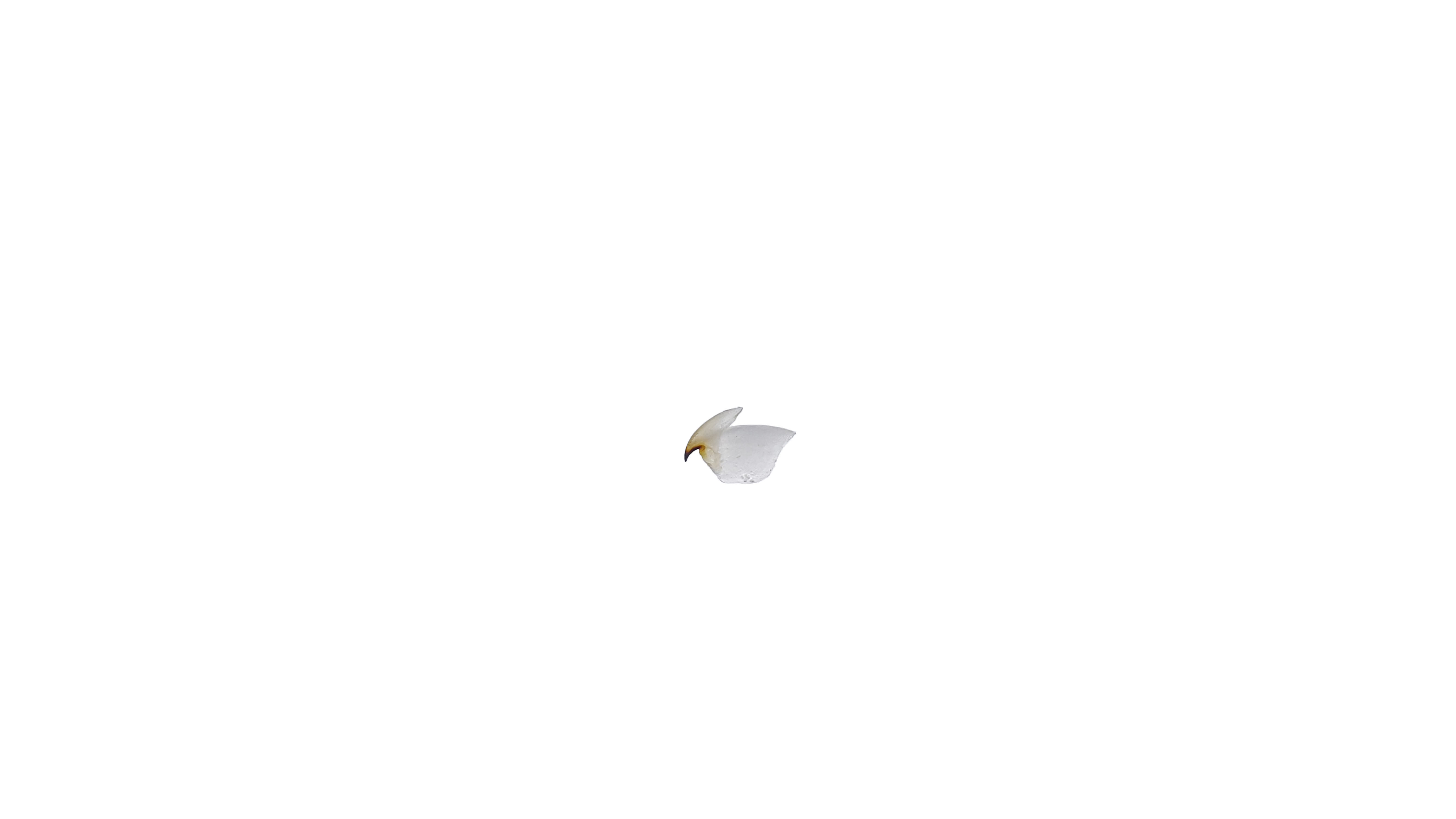

Supplement: Supplemental Information 2 — C2-Sepia aculeata, C3-Sepioteuthis lessoniana, C6-Sepia esculenta, O2-Amphioctopus aegina, S1-Loliolus uyii, S3-Uroteuthis chinensis, S4-Uroteuthis edulis [file peerj-09-11825-s002.zip › _Preprocessing_Upper_Beak/S1/U-l-S1-21.jpg]

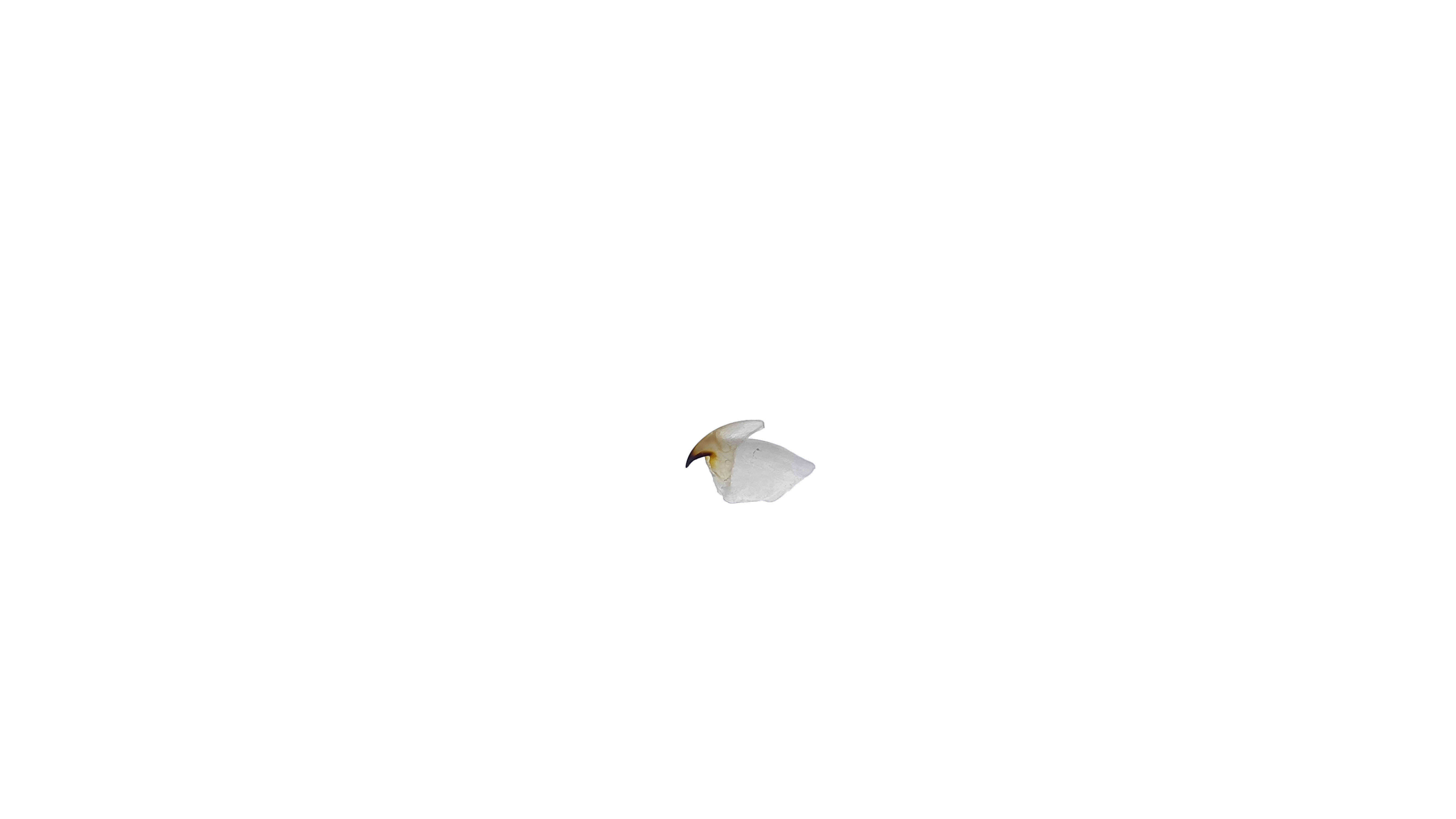

Supplement: Supplemental Information 2 — C2-Sepia aculeata, C3-Sepioteuthis lessoniana, C6-Sepia esculenta, O2-Amphioctopus aegina, S1-Loliolus uyii, S3-Uroteuthis chinensis, S4-Uroteuthis edulis [file peerj-09-11825-s002.zip › _Preprocessing_Upper_Beak/S1/U-l-S1-22.jpg]

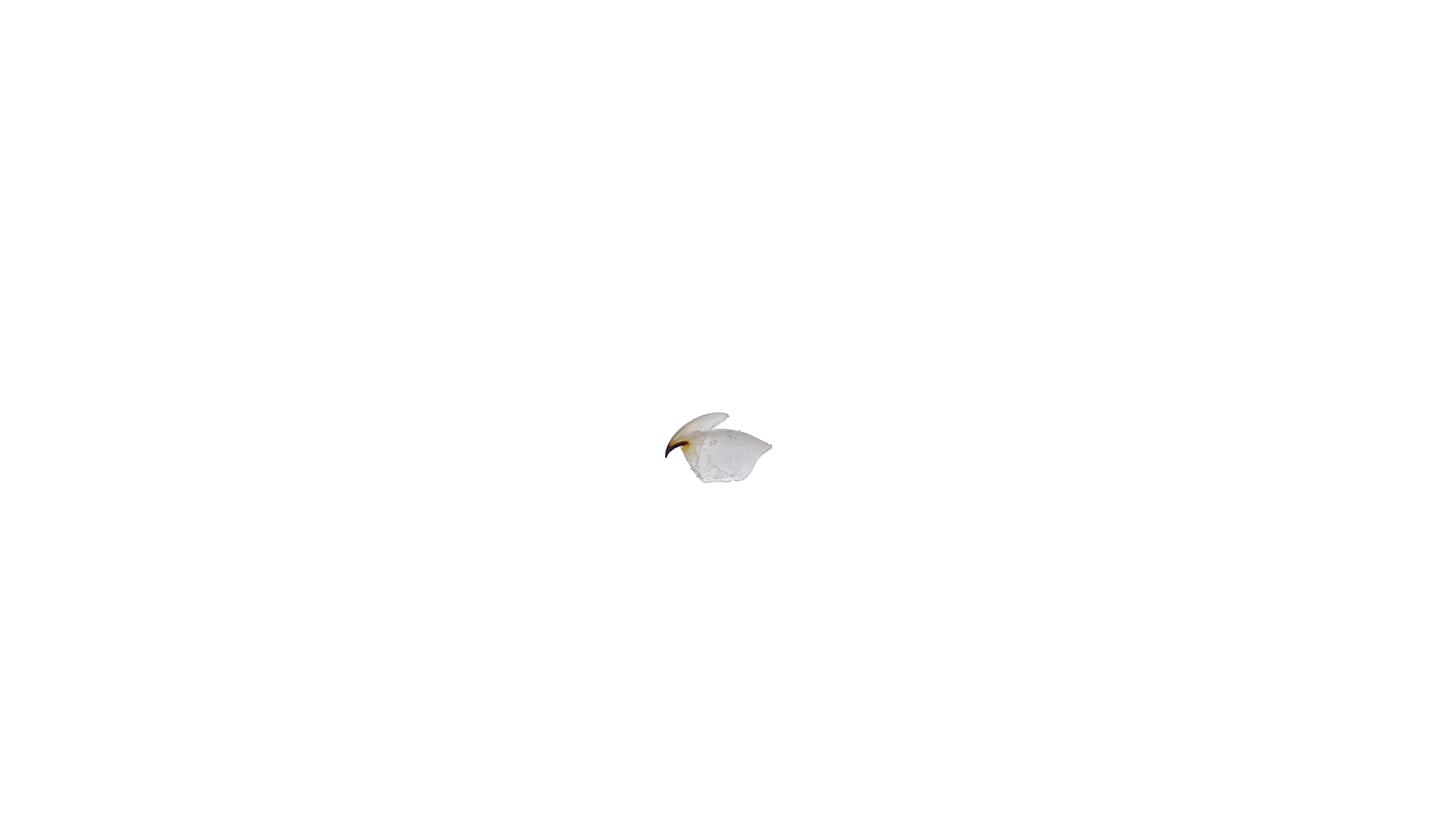

Supplement: Supplemental Information 2 — C2-Sepia aculeata, C3-Sepioteuthis lessoniana, C6-Sepia esculenta, O2-Amphioctopus aegina, S1-Loliolus uyii, S3-Uroteuthis chinensis, S4-Uroteuthis edulis [file peerj-09-11825-s002.zip › _Preprocessing_Upper_Beak/S1/U-l-S1-23.jpg]

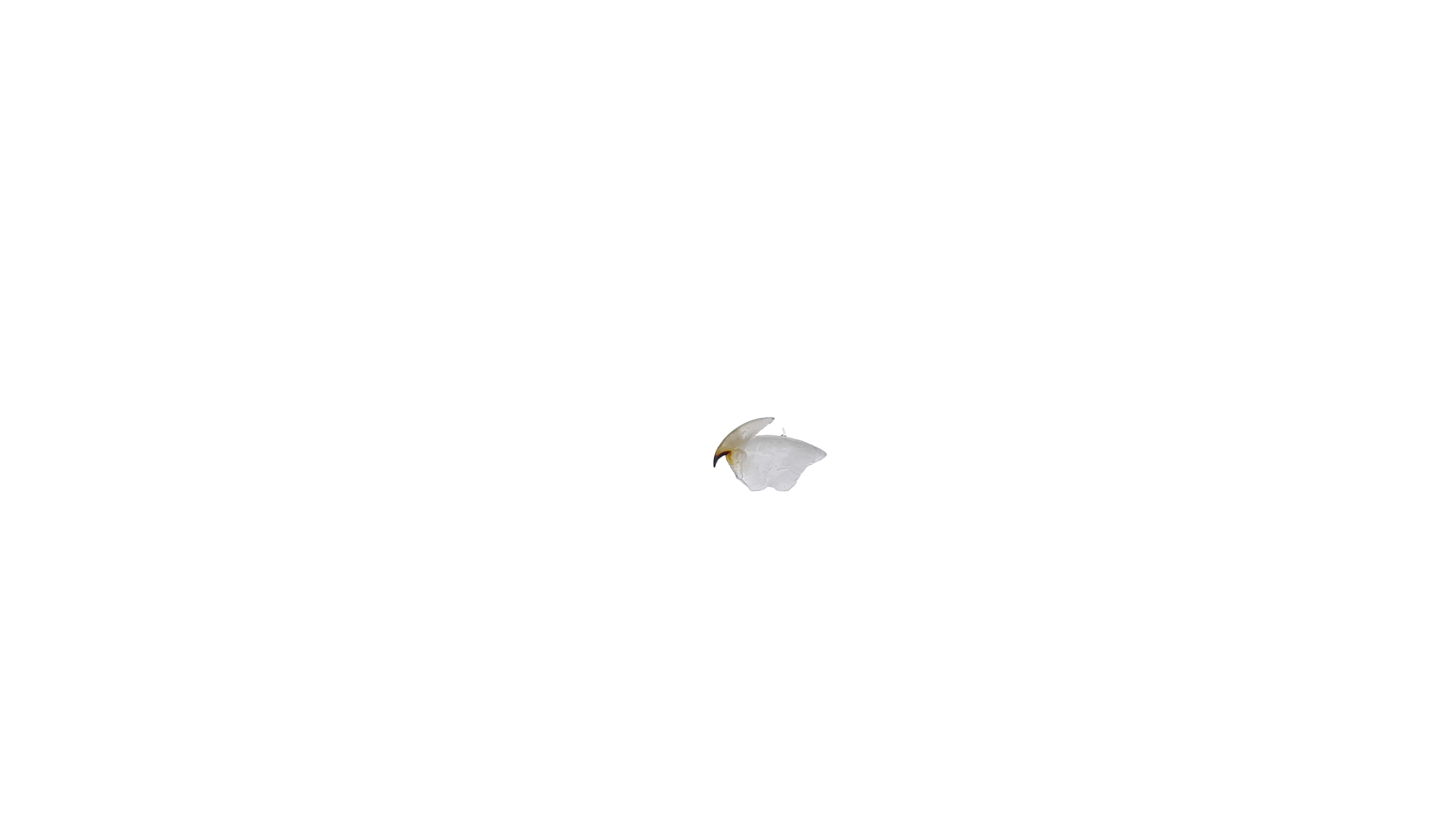

Supplement: Supplemental Information 2 — C2-Sepia aculeata, C3-Sepioteuthis lessoniana, C6-Sepia esculenta, O2-Amphioctopus aegina, S1-Loliolus uyii, S3-Uroteuthis chinensis, S4-Uroteuthis edulis [file peerj-09-11825-s002.zip › _Preprocessing_Upper_Beak/S1/U-l-S1-24.jpg]

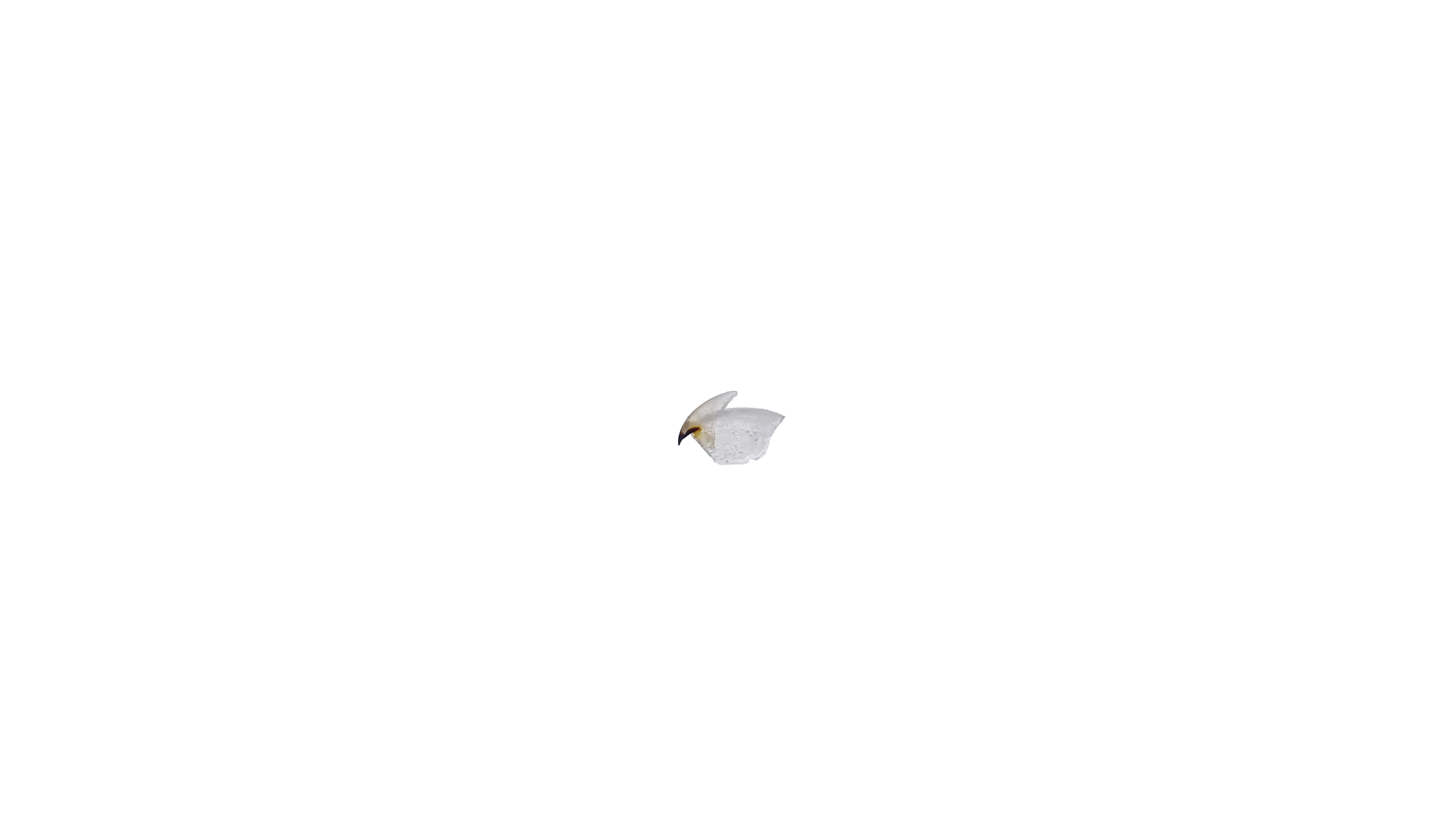

Supplement: Supplemental Information 2 — C2-Sepia aculeata, C3-Sepioteuthis lessoniana, C6-Sepia esculenta, O2-Amphioctopus aegina, S1-Loliolus uyii, S3-Uroteuthis chinensis, S4-Uroteuthis edulis [file peerj-09-11825-s002.zip › _Preprocessing_Upper_Beak/S1/U-l-S1-25.jpg]

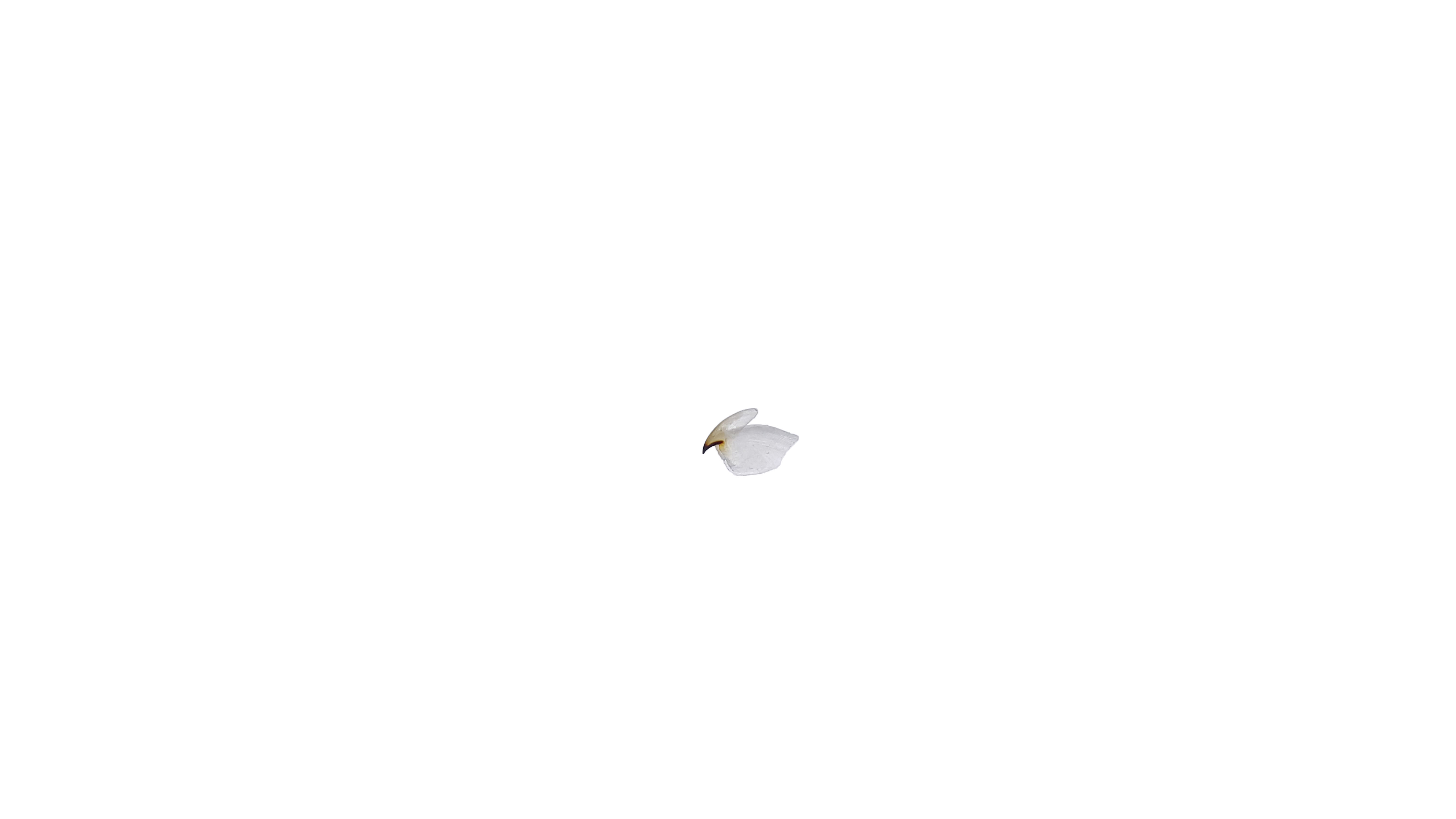

Supplement: Supplemental Information 2 — C2-Sepia aculeata, C3-Sepioteuthis lessoniana, C6-Sepia esculenta, O2-Amphioctopus aegina, S1-Loliolus uyii, S3-Uroteuthis chinensis, S4-Uroteuthis edulis [file peerj-09-11825-s002.zip › _Preprocessing_Upper_Beak/S1/U-l-S1-26.jpg]

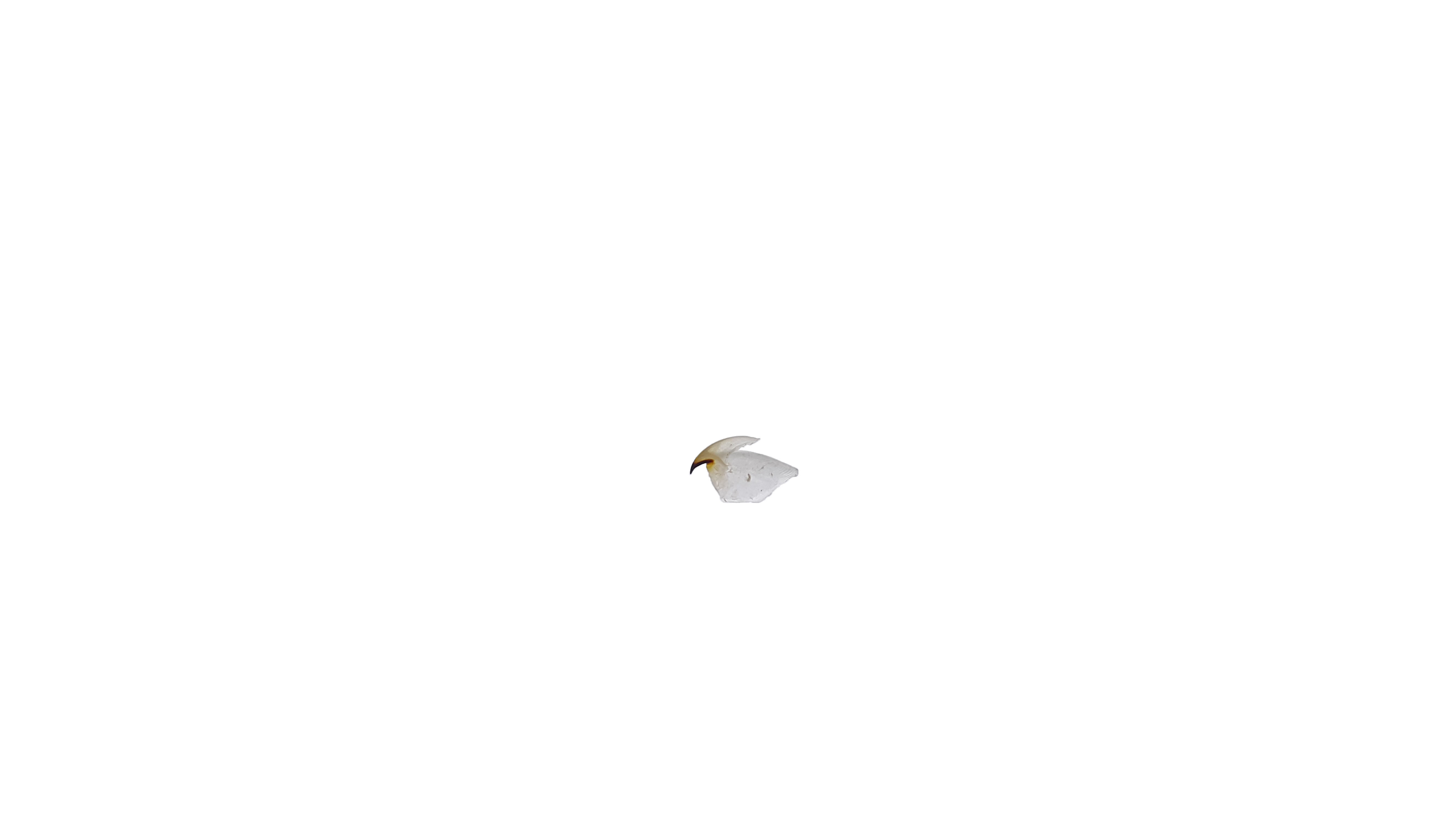

Supplement: Supplemental Information 2 — C2-Sepia aculeata, C3-Sepioteuthis lessoniana, C6-Sepia esculenta, O2-Amphioctopus aegina, S1-Loliolus uyii, S3-Uroteuthis chinensis, S4-Uroteuthis edulis [file peerj-09-11825-s002.zip › _Preprocessing_Upper_Beak/S1/U-l-S1-27.jpg]

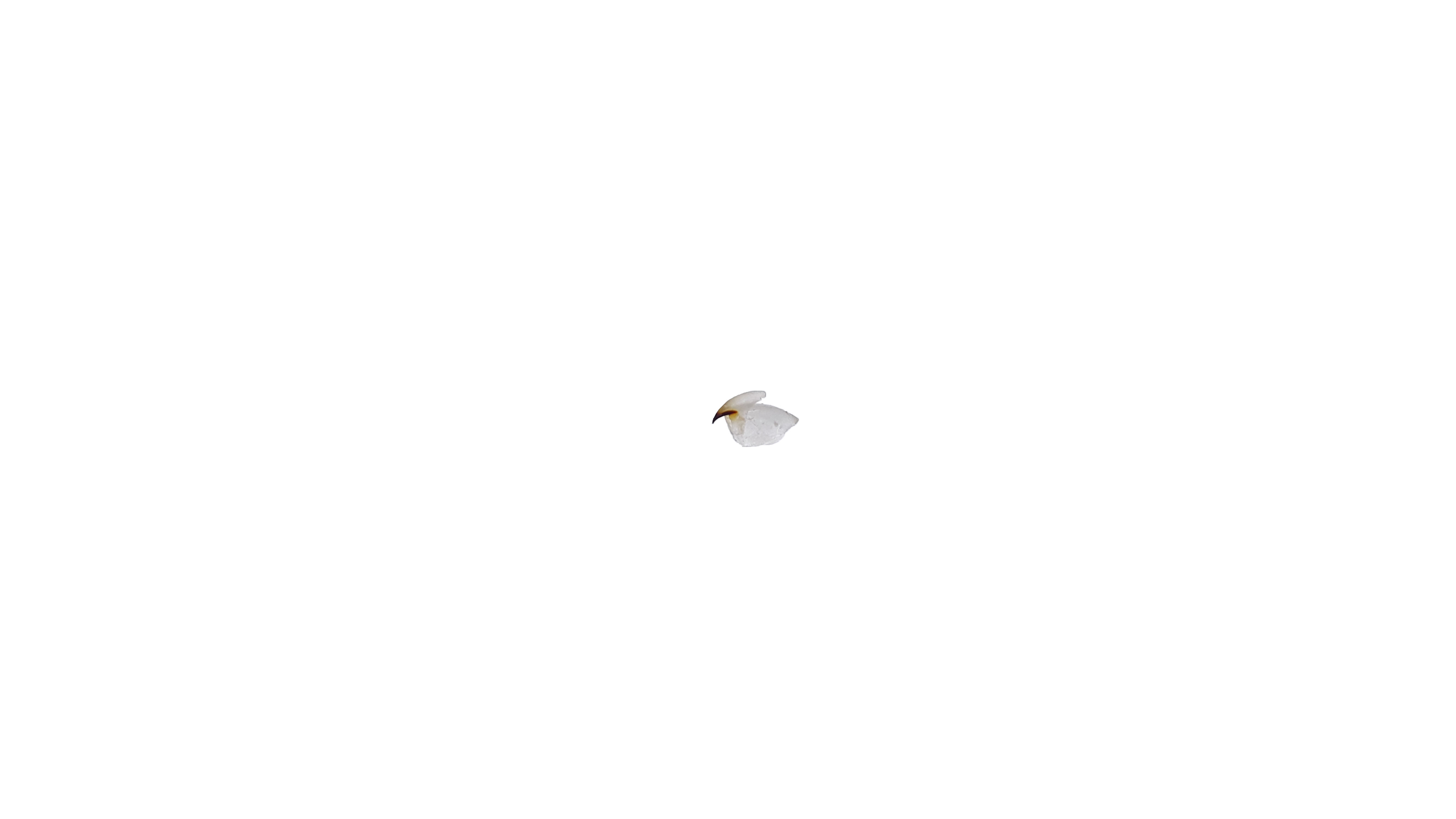

Supplement: Supplemental Information 2 — C2-Sepia aculeata, C3-Sepioteuthis lessoniana, C6-Sepia esculenta, O2-Amphioctopus aegina, S1-Loliolus uyii, S3-Uroteuthis chinensis, S4-Uroteuthis edulis [file peerj-09-11825-s002.zip › _Preprocessing_Upper_Beak/S1/U-l-S1-28.jpg]

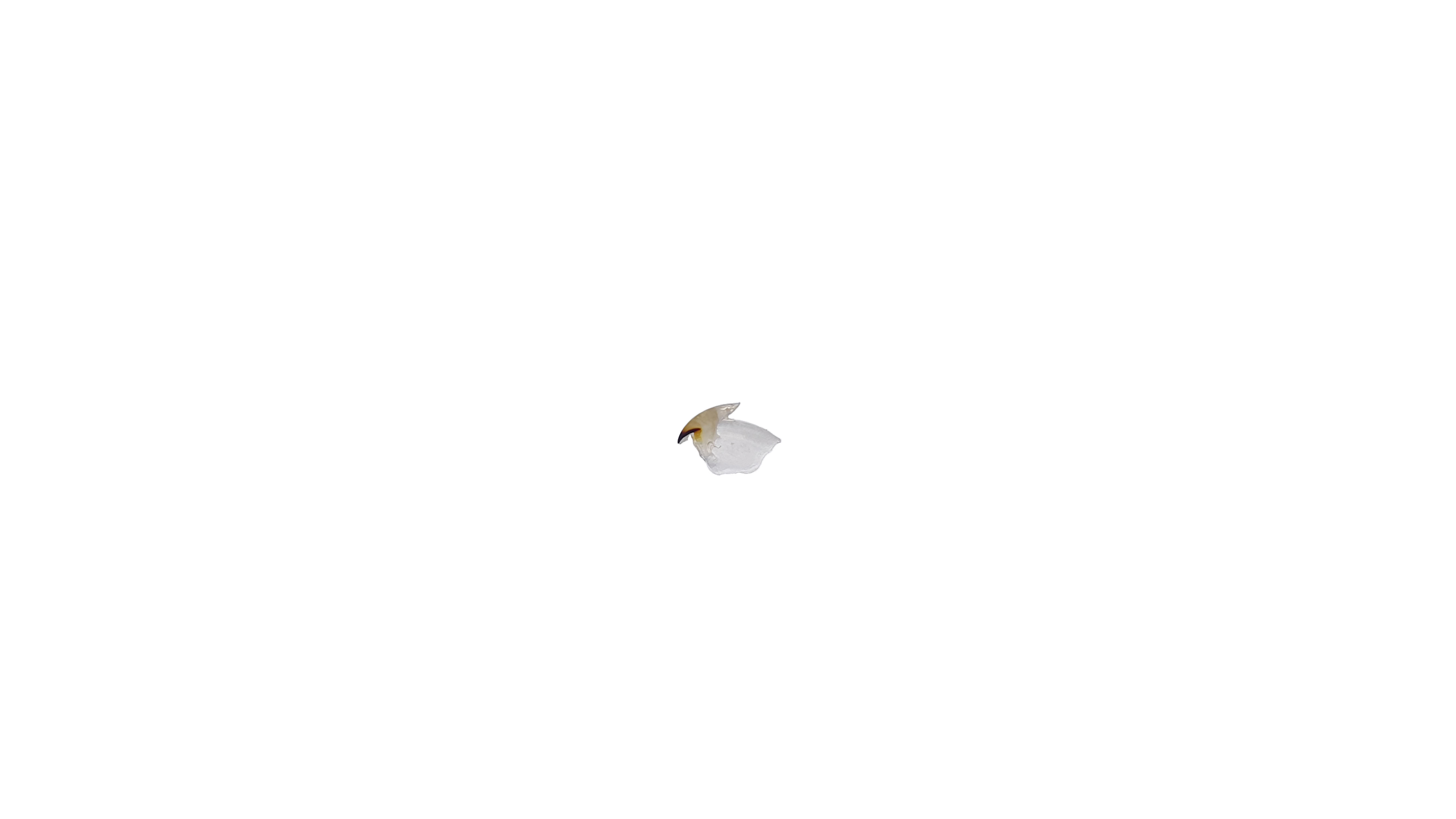

Supplement: Supplemental Information 2 — C2-Sepia aculeata, C3-Sepioteuthis lessoniana, C6-Sepia esculenta, O2-Amphioctopus aegina, S1-Loliolus uyii, S3-Uroteuthis chinensis, S4-Uroteuthis edulis [file peerj-09-11825-s002.zip › _Preprocessing_Upper_Beak/S1/U-l-S1-29.jpg]

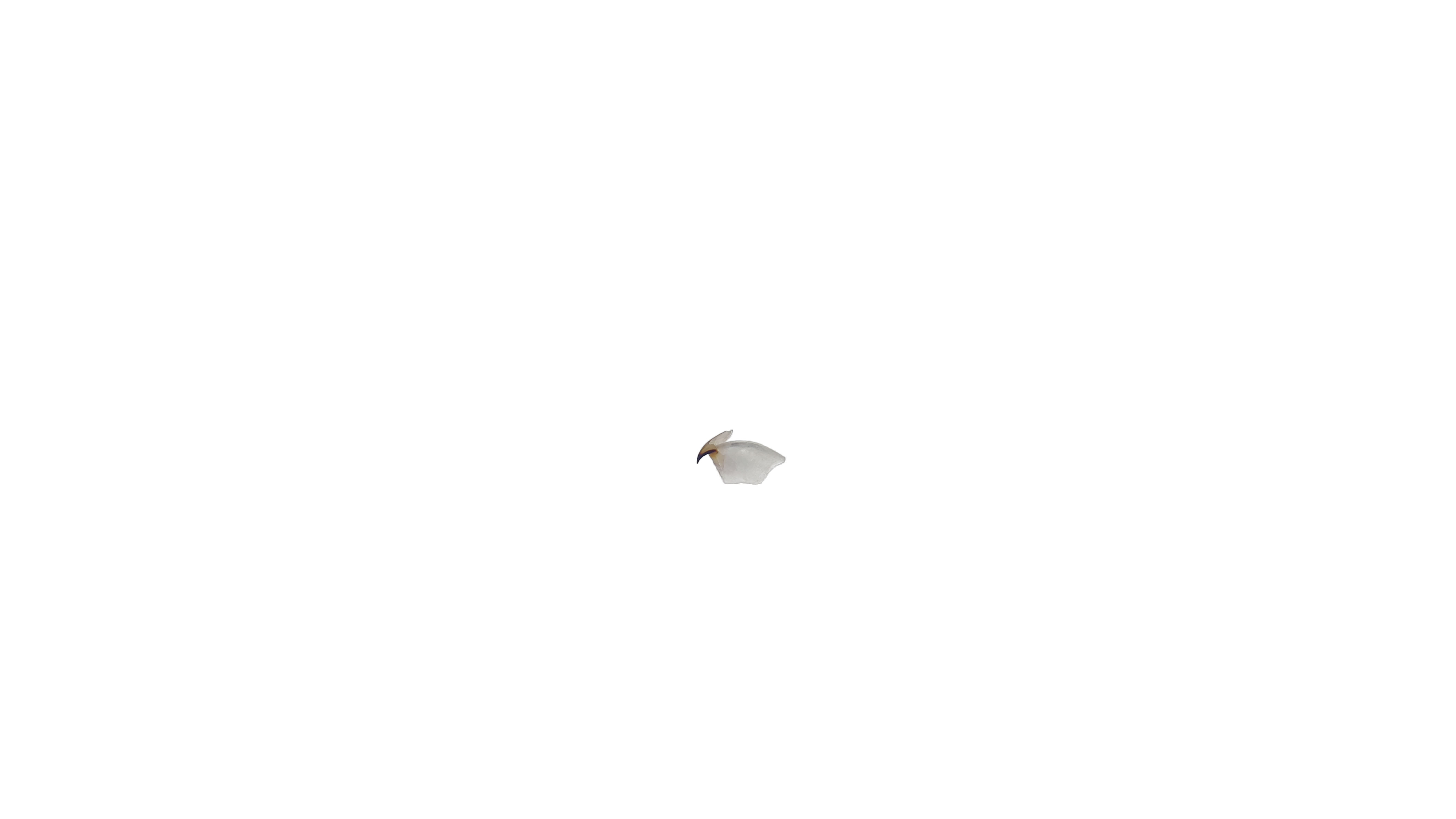

Supplement: Supplemental Information 2 — C2-Sepia aculeata, C3-Sepioteuthis lessoniana, C6-Sepia esculenta, O2-Amphioctopus aegina, S1-Loliolus uyii, S3-Uroteuthis chinensis, S4-Uroteuthis edulis [file peerj-09-11825-s002.zip › _Preprocessing_Upper_Beak/S1/U-l-S1-3.jpg]

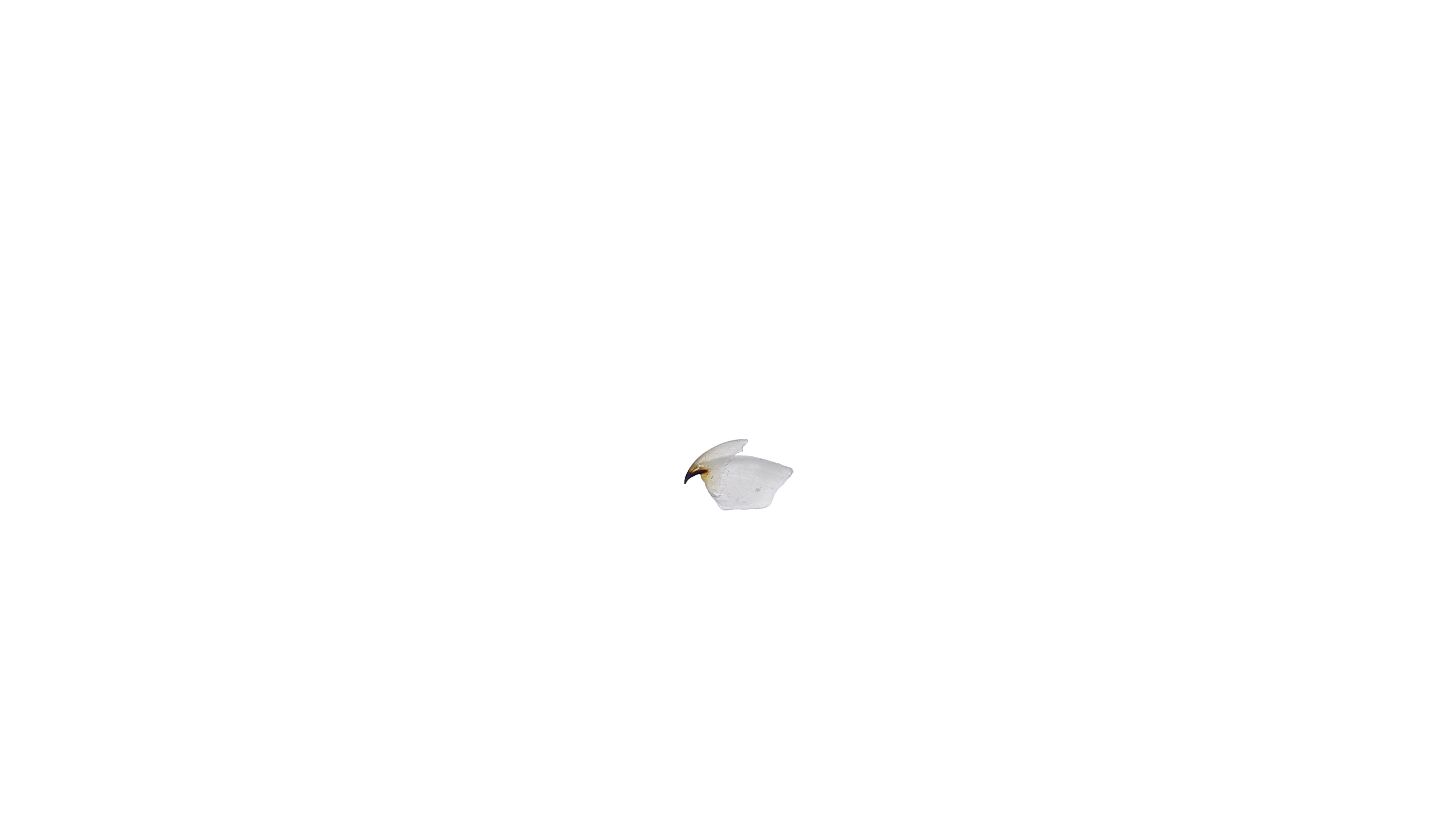

Supplement: Supplemental Information 2 — C2-Sepia aculeata, C3-Sepioteuthis lessoniana, C6-Sepia esculenta, O2-Amphioctopus aegina, S1-Loliolus uyii, S3-Uroteuthis chinensis, S4-Uroteuthis edulis [file peerj-09-11825-s002.zip › _Preprocessing_Upper_Beak/S1/U-l-S1-30.jpg]

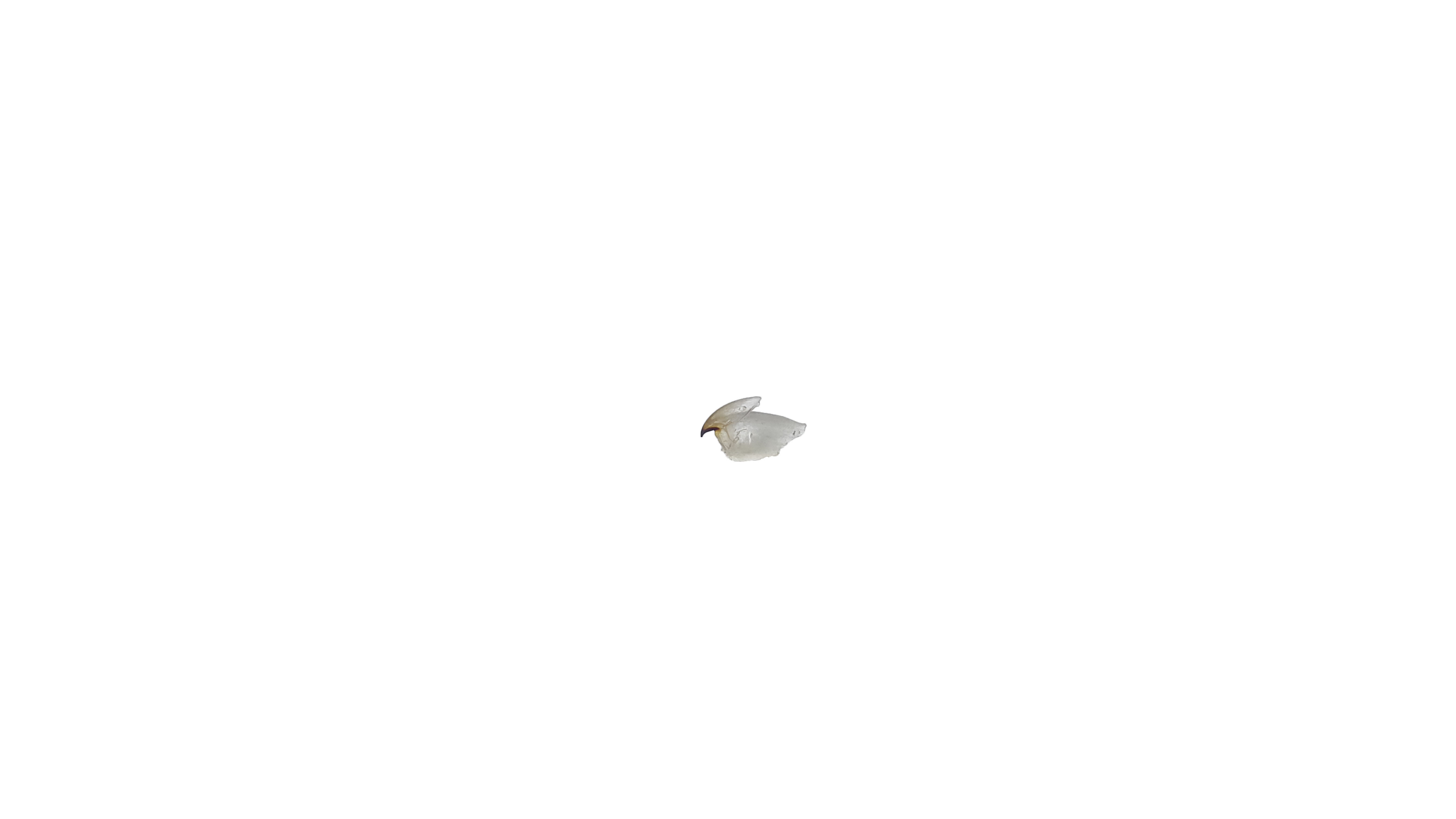

Supplement: Supplemental Information 2 — C2-Sepia aculeata, C3-Sepioteuthis lessoniana, C6-Sepia esculenta, O2-Amphioctopus aegina, S1-Loliolus uyii, S3-Uroteuthis chinensis, S4-Uroteuthis edulis [file peerj-09-11825-s002.zip › _Preprocessing_Upper_Beak/S1/U-l-S1-31.jpg]

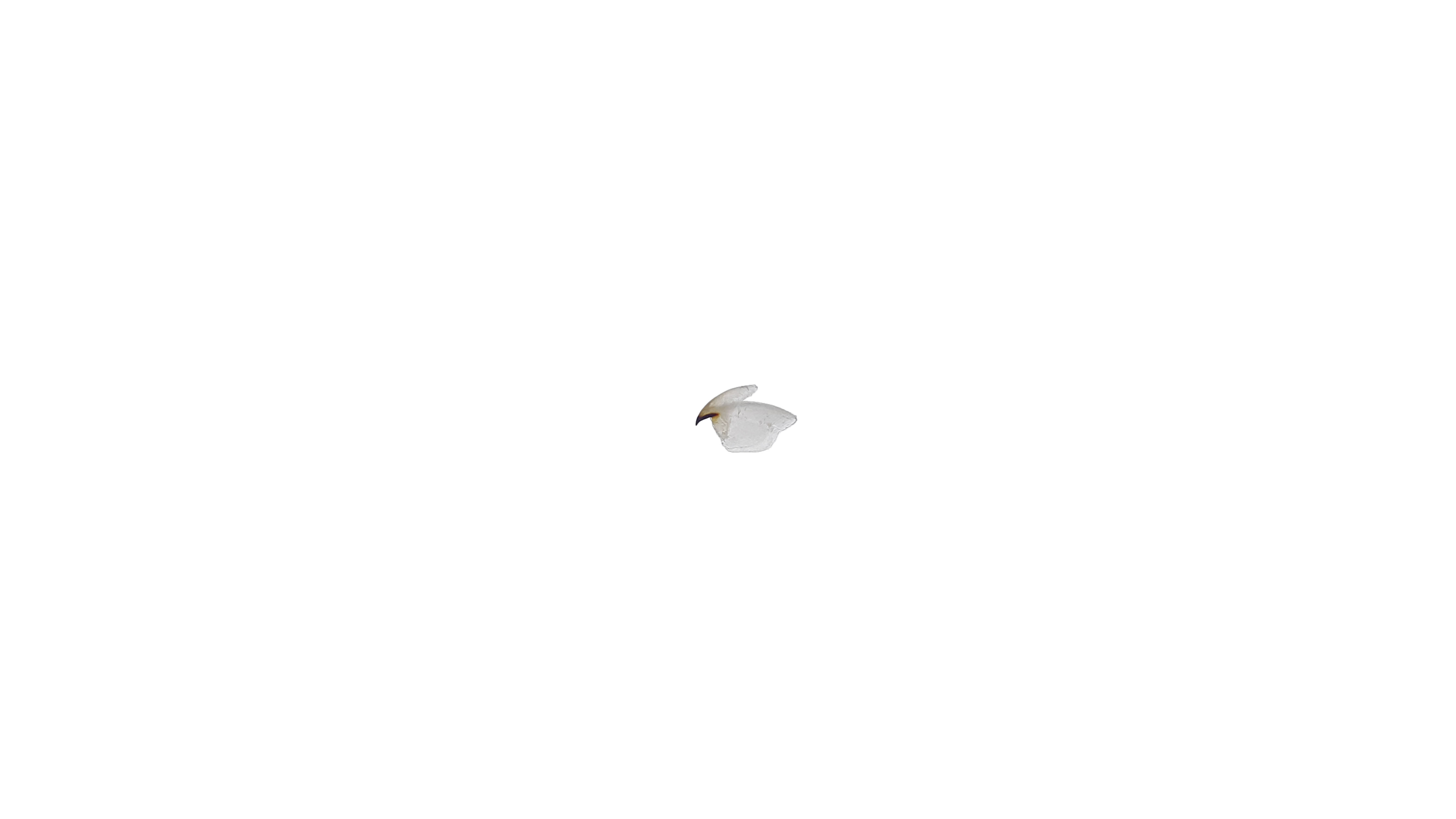

Supplement: Supplemental Information 2 — C2-Sepia aculeata, C3-Sepioteuthis lessoniana, C6-Sepia esculenta, O2-Amphioctopus aegina, S1-Loliolus uyii, S3-Uroteuthis chinensis, S4-Uroteuthis edulis [file peerj-09-11825-s002.zip › _Preprocessing_Upper_Beak/S1/U-l-S1-32.jpg]

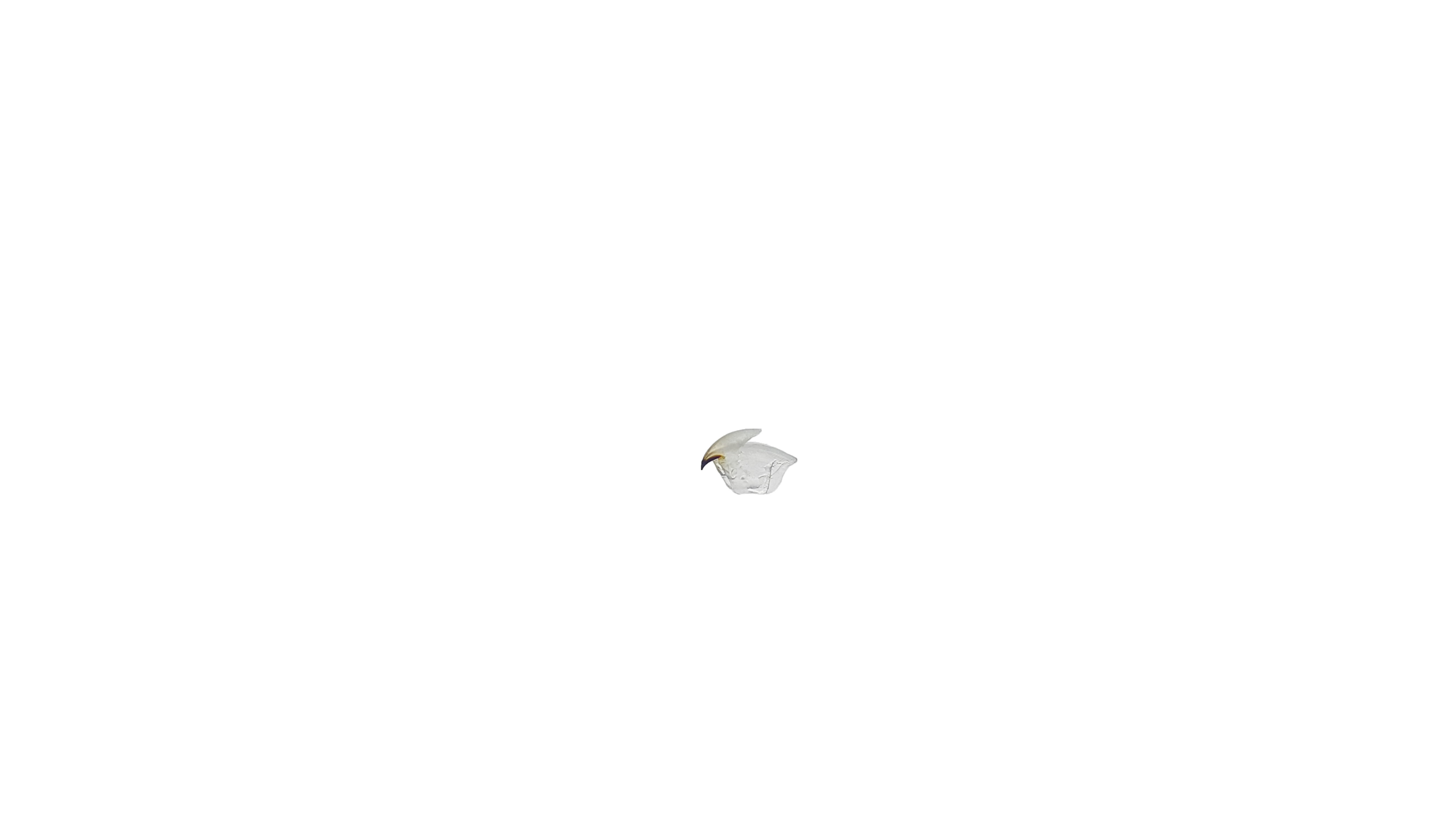

Supplement: Supplemental Information 2 — C2-Sepia aculeata, C3-Sepioteuthis lessoniana, C6-Sepia esculenta, O2-Amphioctopus aegina, S1-Loliolus uyii, S3-Uroteuthis chinensis, S4-Uroteuthis edulis [file peerj-09-11825-s002.zip › _Preprocessing_Upper_Beak/S1/U-l-S1-4.jpg]

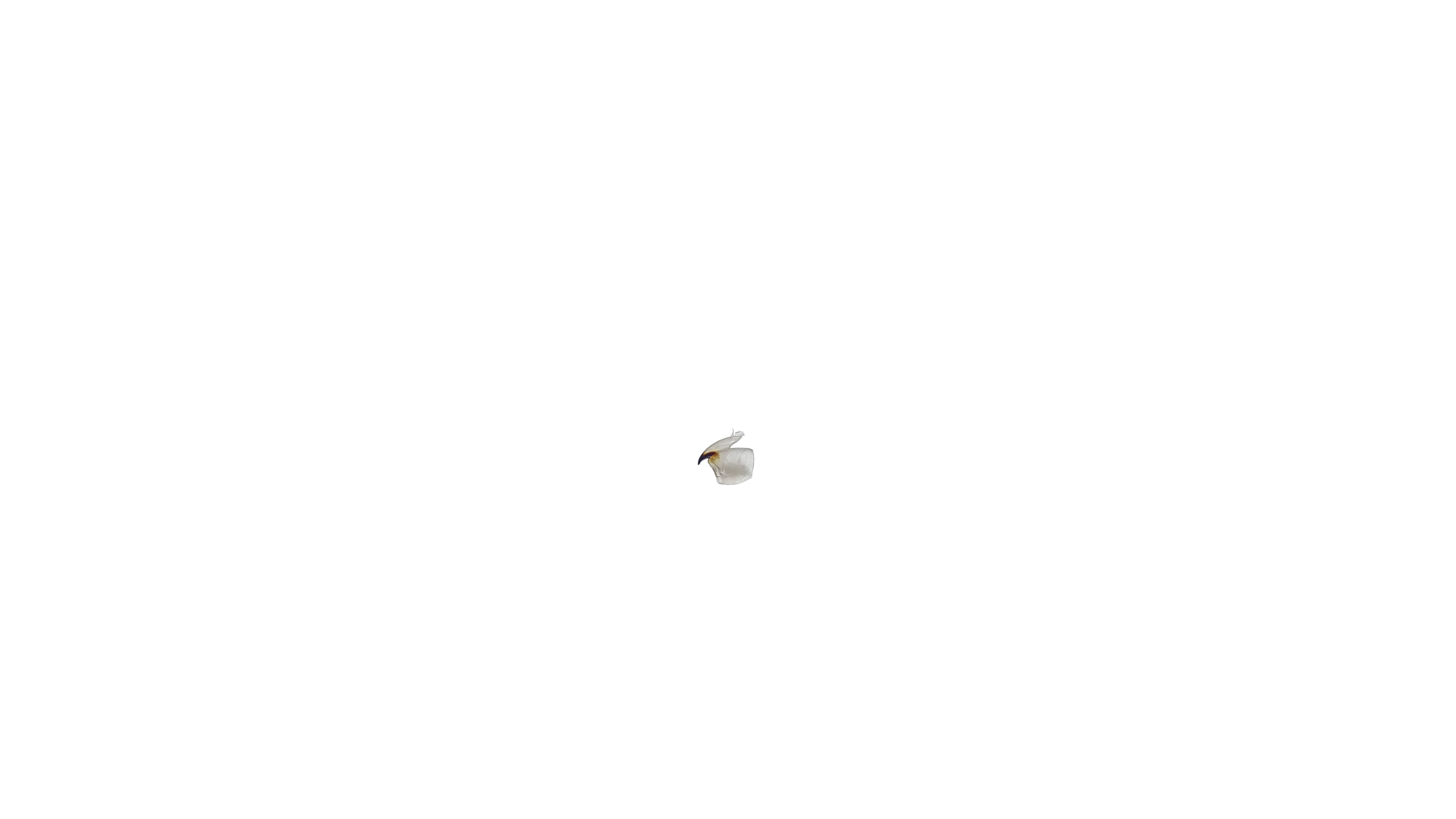

Supplement: Supplemental Information 2 — C2-Sepia aculeata, C3-Sepioteuthis lessoniana, C6-Sepia esculenta, O2-Amphioctopus aegina, S1-Loliolus uyii, S3-Uroteuthis chinensis, S4-Uroteuthis edulis [file peerj-09-11825-s002.zip › _Preprocessing_Upper_Beak/S1/U-l-S1-5.jpg]

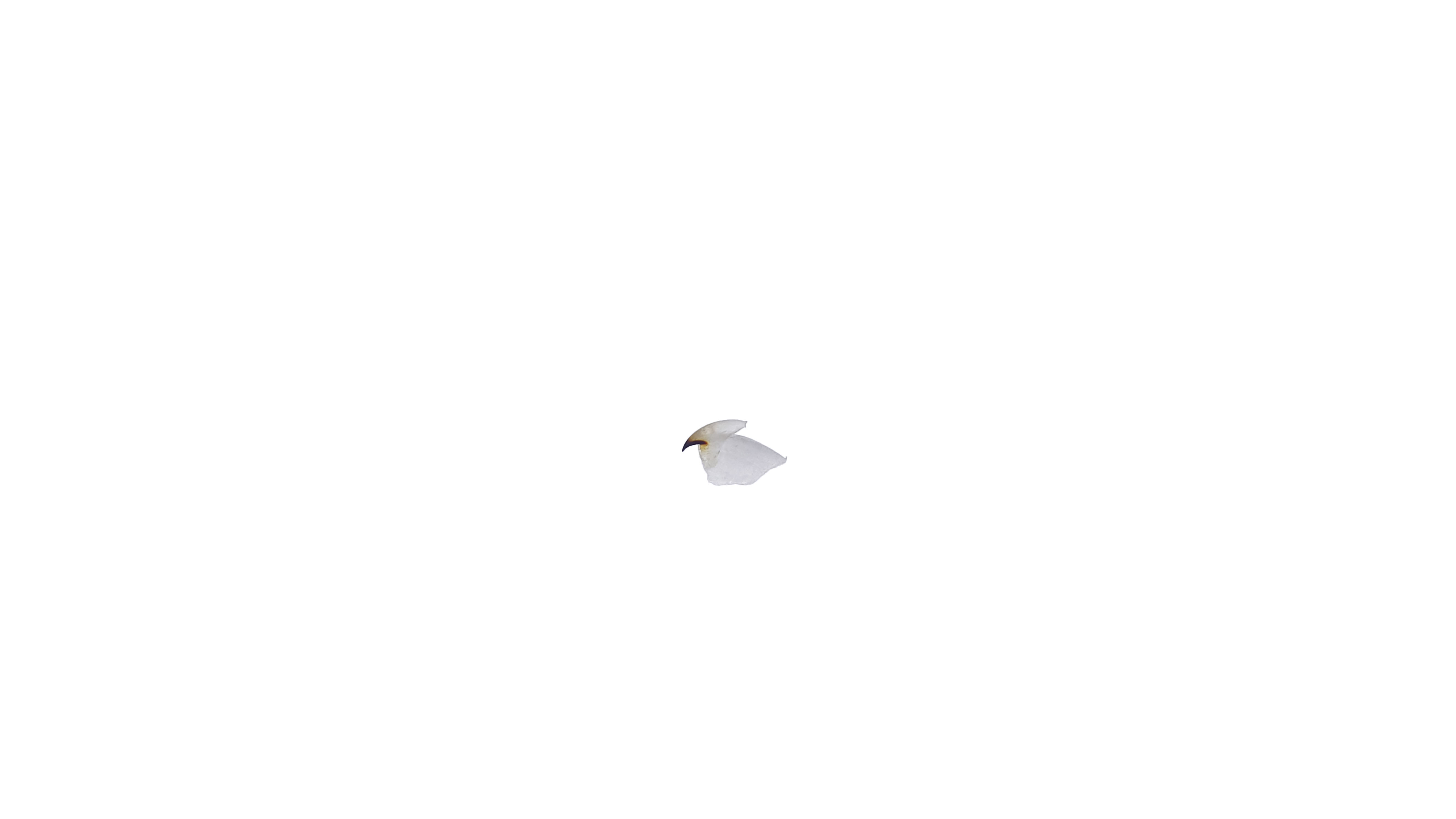

Supplement: Supplemental Information 2 — C2-Sepia aculeata, C3-Sepioteuthis lessoniana, C6-Sepia esculenta, O2-Amphioctopus aegina, S1-Loliolus uyii, S3-Uroteuthis chinensis, S4-Uroteuthis edulis [file peerj-09-11825-s002.zip › _Preprocessing_Upper_Beak/S1/U-l-S1-6.jpg]

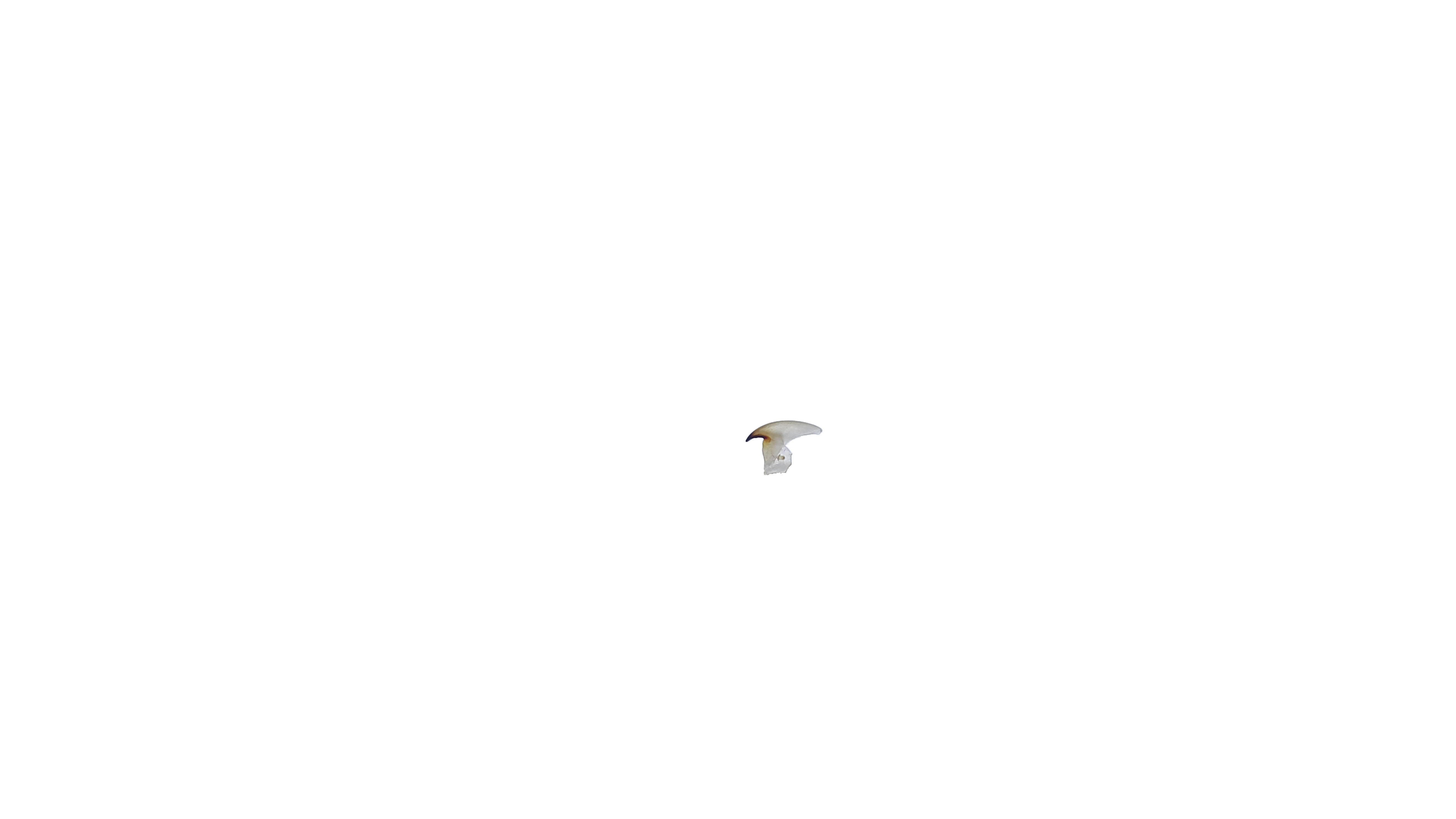

Supplement: Supplemental Information 2 — C2-Sepia aculeata, C3-Sepioteuthis lessoniana, C6-Sepia esculenta, O2-Amphioctopus aegina, S1-Loliolus uyii, S3-Uroteuthis chinensis, S4-Uroteuthis edulis [file peerj-09-11825-s002.zip › _Preprocessing_Upper_Beak/S1/U-l-S1-7.jpg]

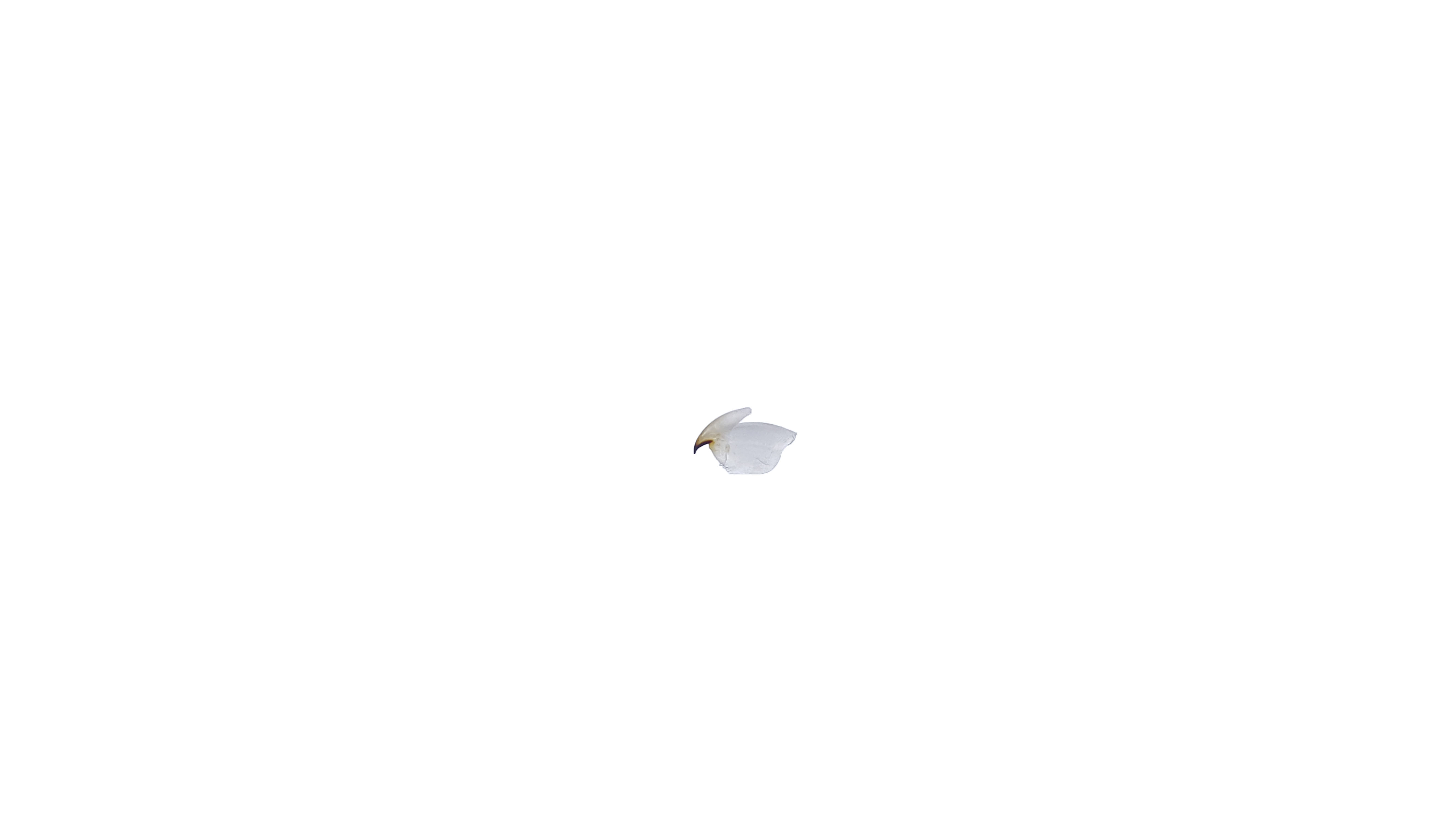

Supplement: Supplemental Information 2 — C2-Sepia aculeata, C3-Sepioteuthis lessoniana, C6-Sepia esculenta, O2-Amphioctopus aegina, S1-Loliolus uyii, S3-Uroteuthis chinensis, S4-Uroteuthis edulis [file peerj-09-11825-s002.zip › _Preprocessing_Upper_Beak/S1/U-l-S1-8.jpg]
